# Real-world assessment and treatment of locally advanced basal cell carcinoma: Findings from the RegiSONIC disease registry

**DOI:** 10.1371/journal.pone.0262151

**Published:** 2022-01-14

**Authors:** Aleksandar Sekulic, Simon Yoo, Ragini Kudchadkar, Julie Guillen, Gary Rogers, Anne Lynn S. Chang, Scott Guenthner, Bernard Raskin, Keith Dawson, Yong Mun, Laura Chu, Edward McKenna, Mario Lacouture

**Affiliations:** 1 Dermatology, Mayo Clinic, Scottsdale, Arizona, United States of America; 2 Department of Dermatology, Feinberg School of Medicine, Northwestern University, Chicago, Illinois, United States of America; 3 Department of Hematology and Medical Oncology, Winship Cancer Institute, Emory University School of Medicine, Atlanta, Georgia, United States of America; 4 UCSF Dermatology and Laser Surgery Center, University of California, San Francisco, California, United States of America; 5 Surgical Dermatology, Tufts New England Medical Center, Boston, Massachusetts, United States of America; 6 Dermatology, Stanford University School of Medicine, Stanford, California, United States of America; 7 Dermatology Center of Indiana/Indiana Clinical Trials Center, Plainfield, Indiana, United States of America; 8 Dermatology, UCLA School of Medicine, Los Angeles, California, United States of America; 9 Medical Affairs, Genentech, South San Francisco, California, United States of America; 10 Biostatistics, Genentech, South San Francisco, California, United States of America; 11 Oncology, Genentech, South San Francisco, California, United States of America; 12 Dermatology, Memorial Sloan Kettering Cancer Center, New York, New York, United States of America; University Hospital Zurich, SWITZERLAND

## Abstract

**Background:**

Limited information is available regarding real-world treatment patterns and their effectiveness and safety in patients with locally advanced basal cell carcinoma, including patients not typically represented in clinical trials. The purpose of the current study was to describe how clinicians diagnose and treat locally advanced basal cell carcinoma in the United States.

**Methods:**

This prospective, multicenter, observational registry study included patients with newly diagnosed, Hedgehog pathway inhibitor–naive locally advanced basal cell carcinoma without basal cell carcinoma nevus syndrome (n = 433) treated at 75 US academic and community practices, including dermatology, Mohs surgery, and medical oncology sites. The main outcomes of this study were treatment patterns and associated effectiveness and safety for patients with locally advanced basal cell carcinoma in real-world settings.

**Results:**

Determination of locally advanced basal cell carcinoma was mainly based on lesion size (79.6% of patients), histopathology (54.3%), extent of involvement (49.0%), and location (46.2%). Within 90 days of determination of locally advanced disease, 115 patients (26.6%) received vismodegib, 251 (58.0%) received surgery/other (non-vismodegib) treatment, and 67 (15.5%) had not yet received treatment (observation). Vismodegib-treated patients had a higher prevalence of high-risk clinical features predictive for locoregional recurrence than those with non-vismodegib treatment or observation. Clinical response rate was 85.1% with vismodegib and 94.9% with non-vismodegib treatment (primarily surgery). The most common adverse events with vismodegib were ageusia/dysgeusia, muscle spasms, alopecia, and weight loss. Rates of cutaneous squamous cell cancers were comparable between vismodegib and non-vismodegib treatment.

**Conclusions:**

This prospective observational study offers insight on real-world practice, treatment selection, and outcomes for a nationally representative sample of US patients with locally advanced basal cell carcinoma. For patients with lesions that were not amenable to surgery, vismodegib treatment was associated with effectiveness and safety that was consistent with that observed in clinical trials.

## Introduction

Nonmelanoma skin cancer is the most common malignant neoplasm diagnosed in the United States [1]. Most basal cell carcinomas (BCCs) are diagnosed early, when surgery is curative; however, an estimated 1% to 10% progress to advanced (ie, locally advanced or metastatic) disease because of delays in treatment, aggressive subtypes, or recurrence/progression after surgery [2–4].

The disease course of advanced BCC is highly variable, and few treatment options are available. Mohs micrographic surgery has been used to treat locally advanced BCC and might be used in conjunction with lymph node dissection in patients with lymph node involvement [5, 6]. In patients experiencing recurrence after repeated surgical treatments or with lesions not amenable to surgery, radiation therapy may be effective [6, 7]; however, its usefulness is limited by lesion location, previous use of radiation, and presence of genetic syndromes, including BCC nevus syndrome (BCCNS; also known as Gorlin syndrome) [7, 8]. There is historically no standard chemotherapy regimen for advanced BCC, although use of platinum agents has been reported [9].

A key molecular driver of BCC is inappropriate activation of the Hedgehog signaling pathway via mutations in the tumor suppressor patched gene (*PTCH1*) and consequent activation of the smoothened gene (*SMO*) [8, 10]. The development and approval of targeted Hedgehog pathway inhibitors (HPIs) provide new options for patients with locally advanced BCC and BCCNS [11–14].

Information regarding the relative effectiveness, safety, and patterns of treatment of locally advanced BCC outside structured clinical trials is scarce. RegiSONIC (An Observational Study of Treatment Patterns and Effectiveness and Safety Outcomes in Advanced Basal Cell Carcinoma and Basal Cell Carcinoma Nevus Syndrome Patients), an advanced BCC registry conducted in the United States, is designed to prospectively evaluate how clinicians diagnose and treat advanced BCC and to characterize the effectiveness, safety, and use of systemic (HPIs and chemotherapies) and local (surgery, radiation, destructive, and topical) treatments in real-world clinical practice, with broad inclusion criteria to capture treatment patterns and outcomes for patients not typically enrolled in clinical trials. Herein, we describe baseline characteristics, diagnosis, and treatment patterns and outcomes in a subset of patients with newly diagnosed locally advanced BCC without BCCNS who were HPI-naive at baseline. This group of patients constituted the largest cohort of patients in the registry who were available for analysis.

## Materials and methods

### Study design

RegiSONIC was a multicenter, prospective, observational, three-cohort study with an enrollment of 503 patients with advanced BCC or BCCNS treated in 75 US academic and community sites, including dermatology, Mohs surgery, and oncology practices. Treatment, procedures, and clinic visit schedules were at the discretion of the treating physician, consistent with routine clinical practice.

The study was conducted in accordance with US Food and Drug Administration regulations, International Conference on Harmonization E6 Guidelines for Good Clinical Practice, and applicable local, state, and federal laws. The protocol was reviewed and approved by the following centralized or local institutional review boards: Atlantic Health System; California Pacific Medical Center IRB; Cleveland Clinic IRB; Colorado Multiple Institutional Review Board; Columbia University Institutional Review Board; Cooper Health System IRB; Emory University IRB; Icahn School for Medicine at Mount Sinai; Loma Linda University IRB; Marshall University IRB; Mayo Clinic IRB; Memorial Sloan Kettering Cancer Center; New England Institutional Review Board; Northeast Hospital Corporation; Northwestern University; Nova Southeastern University; Partners Healthcare System; Springfield Committee for Research Involving Human Subjects; Stanford University IRB; Sterling IRB; University of Arizona IRB; University of California, Irvine Institutional Review Board; University of California, San Diego; University of Miami; University of Minnesota IRB; University of Mississippi Medical Center IRB; University of North Carolina Chapel Hill; and Western Institutional Review Board. All patients provided written informed consent. This study was registered with ClinicalTrials.gov (number, NCT01604252).

### Study population

**Fig 1:**
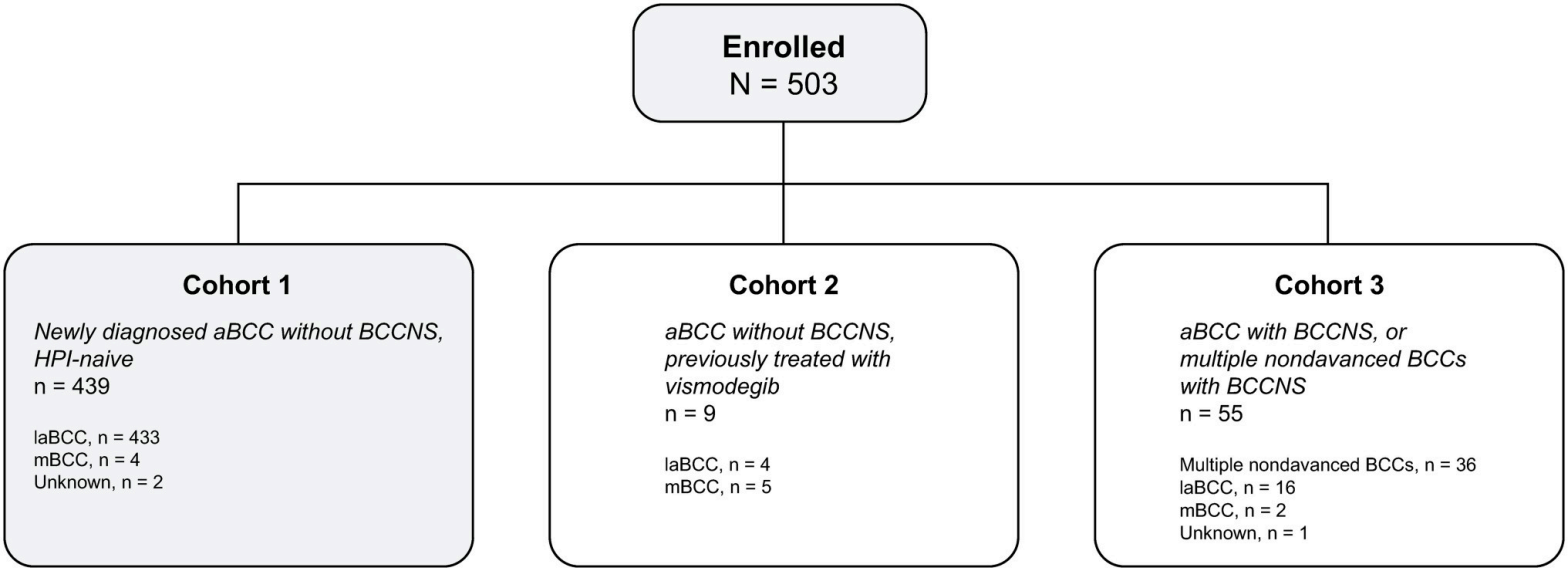
Study disposition. aBCC, advanced basal cell carcinoma; BCCNS, basal cell carcinoma nevus syndrome; HPI, Hedgehog pathway inhibitor; laBCC, locally advanced basal cell carcinoma; mBCC, metastatic basal cell carcinoma.

Eligible patients were adults (aged ≥18 years) with advanced BCC as determined by the treating clinician within 90 days before enrollment or with BCCNS at study entry. Although this study had 3 patient cohorts (Fig 1), only those enrolled in the largest cohort (Cohort 1) were included in this analysis. These patients had advanced BCC without BCCNS, had not been previously treated with an HPI, and had not participated in an advanced BCC- or HPI-related clinical trial within 90 days before enrollment.

Determination of whether BCC was advanced, selection of target lesions, and choice of treatment were at the discretion of the treating clinician. Target lesions were recorded and measured at baseline and assessed at each follow-up visit. Response was assessed by the treating physician according to their method of choice.

### Data collection

Data that included medical conditions/procedures, changes in concomitant medications, disease status/tumor response, Eastern Cooperative Oncology Group (ECOG) performance status, treatment status, and adverse events (AEs) were collected quarterly. All patients were required to complete annual study visits over a 3-year follow-up period. Patients were followed until completion of 3 years of follow-up, death, withdrawal of consent, loss to follow-up, or study termination, whichever occurred first, and analyses included data up to the last data collection timepoint for all enrolled patients.

AE collection was limited to serious AEs (fatal, life-threatening, requiring hospitalization, resulting in significant disability, or otherwise considered medically significant by the clinician), AEs leading to treatment discontinuation, or protocol-defined AEs of interest (muscle spasms, arthralgia, alopecia, dysgeusia/ageusia, unintentional weight loss, fatigue grade ≥2, gastrointestinal events grade ≥2, cardiovascular events, amenorrhea, and squamous cell carcinoma [SCC]).

### Statistical analysis

The objectives of this study were to characterize factors associated with advanced BCC diagnosis, initial treatment choices, treatment effectiveness and safety, and changes in treatments in patients with locally advanced BCC without BCCNS (Cohort 1) in real-world clinical practice. Given the potential for multiple and varied treatments during follow-up, 3 treatment groups were defined based on treatment received within 90 days after locally advanced BCC diagnosis, regardless of any subsequent treatment: 1) vismodegib (received vismodegib alone or in combination with other treatments within 90 days of diagnosis), 2) non-vismodegib treatment (received treatments other than vismodegib [eg, surgery, radiation, photodynamic therapy, topical treatment, other systemic therapy] within 90 days of diagnosis), and 3) observation (did not receive treatment within 90 days of diagnosis, but may have received treatment thereafter).

Descriptive analyses were conducted to summarize patient and clinical disease characteristics at baseline, characterize treatment patterns, and assess effectiveness and safety outcomes of treatment using statistical software (SAS, version 9.2 or later; SAS Institute Inc., Cary, NC, USA).

## Results

### Patients

Between June 2012 and August 2015, 503 eligible patients were enrolled at 75 US academic and community practices, including dermatology, Mohs surgery, and medical oncology sites; 439 patients were enrolled in Cohort 1, 9 patients in Cohort 2, and 55 patients in Cohort 3 (Fig 1). Overall, 37 eligible patients declined to participate. To reduce the risk of selection bias, investigators were encouraged to invite all eligible patients to participate in the study. Data from 433 HPI-naive patients with newly diagnosed, locally advanced BCC without BCCNS enrolled in Cohort 1 are presented; 4 patients with metastatic disease and 2 patients whose disease could not be adequately subclassified were excluded from this analysis.

At data cutoff (August 31, 2017), median follow-up duration was 23.6 months (range 0.03–45.04). All 433 patients had discontinued the study, including 69 patients (15.9%) who completed the study with 3 years of follow-up. The most common reasons for study discontinuation were sponsor decision to terminate the study (150 patients; 34.6%), loss to follow-up (83 patients; 19.2%), and patient decision to withdraw (55 patients; 12.7%).

### Patient demographics

Most patients were aged ≥65 years (56.8%; median, 67 years), and most (62.6%) were men (Table 1). Almost all patients were white (99.5%) and had sun-sensitive skin phototypes (Fitzpatrick skin type I–III; 90.4%).

**Table 1:** Baseline demographics in patients with locally advanced basal cell carcinoma in Cohort 1.

Characteristic	laBCC patients in Cohort 1			
	All	Vismodegib	Non-vismodegib treatment	Observation
	n = 433	n = 115	n = 251	n = 67
**Age, years, median (range)**	67 (23–99)	66 (34–99)	66 (23–93)	69 (35–96)
**Sex, n (%)**	n = 433	n = 115	n = 251	n = 67
Male	271 (62.6)	78 (67.8)	145 (57.8)	48 (71.6)
Female	162 (37.4)	37 (32.2)	106 (42.2)	19 (28.4)
**Race, n (%)** ^a^	n = 433	n = 115	n = 251	n = 67
White	431 (99.5)	114 (99.1)	250 (99.6)	67 (100)
American Indian or Alaskan native	2 (0.5)	0	1 (0.4)	1 (1.5)
Other^b^	2 (0.5)	1 (0.9)	1 (0.4)	0
**Employment status, n (%)**	n = 431	n = 115	n = 251	n = 65
Employed	134 (31.1)	36 (31.3)	80 (31.9)	18 (27.7)
Unemployed	68 (15.8)	19 (16.5)	38 (15.1)	11 (16.9)
Retired	229 (53.1)	60 (52.2)	133 (53.0)	36 (55.4)
**Highest level of education, n (%)**	n = 433	n = 115	n = 251	n = 67
High school graduate or less	148 (34.2)	46 (40.0)	80 (31.9)	22 (32.8)
Some college or associates degree	98 (22.6)	26 (22.6)	58 (23.1)	14 (20.9)
College graduate or above	122 (28.2)	24 (20.9)	81 (32.3)	17 (25.4)
Unknown	65 (15.0)	19 (16.5)	32 (12.7)	14 (20.9)
**Tobacco use, n (%)**	n = 433	n = 115	n = 251	n = 67
Current	60 (13.9)	17(14.8)	37 (14.7)	6 (9.0)
Former	139 (32.1)	46 (40.0)	68 (27.1)	25 (37.3)
Never	227 (52.4)	49 (42.6)	144 (57.4)	34 (50.7)
Unknown	7 (1.6)	3 (2.6)	2 (0.8)	2 (3.0)
**Alcohol use, n (%)**	n = 433	n = 115	n = 251	n = 67
Current	219 (50.6)	50 (43.5)	134 (53.4)	35 (52.2)
Former	63 (14.5)	16 (13.9)	38 (15.1)	9 (13.4)
Never	133 (30.7)	40 (34.8)	73 (29.1)	20 (29.9)
Unknown	18 (4.2)	9 (7.8)	6 (2.4)	3 (4.5)
**Sunscreen use, n (%)**	n = 433	n = 115	n = 251	n = 67
Regularly	133 (30.7)	28 (24.3)	84 (33.5)	21 (31.3)
Occasionally	151 (34.9)	35 (30.4)	90 (35.9)	26 (38.8)
Never	120 (27.7)	39 (33.9)	67 (26.7)	14 (20.9)
Unknown	29 (6.7)	13 (11.3)	10 (4.0)	6 (9.0)
**Fitzpatrick skin type, n (%)**	n = 427	n = 113	n = 250	n = 64
I: Highly sun sensitive	86 (20.1)	28 (24.8)	46 (18.4)	12 (18.8)
II: Very sun sensitive	184 (43.1)	47 (41.6)	106 (42.4)	31 (48.4)
III: Sun sensitive	116 (27.2)	32 (28.3)	68 (27.2)	16 (25.0)
IV: Minimally sun sensitive	32 (7.5)	4 (3.5)	25 (10.0)	3 (4.7)
V: Sun insensitive, rarely burns	9 (2.1)	2 (1.8)	5 (2.0)	2 (3.1)
VI: Sun insensitive, never burns	0	0	0	0

Abbreviation: laBCC, locally advanced basal cell carcinoma.

^a^ Patients may have been counted more than once.

^b^ Two patients of Hispanic ethnicity had race recorded as “other.”

Vismodegib = received vismodegib within 90 days of diagnosis; non-vismodegib treatment = received interventions other than vismodegib within 90 days of diagnosis; observation = received no intervention within 90 days of diagnosis.

Compared with those who received non-vismodegib treatment, vismodegib-treated patients and those who underwent observation were more likely to be men (57.8% vs 67.8% and 71.6%). Vismodegib-treated patients were more likely than those in the non-vismodegib treatment and observation groups to be current or former smokers (54.8% vs 41.8% and 46.3%) and to be less educated (high school graduate or less; 40.0% vs 31.9% and 32.8%) and were less likely to be regular sunscreen users (24.3% vs 33.5% and 31.3%). Patients who received non-vismodegib treatment were more likely to have good baseline performance status than vismodegib-treated patients and those who underwent observation (ECOG performance status 0: 59.3% vs 42.2% or 43.1%). Evaluation of medical history included all dermatologic and nondermatologic medical conditions as well as non–BCC-related surgeries and procedures in patient medical records. These were extracted and coded using Medical Dictionary for Regulatory Activities version 20.0. There were no identifiable factors in medical history associated with treatment choice. Performance status (ECOG) was extracted for the most recent assessment before study enrollment and change in performance status was assessed at each follow-up visit.

### Diagnosis and disease characteristics

For most patients (369; 85%), determination of locally advanced BCC was based on multiple factors, most commonly lesion size (79.6% of patients), histopathology (54.3%), extent of involvement (49.0%), location (46.2%), recurrence (26.7%), and likelihood of curative resection (25.3%) (Table 2). When determination of locally advanced BCC was based on lesion size, lesions measured ≥20 mm in 63.0% of patients; when based on recurrence, 40.9% of patients experienced ≥2 recurrences; when based on location, the lesion site was the face in 69.8% of patients. Evaluation of variables that may reflect lack of access to care or undertreatment (ie, health insurance coverage, psychiatric disorders/dementia, and BCC treatment history) did not provide any insight into factors contributing to the development of advanced BCC (S1 Table).

**Table 2:** Determination of locally advanced basal cell carcinoma in patients in Cohort 1.

Criteria used to make determination of laBCC, n (%)^a^	laBCC patients in Cohort 1			
	All	Vismodegib	Non-vismodegib treatment	Observation
	n = 433	n = 115	n = 251	n = 67
**Lesion size**	343 (79.6)	83 (72.8)	217 (86.5)	43 (65.2)
<10 mm^b^	39 (11.4)	9 (10.8)	25 (11.5)	5 (11.6)
10–19 mm^b^	88 (25.7)	13 (15.7)	64 (29.5)	11 (25.6)
≥20 mm^b^	216 (63.0)	61 (73.5)	128 (59.0)	27 (62.8)
**Histopathology** ^c^	234 (54.3)	57 (50.0)	149 (59.4)	28 (42.4)
**Extent of involvement** ^d^	211 (49.0)	60 (52.6)	122 (48.6)	29 (43.9)
**Location** ^e^	199 (46.2)	76 (66.7)	85 (33.9)	38 (57.6)
**Recurrence**	115 (26.7)	43 (37.7)	41 (16.3)	31 (47.0)
<2^f^	68 (59.1)	28 (65.1)	25 (61.0)	15 (48.4)
≥2^f^	47 (40.9)	15 (34.9)	16 (39.0)	16 (51.6)
**Curative resection unlikely**	109 (25.3)	56 (49.1)	34 (13.5)	19 (28.8)
**Medical contraindication to surgery (eg, comorbidity, concomitant medication)**	57 (13.2)	29 (25.4)	17 (6.8)	11 (16.7)
**Medical contraindication to radiation (eg, age)**	73 (16.9)	35 (30.7)	26 (10.4)	12 (18.2)
**Other**	40 (9.3)	9 (7.9)	19 (7.6)	12 (18.2)

Abbreviation: laBCC, locally advanced basal cell carcinoma.

^a^ Patients may be included in more than 1 category; percentages may therefore add up to >100%.

^b^ Denominator for percentages is the number of patients in whom lesion size was used to determine laBCC.

^c^ No criteria for determination of laBCC based on histopathology were dictated by the protocol.

^d^ Locally invasive (eg, tumor extending into underlying tissue cartilage, bone, nerve).

^e^ Surgery or radiation therapy would result in disfigurement or loss of function.

^f^ Denominator for percentages is the number of patients in whom recurrence was used to determine laBCC.

Vismodegib = received vismodegib within 90 days of diagnosis; non-vismodegib treatment = received treatments other than vismodegib within 90 days of diagnosis; observation = received no intervention within 90 days of diagnosis.

At baseline, 23.3% of patients had multiple clinically visible advanced lesions (Table 3), with a median of 3 lesions per patient. Target lesions were located on the head/neck in 63.6% of patients and were most commonly nodular (64.6%) or morpheaform/infiltrative (27.4%).

**Table 3:** Baseline disease characteristics in patients with locally advanced basal cell carcinoma in Cohort 1.

Characteristic	laBCC patients in Cohort 1			
	All	Vismodegib	Non-vismodegib treatment	Observation
	n = 433	n = 115	n = 251	n = 67
**ECOG performance status prior to treatment, n (%)**	n = 350	n = 83	n = 209	n = 58
0	184 (52.6)	35 (42.2%)	124 (59.3%)	25 (43.1%)
1	36 (10.3)	3 (15.7%)	16 (7.7%)	7 (12.1%)
2	22 (6.3)	7 (8.4%)	13 (6.2%)	2 (3.4%)
3	9 (2.6)	0	4 (1.9%)	5 (8.6%)
4	6 (1.7)	4 (4.8%)	1 (0.5%)	1 (1.7%)
Unknown	93 (26.6)	24 (28.9%)	51 (24.4%)	18 (31.0%)
**History of BCC, n (%)**	n = 433	n = 115	n = 251	n = 67
Yes	281 (64.9)	80 (69.6%)	149 (59.4%)	52 (77.6%)
No	152 (35.1)	35 (30.4%)	102 (40.6%)	15 (22.4%)
**Time from determination of laBCC to enrollment, months, median (range)** ^a^	0.4 (–0.3 to 75.7)	0.099 (–0.30 to 3.68)	0.460 (0.00 to 3.09)	0.559 (–0.20 to 75.66)
**Clinically visible advanced lesions, n (%)**	n = 433	n = 115	n = 251	n = 67
Single	332 (76.7)	77 (67.0%)	207 (82.5%)	48 (71.6%)
Multiple	101 (23.3)	38 (33.0%)	44 (17.5%)	19 (28.4%)
Number of lesions, median (range)^b^	3 (2–50)	4 (2–50)	3 (2–10)	3 (2–10)
Number of lesions, mean (SD)^b^	4.7 (5.5)	6.2 (8.4)	3.9 (2.3)	3.8 (2.5)
**Target lesion location, n (%)**	n = 429	n = 115	n = 249	n = 66
Nose	85 (19.8)	20 (17.5)	53 (21.3)	12 (18.2)
Forehead	50 (11.7)	14 (12.3)	31 (12.4)	5 (7.6)
Ear	38 (8.9)	8 (7.0)	27 (10.8)	3 (4.5)
Cheek	35 (8.2)	9 (7.9)	21 (8.4)	5 (7.6)
Back	34 (7.9)	16 (14.0)	14 (5.6)	4 (6.1)
Scalp	26 (6.1)	7 (6.1)	15 (6.0)	4 (6.1)
Eye	21 (4.9)	7 (6.1)	9 (3.6)	5 (7.6)
Chest	19 (4.4)	5 (4.4)	11 (4.4)	3 (4.5)
Leg	19 (4.4)	6 (5.3)	8 (3.2)	5 (7.6)
Shoulder	16 (3.7)	2 (1.8)	10 (4.0)	4 (6.1)
Arm	14 (3.3)	2 (1.8)	10 (4.0)	2 (3.0)
Neck	12 (2.8)	3 (2.6)	7 (2.8)	2 (3.0)
Chin	6 (1.4)	0	4 (1.6)	2 (3.0)
Abdomen	3 (0.7)	0	2 (0.8)	1 (1.5)
Foot	1 (0.2)	1 (0.9)	0	0
Hand	0	0	0	0
Other	50 (11.7)	14 (12.3)	27 (10.8)	9 (13.6)
**Clinical/histopathologic subtype, n (%)**	n = 390	n = 104	n = 228	n = 58
Nodular	252 (64.6)	66 (63.5)	144 (63.2)	42 (72.4)
Morpheaform/infiltrative	107 (27.4)	34 (32.7)	62 (27.2)	11 (19.0)
Superficial	59 (15.1)	12 (11.5)	38 (16.7)	9 (15.5)
Micronodular	13 (3.3)	5 (4.8)	7 (3.1)	1 (1.7)
Basosquamous	10 (2.6)	5 (4.8)	4 (1.8)	1 (1.7)
Other	40 (10.3)	8 (7.7)	23 (10.1)	9 (15.5)
**Target lesion**	n = 415	n = 107	n = 245	n = 63
Lesion size, mm, mean (SD)	30.4 (36.9)	38.2 (37.6)	28.7 (39.3)	23.3 (21.6)

Abbreviations: BCC, basal cell carcinoma; ECOG, Eastern Cooperative Oncology Group; laBCC, locally advanced basal cell carcinoma.

^a^ In 3 patients, the date of laBCC determination preceded the date of informed consent; 1 of these patients was subsequently determined to be ineligible.

^b^ Among patients with multiple lesions.

Vismodegib = received vismodegib within 90 days of diagnosis; non-vismodegib treatment = received interventions other than vismodegib within 90 days of diagnosis; observation = received no intervention within 90 days of diagnosis.

Disease characteristics differed across treatment groups, with a higher prevalence of clinical features predictive for increased risk of locoregional recurrence in vismodegib-treated patients and patients in the observation group compared with those who received non-vismodegib treatment, including lesion size ≥20 mm (73.5% and 62.8% vs 59.0%), curative resection unlikely (49.1% and 28.8% vs 13.5%), and recurrent disease (37.7% and 47.0% vs 16.3%). However, distribution of lesions across anatomic sites, particularly high-risk areas of the head/neck, were comparable across groups. More patients in the vismodegib group had medical contraindications to surgery or radiotherapy or were expected to have significant cosmetic/functional deficits with local treatment.

### Treatment

Of the 433 patients, 115 (26.6%) were included in the vismodegib group, 251 (58.0%) in the non-vismodegib treatment group, and 67 (15.5%) in the observation group (no treatment within 90 days of enrollment). Median follow-up duration was 25.4 months (range 0.16–39.56), 22.8 months (range 0.03–45.04), and 21.2 months (range 0.03–44.25), respectively.

**Fig 2:**
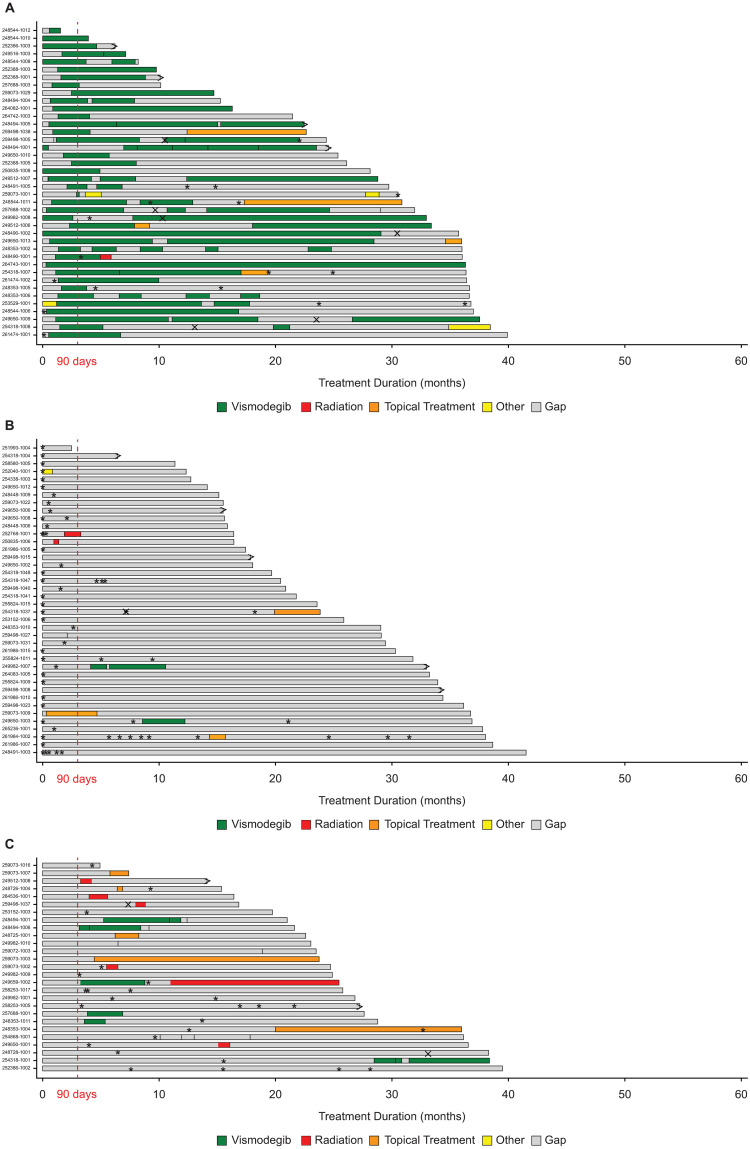
Basal cell carcinoma. Representative treatment patterns among randomly selected patients treated within the first 90 days with vismodegib (n = 40, **A**), non-vismodegib treatment (n = 40, **B**), and observation (n = 28, **C**). Vismodegib = received vismodegib within 90 days of diagnosis; non-vismodegib treatment = received interventions other than vismodegib within 90 days of diagnosis; observation = received no intervention within 90 days of diagnosis. Bar lengths represent duration from date of locally advanced basal cell carcinoma diagnosis to study termination, death, or data cutoff; > denotes death; X denotes initial progression; * denotes surgery.

Patients in the vismodegib group generally remained on vismodegib and, in some cases, restarted vismodegib after intervals of a few to several months off treatment (Fig 2A). Most patients receiving vismodegib (108 patients; 93.9%) had ≥1 interruption or change in treatment, most commonly because of AEs (36 patients), maximum benefit achieved (21 patients), patient decision (19 patients), or treatment holiday (14 patients). The median duration of vismodegib treatment was 6.31 months (range 0.03–31.97).

Of 251 patients in the non-vismodegib treatment group, most (198 patients [78.9%]) received only surgery (eg, Mohs surgery, cryosurgery, excision); 53 patients (21.1%) received other non-vismodegib treatments (eg, topical therapy, radiation, other systemic therapy) within 90 days after locally advanced BCC diagnosis. After 90 days, few patients received other treatments, although patients might have undergone multiple surgeries (Fig 2B). Most patients (287; 98.4%) had ≥1 interruption or change in treatment, most commonly because maximum benefit was achieved (196 patients).

Of 67 patients in the observation group, several patients received treatment after 90 days (Fig 2C), most commonly surgery (19 patients; 28.4%), vismodegib (6 patients; 9.0%), radiation (5 patients; 7.5%), or topical treatment (3 patients; 4.5%).

### Response assessment and effectiveness

Disease assessment at baseline and during treatment consisted primarily of clinical evaluation (93.5%, 90.3%, and 88.9% of patients in the vismodegib, non-vismodegib treatment, and observation groups, respectively), and less frequently, histopathologic evaluation (6.5%, 16.6%, and 13.9%, respectively) or diagnostic imaging (8.6%, 1.1%, and 0, respectively) (S1 Fig). Clinical evaluation was based on visual parameters, such as lesion size (S2 Fig); complete response assessment was most commonly based on lack of clinically visible evidence of BCC (>60%) (S3 Fig). Patients in the non-vismodegib treatment group, most of whom underwent surgery, were more likely to have only one posttreatment disease assessment than vismodegib-treated patients (26.6% vs 5.7%).

Among 101 evaluable vismodegib-treated patients, the clinical response rate was 85.1%, with complete response in 63.4%. Median duration of response was 17.5 months, and disease recurrence was reported in 23.8% of patients. Among 198 evaluable patients in the non-vismodegib treatment group, most of whom underwent surgery, clinical and complete response rates were 94.9% and 91.4%, respectively. Disease recurrence was reported in 2.0% of patients.

### Safety

Because AE collection was limited to serious AEs or AEs of interest unless an AE caused treatment discontinuation, nonserious AEs were less likely to be collected in the non-vismodegib treatment group, as most patients in that group underwent surgery and frequently had only one posttreatment assessment following an assessment of complete response.

In the vismodegib group, AEs were reported in 104 patients (90.4%). The most common all-grade AEs (≥20% of patients) were ageusia/dysgeusia (71 patients; 61.7%), muscle spasms (66 patients; 57.4%), alopecia (58 patients; 50.4%), and weight loss (29 patients; 25.2%). Serious AEs occurred in 29 patients (25.2%), and 24 (20.9%) patients discontinued treatment because of AEs. Cutaneous SCCs were reported in 14 patients (12.2%), with an exposure-adjusted incidence rate of 0.06 cases per patient-year.

In the non-vismodegib treatment group, AEs were reported in 65 patients (25.9%). Serious AEs occurred in 35 patients (13.9%). Cutaneous SCCs were reported in 31 patients (12.4%), with an exposure-adjusted incidence rate of 0.07 cases per patient-year.

## Discussion

In this prospective, observational study, we identified distinct treatment patterns of real-world patients with locally advanced BCC who were given different treatments based on initial clinical presentation. The availability of vismodegib highlighted the need to better define locally advanced BCC. Historically, “advanced” BCCs (although rarely described as such) were those necessitating complex treatment beyond usual standard of care (ie, surgery) or recurring repeatedly despite adequate local treatment [15, 16]. Clinical trial eligibility criteria have defined locally advanced BCC based on lesion size, extent of local invasiveness, tumor location, expected morbidity or mortality from local treatment, low likelihood of curative resection, contraindication to local treatment, or number of recurrences [16]. Many of these criteria are subjective, and little is known about how physicians make the determination of locally advanced BCC in clinical practice. Data from this prospective, observational study indicate that locally advanced BCC determination was typically made based on multiple factors. Lesion size was the most common criterion for determination of locally advanced BCC (almost 80% of patients), frequently in conjunction with histopathology, extent of involvement, or tumor location (around 50% of patients each). Consensus criteria for defining locally advanced BCC as inappropriate for surgery or radiotherapy were recently described and might assist in standardization of locally advanced BCC determination in clinical practice [17].

Delays in diagnosis and treatment can contribute to development of advanced BCC [2–4]. Barriers to accessing care may include lack of health insurance and history of mental illness. In the current study, insurance coverage was high across the study population and was consistent with coverage rates in the general US population [18]. Additionally, prevalences of psychiatric disorders and dementia in the study population were similar across treatment cohorts and similar to or lower than prevalences in the general population [19–21]. Although data on prior treatment history for the target BCC were available for only a subset of patients, rates of nontreatment were generally low across cohorts. Thus, evaluation of variables that may reflect lack of access to care or undertreatment did not provide any insight into factors contributing to the development of advanced BCC in this patient population.

Results also indicate that initial treatment choices for locally advanced BCC are dictated primarily by tumor size, number of tumors, extent of involvement, histopathology, and anatomic location of BCC lesions, which is consistent with anecdotal observations [22]. Vismodegib was the only HPI available throughout study accrual (June 2012 through August 2015), with sonidegib receiving approval in July 2015. Patients treated with vismodegib were clearly different from patients treated with other modalities (predominantly surgery; 78.9%), with a higher prevalence of poor-risk features. In particular, vismodegib was chosen more frequently for patients with multiple or larger lesions (≥20 mm), consistent with considerations of disfigurement and potentially higher risk of recurrence after surgery for patients with larger lesions or lesions located in the periocular, paranasal, or scalp regions [23].

Given the different clinical presentations of vismodegib-treated versus non–vismodegib-treated patients, outcomes should not be compared between treatment groups. However, outcomes for each treatment appear consistent with those reported in the literature with clinical trial populations that typically exclude patients with comorbidities, the elderly, or patients with poor performance status. The clinical response rate in vismodegib-treated patients was consistent with investigator-assessed response rates in the pivotal ERIVANCE-BCC study (60.3%) and STEVIE safety study (68.5%), although the complete response rate was higher than that observed in clinical studies (31.7% in ERIVANCE-BCC and 33.4% in STEVIE), which likely reflects more rigorous methods of response assessment in clinical trials relative to the real-world setting [11, 12]. In these clinical studies, determination of response used formal structured assessments based on physical examination and radiology per Response Evaluation Criteria in Solid Tumors (RECIST), with histopathological confirmation of complete response in the ERIVANCE-BCC study. In contrast, our data suggest that response is primarily determined on the basis of clinical evaluation with infrequent use of histopathology or imaging in the real-world setting. Median duration of response was comparable to those observed in ERIVANCE-BCC (26.2 months) and STEVIE (23.0 months), given differences in follow-up time across studies [11, 12]. Outcomes observed in patients who received non-vismodegib treatment (most commonly, surgery) should be interpreted with caution, as many patients had only one follow-up visit. Nevertheless, the observed recurrence rate for these patients is within the range reported in randomized clinical trials or observational studies of surgical outcomes [3, 24–28].

In general, the safety profile of vismodegib was manageable and similar to that reported in clinical trials [29, 30]. The most common AEs were ageusia/dysgeusia, muscle spasms, alopecia, and weight loss. Concerns have been raised regarding an increased risk of cutaneous SCC with vismodegib, with conflicting findings from retrospective cohort analyses [31, 32]. In this observational study, cutaneous SCC was collected prospectively as an AE of interest and vismodegib treatment did not appear to be associated with an increased risk of SCC. To our knowledge, this report represents the first prospective evaluation of the incidence of cutaneous SCCs in vismodegib-treated patients, including a concurrent group of patients managed primarily by surgical excision serving as a control. Whereas Fitzpatrick skin types were comparable across groups, regular sunscreen use was slightly more common in the non-vismodegib treatment group.

Overall, treatment patterns showed that vismodegib-treated patients often received vismodegib over multiple intervals during follow-up, although a few patients initially treated with vismodegib subsequently underwent surgery. Several reports suggest that neoadjuvant vismodegib might shrink tumors, thereby allowing less extensive surgery with lower risk for morbidity [33–35]. These data suggest the need to further explore the potential utility of HPIs in the neoadjuvant setting.

Strengths of this study are the inclusion of a nationally representative sample of patients from a range of US academic and community practices, including dermatology, Mohs surgery, and medical oncology sites. As a result of broad inclusion criteria, the registry included patients who would typically be excluded from clinical trials. Moreover, patient participation was extensive, with only 37 eligible patients declining to participate over the 3-year accrual period.

Limitations of this study include those commonly associated with observational studies in the real-world setting, such as nonrandomized treatment assignment, lack of standardized assessment, and variation in treatment practices across centers. Moreover, patients were seen by a variety of different providers, including dermatologists, Mohs surgeons, and oncologists. Such diversity contributes to challenges in describing treatment patterns for patients with locally advanced BCC in the real-world setting. The relatively high rate of patient decision to withdraw (12.7%) was comparable to rates observed in clinical trials of vismodegib and other published long-term BCC studies [29, 30, 36–38]. Loss of patients to follow-up is high in BCC studies and probably reflects the older age of BCC populations and the fact that BCC is typically not immediately life-threatening [36–38]. In addition, follow-up of patients was limited because of the sponsor’s decision to close the study 2 years earlier than originally planned.

## Conclusions

The RegiSONIC study offers useful insight on real-world practice and treatment selection in patients with locally advanced BCC and highlights differences in disease characteristics that may drive choice of treatment. Effectiveness and safety of vismodegib in the real-world setting was consistent with that observed in clinical trials.

## Supporting information

S1 Fig Methodology used for disease assessment. Abbreviations: CT, computed tomography; MRI, magnetic resonance imaging. Vismodegib = received vismodegib within 90 days of diagnosis; non-vismodegib treatment = received interventions other than vismodegib within 90 days of diagnosis; Observation = received no intervention within 90 days of diagnosis.
(PDF)

S2 Fig Parameters used to determine response assessment. Abbreviations: BCC, basal cell carcinoma; laBCC, locally advanced basal cell carcinoma; mBCC, metastatic basal cell carcinoma. Vismodegib = received vismodegib within 90 days of diagnosis; non-vismodegib treatment = received interventions other than vismodegib within 90 days of diagnosis; Observation = received no intervention within 90 days of diagnosis.
(PDF)

S3 Fig Criteria for assessment of complete response. Abbreviations: BCC, basal cell carcinoma. Vismodegib = received vismodegib within 90 days of diagnosis; non-vismodegib treatment = received interventions other than vismodegib within 90 days of diagnosis; Observation = received no intervention within 90 days of diagnosis.
(PDF)

S1 Table Health insurance coverage, history of psychiatric disorders/dementia, and BCC treatment history in patients with locally advanced basal cell carcinoma in Cohort 1. Abbreviations: BCC, basal cell carcinoma; laBCC, locally advanced basal cell carcinoma. ^a^Specific diagnoses occurring in 2 or more patients are reported. Vismodegib = received vismodegib within 90 days of diagnosis; non-vismodegib treatment = received interventions other than vismodegib within 90 days of diagnosis; observation = received no intervention within 90 days of diagnosis.
(PDF)

S1 Data (PDF)

## References

[1] Rogers HW, Weinstock MA, Harris AR, et al. Incidence estimate of nonmelanoma skin cancer in the United States, 2006. Arch Dermatol. 2010;146(3):283–7. doi: 10.1001/archdermatol.2010.19 20231499

[2] Alam M, Goldberg LH, Silapunt S, et al. Delayed treatment and continued growth of nonmelanoma skin cancer. J Am Acad Dermatol. 2011;64(5):839–48. doi: 10.1016/j.jaad.2010.06.028 . 21055843

[3] Chren M-M, Linos E, Torres JS, Stuart SE, Parvataneni R, Boscardin WJ. Tumor recurrence 5 years after treatment of cutaneous basal cell carcinoma and squamous cell carcinoma. J Invest Dermatol. 2013;133(5):1188–96. doi: 10.1038/jid.2012.403 23190903 PMC3711403

[4] Mohan SV, Chang AL. Advanced basal cell carcinoma: epidemiology and therapeutic innovations. Curr Dermatol Rep. 2014;3:40–5. doi: 10.1007/s13671-014-0069-y . 24587976 PMC3931971

[5] Goppner D, Leverkus M. Basal cell carcinoma: from the molecular understanding of the pathogenesis to targeted therapy of progressive disease. J Skin Cancer. 2011;2011:650258. doi: 10.1155/2011/650258 . 21253551 PMC3021865

[6] Weinstock MA, Still JM. Assessing current treatment options for patients with severe/advanced basal cell carcinoma. Semin Cutan Med Surg. 2011;30(4 Suppl):S10–S3. doi: 10.1016/j.sder.2011.11.004 22177101

[7] Fecher LA. Systemic therapy for inoperable and metastatic basal cell cancer. Curr Treat Options Oncol. 2013;14:237–48. doi: 10.1007/s11864-013-0233-9 . 23558911

[8] Lo Muzio L. Nevoid basal cell carcinoma syndrome (Gorlin syndrome). Orphanet J Rare Dis. 2008;3:32. doi: 10.1186/1750-1172-3-32 . 19032739 PMC2607262

[9] Ganti AK, Kessinger A. Systemic therapy for disseminated basal cell carcinoma: an uncommon manifestation of a common cancer. Cancer Treat Rev. 2011;37(6):440–3. doi: 10.1016/j.ctrv.2010.12.002 . 21216106

[10] Gorlin RJ, Goltz RW. Multiple nevoid basal-cell epithelioma, jaw cysts and bifid rib. A syndrome. N Engl J Med. 1960;262:908–12. Epub 1960/05/05. doi: 10.1056/NEJM196005052621803 . 13851319

[11] Basset-Seguin N, Hauschild A, Kunstfeld R, et al. Vismodegib in patients with advanced basal cell carcinoma: primary analysis of STEVIE, an international, open-label trial. Eur J Cancer. 2017;86:334–48. doi: 10.1016/j.ejca.2017.08.022 . 29073584

[12] Sekulic A, Migden MR, Basset-Seguin N, et al. Long-term safety and efficacy of vismodegib in patients with advanced basal cell carcinoma: final update of the pivotal ERIVANCE BCC study. BMC Cancer. 2017;17(1):332. doi: 10.1186/s12885-017-3286-5 . 28511673 PMC5433030

[13] Sekulic A, Migden MR, Oro AE, et al. Efficacy and safety of vismodegib in advanced basal-cell carcinoma. N Engl J Med. 2012;366(23):2171–9. doi: 10.1056/NEJMoa1113713 . 22670903 PMC5278761

[14] Migden MR, Guminski A, Gutzmer R, et al. Treatment with two different doses of sonidegib in patients with locally advanced or metastatic basal cell carcinoma (BOLT): a multicentre, randomised, double-blind phase 2 trial. Lancet Oncol. 2015;16(6):716–28. doi: 10.1016/S1470-2045(15)70100-2 . 25981810

[15] Sekulic A, Mangold AR, Northfelt DW, LoRusso PM. Advanced basal cell carcinoma of the skin: targeting the hedgehog pathway. Curr Opin Oncol. 2013;25(3):218–23. doi: 10.1097/CCO.0b013e32835ff438 . 23493193

[16] Maly TJ, Sligh JE. Defining locally advanced Basal cell carcinoma. J Drugs Dermatol. 2014;13(5):528–9. S1545961614P0528X. . 24809873

[17] Peris K, Licitra L, Ascierto PA, et al. Identifying locally advanced basal cell carcinoma eligible for treatment with vismodegib: an expert panel consensus. Future Oncol. 2015;11(4):703–12. doi: 10.2217/fon.14.281 . 25686123

[18] Berchick ER, Hood E, Barnett JC. Health insurance coverage in the United States: 2017 Washington, DC, USA: United States Census Bureau; 2018 [cited 1 Apr 2021]. https://www.census.gov/content/dam/Census/library/publications/2018/demo/p60-264.pdf

[19] National Institute of Mental Health. Statistics: Mental illness. Bethesda, MD, USA: National Institutes of Health; 2021 [updated Jan 2021; cited 1 Apr 2021]. https://www.nimh.nih.gov/health/statistics/mental-illness.shtml

[20] National Institute of Mental Health. Statistics: any anxiety disorder. Bethesda, MD, USA: National Institutes of Health; 2021 [updated Jan 2021; cited 1 Apr 2021]. https://www.nimh.nih.gov/health/statistics/any-anxiety-disorder.shtml

[21] Alzheimer’s Association. 2021 Alzheimer’s disease facts and figures. Alzheimers Dement. 2021;17(3):327–406. Epub 2021/03/24. doi: 10.1002/alz.12328 . 33756057

[22] Lewin JM, Carucci JA. Advances in the management of basal cell carcinoma. F1000Prime Rep. 2015;7:53. doi: 10.12703/P7-53 . 26097726 PMC4447055

[23] Marzuka AG, Book SE. Basal cell carcinoma: pathogenesis, epidemiology, clinical features, diagnosis, histopathology, and management. Yale J Biol Med. 2015;88(2):167–79. . 26029015 PMC4445438

[24] Chren M-M, Torres JS, Stuart SE, Bertenthal D, Labrador RJ, Boscardin WJ. Recurrence after treatment of nonmelanoma skin cancer: a prospective cohort study. Arch Dermatol. 2011;147(5):540–6. doi: 10.1001/archdermatol.2011.109 21576572 PMC3145327

[25] Leibovitch I, Huilgol SC, Selva D, Richards S, Paver R. Basal cell carcinoma treated with Mohs surgery in Australia II. Outcome at 5-year follow-up. J Am Acad Dermatol. 2005;53(3):452–7. doi: 10.1016/j.jaad.2005.04.087 . 16112352

[26] Leibovitch I, Huilgol SC, Selva D, Richards S, Paver R. Basal cell carcinoma treated with Mohs surgery in Australia I. Experience over 10 years. J Am Acad Dermatol. 2005;53(3):445–51. doi: 10.1016/j.jaad.2005.04.083 . 16112351

[27] Mosterd K, Krekels GA, Nieman FH, et al. Surgical excision versus Mohs’ micrographic surgery for primary and recurrent basal-cell carcinoma of the face: a prospective randomised controlled trial with 5-years’ follow-up. Lancet Oncol. 2008;9(12):1149–56. doi: 10.1016/S1470-2045(08)70260-2 . 19010733

[28] Smeets NW, Krekels GA, Ostertag JU, et al. Surgical excision vs Mohs’ micrographic surgery for basal-cell carcinoma of the face: randomised controlled trial. Lancet. 2004;364(9447):1766–72. doi: 10.1016/S0140-6736(04)17399-6 . 15541449

[29] Sekulic A, Migden MR, Lewis K, et al. Pivotal ERIVANCE BCC study: 12-month update of efficacy and safety of vismodegib in advanced basal cell carcinoma. J Am Acad Dermatol. 2015;72:1021–6. doi: 10.1016/j.jaad.2015.03.021 . 25981002

[30] Basset-Seguin N, Hauschild A, Grob JJ, et al. Vismodegib in patients with advanced basal cell carcinoma (STEVIE): a pre-planned interim analysis of an international, open-label trial. Lancet Oncol. 2015;16:729–36. doi: 10.1016/S1470-2045(15)70198-1 . 25981813

[31] Bhutani T, Abrouk M, Sima CS, et al. Risk of cutaneous squamous cell carcinoma after treatment of basal cell carcinoma with vismodegib. J Am Acad Dermatol. 2017;77(4):713–8. doi: 10.1016/j.jaad.2017.03.038 . 28780365

[32] Mohan SV, Chang J, Li S, Henry AS, Wood DJ, Chang AL. Increased risk of cutaneous squamous cell carcinoma after vismodegib therapy for basal cell carcinoma. JAMA Dermat. 2016;152:527–32. doi: 10.1001/jamadermatol.2015.4330 . 26914338

[33] Alcalay J, Tauber G, Fenig E, Hodak E. Vismodegib as a neoadjuvant treatment to mohs surgery for aggressive Basal cell carcinoma. J Drugs Dermatol. 2015;14(3):219–21. S1545961615P0219X. . 25738842

[34] Ally MS, Aasi S, Wysong A, et al. An investigator-initiated open-label clinical trial of vismodegib as a neoadjuvant to surgery for high-risk basal cell carcinoma. J Am Acad Dermatol. 2014;71(5):904–11. doi: 10.1016/j.jaad.2014.05.020 . 24929884

[35] Chang AL, Atwood SX, Tartar DM, Oro AE. Surgical excision after neoadjuvant therapy with vismodegib for a locally advanced basal cell carcinoma and resistant basal carcinomas in Gorlin syndrome. JAMA Dermatol. 2013;149(5):639–41. doi: 10.1001/jamadermatol.2013.30 . 23677114 PMC4006072

[36] van Loo E, Mosterd K, Krekels GA, et al. Surgical excision versus Mohs’ micrographic surgery for basal cell carcinoma of the face: a randomised clinical trial with 10 year follow-up. Eur J Cancer. 2014;50(17):3011–20. doi: 10.1016/j.ejca.2014.08.018 . 25262378

[37] Pandeya N, Purdie DM, Green A, Williams G. Repeated occurrence of basal cell carcinoma of the skin and multifailure survival analysis: follow-up data from the Nambour Skin Cancer Prevention Trial. Am J Epidemiol. 2005;161(8):748–54. doi: 10.1093/aje/kwi098 . 15800267

[38] Maciel PC, Veiga-Filho J, Carvalho MP, Fonseca FE, Ferreira LM, Veiga DF. Quality of life and self-esteem in patients submitted to surgical treatment of skin carcinomas: long-term results. An Bras Dermatol. 2014;89(4):594–8. doi: 10.1590/abd1806-4841.20142742 . 25054746 PMC4148273

