# Supplementary figures and images for: Real-world assessment and treatment of locally advanced basal cell carcinoma: Findings from the RegiSONIC disease registry
Source: PLoS One. 2022 Jan 14;17(1):e0262151. doi: 10.1371/journal.pone.0262151 (PMC8759646; doi:10.1371/journal.pone.0262151)

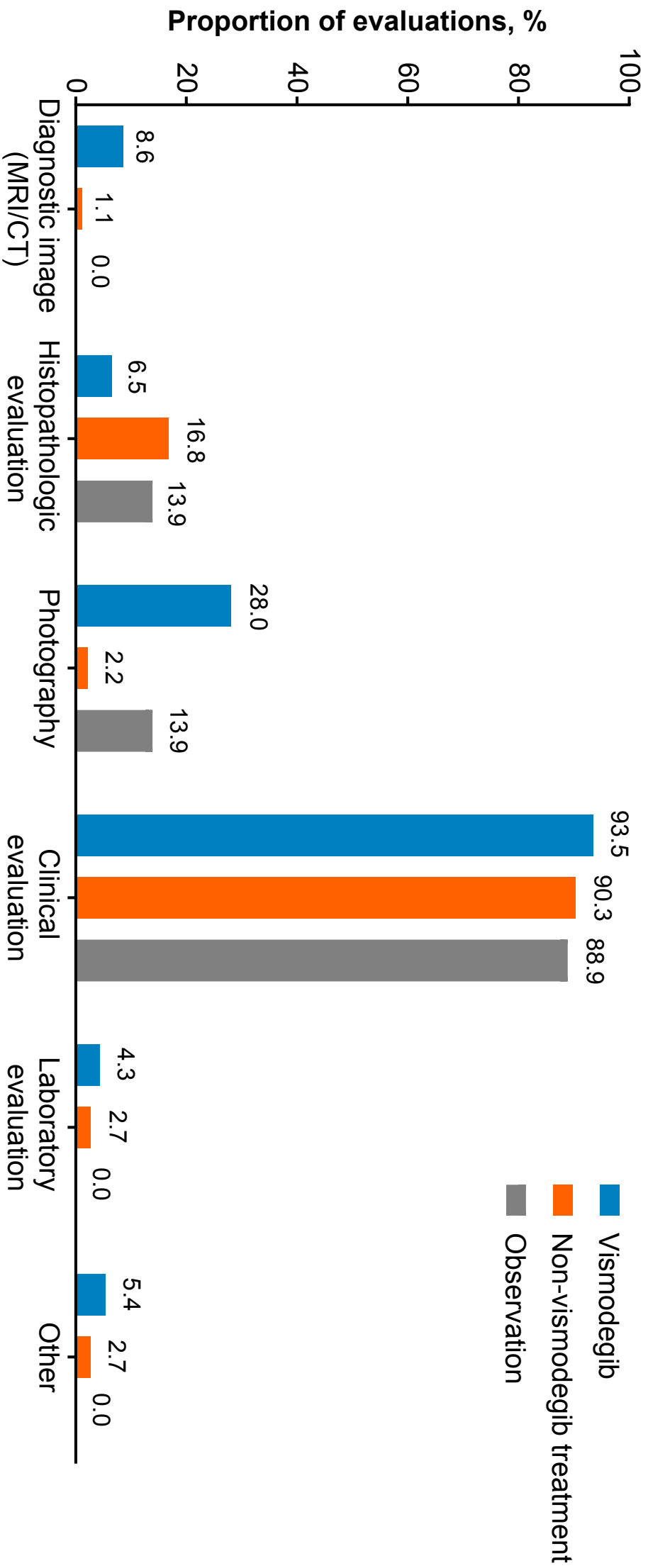

Supplement: S1 Fig — Abbreviations: CT, computed tomography; MRI, magnetic resonance imaging. Vismodegib = received vismodegib within 90 days of diagnosis; non-vismodegib treatment = received interventions other than vismodegib within 90 days of diagnosis; Observation = received no intervention within 90 days of diagnosis. (PDF) [file pone.0262151.s001.pdf]

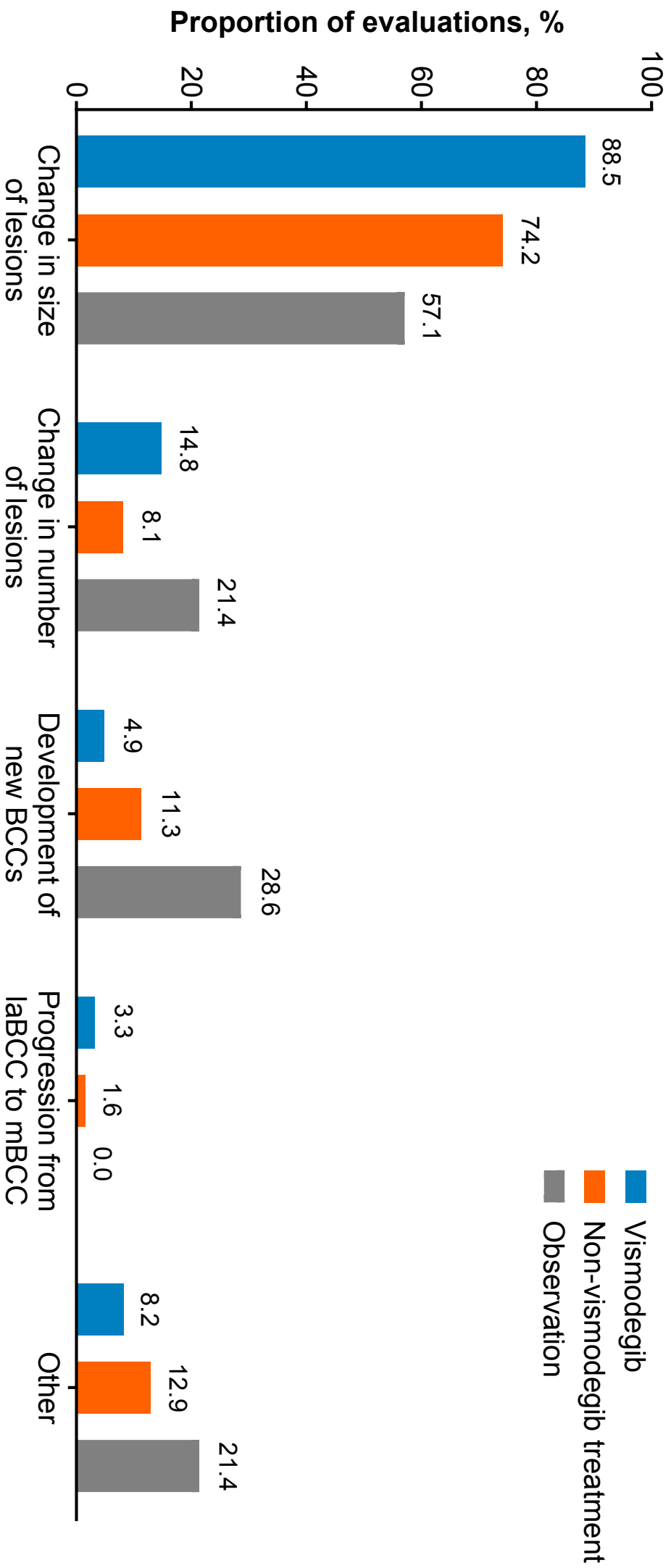

Supplement: S2 Fig — Abbreviations: BCC, basal cell carcinoma; laBCC, locally advanced basal cell carcinoma; mBCC, metastatic basal cell carcinoma. Vismodegib = received vismodegib within 90 days of diagnosis; non-vismodegib treatment = received interventions other than vismodegib within 90 days of diagnosis; Observation = received no intervention within 90 days of diagnosis. (PDF) [file pone.0262151.s002.pdf]

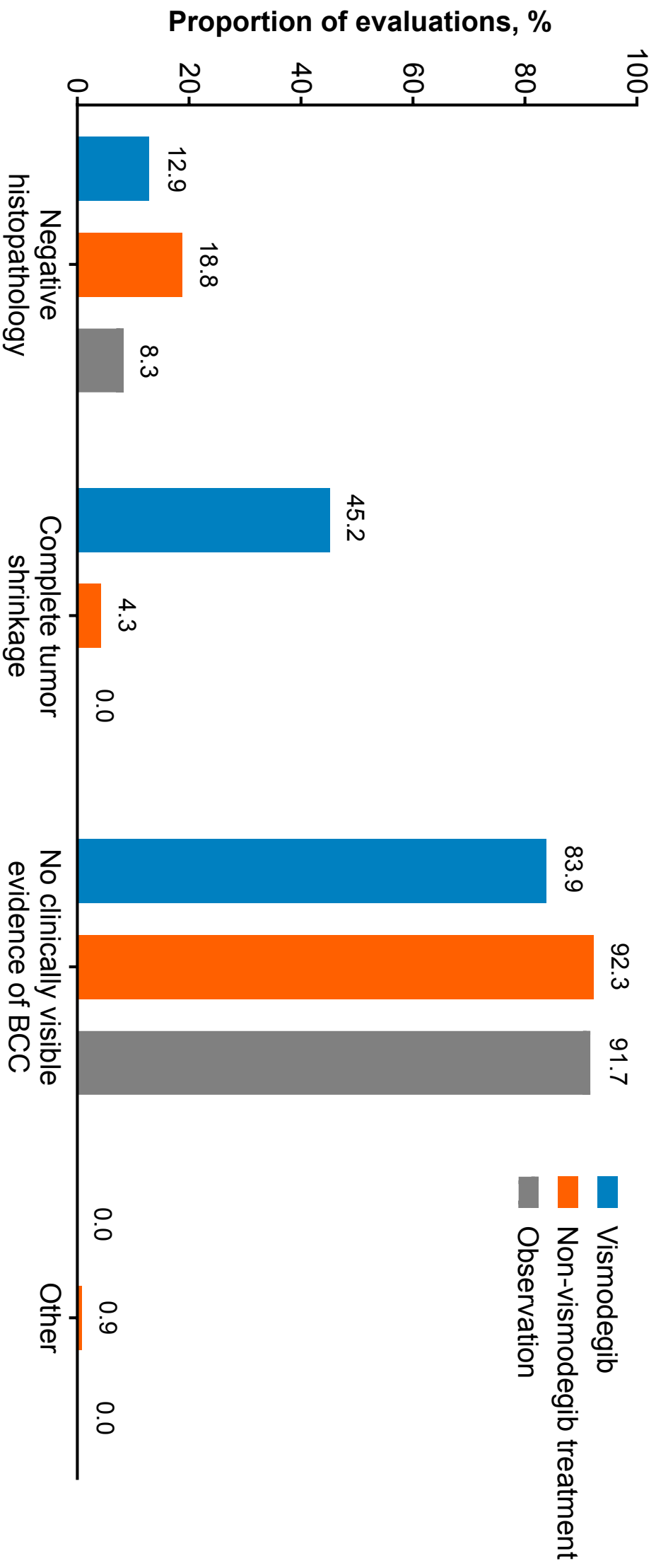

Supplement: S3 Fig — Abbreviations: BCC, basal cell carcinoma. Vismodegib = received vismodegib within 90 days of diagnosis; non-vismodegib treatment = received interventions other than vismodegib within 90 days of diagnosis; Observation = received no intervention within 90 days of diagnosis. (PDF) [file pone.0262151.s003.pdf]
