# Supplementary material for: Real-world assessment and treatment of locally advanced basal cell carcinoma: Findings from the RegiSONIC disease registry
Source: PLoS One. 2022 Jan 14;17(1):e0262151. doi: 10.1371/journal.pone.0262151 (PMC8759646; doi:10.1371/journal.pone.0262151)
Supplement: S1 Table — Abbreviations: BCC, basal cell carcinoma; laBCC, locally advanced basal cell carcinoma. aSpecific diagnoses occurring in 2 or more patients are reported. Vismodegib = received vismodegib within 90 days of diagnosis; non-vismodegib treatment = received interventions other than vismodegib within 90 days of diagnosis; observation = received no intervention within 90 days of diagnosis. (PDF) [file pone.0262151.s004.pdf]

S1 Table. Health insurance coverage, history of psychiatric disorders/dementia, and BCC treatment history in patients with locally advanced basal cell carcinoma in Cohort 1

	laBCC patients in Cohort 1			
**Characteristic**	**All n = 433**	**Vismodegib n = 115**	**Non-vismodegib treatment n = 251**	**Observation n = 67**
**Health insurance, n (%)**	n = 433	n = 115	n = 251	n = 67
Yes	402 (92.8)	102 (88.7)	236 (94.0)	64 (95.5)
No	31 (7.2)	13 (11.3)	15 (6.0)	3 (4.5)
**Type of insurance, n (%)**	n = 402	n = 102	n = 236	n = 64
Medicare	219 (54.5)	58 (56.9)	122 (51.7)	39 (60.9)
Medicaid	47 (11.7)	15 (14.7)	28 (11.9)	4 (6.3)
Private insurance	269 (66.9)	61 (59.8)	169 (71.6)	39 (60.9)
**History of psychiatric disorders/dementia, n (%)**	n = 433	n = 115	n = 251	n = 67
Psychiatric disorders[a]	98 (22.6)	24 (20.9)	55 (21.9)	19 (28.4)
Depression	50 (11.5)	12 (10.4)	28 (11.2)	10 (14.9)
Anxiety	38 (8.8)	16 (13.9)	18 (7.2)	4 (6.0)
Insomnia	21 (4.8)	3 (2.6)	13 (5.2)	5 (7.5)
Bipolar disorder	5 (1.2)	1 (0.9)	3 (1.2)	1 (1.5)
Claustrophobia	2 (0.5)	1 (0.9)	1 (0.4)	0
Psychotic disorder	2 (0.5)	1 (0.9)	1 (0.4)	0
Dementia/Alzheimer's disease	13 (3.0)	5 (4.3)	7 (2.8)	1 (1.5)
**Prior treatment history for target BCC, n (%)**	n = 176	n = 43	n = 100	n = 33
Surgery	132 (75.0)	39 (90.7)	71 (71.0)	22 (66.7)
Radiation	21 (11.9)	10 (23.3)	3 (3.0)	8 (24.2)
Topical treatment	19 (10.8)	6 (14.0)	8 (8.0)	5 (15.2)
Vismodegib	1 (0.6)	1 (2.3)	0	0
Other systemic treatment	1 (0.5)	1 (2.3)	0	0

Characteristic	laBCC patients in Cohort 1			
	**All n = 433**	**Vismodegib n = 115**	**Non-vismodegib treatment n = 251**	**Observation n = 67**
Other	3 (1.7)	1 (2.3)	0	2 (6.1)
None	34 (19.3)	1 (2.3)	26 (26.0)	7 (21.2)

Abbreviations: BCC, basal cell carcinoma; laBCC, locally advanced basal cell carcinoma.
[a] Specific diagnoses occurring in 2 or more patients are reported.
Vismodegib = received vismodegib within 90 days of diagnosis; non-vismodegib treatment = received interventions other than vismodegib within 90 days of diagnosis; observation = received no intervention within 90 days of diagnosis.
