# Supplementary material for: Real-world assessment and treatment of locally advanced basal cell carcinoma: Findings from the RegiSONIC disease registry
Source: PLoS One. 2022 Jan 14;17(1):e0262151. doi: 10.1371/journal.pone.0262151 (PMC8759646; doi:10.1371/journal.pone.0262151)
Supplement: S1 Data — (PDF) [file pone.0262151.s005.pdf]

Note: “Vismo” group is defined as initiating vismodegib less than or equal to 90 days after the date of determination of locally advanced BCC (laBCC) or metastatic BCC (mBCC); “No Vismo” group is defined as not initiating vismodegib (but other BCC treatment) less than or equal to 90 days after the date of determination of laBCC or mBCC; “No Treatment” group is defined as not receiving any BCC treatment less than or equal to 90 days after the date of determination of laBCC or mBCC.

“Vismo Only” and “Surgery Only” are defined as having only 1 of these treatments less than or equal to 90 days after the date of determination of laBCC or mBCC; “Other Therapy” includes individual or combination treatments (e.g., surgery, PDT, ED&C option nomprint nomlogic nosymbolgen option nomprint nomlogic nosymbolgen, topical treatment, cryosurgery, systemic treatment, other) initiated less than or equal to 90 days after date of determination of laBCC or mBCC. For example, if patient receives topical treatment followed by vismo followed by surgery, then all 3 treatments must be initiated within the 90 days of the date of determination of disease

[1] Patients with at least one quarterly update.

[2] Follow-up time in months is defined as (the earliest of the death date, termination date, and data cutoff date - informed consent date + 1) / 30.4375

[3] Denominator is the number of patients who terminated the study.

[4] Denominator is number of deaths.

[5] Optional DNA blood sample.

					Cohort 1: Newly Determined Advanced BCC Patients (Non-BCCNS) Locally Advanced BCC (laBCC)				
			Vismo (N=115)			No Vismo (N=251)			
	All (N=433)	All (N=115)	Vismo Only (N=102)	Surgery Only (N=6)	Other Therapy (N=7)	All (N=251)	Surgery Only (N=198)	Other Therapy (N=53)	No Treatment (N=67)
Patients Enrolled	433	115	102	6	7	251	198	53	67
Previously Enrolled in SHH4476G	NA	NA	NA	NA	NA	NA	NA	NA	NA
Previously Enrolled in SHH4811G	NA	NA	NA	NA	NA	NA	NA	NA	NA
Previously Enrolled in SHH4437G	NA	NA	NA	NA	NA	NA	NA	NA	NA
Patients with Post-baseline Information [1]	412	112	100	6	6	242	190	52	58
Median and Range of Follow-up (months) [2]	23.6 (0.03,45.04)	25.4 (0.16,39.56)	24.9 (1.02,37.82)	33.4 (23.10,39.56)	27.2 (0.16,36.14)	22.8 (0.03,45.04)	21.5 (0.03,43.14)	26.3 (2.79,45.04)	21.2 (0.03,44.25)

					Cohort 1: Newly Determined Advanced BCC Patients (Non-BCCNS) Metastatic BCC (mBCC)				
		Vismo (N=2)			No Vismo (N=1)				
	All (N=4)	All (N=2)	Vismo Only (N=2)	Surgery Only (N=0)	Other Therapy (N=0)	All (N=1)	Surgery Only (N=1)	Other Therapy (N=0)	No Treatment (N=1)
Patients Enrolled	4	2	2	0	0	1	1	0	1
Previously Enrolled in SHH4476G	NA	NA	NA	NA	NA	NA	NA	NA	NA
Previously Enrolled in SHH4811G	NA	NA	NA	NA	NA	NA	NA	NA	NA
Previously Enrolled in SHH4437G	NA	NA	NA	NA	NA	NA	NA	NA	NA
Patients with Post-baseline Information [1]	4	2	2	0	0	1	1	0	1
Median and Range of Follow-up (months) [2]	25.1 (18.30,33.45)	19.7 (18.30,21.06)	19.7 (18.30,21.06)	-	-	29.2 (29.21,29.21)	29.2 (29.21,29.21)	-	33.4 (33.45,33.45)

					Cohort 1: Newly Determined Advanced BCC Patients (Non-BCCNS) Locally Advanced BCC (laBCC)				
		Vismo (N=115)			No Vismo (N=251)				
	All (N=433)	All (N=115)	Vismo Only (N=102)	Surgery Only (N=6)	Other Therapy (N=7)	All (N=251)	Surgery Only (N=198)	Other Therapy (N=53)	No Treatment (N=67)
Patients Terminated the Study	433(100%)	115(100%)	102(100%)	6(100%)	7(100%)	251(100%)	198(100%)	53(100%)	67(100%)
Reason for Termination [3]									
n	433	115	102	6	7	251	198	53	67
Completed Study	69(15.9%)	29(25.2%)	26(25.5%)	1(16.7%)	2(28.6%)	31(12.4%)	20(10.1%)	11(20.8%)	9(13.4%)

Death	37(8.5%)	16(13.9%)	14(13.7%)	0	2(28.6%)	14(5.6%)	9(4.5%)	5(9.4%)	7(10.4%)
Cause of Death [4]									
BCC Disease Progression	2(5.4%)	2(12.5%)	2(14.3%)	0	0	0	0	0	0
Adverse Event	35(94.6%)	14(87.5%)	12(85.7%)	0	2(100%)	14(100%)	9(100%)	5(100%)	7(100%)
Lost to Follow-up	83(19.2%)	20(17.4%)	20(19.6%)	0	0	51(20.3%)	40(20.2%)	11(20.8%)	12(17.9%)
Physician's Decision	31(7.2%)	3(2.6%)	3(2.9%)	0	0	23(9.2%)	20(10.1%)	3(5.7%)	5(7.5%)
Patient's Decision to Withdraw Consent	55(12.7%)	13(11.3%)	12(11.8%)	0	1(14.3%)	32(12.7%)	29(14.6%)	3(5.7%)	10(14.9%)
Sponsor's Decision to Terminate Study	150(34.6%)	33(28.7%)	27(26.5%)	5(83.3%)	1(14.3%)	99(39.4%)	79(39.9%)	20(37.7%)	18(26.9%)
Patient Not Eligible for Study	8(1.8%)	1(0.9%)	0	0	1(14.3%)	1(0.4%)	1(0.5%)	0	6(9.0%)
Other	0	0	0	0	0	0	0	0	0

					Cohort 1: Newly Determined Advanced BCC Patients (Non-BCCNS) Metastatic BCC (mBCC)				
			Vismo (N=2)		No Vismo (N=1)				
	All (N=4)	All (N=2)	Vismo Only (N=2)	Surgery Only (N=0)	Other Therapy (N=0)	All (N=1)	Surgery Only (N=1)	Other Therapy (N=0)	No Treatment (N=1)
Patients Terminated the Study	4(100%)	2(100%)	2(100%)	0	0	1(100%)	1(100%)	0	1(100%)

Reason for Termination [3]									
n	4	2	2	0	0	1	1	0	1
Completed Study	0	0	0	0	0	0	0	0	0
Death	0	0	0	0	0	0	0	0	0
Cause of Death [4]									
BCC Disease Progression	0	0	0	0	0	0	0	0	0
Adverse Event	0	0	0	0	0	0	0	0	0
Lost to Follow-up	0	0	0	0	0	0	0	0	0
Physician's Decision	0	0	0	0	0	0	0	0	0
Patient's Decision to Withdraw Consent	1(25.0%)	1(50.0%)	1(50.0%)	0	0	0	0	0	0
Sponsor's Decision to Terminate Study	3(75.0%)	1(50.0%)	1(50.0%)	0	0	1(100%)	1(100%)	0	1(100%)
Patient Not Eligible for Study	0	0	0	0	0	0	0	0	0
Other	0	0	0	0	0	0	0	0	0
					Cohort 1: Newly Determined Advanced BCC Patients (Non-BCCNS) Locally Advanced BCC (laBCC)				
			Vismo (N=115)		No Vismo (N=251)				
	All (N=433)	All (N=115)	Vismo Only (N=102)	Surgery Only (N=6)	Other Therapy (N=7)	All (N=251)	Surgery Only (N=198)	Other Therapy (N=53)	No Treatment (N=67)

Patient Consent for Blood Sample [5]									
n	433	115	102	6	7	251	198	53	67
Yes	106(24.5%)	48(41.7%)	43(42.2%)	3(50.0%)	2(28.6%)	39(15.5%)	35(17.7%)	4(7.5%)	19(28.4%)
No	327(75.5%)	67(58.3%)	59(57.8%)	3(50.0%)	5(71.4%)	212(84.5%)	163(82.3%)	49(92.5%)	48(71.6%)
Patient Consent for Photography Sub-study?									
n	433	115	102	6	7	251	198	53	67
Yes	70(16.2%)	38(33.0%)	36(35.3%)	1(16.7%)	1(14.3%)	20(8.0%)	17(8.6%)	3(5.7%)	12(17.9%)
No	72(16.6%)	14(12.2%)	9(8.8%)	1(16.7%)	4(57.1%)	49(19.5%)	31(15.7%)	18(34.0%)	9(13.4%)
Not Applicable	291(67.2%)	63(54.8%)	57(55.9%)	4(66.7%)	2(28.6%)	182(72.5%)	150(75.8%)	32(60.4%)	46(68.7%)
Patient Consent for Diagnostic Imaging Sub-Study									
n	433	115	102	6	7	251	198	53	67
Yes	39(9.0%)	16(13.9%)	14(13.7%)	1(16.7%)	1(14.3%)	16(6.4%)	12(6.1%)	4(7.5%)	7(10.4%)
No	69(15.9%)	13(11.3%)	9(8.8%)	0	4(57.1%)	48(19.1%)	29(14.6%)	19(35.8%)	8(11.9%)
Not Applicable	325(75.1%)	86(74.8%)	79(77.5%)	5(83.3%)	2(28.6%)	187(74.5%)	157(79.3%)	30(56.6%)	52(77.6%)
					Cohort 1: Newly Determined Advanced BCC Patients (Non-BCCNS) Metastatic BCC (mBCC)				
		Vismo (N=2)			No Vismo (N=1)				
	All (N=4)	All (N=2)	Vismo Only (N=2)	Surgery Only (N=0)	Other Therapy (N=0)	All (N=1)	Surgery Only (N=1)	Other Therapy (N=0)	No Treatment (N=1)
Patient Consent for Blood Sample [5]									
n	4	2	2	0	0	1	1	0	1
Yes	3(75.0%)	1(50.0%)	1(50.0%)	0	0	1(100%)	1(100%)	0	1(100%)
No	1(25.0%)	1(50.0%)	1(50.0%)	0	0	0	0	0	0

Patient Consent for Photography Sub-study?									
n	4	2	2	0	0	1	1	0	1
Yes	3(75.0%)	2(100%)	2(100%)	0	0	0	0	0	1(100%)
No	0	0	0	0	0	0	0	0	0
Not Applicable	1(25.0%)	0	0	0	0	1(100%)	1(100%)	0	0
Patient Consent for Diagnostic Imaging Sub-Study									
n	4	2	2	0	0	1	1	0	1
Yes	2(50.0%)	1(50.0%)	1(50.0%)	0	0	0	0	0	1(100%)
No	0	0	0	0	0	0	0	0	0
Not Applicable	2(50.0%)	1(50.0%)	1(50.0%)	0	0	1(100%)	1(100%)	0	0

Note: “Vismo” group is defined as initiating vismodegib less than or equal to 90 days after the date of enrollment; “No Vismo” group is defined as not initiating vismodegib (but other BCC treatment) less than or equal to 90 days after the date of enrollment; “No Treatment” group is defined as not receiving any BCC treatment less than or equal to 90 days after the date of enrollment.

“Vismo Only” and “Surgery Only” are defined as having only 1 of these treatments less than or equal to 90 days after the date of enrollment; “Other Therapy” includes individual or combination treatments (e.g., surgery, PDT, ED&C *** DEFINE FORMAT *** *** DEFINE FORMAT ***, topical treatment, cryosurgery, systemic treatment, other) initiated less than or equal to 90 days after date of enrollment. For example, if patient receives topical treatment followed by vismo followed by surgery, then all 3 treatments must be initiated within the 90 days of the date of enrollment.

[1] Patients with at least one quarterly update.

[2] Follow-up time in months is defined as (the earliest of the death date, termination date, and data cutoff date - informed consent date + 1) / 30.4375

[3] Denominator is the number of patients who terminated the study.

[4] Denominator is number of deaths.

[5] Optional DNA blood sample.

	Cohort 2: Advanced BCC Patients (non-BCCNS) in a Prior GNE trial Locally Advanced BCC (laBCC)								
	Vismo (N=1)	No Vismo (N=0)							
	All (N=4)	All (N=1)	Vismo Only (N=1)	Surgery Only (N=0)	Other Therapy (N=0)	All (N=0)	Surgery Only (N=0)	Other Therapy (N=0)	No Treatment (N=3)
Patients Enrolled	4	1	1	0	0	0	0	0	3
Previously Enrolled in SHH4476G	2(50.0%)	1(100%)	1(100%)	0	0	0	0	0	1(33.3%)
Previously Enrolled in SHH4811G	2(50.0%)	0	0	0	0	0	0	0	2(66.7%)
Previously Enrolled in SHH4437G	0	0	0	0	0	0	0	0	0
Patients with Post-baseline Information [1]	4	1	1	0	0	0	0	0	3
Median and Range of Follow-up (months) [2]	24.3 (5.26,35.65)	34.8 (34.76,34.76)	34.8 (34.76,34.76)	-	-	-	-	-	13.8 (5.26,35.65)

	Cohort 2: Advanced BCC Patients (non-BCCNS) in a Prior GNE trial Metastatic BCC (mBCC)		
	Vismo (N=5)	No Vismo (N=0)	

	All (N=5)	All (N=5)	Vismo Only (N=5)	Surgery Only (N=0)	Other Therapy (N=0)	All (N=0)	Surgery Only (N=0)	Other Therapy (N=0)	No Treatment (N=0)
Patients Enrolled	5	5	5	0	0	0	0	0	0
Previously Enrolled in SHH4476G	1(20.0%)	1(20.0%)	1(20.0%)	0	0	0	0	0	0
Previously Enrolled in SHH4811G	4(80.0%)	4(80.0%)	4(80.0%)	0	0	0	0	0	0
Previously Enrolled in SHH4437G	0	0	0	0	0	0	0	0	0
Patients with Post-baseline Information [1]	5	5	5	0	0	0	0	0	0
Median and Range of Follow-up (months) [2]	22.9 (7.16,37.52)	22.9 (7.16,37.52)	22.9 (7.16,37.52)	-	-	-	-	-	-
	Cohort 2: Advanced BCC Patients (non-BCCNS) in a Prior GNE trial Locally Advanced BCC (laBCC)								
	Vismo (N=1)	No Vismo (N=0)							
	All (N=4)	All (N=1)	Vismo Only (N=1)	Surgery Only (N=0)	Other Therapy (N=0)	All (N=0)	Surgery Only (N=0)	Other Therapy (N=0)	No Treatment (N=3)
Patients Terminated the Study	4(100%)	1(100%)	1(100%)	0	0	0	0	0	3(100%)
Reason for Terminatio n [3]									
n	4	1	1	0	0	0	0	0	3
Completed Study	2(50.0%)	1(100%)	1(100%)	0	0	0	0	0	1(33.3%)
Death	1(25.0%)	0	0	0	0	0	0	0	1(33.3%)

Cause of Death [4]									
BCC Disease Progression	1(100%)	0	0	0	0	0	0	0	1(100%)
Adverse Event	0	0	0	0	0	0	0	0	0
Lost to Follow-up	0	0	0	0	0	0	0	0	0
Physician's Decision	0	0	0	0	0	0	0	0	0
Patient's Decision to Withdraw Consent	1(25.0%)	0	0	0	0	0	0	0	1(33.3%)
Sponsor's Decision to Terminate Study	0	0	0	0	0	0	0	0	0
Patient Not Eligible for Study	0	0	0	0	0	0	0	0	0
Other	0	0	0	0	0	0	0	0	0
	Cohort 2: Advanced BCC Patients (non-BCCNS) in a Prior GNE trial Metastatic BCC (mBCC)								
	Vismo (N=5)	No Vismo (N=0)							
	All (N=5)	All (N=5)	Vismo Only (N=5)	Surgery Only (N=0)	Other Therapy (N=0)	All (N=0)	Surgery Only (N=0)	Other Therapy (N=0)	No Treatment (N=0)
Patients Terminated the Study	5(100%)	5(100%)	5(100%)	0	0	0	0	0	0
Reason for Termination [3]									

n	5	5	5	0	0	0	0	0	0
Completed Study	1(20.0%)	1(20.0%)	1(20.0%)	0	0	0	0	0	0
Death	2(40.0%)	2(40.0%)	2(40.0%)	0	0	0	0	0	0
Cause of Death [4]									
BCC Disease Progression	2(100%)	2(100%)	2(100%)	0	0	0	0	0	0
Adverse Event	0	0	0	0	0	0	0	0	0
Lost to Follow-up	0	0	0	0	0	0	0	0	0
Physician's Decision	1(20.0%)	1(20.0%)	1(20.0%)	0	0	0	0	0	0
Patient's Decision to Withdraw Consent	1(20.0%)	1(20.0%)	1(20.0%)	0	0	0	0	0	0
Sponsor's Decision to Terminate Study	0	0	0	0	0	0	0	0	0
Patient Not Eligible for Study	0	0	0	0	0	0	0	0	0
Other	0	0	0	0	0	0	0	0	0
	Cohort 2: Advanced BCC Patients (non-BCCNS) in a Prior GNE trial Locally Advanced BCC (laBCC)								
	Vismo (N=1)	No Vismo (N=0)							
	All (N=4)	All (N=1)	Vismo Only (N=1)	Surgery Only (N=0)	Other Therapy (N=0)	All (N=0)	Surgery Only (N=0)	Other Therapy (N=0)	No Treatment (N=3)

Patient Consent for Blood Sample [5]									
n	4	1	1	0	0	0	0	0	3
Yes	4(100%)	1(100%)	1(100%)	0	0	0	0	0	3(100%)
No	0	0	0	0	0	0	0	0	0
Patient Consent for Photography Sub-study?									
n	4	1	1	0	0	0	0	0	3
Yes	0	0	0	0	0	0	0	0	0
No	0	0	0	0	0	0	0	0	0
Not Applicable	4(100%)	1(100%)	1(100%)	0	0	0	0	0	3(100%)
Patient Consent for Diagnostic Imaging Sub-Study									
n	4	1	1	0	0	0	0	0	3
Yes	0	0	0	0	0	0	0	0	0
No	0	0	0	0	0	0	0	0	0
Not Applicable	4(100%)	1(100%)	1(100%)	0	0	0	0	0	3(100%)
	Cohort 2: Advanced BCC Patients (non-BCCNS) in a Prior GNE trial Metastatic BCC (mBCC)								
	Vismo (N=5)	No Vismo (N=0)							
	All (N=5)	All (N=5)	Vismo Only (N=5)	Surgery Only (N=0)	Other Therapy (N=0)	All (N=0)	Surgery Only (N=0)	Other Therapy (N=0)	No Treatment (N=0)
Patient Consent for Blood Sample [5]									
n	5	5	5	0	0	0	0	0	0
Yes	3(60.0%)	3(60.0%)	3(60.0%)	0	0	0	0	0	0
No	2(40.0%)	2(40.0%)	2(40.0%)	0	0	0	0	0	0
Patient Consent for Photography Sub-study?									

n	5	5	5	0	0	0	0	0	0
Yes	0	0	0	0	0	0	0	0	0
No	0	0	0	0	0	0	0	0	0
Not Applicable	5(100%)	5(100%)	5(100%)	0	0	0	0	0	0
Patient Consent for Diagnostic Imaging Sub-Study									
n	5	5	5	0	0	0	0	0	0
Yes	0	0	0	0	0	0	0	0	0
No	0	0	0	0	0	0	0	0	0
Not Applicable	5(100%)	5(100%)	5(100%)	0	0	0	0	0	0

Note: Reference date: date of enrollment for patients with prior vismo treatment during participation in GNE studies; date of determination of locally advanced BCC or multiple non-advanced BCC or metastatic BCC for patients who didn’t participate in previous GNE vismo studies. “Vismo” group is defined as initiating vismodegib less than or equal to 90 days after the reference date; “No Vismo” group is defined as not initiating vismodegib (but other BCC treatment) less than or equal to 90 days after the reference date; “No Treatment” group is defined as not receiving any BCC treatment less than or equal to 90 days after the reference date.

“Vismo Only” and “Surgery Only” are defined as having only 1 of these treatments less than or equal to 90 days after the reference date; “Other Therapy” includes individual or combination treatments (e.g., surgery, PDT, ED&C, topical treatment, cryosurgery, systemic treatment, other) initiated less than or equal to 90 days after the reference date. For example, if patient receives topical treatment followed by vismo followed by surgery, then all 3 treatments must be initiated within the 90 days of the reference date.

[1] Patients with at least one quarterly update.

[2] Follow-up time in months is defined as (the earliest of the death date, termination date, and data cutoff date - informed consent date + 1) / 30.4375

[3] Denominator is the number of patients who terminated the study.

[4] Denominator is number of deaths.

[5] Optional DNA blood sample.

	Cohort 3: BCCNS Patients with Advanced BCC or Multiple Non-Advanced BCC - Multiple Non-Advanced BCC								
		Vismo (N=10)				No Vismo (N=18)			
	All (N=36)	All (N=10)	Vismo Only (N=6)	Surgery Only (N=1)	Other Therapy (N=3)	All (N=18)	Surgery Only (N=14)	Other Therapy (N=4)	No Treatment (N=8)
Patients Enrolled	36	10	6	1	3	18	14	4	8
Previously Enrolled in SHH4476G	NA	NA	NA	NA	NA	NA	NA	NA	NA
Previously Enrolled in SHH4811G	NA	NA	NA	NA	NA	NA	NA	NA	NA
Previously Enrolled in SHH4437G	NA	NA	NA	NA	NA	NA	NA	NA	NA
Patients with Post-baseline Information [1]	36	10	6	1	3	18	14	4	8
Median and Range of Follow-up (months) [2]	24.0 (0.10,43.27)	32.0 (5.75,38.47)	32.1 (5.75,38.47)	24.5 (24.54,24.54)	35.4 (20.63,36.76)	25.2 (1.64,43.27)	21.6 (1.64,43.27)	33.1 (24.67,37.62)	14.0 (0.10,36.67)

					Cohort 3: BCCNS Patients with Advanced BCC or Multiple Non-Advanced BCC - Locally Advanced BCC (laBCC)				
		Vismo (N=9)				No Vismo (N=1)			
	All (N=16)	All (N=9)	Vismo Only (N=6)	Surgery Only (N=3)	Other Therapy (N=0)	All (N=1)	Surgery Only (N=1)	Other Therapy (N=0)	No Treatment (N=6)
Patients Enrolled	16	9	6	3	0	1	1	0	6
Previously Enrolled in SHH4476G	1(6.3%)	0	0	0	0	0	0	0	1(16.7%)
Previously Enrolled in SHH4811G	1(6.3%)	1(11.1%)	1(16.7%)	0	0	0	0	0	0
Previously Enrolled in SHH4437G	0	0	0	0	0	0	0	0	0
Patients with Post-baseline Information [1]	16	9	6	3	0	1	1	0	6
Median and Range of Follow-up (months) [2]	24.5 (0.03,39.82)	34.6 (2.86,39.82)	29.6 (2.86,39.82)	37.3 (27.89,37.95)	-	36.0 (36.04,36.04)	36.0 (36.04,36.04)	-	18.2 (0.03,24.25)
					Cohort 3: BCCNS Patients with Advanced BCC or Multiple Non-Advanced BCC - Metastatic BCC (mBCC)				
		Vismo (N=1)				No Vismo (N=0)			
	All (N=2)	All (N=1)	Vismo Only (N=1)	Surgery Only (N=0)	Other Therapy (N=0)	All (N=0)	Surgery Only (N=0)	Other Therapy (N=0)	No Treatment (N=1)
Patients Enrolled	2	1	1	0	0	0	0	0	1
Previously Enrolled in SHH4476G	0	0	0	0	0	0	0	0	0

Previously Enrolled in SHH4811G	1(50.0%)	1(100%)	1(100%)	0	0	0	0	0	0
Previously Enrolled in SHH4437G	0	0	0	0	0	0	0	0	0
Patients with Post-baseline Information [1]	2	1	1	0	0	0	0	0	1
Median and Range of Follow-up (months) [2]	12.6 (6.41,18.86)	18.9 (18.86,18.86)	18.9 (18.86,18.86)	-	-	-	-	-	6.4 (6.41,6.41)
					Cohort 3: BCCNS Patients with Advanced BCC or Multiple Non-Advanced BCC - Multiple Non-Advanced BCC				
		Vismo (N=10)			No Vismo (N=18)				
	All (N=36)	All (N=10)	Vismo Only (N=6)	Surgery Only (N=1)	Other Therapy (N=3)	All (N=18)	Surgery Only (N=14)	Other Therapy (N=4)	No Treatment (N=8)
Patients Terminated the Study	36(100%)	10(100%)	6(100%)	1(100%)	3(100%)	18(100%)	14(100%)	4(100%)	8(100%)
Reason for Termination [3]									
n	36	10	6	1	3	18	14	4	8
Completed Study	9(25.0%)	3(30.0%)	2(33.3%)	0	1(33.3%)	5(27.8%)	4(28.6%)	1(25.0%)	1(12.5%)
Death	1(2.8%)	0	0	0	0	1(5.6%)	1(7.1%)	0	0
Cause of Death [4]									
BCC Disease Progression	0	0	0	0	0	0	0	0	0

Adverse Event	1(100%)	0	0	0	0	1(100%)	1(100%)	0	0
Lost to Follow-up	8(22.2%)	3(30.0%)	2(33.3%)	0	1(33.3%)	4(22.2%)	2(14.3%)	2(50.0%)	1(12.5%)
Physician's Decision	3(8.3%)	0	0	0	0	2(11.1%)	2(14.3%)	0	1(12.5%)
Patient's Decision to Withdraw Consent	7(19.4%)	1(10.0%)	1(16.7%)	0	0	3(16.7%)	2(14.3%)	1(25.0%)	3(37.5%)
Sponsor's Decision to Terminate Study	4(11.1%)	3(30.0%)	1(16.7%)	1(100%)	1(33.3%)	1(5.6%)	1(7.1%)	0	0
Patient Not Eligible for Study	3(8.3%)	0	0	0	0	2(11.1%)	2(14.3%)	0	1(12.5%)
Other	1(2.8%)	0	0	0	0	0	0	0	1(12.5%)
					Cohort 3: BCCNS Patients with Advanced BCC or Multiple Non-Advanced BCC - Locally Advanced BCC (laBCC)				
		Vismo (N=9)				No Vismo (N=1)			
	All (N=16)	All (N=9)	Vismo Only (N=6)	Surgery Only (N=3)	Other Therapy (N=0)	All (N=1)	Surgery Only (N=1)	Other Therapy (N=0)	No Treatment (N=6)
Patients Terminated the Study	16(100%)	9(100%)	6(100%)	3(100%)	0	1(100%)	1(100%)	0	6(100%)
Reason for Termination [3]									
n	16	9	6	3	0	1	1	0	6
Completed Study	6(37.5%)	5(55.6%)	3(50.0%)	2(66.7%)	0	1(100%)	1(100%)	0	0
Death	1(6.3%)	1(11.1%)	1(16.7%)	0	0	0	0	0	0
Cause of Death [4]									

BCC Disease Progression	0	0	0	0	0	0	0	0	0
Adverse Event	1(100%)	1(100%)	1(100%)	0	0	0	0	0	0
Lost to Follow-up	3(18.8%)	1(11.1%)	1(16.7%)	0	0	0	0	0	2(33.3%)
Physician's Decision	1(6.3%)	1(11.1%)	0	1(33.3%)	0	0	0	0	0
Patient's Decision to Withdraw Consent	4(25.0%)	1(11.1%)	1(16.7%)	0	0	0	0	0	3(50.0%)
Sponsor's Decision to Terminate Study	1(6.3%)	0	0	0	0	0	0	0	1(16.7%)
Patient Not Eligible for Study	0	0	0	0	0	0	0	0	0
Other	0	0	0	0	0	0	0	0	0
					Cohort 3: BCCNS Patients with Advanced BCC or Multiple Non-Advanced BCC - Metastatic BCC (mBCC)				
			Vismo (N=1)		No Vismo (N=0)				
	All (N=2)	All (N=1)	Vismo Only (N=1)	Surgery Only (N=0)	Other Therapy (N=0)	All (N=0)	Surgery Only (N=0)	Other Therapy (N=0)	No Treatment (N=1)
Patients Terminated the Study	2(100%)	1(100%)	1(100%)	0	0	0	0	0	1(100%)
Reason for Termination [3]									
n	2	1	1	0	0	0	0	0	1
Completed Study	0	0	0	0	0	0	0	0	0
Death	1(50.0%)	0	0	0	0	0	0	0	1(100%)

Cause of Death [4]									
BCC Disease Progression	1(100%)	0	0	0	0	0	0	0	1(100%)
Adverse Event	0	0	0	0	0	0	0	0	0
Lost to Follow-up	0	0	0	0	0	0	0	0	0
Physician's Decision	0	0	0	0	0	0	0	0	0
Patient's Decision to Withdraw Consent	1(50.0%)	1(100%)	1(100%)	0	0	0	0	0	0
Sponsor's Decision to Terminate Study	0	0	0	0	0	0	0	0	0
Patient Not Eligible for Study	0	0	0	0	0	0	0	0	0
Other	0	0	0	0	0	0	0	0	0

					Cohort 3: BCCNS Patients with Advanced BCC or Multiple Non-Advanced BCC - Multiple Non-Advanced BCC				
		Vismo (N=10)			No Vismo (N=18)				
	All (N=36)	All (N=10)	Vismo Only (N=6)	Surgery Only (N=1)	Other Therapy (N=3)	All (N=18)	Surgery Only (N=14)	Other Therapy (N=4)	No Treatment (N=8)
Patient Consent for Blood Sample [5]									
n	36	10	6	1	3	18	14	4	8
Yes	13(36.1%)	4(40.0%)	3(50.0%)	1(100%)	0	6(33.3%)	2(14.3%)	4(100%)	3(37.5%)

No	23(63.9%)	6(60.0%)	3(50.0%)	0	3(100%)	12(66.7%)	12(85.7%)	0	5(62.5%)
Patient Consent for Photography Sub-study?									
n	36	10	6	1	3	18	14	4	8
Yes	10(27.8%)	2(20.0%)	2(33.3%)	0	0	6(33.3%)	6(42.9%)	0	2(25.0%)
No	6(16.7%)	3(30.0%)	1(16.7%)	0	2(66.7%)	3(16.7%)	3(21.4%)	0	0
Not Applicable	20(55.6%)	5(50.0%)	3(50.0%)	1(100%)	1(33.3%)	9(50.0%)	5(35.7%)	4(100%)	6(75.0%)
Patient Consent for Diagnostic Imaging Sub-Study									
n	36	10	6	1	3	18	14	4	8
Yes	2(5.6%)	1(10.0%)	1(16.7%)	0	0	1(5.6%)	1(7.1%)	0	0
No	9(25.0%)	2(20.0%)	0	0	2(66.7%)	7(38.9%)	7(50.0%)	0	0
Not Applicable	25(69.4%)	7(70.0%)	5(83.3%)	1(100%)	1(33.3%)	10(55.6%)	6(42.9%)	4(100%)	8(100%)
					Cohort 3: BCCNS Patients with Advanced BCC or Multiple Non-Advanced BCC - Locally Advanced BCC (laBCC)				
		Vismo (N=9)			No Vismo (N=1)				
	All (N=16)	All (N=9)	Vismo Only (N=6)	Surgery Only (N=3)	Other Therapy (N=0)	All (N=1)	Surgery Only (N=1)	Other Therapy (N=0)	No Treatment (N=6)
Patient Consent for Blood Sample [5]									
n	16	9	6	3	0	1	1	0	6
Yes	8(50.0%)	7(77.8%)	5(83.3%)	2(66.7%)	0	0	0	0	1(16.7%)
No	8(50.0%)	2(22.2%)	1(16.7%)	1(33.3%)	0	1(100%)	1(100%)	0	5(83.3%)
Patient Consent for Photography Sub-study?									
n	16	9	6	3	0	1	1	0	6
Yes	5(31.3%)	3(33.3%)	1(16.7%)	2(66.7%)	0	1(100%)	1(100%)	0	1(16.7%)
No	4(25.0%)	3(33.3%)	3(50.0%)	0	0	0	0	0	1(16.7%)
Not Applicable	7(43.8%)	3(33.3%)	2(33.3%)	1(33.3%)	0	0	0	0	4(66.7%)

Patient Consent for Diagnostic Imaging Sub-Study									
n	16	9	6	3	0	1	1	0	6
Yes	1(6.3%)	1(11.1%)	0	1(33.3%)	0	0	0	0	0
No	5(31.3%)	3(33.3%)	3(50.0%)	0	0	1(100%)	1(100%)	0	1(16.7%)
Not Applicable	10(62.5%)	5(55.6%)	3(50.0%)	2(66.7%)	0	0	0	0	5(83.3%)
					Cohort 3: BCCNS Patients with Advanced BCC or Multiple Non-Advanced BCC - Metastatic BCC (mBCC)				
			Vismo (N=1)			No Vismo (N=0)			
	All (N=2)	All (N=1)	Vismo Only (N=1)	Surgery Only (N=0)	Other Therapy (N=0)	All (N=0)	Surgery Only (N=0)	Other Therapy (N=0)	No Treatment (N=1)
Patient Consent for Blood Sample [5]									
n	2	1	1	0	0	0	0	0	1
Yes	1(50.0%)	0	0	0	0	0	0	0	1(100%)
No	1(50.0%)	1(100%)	1(100%)	0	0	0	0	0	0
Patient Consent for Photography Sub-study?									
n	2	1	1	0	0	0	0	0	1
Yes	1(50.0%)	0	0	0	0	0	0	0	1(100%)
No	0	0	0	0	0	0	0	0	0
Not Applicable	1(50.0%)	1(100%)	1(100%)	0	0	0	0	0	0
Patient Consent for Diagnostic Imaging Sub-Study									
n	2	1	1	0	0	0	0	0	1
Yes	0	0	0	0	0	0	0	0	0
No	0	0	0	0	0	0	0	0	0
Not Applicable	2(100%)	1(100%)	1(100%)	0	0	0	0	0	1(100%)

Note: “Vismo” group is defined as initiating vismodegib less than or equal to 90 days after the date of determination of locally advanced BCC (laBCC) or metastatic BCC (mBCC); “No Vismo” group is defined as not initiating vismodegib (but other BCC treatment) less than or equal to 90 days after the date of determination of laBCC or mBCC; “No Treatment” group is defined as not receiving any BCC treatment less than or equal to 90 days after the date of determination of laBCC or mBCC.

“Vismo Only” and “Surgery Only” are defined as having only 1 of these treatments less than or equal to 90 days after the date of determination of laBCC or mBCC; “Other Therapy” includes individual or combination treatments (e.g., surgery, PDT, ED&C, topical treatment, cryosurgery, systemic treatment, other) initiated less than or equal to 90 days after date of determination of laBCC or mBCC. For example, if patient receives topical treatment followed by vismo followed by surgery, then all 3 treatments must be initiated within the 90 days of the date of determination of disease.

[1] The descriptive statistics are based on the patients with a non-missing result (n). The denominator for 'Unknown'/'Not Available' is the column header (N).

[2] A patient may be counted in more than one category. The percentages may add up to more than 100%.

					Cohort 1: Newly Determined Advanced BCC Patients (Non-BCCNS) Locally Advanced BCC (laBCC)				
		Vismo (N=115)			No Vismo (N=251)				
	All (N=433)	All (N=115)	Vismo Only (N=102)	Surgery Only (N=6)	Other Therapy (N=7)	All (N=251)	Surgery Only (N=198)	Other Therapy (N=53)	No Treatment (N=67)
Age (years)									
n	433	115	102	6	7	251	198	53	67
Mean (SD)	67.2 (14.18)	67.7 (14.97)	67.6 (15.27)	65.2 (10.61)	71.1 (14.80)	66.4 (13.80)	66.0 (13.36)	67.8 (15.40)	69.8 (14.04)
Median	67.0	66.0	66.5	65.0	73.0	66.0	66.0	67.0	69.0
Range	(23, 99)	(34, 99)	(34, 99)	(50, 80)	(53, 91)	(23, 93)	(23, 93)	(38, 93)	(35, 96)
Age Group (years)									
n	433	115	102	6	7	251	198	53	67
18 - <30	1(0.2%)	0	0	0	0	1(0.4%)	1(0.5%)	0	0
30 - <40	10(2.3%)	2(1.7%)	2(2.0%)	0	0	7(2.8%)	6(3.0%)	1(1.9%)	1(1.5%)
40 - <50	38(8.8%)	13(11.3%)	13(12.7%)	0	0	20(8.0%)	12(6.1%)	8(15.1%)	5(7.5%)
50 - <60	83(19.2%)	16(13.9%)	12(11.8%)	2(33.3%)	2(28.6%)	56(22.3%)	50(25.3%)	6(11.3%)	11(16.4%)
60 - <70	118(27.3%)	38(33.0%)	35(34.3%)	2(33.3%)	1(14.3%)	62(24.7%)	50(25.3%)	12(22.6%)	18(26.9%)
70 - <80	83(19.2%)	14(12.2%)	11(10.8%)	1(16.7%)	2(28.6%)	54(21.5%)	41(20.7%)	13(24.5%)	15(22.4%)
80 - <90	78(18.0%)	23(20.0%)	21(20.6%)	1(16.7%)	1(14.3%)	43(17.1%)	35(17.7%)	8(15.1%)	12(17.9%)
>=90	22(5.1%)	9(7.8%)	8(7.8%)	0	1(14.3%)	8(3.2%)	3(1.5%)	5(9.4%)	5(7.5%)

					Cohort 1: Newly Determined Advanced BCC Patients (Non-BCCNS) Metastatic BCC (mBCC)				
		Vismo (N=2)			No Vismo (N=1)				
	All (N=4)	All (N=2)	Vismo Only (N=2)	Surgery Only (N=0)	Other Therapy (N=0)	All (N=1)	Surgery Only (N=1)	Other Therapy (N=0)	No Treatment (N=1)
Age (years)									
n	4	2	2	0	0	1	1	0	1
Mean (SD)	67.8 (14.52)	57.5 (3.54)	57.5 (3.54)			68.0 (-)	68.0 (-)		88.0 (-)
Median	64.0	57.5	57.5			68.0	68.0		88.0

Range	(55, 88)	(55, 60)	(55, 60)			(68, 68)	(68, 68)		(88, 88)
Age Group (years)									
n	4	2	2	0	0	1	1	0	1
18 - <30	0	0	0	0	0	0	0	0	0
30 - <40	0	0	0	0	0	0	0	0	0
40 - <50	0	0	0	0	0	0	0	0	0
50 - <60	1(25.0%)	1(50.0%)	1(50.0%)	0	0	0	0	0	0
60 - <70	2(50.0%)	1(50.0%)	1(50.0%)	0	0	1(100%)	1(100%)	0	0
70 - <80	0	0	0	0	0	0	0	0	0
80 - <90	1(25.0%)	0	0	0	0	0	0	0	1(100%)
>=90	0	0	0	0	0	0	0	0	0

Cohort 1: Newly Determined Advanced BCC Patients (Non-BCCNS) Locally Advanced BCC (laBCC)									
		Vismo (N=115)			No Vismo (N=251)				
	All (N=433)	All (N=115)	Vismo Only (N=102)	Surgery Only (N=6)	Other Therapy (N=7)	All (N=251)	Surgery Only (N=198)	Other Therapy (N=53)	No Treatment (N=67)
Age Group (years)									
n	433	115	102	6	7	251	198	53	67
<65	187(43.2%)	51(44.3%)	46(45.1%)	2(33.3%)	3(42.9%)	111(44.2%)	90(45.5%)	21(39.6%)	25(37.3%)
>=65	246(56.8%)	64(55.7%)	56(54.9%)	4(66.7%)	4(57.1%)	140(55.8%)	108(54.5%)	32(60.4%)	42(62.7%)
Sex									
n	433	115	102	6	7	251	198	53	67
Male	271(62.6%)	78(67.8%)	70(68.6%)	6(100%)	2(28.6%)	145(57.8%)	116(58.6%)	29(54.7%)	48(71.6%)
Female	162(37.4%)	37(32.2%)	32(31.4%)	0	5(71.4%)	106(42.2%)	82(41.4%)	24(45.3%)	19(28.4%)
Female of Childbearing Potential									
n	161	37	32	0	5	105	81	24	19
Yes	15(9.3%)	3(8.1%)	3(9.4%)	0	0	8(7.6%)	5(6.2%)	3(12.5%)	4(21.1%)
No	146(90.7%)	34(91.9%)	29(90.6%)	0	5(100%)	97(92.4%)	76(93.8%)	21(87.5%)	15(78.9%)

Cohort 1: Newly Determined Advanced BCC Patients (Non-BCCNS) Metastatic BCC (mBCC)									
		Vismo (N=2)			No Vismo (N=1)				
	All (N=4)	All (N=2)	Vismo Only (N=2)	Surgery Only (N=0)	Other Therapy (N=0)	All (N=1)	Surgery Only (N=1)	Other Therapy (N=0)	No Treatment (N=1)
Age Group (years)									

n	4	2	2	0	0	1	1	0	1
<65	2(50.0%)	2(100%)	2(100%)	0	0	0	0	0	0
>=65	2(50.0%)	0	0	0	0	1(100%)	1(100%)	0	1(100%)
Sex									
n	4	2	2	0	0	1	1	0	1
Male	3(75.0%)	1(50.0%)	1(50.0%)	0	0	1(100%)	1(100%)	0	1(100%)
Female	1(25.0%)	1(50.0%)	1(50.0%)	0	0	0	0	0	0
Female of Childbearing Potential									
n	1	1	1	0	0	0	0	0	0
Yes	0	0	0	0	0	0	0	0	0
No	1(100%)	1(100%)	1(100%)	0	0	0	0	0	0
					Cohort 1: Newly Determined Advanced BCC Patients (Non-BCCNS) Locally Advanced BCC (laBCC)				
		Vismo (N=115)			No Vismo (N=251)				
	All (N=433)	All (N=115)	Vismo Only (N=102)	Surgery Only (N=6)	Other Therapy (N=7)	All (N=251)	Surgery Only (N=198)	Other Therapy (N=53)	No Treatment (N=67)
If Yes to FCBP, is Female Still Menstruating									
n	15	3	3	0	0	8	5	3	4
Yes	15(100%)	3(100%)	3(100%)	0	0	8(100%)	5(100%)	3(100%)	4(100%)
No	0	0	0	0	0	0	0	0	0
If No to FCBP, Reason									
n	146	34	29	0	5	97	76	21	15
Menopause	101(69.2%)	27(79.4%)	23(79.3%)	0	4(80.0%)	63(64.9%)	48(63.2%)	15(71.4%)	11(73.3%)
Hysterectomy	36(24.7%)	6(17.6%)	5(17.2%)	0	1(20.0%)	29(29.9%)	23(30.3%)	6(28.6%)	1(6.7%)
Tubal Ligation	9(6.2%)	1(2.9%)	1(3.4%)	0	0	5(5.2%)	5(6.6%)	0	3(20.0%)
Other	0	0	0	0	0	0	0	0	0
					Cohort 1: Newly Determined Advanced BCC Patients (Non-BCCNS) Metastatic BCC (mBCC)				
		Vismo (N=2)			No Vismo (N=1)				

	All (N=4)	All (N=2)	Vismo Only (N=2)	Surgery Only (N=0)	Other Therapy (N=0)	All (N=1)	Surgery Only (N=1)	Other Therapy (N=0)	No Treatment (N=1)
If Yes to FCBP, is Female Still Menstruating									
n	0	0	0	0	0	0	0	0	0
Yes	0	0	0	0	0	0	0	0	0
No	0	0	0	0	0	0	0	0	0
If No to FCBP, Reason									
n	1	1	1	0	0	0	0	0	0
Menopause	0	0	0	0	0	0	0	0	0
Hysterectomy	0	0	0	0	0	0	0	0	0
Tubal Ligation	1(100%)	1(100%)	1(100%)	0	0	0	0	0	0
Other	0	0	0	0	0	0	0	0	0

					Cohort 1: Newly Determined Advanced BCC Patients (Non-BCCNS) Locally Advanced BCC (laBCC)				
			Vismo (N=115)		No Vismo (N=251)				
	All (N=433)	All (N=115)	Vismo Only (N=102)	Surgery Only (N=6)	Other Therapy (N=7)	All (N=251)	Surgery Only (N=198)	Other Therapy (N=53)	No Treatment (N=67)
Ethnicity									
n	433	115	102	6	7	251	198	53	67
Not Hispanic or Latino	411(94.9%)	108(93.9%)	95(93.1%)	6(100%)	7(100%)	241(96.0%)	191(96.5%)	50(94.3%)	62(92.5%)
Hispanic or Latino	14(3.2%)	6(5.2%)	6(5.9%)	0	0	4(1.6%)	3(1.5%)	1(1.9%)	4(6.0%)
Not Available	8(1.8%)	1(0.9%)	1(1.0%)	0	0	6(2.4%)	4(2.0%)	2(3.8%)	1(1.5%)
Race [2]									
n	433	115	102	6	7	251	198	53	67
White	431(99.5%)	114(99.1%)	101(99.0%)	6(100%)	7(100%)	250(99.6%)	197(99.5%)	53(100%)	67(100%)
Asian	0	0	0	0	0	0	0	0	0

Black or African American	0	0	0	0	0	0	0	0	0
Native Hawaiian or Pacific Islander	0	0	0	0	0	0	0	0	0
American Indian or Alaskan Native	2(0.5%)	0	0	0	0	1(0.4%)	1(0.5%)	0	1(1.5%)
Other	2(0.5%)	1(0.9%)	1(1.0%)	0	0	1(0.4%)	1(0.5%)	0	0
					Cohort 1: Newly Determined Advanced BCC Patients (Non-BCCNS) Metastatic BCC (mBCC)				
			Vismo (N=2)			No Vismo (N=1)			
	All (N=4)	All (N=2)	Vismo Only (N=2)	Surgery Only (N=0)	Other Therapy (N=0)	All (N=1)	Surgery Only (N=1)	Other Therapy (N=0)	No Treatment (N=1)
Ethnicity									
n	4	2	2	0	0	1	1	0	1
Not Hispanic or Latino	4(100%)	2(100%)	2(100%)	0	0	1(100%)	1(100%)	0	1(100%)
Hispanic or Latino	0	0	0	0	0	0	0	0	0
Not Available	0	0	0	0	0	0	0	0	0
Race [2]									
n	4	2	2	0	0	1	1	0	1
White	4(100%)	2(100%)	2(100%)	0	0	1(100%)	1(100%)	0	1(100%)
Asian	0	0	0	0	0	0	0	0	0
Black or African American	0	0	0	0	0	0	0	0	0
Native Hawaiian or Pacific Islander	0	0	0	0	0	0	0	0	0
American Indian or Alaskan Native	0	0	0	0	0	0	0	0	0
Other	0	0	0	0	0	0	0	0	0
					Cohort 1: Newly Determined Advanced BCC Patients (Non-BCCNS) Locally Advanced BCC (laBCC)				
			Vismo (N=115)			No Vismo (N=251)			

	All (N=433)	All (N=115)	Vismo Only (N=102)	Surgery Only (N=6)	Other Therapy (N=7)	All (N=251)	Surgery Only (N=198)	Other Therapy (N=53)	No Treatment (N=67)
Height (inches)									
n	345	95	86	2	7	196	155	41	54
Mean (SD)	67.75 (4.334)	67.85 (4.594)	67.85 (4.746)	71.74 (1.787)	66.71 (2.138)	67.58 (4.270)	67.75 (4.410)	66.95 (3.671)	68.19 (4.137)
Median	68.00	68.00	68.06	71.74	67.00	68.00	68.00	67.00	68.95
Range	(55.9, 78.0)	(55.9, 78.0)	(55.9, 78.0)	(70.5, 73.0)	(64.0, 70.0)	(56.0, 78.0)	(56.0, 78.0)	(59.0, 73.0)	(58.0, 76.0)
Height (m)									
n	345	95	86	2	7	196	155	41	54
Mean (SD)	1.721 (0.1101)	1.723 (0.1167)	1.723 (0.1205)	1.822 (0.0454)	1.695 (0.0543)	1.717 (0.1085)	1.721 (0.1120)	1.700 (0.0932)	1.732 (0.1051)
Median	1.727	1.727	1.729	1.822	1.702	1.727	1.727	1.702	1.751
Range	(1.42, 1.98)	(1.42, 1.98)	(1.42, 1.98)	(1.79, 1.85)	(1.63, 1.78)	(1.42, 1.98)	(1.42, 1.98)	(1.50, 1.85)	(1.47, 1.93)
Weight at Study Enrollment (lbs)									
n	349	96	86	3	7	204	163	41	49
Mean (SD)	181.78 (46.818)	181.14 (48.885)	179.83 (50.485)	214.74 (27.333)	182.86 (29.818)	182.46 (46.232)	183.64 (47.081)	177.77 (42.917)	180.24 (46.013)
Median	179.00	177.50	174.00	220.46	180.00	178.50	179.40	173.00	180.00
Range	(66.9, 370.0)	(74.0, 339.0)	(74.0, 339.0)	(185.0, 238.8)	(150.0, 230.0)	(93.0, 370.0)	(93.0, 370.0)	(112.0, 275.0)	(66.9, 340.0)

					Cohort 1: Newly Determined Advanced BCC Patients (Non-BCCNS) Metastatic BCC (mBCC)				
		Vismo (N=2)			No Vismo (N=1)				
	All (N=4)	All (N=2)	Vismo Only (N=2)	Surgery Only (N=0)	Other Therapy (N=0)	All (N=1)	Surgery Only (N=1)	Other Therapy (N=0)	No Treatment (N=1)
Height (inches)									
n	4	2	2	0	0	1	1	0	1
Mean (SD)	65.45 (4.142)	65.16 (2.505)	65.16 (2.505)			70.47 (-)	70.47 (-)		61.00 (-)
Median	65.16	65.16	65.16			70.47	70.47		61.00
Range	(61.0, 70.5)	(63.4, 66.9)	(63.4, 66.9)			(70.5, 70.5)	(70.5, 70.5)		(61.0, 61.0)
Height (m)									
n	4	2	2	0	0	1	1	0	1
Mean (SD)	1.662 (0.1052)	1.655 (0.0636)	1.655 (0.0636)			1.790 (-)	1.790 (-)		1.549 (-)
Median	1.655	1.655	1.655			1.790	1.790		1.549

Range	(1.55, 1.79)	(1.61, 1.70)	(1.61, 1.70)			(1.79, 1.79)	(1.79, 1.79)		(1.55, 1.55)
Weight at Study Enrollment (lbs)									
n	4	2	2	0	0	1	1	0	1
Mean (SD)	144.23 (35.895)	138.01 (20.889)	138.01 (20.889)			190.92 (-)	190.92 (-)		110.00 (-)
Median	138.01	138.01	138.01			190.92	190.92		110.00
Range	(110.0, 190.9)	(123.2, 152.8)	(123.2, 152.8)			(190.9, 190.9)	(190.9, 190.9)		(110.0, 110.0)

					Cohort 1: Newly Determined Advanced BCC Patients (Non-BCCNS) Locally Advanced BCC (laBCC)				
		Vismo (N=115)			No Vismo (N=251)				
	All (N=433)	All (N=115)	Vismo Only (N=102)	Surgery Only (N=6)	Other Therapy (N=7)	All (N=251)	Surgery Only (N=198)	Other Therapy (N=53)	No Treatment (N=67)
Weight at Study Enrollment (kg)									
n	349	96	86	3	7	204	163	41	49
Mean (SD)	82.46 (21.236)	82.16 (22.174)	81.57 (22.900)	97.40 (12.398)	82.94 (13.525)	82.76 (20.971)	83.30 (21.356)	80.64 (19.467)	81.75 (20.871)
Median	81.19	80.51	78.93	100.00	81.65	80.97	81.37	78.47	81.65
Range	(30.3, 167.8)	(33.6, 153.8)	(33.6, 153.8)	(83.9, 108.3)	(68.0, 104.3)	(42.2, 167.8)	(42.2, 167.8)	(50.8, 124.7)	(30.3, 154.2)
BMI at Study Enrollment (kg/m^2) [1]									
n	331	91	82	2	7	192	152	40	48
Mean (SD)	27.78 (6.213)	27.32 (5.868)	27.18 (6.041)	27.81 (4.810)	28.84 (4.114)	28.11 (6.130)	28.28 (6.414)	27.47 (4.920)	27.35 (7.165)
Median	26.58	26.51	26.50	27.81	29.95	26.92	26.92	26.91	26.15
Range	(10.5, 58.2)	(16.1, 46.5)	(16.1, 46.5)	(24.4, 31.2)	(24.3, 35.0)	(16.1, 58.2)	(16.1, 58.2)	(17.2, 39.5)	(10.5, 51.7)

					Cohort 1: Newly Determined Advanced BCC Patients (Non-BCCNS) Metastatic BCC (mBCC)				
		Vismo (N=2)			No Vismo (N=1)				
	All (N=4)	All (N=2)	Vismo Only (N=2)	Surgery Only (N=0)	Other Therapy (N=0)	All (N=1)	Surgery Only (N=1)	Other Therapy (N=0)	No Treatment (N=1)
Weight at Study Enrollment (kg)									

n	4	2	2	0	0	1	1	0	1
Mean (SD)	65.42 (16.282)	62.60 (9.475)	62.60 (9.475)			86.60 (-)	86.60 (-)		49.90 (-)
Median	62.60	62.60	62.60			86.60	86.60		49.90
Range	(49.9, 86.6)	(55.9, 69.3)	(55.9, 69.3)			(86.6, 86.6)	(86.6, 86.6)		(49.9, 49.9)
BMI at Study Enrollment (kg/m^2) [1]									
n	4	2	2	0	0	1	1	0	1
Mean (SD)	23.34 (2.810)	22.77 (1.707)	22.77 (1.707)			27.03 (-)	27.03 (-)		20.78 (-)
Median	22.77	22.77	22.77			27.03	27.03		20.78
Range	(20.8, 27.0)	(21.6, 24.0)	(21.6, 24.0)			(27.0, 27.0)	(27.0, 27.0)		(20.8, 20.8)

	Cohort 1: Newly Determined Advanced BCC Patients (Non-BCCNS) Locally Advanced BCC (laBCC)								
		Vismo (N=115)			No Vismo (N=251)				
	All (N=433)	All (N=115)	Vismo Only (N=102)	Surgery Only (N=6)	Other Therapy (N=7)	All (N=251)	Surgery Only (N=198)	Other Therapy (N=53)	No Treatment (N=67)
Weight at Start of Treatment (lbs) [1]									
n	247	63	60	2	1	143	117	26	41
Mean (SD)	184.49 (47.362)	189.75 (47.984)	188.02 (48.476)	229.61 (12.939)	214.00 (-)	182.77 (48.204)	182.98 (48.911)	181.80 (45.789)	182.40 (43.804)
Median	180.00	184.00	180.39	229.61	214.00	178.00	178.00	176.50	182.00
Range	(93.0, 371.0)	(120.0, 339.0)	(120.0, 339.0)	(220.5, 238.8)	(214.0, 214.0)	(93.0, 371.0)	(93.0, 371.0)	(114.0, 275.0)	(118.6, 340.0)
Unknown	40(9.2%)	11(9.6%)	8(7.8%)	2(33.3%)	1(14.3%)	23(9.2%)	20(10.1%)	3(5.7%)	6(9.0%)
Not Available	140(32.3%)	40(34.8%)	33(32.4%)	2(33.3%)	5(71.4%)	80(31.9%)	58(29.3%)	22(41.5%)	20(29.9%)
Weight at Start of Treatment (kg) [1]									
n	247	63	60	2	1	143	117	26	41
Mean (SD)	83.68 (21.483)	86.07 (21.765)	85.29 (21.988)	104.15 (5.869)	97.07 (-)	82.90 (21.865)	83.00 (22.186)	82.46 (20.769)	82.73 (19.869)
Median	81.65	83.46	81.82	104.15	97.07	80.74	80.74	80.06	82.55
Range	(42.2, 168.3)	(54.4, 153.8)	(54.4, 153.8)	(100.0, 108.3)	(97.1, 97.1)	(42.2, 168.3)	(42.2, 168.3)	(51.7, 124.7)	(53.8, 154.2)
Unknown	40(9.2%)	11(9.6%)	8(7.8%)	2(33.3%)	1(14.3%)	23(9.2%)	20(10.1%)	3(5.7%)	6(9.0%)
Not Available	140(32.3%)	40(34.8%)	33(32.4%)	2(33.3%)	5(71.4%)	80(31.9%)	58(29.3%)	22(41.5%)	20(29.9%)

					Cohort 1: Newly Determined Advanced BCC Patients (Non-BCCNS) Metastatic BCC (mBCC)				
			Vismo (N=2)			No Vismo (N=1)			
	All (N=4)	All (N=2)	Vismo Only (N=2)	Surgery Only (N=0)	Other Therapy (N=0)	All (N=1)	Surgery Only (N=1)	Other Therapy (N=0)	No Treatment (N=1)
Weight at Start of Treatment (lbs) [1]									
n	2	1	1	0	0	0	0	0	1
Mean (SD)	116.62 (9.361)	123.24 (-)	123.24 (-)						110.00 (-)
Median	116.62	123.24	123.24						110.00
Range	(110.0, 123.2)	(123.2, 123.2)	(123.2, 123.2)						(110.0, 110.0)
Unknown	0	0	0	0	0	0	0	0	0
Not Available	2(50.0%)	1(50.0%)	1(50.0%)	0	0	1(100%)	1(100%)	0	0
Weight at Start of Treatment (kg) [1]									
n	2	1	1	0	0	0	0	0	1
Mean (SD)	52.90 (4.246)	55.90 (-)	55.90 (-)						49.90 (-)
Median	52.90	55.90	55.90						49.90
Range	(49.9, 55.9)	(55.9, 55.9)	(55.9, 55.9)						(49.9, 49.9)
Unknown	0	0	0	0	0	0	0	0	0
Not Available	2(50.0%)	1(50.0%)	1(50.0%)	0	0	1(100%)	1(100%)	0	0
					Cohort 1: Newly Determined Advanced BCC Patients (Non-BCCNS) Locally Advanced BCC (laBCC)				
			Vismo (N=115)			No Vismo (N=251)			
	All (N=433)	All (N=115)	Vismo Only (N=102)	Surgery Only (N=6)	Other Therapy (N=7)	All (N=251)	Surgery Only (N=198)	Other Therapy (N=53)	No Treatment (N=67)
BMI at Start of Treatment (kg/m^2) [1]									
n	230	60	58	1	1	132	107	25	38
Mean (SD)	27.96 (6.085)	28.06 (5.489)	27.96 (5.557)	31.21 (-)	30.71 (-)	28.07 (6.192)	28.09 (6.431)	27.96 (5.157)	27.42 (6.712)
Median	26.84	27.28	27.19	31.21	30.71	26.92	26.83	27.97	25.79
Range	(16.1, 58.2)	(18.9, 42.4)	(18.9, 42.4)	(31.2, 31.2)	(30.7, 30.7)	(16.1, 58.2)	(16.1, 58.2)	(17.5, 39.5)	(19.2, 51.7)

Highest Level of Education Attained									
n	433	115	102	6	7	251	198	53	67
High School Graduate or Less	148(34.2%)	46(40.0%)	42(41.2%)	0	4(57.1%)	80(31.9%)	60(30.3%)	20(37.7%)	22(32.8%)
Some College or Associates Degree	98(22.6%)	26(22.6%)	21(20.6%)	3(50.0%)	2(28.6%)	58(23.1%)	49(24.7%)	9(17.0%)	14(20.9%)
College Graduate or Above	122(28.2%)	24(20.9%)	21(20.6%)	3(50.0%)	0	81(32.3%)	63(31.8%)	18(34.0%)	17(25.4%)
Unknown	65(15.0%)	19(16.5%)	18(17.6%)	0	1(14.3%)	32(12.7%)	26(13.1%)	6(11.3%)	14(20.9%)
					Cohort 1: Newly Determined Advanced BCC Patients (Non-BCCNS) Metastatic BCC (mBCC)				
			Vismo (N=2)			No Vismo (N=1)			
	All (N=4)	All (N=2)	Vismo Only (N=2)	Surgery Only (N=0)	Other Therapy (N=0)	All (N=1)	Surgery Only (N=1)	Other Therapy (N=0)	No Treatment (N=1)
BMI at Start of Treatment (kg/m^2) [1]									
n	2	1	1	0	0	0	0	0	1
Mean (SD)	21.17 (0.553)	21.57 (-)	21.57 (-)						20.78 (-)
Median	21.17	21.57	21.57						20.78
Range	(20.8, 21.6)	(21.6, 21.6)	(21.6, 21.6)						(20.8, 20.8)
Highest Level of Education Attained									
n	4	2	2	0	0	1	1	0	1
High School Graduate or Less	1(25.0%)	0	0	0	0	0	0	0	1(100%)
Some College or Associates Degree	1(25.0%)	1(50.0%)	1(50.0%)	0	0	0	0	0	0
College Graduate or Above	2(50.0%)	1(50.0%)	1(50.0%)	0	0	1(100%)	1(100%)	0	0
Unknown	0	0	0	0	0	0	0	0	0
					Cohort 1: Newly Determined Advanced BCC Patients (Non-BCCNS) Locally Advanced BCC (laBCC)				
			Vismo (N=115)			No Vismo (N=251)			

	All (N=433)	All (N=115)	Vismo Only (N=102)	Surgery Only (N=6)	Other Therapy (N=7)	All (N=251)	Surgery Only (N=198)	Other Therapy (N=53)	No Treatment (N=67)
Marital Status									
n	433	115	102	6	7	251	198	53	67
Never Married	47(10.9%)	17(14.8%)	16(15.7%)	1(16.7%)	0	26(10.4%)	20(10.1%)	6(11.3%)	4(6.0%)
Married	234(54.0%)	49(42.6%)	43(42.2%)	4(66.7%)	2(28.6%)	149(59.4%)	120(60.6%)	29(54.7%)	36(53.7%)
Widowed	63(14.5%)	20(17.4%)	18(17.6%)	0	2(28.6%)	36(14.3%)	26(13.1%)	10(18.9%)	7(10.4%)
Divorced	63(14.5%)	18(15.7%)	15(14.7%)	1(16.7%)	2(28.6%)	33(13.1%)	27(13.6%)	6(11.3%)	12(17.9%)
Separated	7(1.6%)	2(1.7%)	2(2.0%)	0	0	5(2.0%)	3(1.5%)	2(3.8%)	0
Unknown	19(4.4%)	9(7.8%)	8(7.8%)	0	1(14.3%)	2(0.8%)	2(1.0%)	0	8(11.9%)
Does the Patient Live Alone?									
n	433	115	102	6	7	251	198	53	67
Yes	105(24.2%)	29(25.2%)	26(25.5%)	2(33.3%)	1(14.3%)	58(23.1%)	46(23.2%)	12(22.6%)	18(26.9%)
No	306(70.7%)	78(67.8%)	68(66.7%)	4(66.7%)	6(85.7%)	186(74.1%)	146(73.7%)	40(75.5%)	42(62.7%)
Unknown	22(5.1%)	8(7.0%)	8(7.8%)	0	0	7(2.8%)	6(3.0%)	1(1.9%)	7(10.4%)

					Cohort 1: Newly Determined Advanced BCC Patients (Non-BCCNS) Metastatic BCC (mBCC)				
		Vismo (N=2)			No Vismo (N=1)				
	All (N=4)	All (N=2)	Vismo Only (N=2)	Surgery Only (N=0)	Other Therapy (N=0)	All (N=1)	Surgery Only (N=1)	Other Therapy (N=0)	No Treatment (N=1)
Marital Status									
n	4	2	2	0	0	1	1	0	1
Never Married	0	0	0	0	0	0	0	0	0
Married	3(75.0%)	2(100%)	2(100%)	0	0	1(100%)	1(100%)	0	0
Widowed	1(25.0%)	0	0	0	0	0	0	0	1(100%)
Divorced	0	0	0	0	0	0	0	0	0
Separated	0	0	0	0	0	0	0	0	0
Unknown	0	0	0	0	0	0	0	0	0
Does the Patient Live Alone?									
n	4	2	2	0	0	1	1	0	1
Yes	0	0	0	0	0	0	0	0	0
No	4(100%)	2(100%)	2(100%)	0	0	1(100%)	1(100%)	0	1(100%)
Unknown	0	0	0	0	0	0	0	0	0

		Cohort 1: Newly Determined Advanced BCC Patients (Non-BCCNS) Locally Advanced BCC (laBCC)							
		Vismo (N=115)				No Vismo (N=251)			
	All (N=433)	All (N=115)	Vismo Only (N=102)	Surgery Only (N=6)	Other Therapy (N=7)	All (N=251)	Surgery Only (N=198)	Other Therapy (N=53)	No Treatment (N=67)
Employment Status									
n	431	115	102	6	7	251	198	53	65
Employed	134(31.1%)	36(31.3%)	33(32.4%)	2(33.3%)	1(14.3%)	80(31.9%)	66(33.3%)	14(26.4%)	18(27.7%)
Unemployed	68(15.8%)	19(16.5%)	16(15.7%)	0	3(42.9%)	38(15.1%)	29(14.6%)	9(17.0%)	11(16.9%)
Retired	229(53.1%)	60(52.2%)	53(52.0%)	4(66.7%)	3(42.9%)	133(53.0%)	103(52.0%)	30(56.6%)	36(55.4%)
Tobacco Use									
n	433	115	102	6	7	251	198	53	67
Current	60(13.9%)	17(14.8%)	16(15.7%)	1(16.7%)	0	37(14.7%)	27(13.6%)	10(18.9%)	6(9.0%)
Former	139(32.1%)	46(40.0%)	40(39.2%)	4(66.7%)	2(28.6%)	68(27.1%)	58(29.3%)	10(18.9%)	25(37.3%)
Never	227(52.4%)	49(42.6%)	43(42.2%)	1(16.7%)	5(71.4%)	144(57.4%)	111(56.1%)	33(62.3%)	34(50.7%)
Unknown	7(1.6%)	3(2.6%)	3(2.9%)	0	0	2(0.8%)	2(1.0%)	0	2(3.0%)

		Cohort 1: Newly Determined Advanced BCC Patients (Non-BCCNS) Metastatic BCC (mBCC)							
		Vismo (N=2)				No Vismo (N=1)			
	All (N=4)	All (N=2)	Vismo Only (N=2)	Surgery Only (N=0)	Other Therapy (N=0)	All (N=1)	Surgery Only (N=1)	Other Therapy (N=0)	No Treatment (N=1)
Employment Status									
n	4	2	2	0	0	1	1	0	1
Employed	3(75.0%)	2(100%)	2(100%)	0	0	1(100%)	1(100%)	0	0
Unemployed	0	0	0	0	0	0	0	0	0
Retired	1(25.0%)	0	0	0	0	0	0	0	1(100%)
Tobacco Use									
n	4	2	2	0	0	1	1	0	1
Current	1(25.0%)	1(50.0%)	1(50.0%)	0	0	0	0	0	0
Former	2(50.0%)	1(50.0%)	1(50.0%)	0	0	0	0	0	1(100%)
Never	1(25.0%)	0	0	0	0	1(100%)	1(100%)	0	0
Unknown	0	0	0	0	0	0	0	0	0

		Cohort 1: Newly Determined Advanced BCC Patients (Non-BCCNS) Locally Advanced BCC (laBCC)							
		Vismo (N=115)				No Vismo (N=251)			
	All (N=433)	All (N=115)	Vismo Only (N=102)	Surgery Only (N=6)	Other Therapy (N=7)	All (N=251)	Surgery Only (N=198)	Other Therapy (N=53)	No Treatment (N=67)

Alcohol Use									
n	433	115	102	6	7	251	198	53	67
Current	219(50.6%)	50(43.5%)	46(45.1%)	4(66.7%)	0	134(53.4%)	109(55.1%)	25(47.2%)	35(52.2%)
Former	63(14.5%)	16(13.9%)	16(15.7%)	0	0	38(15.1%)	32(16.2%)	6(11.3%)	9(13.4%)
Never	133(30.7%)	40(34.8%)	32(31.4%)	2(33.3%)	6(85.7%)	73(29.1%)	53(26.8%)	20(37.7%)	20(29.9%)
Unknown	18(4.2%)	9(7.8%)	8(7.8%)	0	1(14.3%)	6(2.4%)	4(2.0%)	2(3.8%)	3(4.5%)
If Current, Average Drinks/ Week									
n	216	49	45	4	0	132	107	25	35
>14 Drinks/ Week	18(8.3%)	5(10.2%)	4(8.9%)	1(25.0%)	0	12(9.1%)	9(8.4%)	3(12.0%)	1(2.9%)
7-14 Drinks/ Week	30(13.9%)	4(8.2%)	4(8.9%)	0	0	21(15.9%)	18(16.8%)	3(12.0%)	5(14.3%)
<7 Drinks/ Week	168(77.8%)	40(81.6%)	37(82.2%)	3(75.0%)	0	99(75.0%)	80(74.8%)	19(76.0%)	29(82.9%)
Unknown	0	0	0	0	0	0	0	0	0
					Cohort 1: Newly Determined Advanced BCC Patients (Non-BCCNS) Metastatic BCC (mBCC)				
			Vismo (N=2)			No Vismo (N=1)			
	All (N=4)	All (N=2)	Vismo Only (N=2)	Surgery Only (N=0)	Other Therapy (N=0)	All (N=1)	Surgery Only (N=1)	Other Therapy (N=0)	No Treatment (N=1)
Alcohol Use									
n	4	2	2	0	0	1	1	0	1
Current	3(75.0%)	2(100%)	2(100%)	0	0	1(100%)	1(100%)	0	0
Former	1(25.0%)	0	0	0	0	0	0	0	1(100%)
Never	0	0	0	0	0	0	0	0	0
Unknown	0	0	0	0	0	0	0	0	0
If Current, Average Drinks/ Week									
n	3	2	2	0	0	1	1	0	0
>14 Drinks/ Week	0	0	0	0	0	0	0	0	0

7-14 Drinks/ Week	0	0	0	0	0	0	0	0	0
<7 Drinks/ Week	3(100%)	2(100%)	2(100%)	0	0	1(100%)	1(100%)	0	0
Unknown	0	0	0	0	0	0	0	0	0
					Cohort 1: Newly Determined Advanced BCC Patients (Non-BCCNS) Locally Advanced BCC (laBCC)				
			Vismo (N=115)			No Vismo (N=251)			
	All (N=433)	All (N=115)	Vismo Only (N=102)	Surgery Only (N=6)	Other Therapy (N=7)	All (N=251)	Surgery Only (N=198)	Other Therapy (N=53)	No Treatment (N=67)
Sunscreen Use									
n	433	115	102	6	7	251	198	53	67
Regularly	133(30.7%)	28(24.3%)	25(24.5%)	3(50.0%)	0	84(33.5%)	67(33.8%)	17(32.1%)	21(31.3%)
Occasionally	151(34.9%)	35(30.4%)	33(32.4%)	1(16.7%)	1(14.3%)	90(35.9%)	75(37.9%)	15(28.3%)	26(38.8%)
Never	120(27.7%)	39(33.9%)	33(32.4%)	1(16.7%)	5(71.4%)	67(26.7%)	49(24.7%)	18(34.0%)	14(20.9%)
Unknown	29(6.7%)	13(11.3%)	11(10.8%)	1(16.7%)	1(14.3%)	10(4.0%)	7(3.5%)	3(5.7%)	6(9.0%)
Age at First Sunscreen Use									
n	290	63	58	4	1	178	145	33	49
<18	17(5.9%)	3(4.8%)	3(5.2%)	0	0	11(6.2%)	8(5.5%)	3(9.1%)	3(6.1%)
18 - 39	83(28.6%)	16(25.4%)	16(27.6%)	0	0	58(32.6%)	45(31.0%)	13(39.4%)	9(18.4%)
40 - 64	102(35.2%)	24(38.1%)	21(36.2%)	2(50.0%)	1(100%)	60(33.7%)	49(33.8%)	11(33.3%)	18(36.7%)
>=65	31(10.7%)	4(6.3%)	4(6.9%)	0	0	18(10.1%)	17(11.7%)	1(3.0%)	9(18.4%)
Unknown	57(19.7%)	16(25.4%)	14(24.1%)	2(50.0%)	0	31(17.4%)	26(17.9%)	5(15.2%)	10(20.4%)
					Cohort 1: Newly Determined Advanced BCC Patients (Non-BCCNS) Metastatic BCC (mBCC)				
			Vismo (N=2)			No Vismo (N=1)			
	All (N=4)	All (N=2)	Vismo Only (N=2)	Surgery Only (N=0)	Other Therapy (N=0)	All (N=1)	Surgery Only (N=1)	Other Therapy (N=0)	No Treatment (N=1)
Sunscreen Use									
n	4	2	2	0	0	1	1	0	1
Regularly	1(25.0%)	1(50.0%)	1(50.0%)	0	0	0	0	0	0
Occasionally	1(25.0%)	1(50.0%)	1(50.0%)	0	0	0	0	0	0
Never	1(25.0%)	0	0	0	0	0	0	0	1(100%)
Unknown	1(25.0%)	0	0	0	0	1(100%)	1(100%)	0	0

Age at First Sunscreen Use									
n	2	2	2	0	0	0	0	0	0
<18	0	0	0	0	0	0	0	0	0
18 - 39	1(50.0%)	1(50.0%)	1(50.0%)	0	0	0	0	0	0
40 - 64	0	0	0	0	0	0	0	0	0
>=65	0	0	0	0	0	0	0	0	0
Unknown	1(50.0%)	1(50.0%)	1(50.0%)	0	0	0	0	0	0

					Cohort 1: Newly Determined Advanced BCC Patients (Non-BCCNS) Locally Advanced BCC (laBCC)				
			Vismo (N=115)		No Vismo (N=251)				
	All (N=433)	All (N=115)	Vismo Only (N=102)	Surgery Only (N=6)	Other Therapy (N=7)	All (N=251)	Surgery Only (N=198)	Other Therapy (N=53)	No Treatment (N=67)
Fitzpatrick Skin Type									
n	427	113	100	6	7	250	198	52	64
I - Highly Sun Sensitive	86(20.1%)	28(24.8%)	25(25.0%)	0	3(42.9%)	46(18.4%)	35(17.7%)	11(21.2%)	12(18.8%)
II - Very Sun Sensitive	184(43.1%)	47(41.6%)	43(43.0%)	1(16.7%)	3(42.9%)	106(42.4%)	81(40.9%)	25(48.1%)	31(48.4%)
III - Sun Sensitive	116(27.2%)	32(28.3%)	26(26.0%)	5(83.3%)	1(14.3%)	68(27.2%)	57(28.8%)	11(21.2%)	16(25.0%)
IV - Minimally Sun Sensitive	32(7.5%)	4(3.5%)	4(4.0%)	0	0	25(10.0%)	20(10.1%)	5(9.6%)	3(4.7%)
V - Sun Insensitive, Rarely Burns	9(2.1%)	2(1.8%)	2(2.0%)	0	0	5(2.0%)	5(2.5%)	0	2(3.1%)
VI - Sun Insensitive, Never Burns	0	0	0	0	0	0	0	0	0

					Cohort 1: Newly Determined Advanced BCC Patients (Non-BCCNS) Metastatic BCC (mBCC)				
			Vismo (N=2)		No Vismo (N=1)				
	All (N=4)	All (N=2)	Vismo Only (N=2)	Surgery Only (N=0)	Other Therapy (N=0)	All (N=1)	Surgery Only (N=1)	Other Therapy (N=0)	No Treatment (N=1)
Fitzpatrick Skin Type									

n	3	1	1	0	0	1	1	0	1
I - Highly Sun Sensitive	1(33.3%)	0	0	0	0	1(100%)	1(100%)	0	0
II - Very Sun Sensitive	1(33.3%)	0	0	0	0	0	0	0	1(100%)
III - Sun Sensitive	1(33.3%)	1(100%)	1(100%)	0	0	0	0	0	0
IV - Minimally Sun Sensitive	0	0	0	0	0	0	0	0	0
V - Sun Insensitive, Rarely Burns	0	0	0	0	0	0	0	0	0
VI - Sun Insensitive, Never Burns	0	0	0	0	0	0	0	0	0

Note: “Vismo” group is defined as initiating vismodegib less than or equal to 90 days after the date of determination of locally advanced BCC (laBCC) or metastatic BCC (mBCC); “No Vismo” group is defined as not initiating vismodegib (but other BCC treatment) less than or equal to 90 days after the date of determination of laBCC or mBCC; “No Treatment” group is defined as not receiving any BCC treatment less than or equal to 90 days after the date of determination of laBCC or mBCC.

For patients who didn’t participate in previous GNE vismo studies (cohort 1 and part of cohort 3 patients), “vismo” group is defined as receiving vismo within less than or equal to 90 days on or after the date of determination of locally advanced BCC or multiple non-advanced BCCs (cohort 3 only) or metastatic BCC. Otherwise, the patient is included in “No vismo” group.

“Vismo Only” and “Surgery Only” are defined as having only 1 of these treatments less than or equal to 90 days after the date of determination of laBCC or mBCC; “Other Therapy” includes individual or combination treatments (e.g., surgery, PDT, ED&C, topical treatment, cryosurgery, systemic treatment, other) initiated less than or equal to 90 days after date of determination of laBCC or mBCC. For example, if patient receives topical treatment followed by vismo followed by surgery, then all 3 treatments must be initiated within the 90 days of the date of determination of disease.

[1] Denominator is number of patients who have private insurance or Medicare or Medicaid. A patient may be counted in more than one category. The percentages may add up to more than 100%.

[2] Denominator is number of patients who have Medicare. A patient may be counted in more than one category. The percentages may add up to more than 100%.

[3] Denominator is number of patients who have private insurance.

					Cohort 1: Newly Determined Advanced BCC Patients (Non-BCCNS) Locally Advanced BCC (laBCC)			
			Vismo (N=115)			No Vismo (N=251)		
	All (N=433)	All (N=115)	Vismo Only (N=102)	Surgery Only (N=6)	Other Therapy (N=7)	All (N=251)	Surgery Only (N=198)	Other Therapy (N=53)	No Treatment (N=67)
Does the Patient Have Private Insurance/ Medicare/ Medicaid?									
n	433	115	102	6	7	251	198	53	67
Yes	402(92.8%)	102(88.7%)	90(88.2%)	5(83.3%)	7(100%)	236(94.0%)	184(92.9%)	52(98.1%)	64(95.5%)
No	31(7.2%)	13(11.3%)	12(11.8%)	1(16.7%)	0	15(6.0%)	14(7.1%)	1(1.9%)	3(4.5%)
If Yes, [1]									
Medicare	219(54.5%)	58(56.9%)	51(56.7%)	2(40.0%)	5(71.4%)	122(51.7%)	94(51.1%)	28(53.8%)	39(60.9%)
Medicaid	47(11.7%)	15(14.7%)	10(11.1%)	0	5(71.4%)	28(11.9%)	20(10.9%)	8(15.4%)	4(6.3%)
Private Insurance	269(66.9%)	61(59.8%)	57(63.3%)	3(60.0%)	1(14.3%)	169(71.6%)	133(72.3%)	36(69.2%)	39(60.9%)
If Medicare, [2]									

Original Medicare Plan (Part A and B)	195(89.0%)	51(87.9%)	44(86.3%)	2(100%)	5(100%)	111(91.0%)	87(92.6%)	24(85.7%)	33(84.6%)
Medicare Advantage (Part C)	13(5.9%)	3(5.2%)	3(5.9%)	0	0	4(3.3%)	3(3.2%)	1(3.6%)	6(15.4%)
Medicare Supplemental Insurance or “Medigap”	27(12.3%)	8(13.8%)	8(15.7%)	0	0	17(13.9%)	13(13.8%)	4(14.3%)	2(5.1%)
Medicare Prescription Drug Coverage (Part D)	30(13.7%)	12(20.7%)	11(21.6%)	1(50.0%)	0	10(8.2%)	9(9.6%)	1(3.6%)	8(20.5%)
					Cohort 1: Newly Determined Advanced BCC Patients (Non-BCCNS) Metastatic BCC (mBCC)				
			Vismo (N=2)		No Vismo (N=1)				
	All (N=4)	All (N=2)	Vismo Only (N=2)	Surgery Only (N=0)	Other Therapy (N=0)	All (N=1)	Surgery Only (N=1)	Other Therapy (N=0)	No Treatment (N=1)
Does the Patient Have Private Insurance/ Medicare/ Medicaid?									
n	4	2	2	0	0	1	1	0	1
Yes	4(100%)	2(100%)	2(100%)	0	0	1(100%)	1(100%)	0	1(100%)
No	0	0	0	0	0	0	0	0	0
If Yes, [1]									

Medicare	2(50.0%)	0	0	0	0	1(100%)	1(100%)	0	1(100%)
Medicaid	1(25.0%)	1(50.0%)	1(50.0%)	0	0	0	0	0	0
Private Insurance	3(75.0%)	2(100%)	2(100%)	0	0	0	0	0	1(100%)
If Medicare, [2]									
Original Medicare Plan (Part A and B)	2(100%)	0	0	0	0	1(100%)	1(100%)	0	1(100%)
Medicare Advantage (Part C)	0	0	0	0	0	0	0	0	0
Medicare Supplemental Insurance or "Medigap"	0	0	0	0	0	0	0	0	0
Medicare Prescription Drug Coverage (Part D)	0	0	0	0	0	0	0	0	0
					Cohort 1: Newly Determined Advanced BCC Patients (Non-BCCNS) Locally Advanced BCC (laBCC)				
			Vismo (N=115)			No Vismo (N=251)			
	All (N=433)	All (N=115)	Vismo Only (N=102)	Surgery Only (N=6)	Other Therapy (N=7)	All (N=251)	Surgery Only (N=198)	Other Therapy (N=53)	No Treatment (N=67)

If Private Insurance, [3]									
HMO (Health Maintenance Organization)	65(24.2%)	20(32.8%)	18(31.6%)	2(66.7%)	0	31(18.3%)	20(15.0%)	11(30.6%)	14(35.9%)
POS (Point of Service)	18(6.7%)	3(4.9%)	3(5.3%)	0	0	12(7.1%)	12(9.0%)	0	3(7.7%)
PPO (Preferred Provider Organization)	128(47.6%)	18(29.5%)	16(28.1%)	1(33.3%)	1(100%)	97(57.4%)	78(58.6%)	19(52.8%)	13(33.3%)
FFS (Fee for Service) or Indemnity	2(0.7%)	1(1.6%)	1(1.8%)	0	0	1(0.6%)	1(0.8%)	0	0
Unknown	20(7.4%)	6(9.8%)	6(10.5%)	0	0	9(5.3%)	6(4.5%)	3(8.3%)	5(12.8%)
Other	36(13.4%)	13(21.3%)	13(22.8%)	0	0	19(11.2%)	16(12.0%)	3(8.3%)	4(10.3%)

					Cohort 1: Newly Determined Advanced BCC Patients (Non-BCCNS) Metastatic BCC (mBCC)				
			Vismo (N=2)			No Vismo (N=1)			
	All (N=4)	All (N=2)	Vismo Only (N=2)	Surgery Only (N=0)	Other Therapy (N=0)	All (N=1)	Surgery Only (N=1)	Other Therapy (N=0)	No Treatment (N=1)
If Private Insurance, [3]									

HMO (Health Maintenance Organization)	1(33.3%)	1(50.0%)	1(50.0%)	0	0	0	0	0	0
POS (Point of Service)	1(33.3%)	1(50.0%)	1(50.0%)	0	0	0	0	0	0
PPO (Preferred Provider Organization)	0	0	0	0	0	0	0	0	0
FFS (Fee for Service) or Indemnity	0	0	0	0	0	0	0	0	0
Unknown	1(33.3%)	0	0	0	0	0	0	0	1(100%)
Other	0	0	0	0	0	0	0	0	0

Note: “Vismo” group is defined as initiating vismodegib less than or equal to 90 days after the date of determination of locally advanced BCC (laBCC) or metastatic BCC (mBCC); “No Vismo” group is defined as not initiating vismodegib (but other BCC treatment) less than or equal to 90 days after the date of determination of laBCC or mBCC; “No Treatment” group is defined as not receiving any BCC treatment less than or equal to 90 days after the date of determination of laBCC or mBCC.

“Vismo Only” and “Surgery Only” are defined as having only 1 of these treatments less than or equal to 90 days after the date of determination of laBCC or mBCC; “Other Therapy” includes individual or combination treatments (e.g., surgery, PDT, ED&C, topical treatment, cryosurgery, systemic treatment, other) initiated less than or equal to 90 days after date of determination of laBCC or mBCC. For example, if patient receives topical treatment followed by vismo followed by surgery, then all 3 treatments must be initiated within the 90 days of the date of determination of disease.

In System Organ Class and Preferred Term summarization, a patient is counted once if the patient reported one or more conditions or procedures. Percentages are based on the number of enrolled patients in each cohort. Medical history and surgical history are coded using MedDRA version 20.0 and system organ classes and preferred terms are sorted in decreasing frequency based on the overall number of reports in Cohort 1.

	Cohort 1: Newly Determined Advanced BCC Patients (Non-BCCNS) Locally Advanced BCC (laBCC)								
		Vismo (N=115)			No Vismo (N=251)				
	All (N=433)	All (N=115)	Vismo Only (N=102)	Surgery Only (N=6)	Other Therapy (N=7)	All (N=251)	Surgery Only (N=198)	Other Therapy (N=53)	No Treatment (N=67)
Patients With Any Non-BCC Medical History or Surgical History	417(96.3%)	110(95.7%)	97(95.1%)	6(100%)	7(100%)	242(96.4%)	189(95.5%)	53(100%)	65(97.0%)
Surgical and medical procedures	261(60.3%)	60(52.2%)	55(53.9%)	2(33.3%)	3(42.9%)	162(64.5%)	123(62.1%)	39(73.6%)	39(58.2%)
Hysterectomy	40(9.2%)	5(4.3%)	4(3.9%)	0	1(14.3%)	34(13.5%)	27(13.6%)	7(13.2%)	1(1.5%)
Appendicectomy	31(7.2%)	8(7.0%)	8(7.8%)	0	0	18(7.2%)	15(7.6%)	3(5.7%)	5(7.5%)
Cholecystectomy	23(5.3%)	5(4.3%)	4(3.9%)	0	1(14.3%)	16(6.4%)	11(5.6%)	5(9.4%)	2(3.0%)
Skin neoplasm excision	22(5.1%)	8(7.0%)	7(6.9%)	1(16.7%)	0	13(5.2%)	12(6.1%)	1(1.9%)	1(1.5%)
Tonsillectomy	21(4.8%)	7(6.1%)	6(5.9%)	0	1(14.3%)	10(4.0%)	9(4.5%)	1(1.9%)	4(6.0%)
Cataract operation	19(4.4%)	3(2.6%)	2(2.0%)	0	1(14.3%)	12(4.8%)	6(3.0%)	6(11.3%)	4(6.0%)
Cancer surgery	15(3.5%)	4(3.5%)	2(2.0%)	1(16.7%)	1(14.3%)	6(2.4%)	6(3.0%)	0	5(7.5%)
Knee arthroplasty	14(3.2%)	2(1.7%)	1(1.0%)	0	1(14.3%)	8(3.2%)	4(2.0%)	4(7.5%)	4(6.0%)

Cardiac pacemaker insertion	13(3.0%)	3(2.6%)	3(2.9%)	0	0	6(2.4%)	4(2.0%)	2(3.8%)	4(6.0%)
Coronary artery bypass	13(3.0%)	1(0.9%)	1(1.0%)	0	0	7(2.8%)	4(2.0%)	3(5.7%)	5(7.5%)
Female sterilisation	12(2.8%)	2(1.7%)	2(2.0%)	0	0	7(2.8%)	4(2.0%)	3(5.7%)	3(4.5%)
Hip arthroplasty	12(2.8%)	3(2.6%)	2(2.0%)	0	1(14.3%)	8(3.2%)	6(3.0%)	2(3.8%)	1(1.5%)
Skin cryotherapy	12(2.8%)	0	0	0	0	9(3.6%)	6(3.0%)	3(5.7%)	3(4.5%)
					Cohort 1: Newly Determined Advanced BCC Patients (Non-BCCNS) Metastatic BCC (mBCC)				
			Vismo (N=2)		No Vismo (N=1)				
	All (N=4)	All (N=2)	Vismo Only (N=2)	Surgery Only (N=0)	Other Therapy (N=0)	All (N=1)	Surgery Only (N=1)	Other Therapy (N=0)	No Treatment (N=1)
Patients With Any Non-BCC Medical History or Surgical History	4(100%)	2(100%)	2(100%)	0	0	1(100%)	1(100%)	0	1(100%)
Surgical and medical procedures	3(75.0%)	1(50.0%)	1(50.0%)	0	0	1(100%)	1(100%)	0	1(100%)
Hysterectomy	0	0	0	0	0	0	0	0	0
Appendicectomy	0	0	0	0	0	0	0	0	0
Cholecystectomy	1(25.0%)	0	0	0	0	1(100%)	1(100%)	0	0
Skin neoplasm excision	0	0	0	0	0	0	0	0	0
Tonsillectomy	0	0	0	0	0	0	0	0	0
Cataract operation	1(25.0%)	0	0	0	0	1(100%)	1(100%)	0	0
Cancer surgery	0	0	0	0	0	0	0	0	0

Knee arthroplasty	0	0	0	0	0	0	0	0	0
Cardiac pacemaker insertion	0	0	0	0	0	0	0	0	0
Coronary artery bypass	0	0	0	0	0	0	0	0	0
Female sterilisation	1(25.0%)	1(50.0%)	1(50.0%)	0	0	0	0	0	0
Hip arthroplasty	0	0	0	0	0	0	0	0	0
Skin cryotherapy	0	0	0	0	0	0	0	0	0

					Cohort 1: Newly Determined Advanced BCC Patients (Non-BCCNS) Locally Advanced BCC (laBCC)				
		Vismo (N=115)			No Vismo (N=251)				
	All (N=433)	All (N=115)	Vismo Only (N=102)	Surgery Only (N=6)	Other Therapy (N=7)	All (N=251)	Surgery Only (N=198)	Other Therapy (N=53)	No Treatment (N=67)
Surgical and medical procedures (Cont'd)									
Coronary arterial stent insertion	11(2.5%)	2(1.7%)	2(2.0%)	0	0	5(2.0%)	3(1.5%)	2(3.8%)	4(6.0%)
Micrographic skin surgery	11(2.5%)	2(1.7%)	2(2.0%)	0	0	9(3.6%)	8(4.0%)	1(1.9%)	0
Cryotherapy	10(2.3%)	1(0.9%)	0	1(16.7%)	0	9(3.6%)	7(3.5%)	2(3.8%)	0
Hernia repair	10(2.3%)	1(0.9%)	1(1.0%)	0	0	9(3.6%)	6(3.0%)	3(5.7%)	0
Inguinal hernia repair	9(2.1%)	0	0	0	0	8(3.2%)	4(2.0%)	4(7.5%)	1(1.5%)
Rotator cuff repair	10(2.3%)	1(0.9%)	0	0	1(14.3%)	6(2.4%)	5(2.5%)	1(1.9%)	3(4.5%)
Tenoplasty	8(1.8%)	1(0.9%)	1(1.0%)	0	0	6(2.4%)	5(2.5%)	1(1.9%)	1(1.5%)
Fracture treatment	7(1.6%)	1(0.9%)	1(1.0%)	0	0	5(2.0%)	4(2.0%)	1(1.9%)	1(1.5%)

Mastectomy	7(1.6%)	1(0.9%)	1(1.0%)	0	0	6(2.4%)	6(3.0%)	0	0
Intervertebral disc operation	6(1.4%)	2(1.7%)	1(1.0%)	0	1(14.3%)	3(1.2%)	2(1.0%)	1(1.9%)	1(1.5%)
Plastic surgery to the face	6(1.4%)	2(1.7%)	2(2.0%)	0	0	4(1.6%)	3(1.5%)	1(1.9%)	0
Radiotherapy	6(1.4%)	2(1.7%)	1(1.0%)	1(16.7%)	0	3(1.2%)	3(1.5%)	0	1(1.5%)
Spinal operation	6(1.4%)	0	0	0	0	4(1.6%)	2(1.0%)	2(3.8%)	2(3.0%)
Tumour excision	6(1.4%)	0	0	0	0	6(2.4%)	4(2.0%)	2(3.8%)	0
Caesarean section	5(1.2%)	0	0	0	0	4(1.6%)	3(1.5%)	1(1.9%)	1(1.5%)

					Cohort 1: Newly Determined Advanced BCC Patients (Non-BCCNS) Metastatic BCC (mBCC)				
			Vismo (N=2)			No Vismo (N=1)			
	All (N=4)	All (N=2)	Vismo Only (N=2)	Surgery Only (N=0)	Other Therapy (N=0)	All (N=1)	Surgery Only (N=1)	Other Therapy (N=0)	No Treatment (N=1)
Surgical and medical procedures (Cont'd)									
Coronary arterial stent insertion	0	0	0	0	0	0	0	0	0
Micrographic skin surgery	0	0	0	0	0	0	0	0	0
Cryotherapy	0	0	0	0	0	0	0	0	0
Hernia repair	0	0	0	0	0	0	0	0	0
Inguinal hernia repair	1(25.0%)	0	0	0	0	1(100%)	1(100%)	0	0
Rotator cuff repair	0	0	0	0	0	0	0	0	0
Tenoplasty	0	0	0	0	0	0	0	0	0
Fracture treatment	0	0	0	0	0	0	0	0	0
Mastectomy	0	0	0	0	0	0	0	0	0

Intervertebral disc operation	0	0	0	0	0	0	0	0	0
Plastic surgery to the face	0	0	0	0	0	0	0	0	0
Radiotherapy	0	0	0	0	0	0	0	0	0
Spinal operation	0	0	0	0	0	0	0	0	0
Tumour excision	0	0	0	0	0	0	0	0	0
Caesarean section	0	0	0	0	0	0	0	0	0
	Cohort 1: Newly Determined Advanced BCC Patients (Non-BCCNS) Locally Advanced BCC (laBCC)								
		Vismo (N=115)				No Vismo (N=251)			
	All (N=433)	All (N=115)	Vismo Only (N=102)	Surgery Only (N=6)	Other Therapy (N=7)	All (N=251)	Surgery Only (N=198)	Other Therapy (N=53)	No Treatment (N=67)
Surgical and medical procedures (Cont'd)									
Knee operation	5(1.2%)	1(0.9%)	1(1.0%)	0	0	3(1.2%)	2(1.0%)	1(1.9%)	1(1.5%)
Skin graft	5(1.2%)	3(2.6%)	3(2.9%)	0	0	1(0.4%)	1(0.5%)	0	1(1.5%)
Vasectomy	5(1.2%)	3(2.6%)	2(2.0%)	0	1(14.3%)	2(0.8%)	2(1.0%)	0	0
Breast conserving surgery	4(0.9%)	0	0	0	0	4(1.6%)	3(1.5%)	1(1.9%)	0
Carpal tunnel decompression	4(0.9%)	1(0.9%)	0	0	1(14.3%)	3(1.2%)	2(1.0%)	1(1.9%)	0
Haemorrhoid operation	4(0.9%)	1(0.9%)	1(1.0%)	0	0	3(1.2%)	1(0.5%)	2(3.8%)	0
Prostatectomy	4(0.9%)	0	0	0	0	3(1.2%)	2(1.0%)	1(1.9%)	1(1.5%)
Renal stone removal	4(0.9%)	0	0	0	0	2(0.8%)	2(1.0%)	0	2(3.0%)
Spinal fusion surgery	4(0.9%)	2(1.7%)	0	1(16.7%)	1(14.3%)	2(0.8%)	2(1.0%)	0	0
Thyroidectomy	4(0.9%)	1(0.9%)	0	0	1(14.3%)	3(1.2%)	2(1.0%)	1(1.9%)	0

Uterine dilation and curettage	3(0.7%)	0	0	0	0	3(1.2%)	2(1.0%)	1(1.9%)	0
Anticoagulant therapy	3(0.7%)	0	0	0	0	2(0.8%)	2(1.0%)	0	1(1.5%)
Cardiac ablation	3(0.7%)	0	0	0	0	1(0.4%)	1(0.5%)	0	2(3.0%)
Chemotherapy	3(0.7%)	0	0	0	0	3(1.2%)	3(1.5%)	0	0
Chondroplasty	3(0.7%)	0	0	0	0	2(0.8%)	2(1.0%)	0	1(1.5%)
Coronary angioplasty	2(0.5%)	0	0	0	0	1(0.4%)	1(0.5%)	0	1(1.5%)
					Cohort 1: Newly Determined Advanced BCC Patients (Non-BCCNS) Metastatic BCC (mBCC)				
			Vismo (N=2)			No Vismo (N=1)			
	All (N=4)	All (N=2)	Vismo Only (N=2)	Surgery Only (N=0)	Other Therapy (N=0)	All (N=1)	Surgery Only (N=1)	Other Therapy (N=0)	No Treatment (N=1)
Surgical and medical procedures (Cont'd)									
Knee operation	0	0	0	0	0	0	0	0	0
Skin graft	0	0	0	0	0	0	0	0	0
Vasectomy	0	0	0	0	0	0	0	0	0
Breast conserving surgery	0	0	0	0	0	0	0	0	0
Carpal tunnel decompression	0	0	0	0	0	0	0	0	0
Haemorrhoid operation	0	0	0	0	0	0	0	0	0
Prostatectomy	0	0	0	0	0	0	0	0	0
Renal stone removal	0	0	0	0	0	0	0	0	0
Spinal fusion surgery	0	0	0	0	0	0	0	0	0
Thyroidectomy	0	0	0	0	0	0	0	0	0

Uterine dilation and curettage	1(25.0%)	1(50.0%)	1(50.0%)	0	0	0	0	0	0
Anticoagulant therapy	0	0	0	0	0	0	0	0	0
Cardiac ablation	0	0	0	0	0	0	0	0	0
Chemotherapy	0	0	0	0	0	0	0	0	0
Chondroplasty	0	0	0	0	0	0	0	0	0
Coronary angioplasty	1(25.0%)	0	0	0	0	0	0	0	1(100%)
					Cohort 1: Newly Determined Advanced BCC Patients (Non-BCCNS) Locally Advanced BCC (laBCC)				
		Vismo (N=115)				No Vismo (N=251)			
	All (N=433)	All (N=115)	Vismo Only (N=102)	Surgery Only (N=6)	Other Therapy (N=7)	All (N=251)	Surgery Only (N=198)	Other Therapy (N=53)	No Treatment (N=67)
Surgical and medical procedures (Cont'd)									
Cystopexy	3(0.7%)	1(0.9%)	1(1.0%)	0	0	2(0.8%)	1(0.5%)	1(1.9%)	0
Eye operation	3(0.7%)	2(1.7%)	2(2.0%)	0	0	1(0.4%)	1(0.5%)	0	0
Hernia hiatus repair	3(0.7%)	1(0.9%)	0	0	1(14.3%)	1(0.4%)	1(0.5%)	0	1(1.5%)
Laser therapy	3(0.7%)	1(0.9%)	0	0	1(14.3%)	2(0.8%)	2(1.0%)	0	0
Limb operation	3(0.7%)	1(0.9%)	1(1.0%)	0	0	2(0.8%)	1(0.5%)	1(1.9%)	0
Mammoplasty	3(0.7%)	0	0	0	0	2(0.8%)	0	2(3.8%)	1(1.5%)
Nasal septal operation	3(0.7%)	0	0	0	0	2(0.8%)	1(0.5%)	1(1.9%)	1(1.5%)
Radiotherapy to prostate	3(0.7%)	0	0	0	0	1(0.4%)	1(0.5%)	0	2(3.0%)
Retinal operation	3(0.7%)	1(0.9%)	0	0	1(14.3%)	2(0.8%)	2(1.0%)	0	0
Spinal laminectomy	3(0.7%)	0	0	0	0	2(0.8%)	1(0.5%)	1(1.9%)	1(1.5%)

Surgery	3(0.7%)	2(1.7%)	2(2.0%)	0	0	1(0.4%)	1(0.5%)	0	0
Umbilical hernia repair	3(0.7%)	1(0.9%)	0	0	1(14.3%)	2(0.8%)	1(0.5%)	1(1.9%)	0
Wisdom teeth removal	3(0.7%)	0	0	0	0	3(1.2%)	3(1.5%)	0	0
Wrist surgery	3(0.7%)	0	0	0	0	2(0.8%)	2(1.0%)	0	1(1.5%)
Abdominal hernia repair	2(0.5%)	0	0	0	0	2(0.8%)	2(1.0%)	0	0
Acoustic neuroma removal	2(0.5%)	0	0	0	0	2(0.8%)	2(1.0%)	0	0
Adenotonsillectomy	2(0.5%)	2(1.7%)	2(2.0%)	0	0	0	0	0	0
					Cohort 1: Newly Determined Advanced BCC Patients (Non-BCCNS) Metastatic BCC (mBCC)				
		Vismo (N=2)			No Vismo (N=1)				
	All (N=4)	All (N=2)	Vismo Only (N=2)	Surgery Only (N=0)	Other Therapy (N=0)	All (N=1)	Surgery Only (N=1)	Other Therapy (N=0)	No Treatment (N=1)
Surgical and medical procedures (Cont'd)									
Cystopexy	0	0	0	0	0	0	0	0	0
Eye operation	0	0	0	0	0	0	0	0	0
Hernia hiatus repair	0	0	0	0	0	0	0	0	0
Laser therapy	0	0	0	0	0	0	0	0	0
Limb operation	0	0	0	0	0	0	0	0	0
Mammoplasty	0	0	0	0	0	0	0	0	0
Nasal septal operation	0	0	0	0	0	0	0	0	0
Radiotherapy to prostate	0	0	0	0	0	0	0	0	0
Retinal operation	0	0	0	0	0	0	0	0	0

Spinal laminectomy	0	0	0	0	0	0	0	0	0
Surgery	0	0	0	0	0	0	0	0	0
Umbilical hernia repair	0	0	0	0	0	0	0	0	0
Wisdom teeth removal	0	0	0	0	0	0	0	0	0
Wrist surgery	0	0	0	0	0	0	0	0	0
Abdominal hernia repair	0	0	0	0	0	0	0	0	0
Acoustic neuroma removal	0	0	0	0	0	0	0	0	0
Adenotonsillectomy	0	0	0	0	0	0	0	0	0
						Cohort 1: Newly Determined Advanced BCC Patients (Non-BCCNS) Locally Advanced BCC (laBCC)			
		Vismo (N=115)				No Vismo (N=251)			
	All (N=433)	All (N=115)	Vismo Only (N=102)	Surgery Only (N=6)	Other Therapy (N=7)	All (N=251)	Surgery Only (N=198)	Other Therapy (N=53)	No Treatment (N=67)
Surgical and medical procedures (Cont'd)									
Angioplasty	2(0.5%)	1(0.9%)	1(1.0%)	0	0	1(0.4%)	1(0.5%)	0	0
Ankle operation	2(0.5%)	0	0	0	0	2(0.8%)	2(1.0%)	0	0
Aortic aneurysm repair	2(0.5%)	1(0.9%)	1(1.0%)	0	0	0	0	0	1(1.5%)
Aortic valve replacement	2(0.5%)	0	0	0	0	1(0.4%)	1(0.5%)	0	1(1.5%)
Cardioversion	2(0.5%)	0	0	0	0	2(0.8%)	1(0.5%)	1(1.9%)	0
Carotid angioplasty	2(0.5%)	1(0.9%)	1(1.0%)	0	0	1(0.4%)	0	1(1.9%)	0

Carotid endarterectomy	2(0.5%)	0	0	0	0	2(0.8%)	1(0.5%)	1(1.9%)	0
Colectomy	2(0.5%)	0	0	0	0	1(0.4%)	0	1(1.9%)	1(1.5%)
Corneal transplant	2(0.5%)	1(0.9%)	1(1.0%)	0	0	1(0.4%)	1(0.5%)	0	0
Gastric bypass	2(0.5%)	1(0.9%)	1(1.0%)	0	0	1(0.4%)	0	1(1.9%)	0
Gastrostomy	2(0.5%)	1(0.9%)	1(1.0%)	0	0	1(0.4%)	1(0.5%)	0	0
Heart valve replacement	2(0.5%)	0	0	0	0	1(0.4%)	1(0.5%)	0	1(1.5%)
Intraocular lens implant	2(0.5%)	1(0.9%)	0	0	1(14.3%)	1(0.4%)	1(0.5%)	0	0
Joint arthroplasty	2(0.5%)	1(0.9%)	1(1.0%)	0	0	0	0	0	1(1.5%)
Joint surgery	2(0.5%)	1(0.9%)	0	0	1(14.3%)	1(0.4%)	1(0.5%)	0	0
Keratomileusis	2(0.5%)	1(0.9%)	0	0	1(14.3%)	1(0.4%)	1(0.5%)	0	0
Medical device implantation	2(0.5%)	1(0.9%)	1(1.0%)	0	0	1(0.4%)	1(0.5%)	0	0

					Cohort 1: Newly Determined Advanced BCC Patients (Non-BCCNS) Metastatic BCC (mBCC)				
			Vismo (N=2)			No Vismo (N=1)			
	All (N=4)	All (N=2)	Vismo Only (N=2)	Surgery Only (N=0)	Other Therapy (N=0)	All (N=1)	Surgery Only (N=1)	Other Therapy (N=0)	No Treatment (N=1)
Surgical and medical procedures (Cont'd)									
Angioplasty	0	0	0	0	0	0	0	0	0
Ankle operation	0	0	0	0	0	0	0	0	0
Aortic aneurysm repair	0	0	0	0	0	0	0	0	0
Aortic valve replacement	0	0	0	0	0	0	0	0	0

Cardioversion	0	0	0	0	0	0	0	0	0
Carotid angioplasty	0	0	0	0	0	0	0	0	0
Carotid endarterectomy	0	0	0	0	0	0	0	0	0
Colectomy	0	0	0	0	0	0	0	0	0
Corneal transplant	0	0	0	0	0	0	0	0	0
Gastric bypass	0	0	0	0	0	0	0	0	0
Gastrostomy	0	0	0	0	0	0	0	0	0
Heart valve replacement	0	0	0	0	0	0	0	0	0
Intraocular lens implant	0	0	0	0	0	0	0	0	0
Joint arthroplasty	0	0	0	0	0	0	0	0	0
Joint surgery	0	0	0	0	0	0	0	0	0
Keratomileusis	0	0	0	0	0	0	0	0	0
Medical device implantation	0	0	0	0	0	0	0	0	0
					Cohort 1: Newly Determined Advanced BCC Patients (Non-BCCNS) Locally Advanced BCC (laBCC)				
			Vismo (N=115)			No Vismo (N=251)			
	All (N=433)	All (N=115)	Vismo Only (N=102)	Surgery Only (N=6)	Other Therapy (N=7)	All (N=251)	Surgery Only (N=198)	Other Therapy (N=53)	No Treatment (N=67)
Surgical and medical procedures (Cont'd)									
Medical device removal	2(0.5%)	1(0.9%)	0	0	1(14.3%)	0	0	0	1(1.5%)
Nephrectomy	2(0.5%)	1(0.9%)	1(1.0%)	0	0	1(0.4%)	1(0.5%)	0	0

Open reduction of fracture	2(0.5%)	1(0.9%)	1(1.0%)	0	0	1(0.4%)	0	1(1.9%)	0
Orbitotomy	2(0.5%)	1(0.9%)	1(1.0%)	0	0	0	0	0	1(1.5%)
Pain management	2(0.5%)	1(0.9%)	0	0	1(14.3%)	1(0.4%)	0	1(1.9%)	0
Prostatic operation	2(0.5%)	0	0	0	0	1(0.4%)	1(0.5%)	0	1(1.5%)
Renal transplant	2(0.5%)	0	0	0	0	1(0.4%)	1(0.5%)	0	1(1.5%)
Shoulder operation	2(0.5%)	0	0	0	0	2(0.8%)	2(1.0%)	0	0
Sinus operation	2(0.5%)	0	0	0	0	1(0.4%)	0	1(1.9%)	1(1.5%)
Soft tissue flap operation	2(0.5%)	1(0.9%)	1(1.0%)	0	0	0	0	0	1(1.5%)
Stent placement	2(0.5%)	0	0	0	0	1(0.4%)	0	1(1.9%)	1(1.5%)
Synovial cyst removal	2(0.5%)	0	0	0	0	2(0.8%)	2(1.0%)	0	0
Tracheostomy	2(0.5%)	1(0.9%)	1(1.0%)	0	0	1(0.4%)	1(0.5%)	0	0
Transurethral prostatectomy	2(0.5%)	0	0	0	0	2(0.8%)	1(0.5%)	1(1.9%)	0
Varicose vein operation	2(0.5%)	1(0.9%)	1(1.0%)	0	0	0	0	0	1(1.5%)
Abdominal operation	1(0.2%)	0	0	0	0	0	0	0	1(1.5%)
Abdominoplasty	1(0.2%)	0	0	0	0	1(0.4%)	0	1(1.9%)	0

					Cohort 1: Newly Determined Advanced BCC Patients (Non-BCCNS) Metastatic BCC (mBCC)				
		Vismo (N=2)			No Vismo (N=1)				
	All (N=4)	All (N=2)	Vismo Only (N=2)	Surgery Only (N=0)	Other Therapy (N=0)	All (N=1)	Surgery Only (N=1)	Other Therapy (N=0)	No Treatment (N=1)
Surgical and medical procedures (Cont'd)									

		Cohort 1: Newly Determined Advanced BCC Patients (Non-BCCNS) Locally Advanced BCC (laBCC)							
		Vismo (N=115)				No Vismo (N=251)			
	All (N=433)	All (N=115)	Vismo Only (N=102)	Surgery Only (N=6)	Other Therapy (N=7)	All (N=251)	Surgery Only (N=198)	Other Therapy (N=53)	No Treatment (N=67)
Medical device removal	0	0	0	0	0	0	0	0	0
Nephrectomy	0	0	0	0	0	0	0	0	0
Open reduction of fracture	0	0	0	0	0	0	0	0	0
Orbitotomy	0	0	0	0	0	0	0	0	0
Pain management	0	0	0	0	0	0	0	0	0
Prostatic operation	0	0	0	0	0	0	0	0	0
Renal transplant	0	0	0	0	0	0	0	0	0
Shoulder operation	0	0	0	0	0	0	0	0	0
Sinus operation	0	0	0	0	0	0	0	0	0
Soft tissue flap operation	0	0	0	0	0	0	0	0	0
Stent placement	0	0	0	0	0	0	0	0	0
Synovial cyst removal	0	0	0	0	0	0	0	0	0
Tracheostomy	0	0	0	0	0	0	0	0	0
Transurethral prostatectomy	0	0	0	0	0	0	0	0	0
Varicose vein operation	0	0	0	0	0	0	0	0	0
Abdominal operation	0	0	0	0	0	0	0	0	0
Abdominoplasty	0	0	0	0	0	0	0	0	0

Surgical and medical procedures (Cont'd)									
Abscess drainage	1(0.2%)	1(0.9%)	1(1.0%)	0	0	0	0	0	0
Adhesiolysis	1(0.2%)	0	0	0	0	1(0.4%)	1(0.5%)	0	0
Antibiotic therapy	1(0.2%)	0	0	0	0	1(0.4%)	1(0.5%)	0	0
Arthrodesis	1(0.2%)	0	0	0	0	1(0.4%)	1(0.5%)	0	0
Bladder catheterisation	1(0.2%)	0	0	0	0	1(0.4%)	1(0.5%)	0	0
Blepharoplasty	1(0.2%)	0	0	0	0	1(0.4%)	1(0.5%)	0	0
Bone debridement	1(0.2%)	0	0	0	0	1(0.4%)	1(0.5%)	0	0
Bone lesion excision	1(0.2%)	0	0	0	0	1(0.4%)	1(0.5%)	0	0
Bone marrow transplant	1(0.2%)	1(0.9%)	1(1.0%)	0	0	0	0	0	0
Brain operation	1(0.2%)	0	0	0	0	0	0	0	1(1.5%)
Breast operation	1(0.2%)	0	0	0	0	0	0	0	1(1.5%)
Breast reconstruction	1(0.2%)	0	0	0	0	1(0.4%)	1(0.5%)	0	0
Breast tumour excision	1(0.2%)	0	0	0	0	1(0.4%)	1(0.5%)	0	0
Cardiac operation	1(0.2%)	1(0.9%)	1(1.0%)	0	0	0	0	0	0
Carotid artery stent insertion	1(0.2%)	0	0	0	0	1(0.4%)	0	1(1.9%)	0
Cholecystostomy	1(0.2%)	0	0	0	0	1(0.4%)	0	1(1.9%)	0
					Cohort 1: Newly Determined Advanced BCC Patients (Non-BCCNS) Metastatic BCC (mBCC)				
			Vismo (N=2)			No Vismo (N=1)			
	All (N=4)	All (N=2)	Vismo Only (N=2)	Surgery Only (N=0)	Other Therapy (N=0)	All (N=1)	Surgery Only (N=1)	Other Therapy (N=0)	No Treatment (N=1)

					Cohort 1: Newly Determined Advanced BCC Patients (Non-BCCNS) Locally Advanced BCC (laBCC)				
		Vismo (N=115)			No Vismo (N=251)				
	All (N=433)	All (N=115)	Vismo Only (N=102)	Surgery Only (N=6)	Other Therapy (N=7)	All (N=251)	Surgery Only (N=198)	Other Therapy (N=53)	No Treatment (N=67)
Surgical and medical procedures (Cont'd)									
Abscess drainage	0	0	0	0	0	0	0	0	0
Adhesiolysis	0	0	0	0	0	0	0	0	0
Antibiotic therapy	0	0	0	0	0	0	0	0	0
Arthrodesis	0	0	0	0	0	0	0	0	0
Bladder catheterisation	0	0	0	0	0	0	0	0	0
Blepharoplasty	0	0	0	0	0	0	0	0	0
Bone debridement	0	0	0	0	0	0	0	0	0
Bone lesion excision	0	0	0	0	0	0	0	0	0
Bone marrow transplant	0	0	0	0	0	0	0	0	0
Brain operation	0	0	0	0	0	0	0	0	0
Breast operation	0	0	0	0	0	0	0	0	0
Breast reconstruction	0	0	0	0	0	0	0	0	0
Breast tumour excision	0	0	0	0	0	0	0	0	0
Cardiac operation	0	0	0	0	0	0	0	0	0
Carotid artery stent insertion	0	0	0	0	0	0	0	0	0
Cholecystostomy	0	0	0	0	0	0	0	0	0

Surgical and medical procedures (Cont'd)									
Cranioplasty	1(0.2%)	0	0	0	0	1(0.4%)	0	1(1.9%)	0
Craniotomy	1(0.2%)	0	0	0	0	1(0.4%)	1(0.5%)	0	0
Cyst removal	1(0.2%)	0	0	0	0	1(0.4%)	1(0.5%)	0	0
Dacryocystorhinostomy	1(0.2%)	0	0	0	0	0	0	0	1(1.5%)
Dental implantation	1(0.2%)	0	0	0	0	1(0.4%)	0	1(1.9%)	0
Dupuytren's contracture operation	1(0.2%)	0	0	0	0	1(0.4%)	1(0.5%)	0	0
Elbow operation	1(0.2%)	0	0	0	0	1(0.4%)	1(0.5%)	0	0
Eyelid operation	1(0.2%)	1(0.9%)	1(1.0%)	0	0	0	0	0	0
Finger amputation	1(0.2%)	1(0.9%)	1(1.0%)	0	0	0	0	0	0
Foot operation	1(0.2%)	0	0	0	0	1(0.4%)	1(0.5%)	0	0
Gallbladder operation	1(0.2%)	0	0	0	0	1(0.4%)	1(0.5%)	0	0
Gastrointestinal tube insertion	1(0.2%)	0	0	0	0	1(0.4%)	1(0.5%)	0	0
Haemangioma removal	1(0.2%)	0	0	0	0	1(0.4%)	0	1(1.9%)	0
Heart transplant	1(0.2%)	0	0	0	0	1(0.4%)	1(0.5%)	0	0
Heart valve operation	1(0.2%)	0	0	0	0	1(0.4%)	0	1(1.9%)	0
					Cohort 1: Newly Determined Advanced BCC Patients (Non-BCCNS) Metastatic BCC (mBCC)				
			Vismo (N=2)			No Vismo (N=1)			
	All (N=4)	All (N=2)	Vismo Only (N=2)	Surgery Only (N=0)	Other Therapy (N=0)	All (N=1)	Surgery Only (N=1)	Other Therapy (N=0)	No Treatment (N=1)
Surgical and medical procedures (Cont'd)									

Cranioplasty	0	0	0	0	0	0	0	0	0
Craniotomy	0	0	0	0	0	0	0	0	0
Cyst removal	0	0	0	0	0	0	0	0	0
Dacryocystorhinostomy	0	0	0	0	0	0	0	0	0
Dental implantation	0	0	0	0	0	0	0	0	0
Dupuytren's contracture operation	0	0	0	0	0	0	0	0	0
Elbow operation	0	0	0	0	0	0	0	0	0
Eyelid operation	0	0	0	0	0	0	0	0	0
Finger amputation	0	0	0	0	0	0	0	0	0
Foot operation	0	0	0	0	0	0	0	0	0
Gallbladder operation	0	0	0	0	0	0	0	0	0
Gastrointestinal tube insertion	0	0	0	0	0	0	0	0	0
Haemangioma removal	0	0	0	0	0	0	0	0	0
Heart transplant	0	0	0	0	0	0	0	0	0
Heart valve operation	0	0	0	0	0	0	0	0	0
					Cohort 1: Newly Determined Advanced BCC Patients (Non-BCCNS) Locally Advanced BCC (laBCC)				
			Vismo (N=115)			No Vismo (N=251)			
	All (N=433)	All (N=115)	Vismo Only (N=102)	Surgery Only (N=6)	Other Therapy (N=7)	All (N=251)	Surgery Only (N=198)	Other Therapy (N=53)	No Treatment (N=67)
Surgical and medical procedures (Cont'd)									
Hip surgery	1(0.2%)	1(0.9%)	1(1.0%)	0	0	0	0	0	0

Implantable defibrillator insertion	1(0.2%)	1(0.9%)	1(1.0%)	0	0	0	0	0	0
Intestinal operation	1(0.2%)	0	0	0	0	1(0.4%)	0	1(1.9%)	0
Intra-cerebral aneurysm operation	1(0.2%)	0	0	0	0	1(0.4%)	1(0.5%)	0	0
Intramedullary rod insertion	1(0.2%)	1(0.9%)	1(1.0%)	0	0	0	0	0	0
Ligament operation	1(0.2%)	0	0	0	0	1(0.4%)	1(0.5%)	0	0
Lung operation	1(0.2%)	0	0	0	0	1(0.4%)	1(0.5%)	0	0
Mastoid operation	1(0.2%)	1(0.9%)	1(1.0%)	0	0	0	0	0	0
Maxillofacial operation	1(0.2%)	1(0.9%)	1(1.0%)	0	0	0	0	0	0
Meniscus operation	1(0.2%)	0	0	0	0	0	0	0	1(1.5%)
Meniscus removal	1(0.2%)	1(0.9%)	0	0	1(14.3%)	0	0	0	0
Mitral valve replacement	1(0.2%)	1(0.9%)	0	0	1(14.3%)	0	0	0	0
Mole excision	1(0.2%)	0	0	0	0	1(0.4%)	1(0.5%)	0	0
Mouth cyst excision	1(0.2%)	1(0.9%)	1(1.0%)	0	0	0	0	0	0
Muscle flap operation	1(0.2%)	0	0	0	0	1(0.4%)	0	1(1.9%)	0

					Cohort 1: Newly Determined Advanced BCC Patients (Non-BCCNS) Metastatic BCC (mBCC)				
		Vismo (N=2)			No Vismo (N=1)				
	All (N=4)	All (N=2)	Vismo Only (N=2)	Surgery Only (N=0)	Other Therapy (N=0)	All (N=1)	Surgery Only (N=1)	Other Therapy (N=0)	No Treatment (N=1)
Surgical and medical procedures (Cont'd)									
Hip surgery	0	0	0	0	0	0	0	0	0

Implantable defibrillator insertion	0	0	0	0	0	0	0	0	0
Intestinal operation	0	0	0	0	0	0	0	0	0
Intra-cerebral aneurysm operation	0	0	0	0	0	0	0	0	0
Intramedullary rod insertion	0	0	0	0	0	0	0	0	0
Ligament operation	0	0	0	0	0	0	0	0	0
Lung operation	0	0	0	0	0	0	0	0	0
Mastoid operation	0	0	0	0	0	0	0	0	0
Maxillofacial operation	0	0	0	0	0	0	0	0	0
Meniscus operation	0	0	0	0	0	0	0	0	0
Meniscus removal	0	0	0	0	0	0	0	0	0
Mitral valve replacement	0	0	0	0	0	0	0	0	0
Mole excision	0	0	0	0	0	0	0	0	0
Mouth cyst excision	0	0	0	0	0	0	0	0	0
Muscle flap operation	0	0	0	0	0	0	0	0	0
					Cohort 1: Newly Determined Advanced BCC Patients (Non-BCCNS) Locally Advanced BCC (laBCC)				
			Vismo (N=115)			No Vismo (N=251)			
	All (N=433)	All (N=115)	Vismo Only (N=102)	Surgery Only (N=6)	Other Therapy (N=7)	All (N=251)	Surgery Only (N=198)	Other Therapy (N=53)	No Treatment (N=67)
Surgical and medical procedures (Cont'd)									

Neck surgery	1(0.2%)	0	0	0	0	1(0.4%)	0	1(1.9%)	0
Oophorectomy	1(0.2%)	0	0	0	0	1(0.4%)	1(0.5%)	0	0
Orchidectomy	1(0.2%)	0	0	0	0	0	0	0	1(1.5%)
Parathyroid gland operation	1(0.2%)	0	0	0	0	1(0.4%)	1(0.5%)	0	0
Parotidectomy	1(0.2%)	0	0	0	0	1(0.4%)	0	1(1.9%)	0
Penile prosthesis insertion	1(0.2%)	0	0	0	0	1(0.4%)	1(0.5%)	0	0
Peripheral artery bypass	1(0.2%)	0	0	0	0	0	0	0	1(1.5%)
Phlebectomy	1(0.2%)	1(0.9%)	1(1.0%)	0	0	0	0	0	0
Pilonidal sinus repair	1(0.2%)	0	0	0	0	1(0.4%)	1(0.5%)	0	0
Pituitary tumour removal	1(0.2%)	0	0	0	0	1(0.4%)	1(0.5%)	0	0
Pneumonectomy	1(0.2%)	0	0	0	0	1(0.4%)	0	1(1.9%)	0
Polypectomy	1(0.2%)	0	0	0	0	1(0.4%)	0	1(1.9%)	0
Prosthesis implantation	1(0.2%)	1(0.9%)	1(1.0%)	0	0	0	0	0	0
Radiculotomy	1(0.2%)	0	0	0	0	1(0.4%)	1(0.5%)	0	0
Rectal fistula repair	1(0.2%)	0	0	0	0	1(0.4%)	1(0.5%)	0	0
Removal of foreign body	1(0.2%)	0	0	0	0	0	0	0	1(1.5%)
Removal of internal fixation	1(0.2%)	0	0	0	0	1(0.4%)	0	1(1.9%)	0
					Cohort 1: Newly Determined Advanced BCC Patients (Non-BCCNS) Metastatic BCC (mBCC)				
		Vismo (N=2)			No Vismo (N=1)				
	All (N=4)	All (N=2)	Vismo Only (N=2)	Surgery Only (N=0)	Other Therapy (N=0)	All (N=1)	Surgery Only (N=1)	Other Therapy (N=0)	No Treatment (N=1)

Surgical and medical procedures (Cont'd)									
Neck surgery	0	0	0	0	0	0	0	0	0
Oophorectomy	0	0	0	0	0	0	0	0	0
Orchidectomy	0	0	0	0	0	0	0	0	0
Parathyroid gland operation	0	0	0	0	0	0	0	0	0
Parotidectomy	0	0	0	0	0	0	0	0	0
Penile prosthesis insertion	0	0	0	0	0	0	0	0	0
Peripheral artery bypass	0	0	0	0	0	0	0	0	0
Phlebectomy	0	0	0	0	0	0	0	0	0
Pilonidal sinus repair	0	0	0	0	0	0	0	0	0
Pituitary tumour removal	0	0	0	0	0	0	0	0	0
Pneumonectomy	0	0	0	0	0	0	0	0	0
Polypectomy	0	0	0	0	0	0	0	0	0
Prosthesis implantation	0	0	0	0	0	0	0	0	0
Radiculotomy	0	0	0	0	0	0	0	0	0
Rectal fistula repair	0	0	0	0	0	0	0	0	0
Removal of foreign body	0	0	0	0	0	0	0	0	0
Removal of internal fixation	0	0	0	0	0	0	0	0	0

	Cohort 1: Newly Determined Advanced BCC Patients (Non-BCCNS) Locally Advanced BCC (laBCC)		
	Vismo (N=115)	No Vismo (N=251)	

	All (N=433)	All (N=115)	Vismo Only (N=102)	Surgery Only (N=6)	Other Therapy (N=7)	All (N=251)	Surgery Only (N=198)	Other Therapy (N=53)	No Treatment (N=67)
Surgical and medical procedures (Cont'd)									
Retinopexy	1(0.2%)	1(0.9%)	1(1.0%)	0	0	0	0	0	0
Rhinectomy	1(0.2%)	1(0.9%)	1(1.0%)	0	0	0	0	0	0
Rhinoplasty	1(0.2%)	1(0.9%)	1(1.0%)	0	0	0	0	0	0
Shoulder arthroplasty	1(0.2%)	0	0	0	0	0	0	0	1(1.5%)
Sigmoidectomy	1(0.2%)	0	0	0	0	1(0.4%)	1(0.5%)	0	0
Skin operation	1(0.2%)	1(0.9%)	1(1.0%)	0	0	0	0	0	0
Small intestinal resection	1(0.2%)	1(0.9%)	1(1.0%)	0	0	0	0	0	0
Spinal rod insertion	1(0.2%)	0	0	0	0	1(0.4%)	1(0.5%)	0	0
Splenectomy	1(0.2%)	0	0	0	0	0	0	0	1(1.5%)
Suture insertion	1(0.2%)	1(0.9%)	1(1.0%)	0	0	0	0	0	0
Tendon sheath incision	1(0.2%)	1(0.9%)	0	0	1(14.3%)	0	0	0	0
Thyroid nodule removal	1(0.2%)	0	0	0	0	0	0	0	1(1.5%)
Toe amputation	1(0.2%)	0	0	0	0	1(0.4%)	1(0.5%)	0	0
Toe operation	1(0.2%)	0	0	0	0	0	0	0	1(1.5%)
Transfusion	1(0.2%)	0	0	0	0	1(0.4%)	0	1(1.9%)	0
Tricuspid valve replacement	1(0.2%)	0	0	0	0	0	0	0	1(1.5%)
Vascular cauterisation	1(0.2%)	0	0	0	0	1(0.4%)	0	1(1.9%)	0

Cohort 1: Newly Determined Advanced BCC Patients (Non-BCCNS) Metastatic BCC (mBCC)

Vismo (N=2) | No Vismo (N=1)

	All (N=4)	All (N=2)	Vismo Only (N=2)	Surgery Only (N=0)	Other Therapy (N=0)	All (N=1)	Surgery Only (N=1)	Other Therapy (N=0)	No Treatment (N=1)
Surgical and medical procedures (Cont'd)									
Retinopexy	0	0	0	0	0	0	0	0	0
Rhinectomy	0	0	0	0	0	0	0	0	0
Rhinoplasty	0	0	0	0	0	0	0	0	0
Shoulder arthroplasty	0	0	0	0	0	0	0	0	0
Sigmoidectomy	0	0	0	0	0	0	0	0	0
Skin operation	0	0	0	0	0	0	0	0	0
Small intestinal resection	0	0	0	0	0	0	0	0	0
Spinal rod insertion	0	0	0	0	0	0	0	0	0
Splenectomy	0	0	0	0	0	0	0	0	0
Suture insertion	0	0	0	0	0	0	0	0	0
Tendon sheath incision	0	0	0	0	0	0	0	0	0
Thyroid nodule removal	0	0	0	0	0	0	0	0	0
Toe amputation	0	0	0	0	0	0	0	0	0
Toe operation	0	0	0	0	0	0	0	0	0
Transfusion	0	0	0	0	0	0	0	0	0
Tricuspid valve replacement	0	0	0	0	0	0	0	0	0
Vascular cauterisation	0	0	0	0	0	0	0	0	0

		Cohort 1: Newly Determined Advanced BCC Patients (Non-BCCNS) Locally Advanced BCC (laBCC)	
	Vismo (N=115)	No Vismo (N=251)	

	All (N=433)	All (N=115)	Vismo Only (N=102)	Surgery Only (N=6)	Other Therapy (N=7)	All (N=251)	Surgery Only (N=198)	Other Therapy (N=53)	No Treatment (N=67)
Surgical and medical procedures (Cont'd)									
Vascular graft	1(0.2%)	0	0	0	0	1(0.4%)	1(0.5%)	0	0
Vascular stent insertion	1(0.2%)	0	0	0	0	0	0	0	1(1.5%)
Vitamin supplementation	1(0.2%)	0	0	0	0	1(0.4%)	1(0.5%)	0	0
Wound treatment	1(0.2%)	0	0	0	0	1(0.4%)	1(0.5%)	0	0
Vascular disorders	250(57.7%)	66(57.4%)	56(54.9%)	4(66.7%)	6(85.7%)	141(56.2%)	112(56.6%)	29(54.7%)	43(64.2%)
Hypertension	236(54.5%)	63(54.8%)	53(52.0%)	4(66.7%)	6(85.7%)	133(53.0%)	106(53.5%)	27(50.9%)	40(59.7%)
Peripheral vascular disorder	7(1.6%)	2(1.7%)	2(2.0%)	0	0	3(1.2%)	2(1.0%)	1(1.9%)	2(3.0%)
Aortic aneurysm	5(1.2%)	1(0.9%)	1(1.0%)	0	0	1(0.4%)	1(0.5%)	0	3(4.5%)
Deep vein thrombosis	4(0.9%)	1(0.9%)	1(1.0%)	0	0	3(1.2%)	1(0.5%)	2(3.8%)	0
Peripheral venous disease	4(0.9%)	1(0.9%)	1(1.0%)	0	0	1(0.4%)	0	1(1.9%)	2(3.0%)
Thrombosis	4(0.9%)	1(0.9%)	1(1.0%)	0	0	3(1.2%)	3(1.5%)	0	0
Arteriosclerosis	2(0.5%)	1(0.9%)	1(1.0%)	0	0	1(0.4%)	1(0.5%)	0	0
Essential hypertension	2(0.5%)	1(0.9%)	1(1.0%)	0	0	0	0	0	1(1.5%)
Intermittent claudication	2(0.5%)	1(0.9%)	1(1.0%)	0	0	1(0.4%)	0	1(1.9%)	0
Raynaud's phenomenon	2(0.5%)	1(0.9%)	1(1.0%)	0	0	1(0.4%)	1(0.5%)	0	0
Varicose vein	2(0.5%)	0	0	0	0	0	0	0	2(3.0%)

					Cohort 1: Newly Determined Advanced BCC Patients (Non-BCCNS) Metastatic BCC (mBCC)				
			Vismo (N=2)			No Vismo (N=1)			
	All (N=4)	All (N=2)	Vismo Only (N=2)	Surgery Only (N=0)	Other Therapy (N=0)	All (N=1)	Surgery Only (N=1)	Other Therapy (N=0)	No Treatment (N=1)
Surgical and medical procedures (Cont'd)									
Vascular graft	0	0	0	0	0	0	0	0	0
Vascular stent insertion	0	0	0	0	0	0	0	0	0
Vitamin supplementation	0	0	0	0	0	0	0	0	0
Wound treatment	0	0	0	0	0	0	0	0	0
Vascular disorders	2(50.0%)	1(50.0%)	1(50.0%)	0	0	0	0	0	1(100%)
Hypertension	2(50.0%)	1(50.0%)	1(50.0%)	0	0	0	0	0	1(100%)
Peripheral vascular disorder	0	0	0	0	0	0	0	0	0
Aortic aneurysm	0	0	0	0	0	0	0	0	0
Deep vein thrombosis	0	0	0	0	0	0	0	0	0
Peripheral venous disease	0	0	0	0	0	0	0	0	0
Thrombosis	0	0	0	0	0	0	0	0	0
Arteriosclerosis	0	0	0	0	0	0	0	0	0
Essential hypertension	0	0	0	0	0	0	0	0	0
Intermittent claudication	0	0	0	0	0	0	0	0	0
Raynaud's phenomenon	0	0	0	0	0	0	0	0	0

Varicose vein	0	0	0	0	0	0	0	0	0
					Cohort 1: Newly Determined Advanced BCC Patients (Non-BCCNS) Locally Advanced BCC (laBCC)				
		Vismo (N=115)			No Vismo (N=251)				
	All (N=433)	All (N=115)	Vismo Only (N=102)	Surgery Only (N=6)	Other Therapy (N=7)	All (N=251)	Surgery Only (N=198)	Other Therapy (N=53)	No Treatment (N=67)
Vascular disorders (Cont'd)									
Angiopathy	1(0.2%)	0	0	0	0	0	0	0	1(1.5%)
Aortic arteriosclerosis	1(0.2%)	1(0.9%)	1(1.0%)	0	0	0	0	0	0
Aortic rupture	1(0.2%)	0	0	0	0	1(0.4%)	1(0.5%)	0	0
Arterial occlusive disease	1(0.2%)	0	0	0	0	1(0.4%)	1(0.5%)	0	0
Embolism venous	1(0.2%)	0	0	0	0	1(0.4%)	1(0.5%)	0	0
Haemorrhage	1(0.2%)	1(0.9%)	1(1.0%)	0	0	0	0	0	0
Hot flush	1(0.2%)	0	0	0	0	1(0.4%)	1(0.5%)	0	0
Hypotension	1(0.2%)	0	0	0	0	1(0.4%)	1(0.5%)	0	0
Orthostatic hypotension	1(0.2%)	0	0	0	0	1(0.4%)	1(0.5%)	0	0
Peripheral arterial occlusive disease	1(0.2%)	1(0.9%)	1(1.0%)	0	0	0	0	0	0
Metabolism and nutrition disorders	190(43.9%)	44(38.3%)	37(36.3%)	1(16.7%)	6(85.7%)	113(45.0%)	89(44.9%)	24(45.3%)	33(49.3%)
Hypercholesterolaemia	83(19.2%)	22(19.1%)	19(18.6%)	1(16.7%)	2(28.6%)	48(19.1%)	39(19.7%)	9(17.0%)	13(19.4%)
Hyperlipidaemia	60(13.9%)	8(7.0%)	7(6.9%)	0	1(14.3%)	37(14.7%)	28(14.1%)	9(17.0%)	15(22.4%)
Diabetes mellitus	35(8.1%)	8(7.0%)	6(5.9%)	1(16.7%)	1(14.3%)	18(7.2%)	12(6.1%)	6(11.3%)	9(13.4%)
Type 2 diabetes mellitus	23(5.3%)	9(7.8%)	9(8.8%)	0	0	13(5.2%)	11(5.6%)	2(3.8%)	1(1.5%)

					Cohort 1: Newly Determined Advanced BCC Patients (Non-BCCNS) Metastatic BCC (mBCC)				
		Vismo (N=2)			No Vismo (N=1)				
	All (N=4)	All (N=2)	Vismo Only (N=2)	Surgery Only (N=0)	Other Therapy (N=0)	All (N=1)	Surgery Only (N=1)	Other Therapy (N=0)	No Treatment (N=1)
Vascular disorders (Cont'd)									
Angiopathy	0	0	0	0	0	0	0	0	0
Aortic arteriosclerosis	0	0	0	0	0	0	0	0	0
Aortic rupture	0	0	0	0	0	0	0	0	0
Arterial occlusive disease	0	0	0	0	0	0	0	0	0
Embolism venous	0	0	0	0	0	0	0	0	0
Haemorrhage	0	0	0	0	0	0	0	0	0
Hot flush	0	0	0	0	0	0	0	0	0
Hypotension	0	0	0	0	0	0	0	0	0
Orthostatic hypotension	0	0	0	0	0	0	0	0	0
Peripheral arterial occlusive disease	0	0	0	0	0	0	0	0	0
Metabolism and nutrition disorders	1(25.0%)	0	0	0	0	0	0	0	1(100%)
Hypercholesterolaemia	0	0	0	0	0	0	0	0	0
Hyperlipidaemia	1(25.0%)	0	0	0	0	0	0	0	1(100%)
Diabetes mellitus	0	0	0	0	0	0	0	0	0
Type 2 diabetes mellitus	0	0	0	0	0	0	0	0	0

					Cohort 1: Newly Determined Advanced BCC Patients (Non-BCCNS) Locally Advanced BCC (laBCC)				
		Vismo (N=115)			No Vismo (N=251)				

	All (N=433)	All (N=115)	Vismo Only (N=102)	Surgery Only (N=6)	Other Therapy (N=7)	All (N=251)	Surgery Only (N=198)	Other Therapy (N=53)	No Treatment (N=67)
Metabolism and nutrition disorders (Cont'd)									
Gout	13(3.0%)	0	0	0	0	9(3.6%)	8(4.0%)	1(1.9%)	4(6.0%)
Hypokalaemia	10(2.3%)	2(1.7%)	1(1.0%)	0	1(14.3%)	8(3.2%)	5(2.5%)	3(5.7%)	0
Iron deficiency	7(1.6%)	1(0.9%)	0	0	1(14.3%)	6(2.4%)	2(1.0%)	4(7.5%)	0
Vitamin D deficiency	7(1.6%)	2(1.7%)	1(1.0%)	0	1(14.3%)	4(1.6%)	3(1.5%)	1(1.9%)	1(1.5%)
Dyslipidaemia	5(1.2%)	0	0	0	0	4(1.6%)	4(2.0%)	0	1(1.5%)
Obesity	5(1.2%)	0	0	0	0	4(1.6%)	4(2.0%)	0	1(1.5%)
Hyperglycaemia	4(0.9%)	1(0.9%)	0	1(16.7%)	0	3(1.2%)	2(1.0%)	1(1.9%)	0
Hypovitaminosis	3(0.7%)	1(0.9%)	1(1.0%)	0	0	2(0.8%)	1(0.5%)	1(1.9%)	0
Vitamin B12 deficiency	3(0.7%)	1(0.9%)	1(1.0%)	0	0	0	0	0	2(3.0%)
Decreased appetite	2(0.5%)	1(0.9%)	1(1.0%)	0	0	1(0.4%)	1(0.5%)	0	0
Malnutrition	2(0.5%)	2(1.7%)	1(1.0%)	0	1(14.3%)	0	0	0	0
Type 1 diabetes mellitus	2(0.5%)	0	0	0	0	2(0.8%)	2(1.0%)	0	0
Dehydration	1(0.2%)	1(0.9%)	1(1.0%)	0	0	0	0	0	0
Diabetes mellitus inadequate control	1(0.2%)	0	0	0	0	1(0.4%)	1(0.5%)	0	0
Electrolyte imbalance	1(0.2%)	0	0	0	0	0	0	0	1(1.5%)
Fluid retention	1(0.2%)	1(0.9%)	1(1.0%)	0	0	0	0	0	0

					Cohort 1: Newly Determined Advanced BCC Patients (Non-BCCNS) Metastatic BCC (mBCC)				
			Vismo (N=2)			No Vismo (N=1)			
	All (N=4)	All (N=2)	Vismo Only (N=2)	Surgery Only (N=0)	Other Therapy (N=0)	All (N=1)	Surgery Only (N=1)	Other Therapy (N=0)	No Treatment (N=1)

	Cohort 1: Newly Determined Advanced BCC Patients (Non-BCCNS) Locally Advanced BCC (laBCC)								
		Vismo (N=115)				No Vismo (N=251)			
	All (N=433)	All (N=115)	Vismo Only (N=102)	Surgery Only (N=6)	Other Therapy (N=7)	All (N=251)	Surgery Only (N=198)	Other Therapy (N=53)	No Treatment (N=67)
Metabolism and nutrition disorders (Cont'd)									
Gout	0	0	0	0	0	0	0	0	0
Hypokalaemia	0	0	0	0	0	0	0	0	0
Iron deficiency	0	0	0	0	0	0	0	0	0
Vitamin D deficiency	0	0	0	0	0	0	0	0	0
Dyslipidaemia	0	0	0	0	0	0	0	0	0
Obesity	0	0	0	0	0	0	0	0	0
Hyperglycaemia	0	0	0	0	0	0	0	0	0
Hypovitaminosis	0	0	0	0	0	0	0	0	0
Vitamin B12 deficiency	0	0	0	0	0	0	0	0	0
Decreased appetite	0	0	0	0	0	0	0	0	0
Malnutrition	0	0	0	0	0	0	0	0	0
Type 1 diabetes mellitus	0	0	0	0	0	0	0	0	0
Dehydration	0	0	0	0	0	0	0	0	0
Diabetes mellitus inadequate control	0	0	0	0	0	0	0	0	0
Electrolyte imbalance	0	0	0	0	0	0	0	0	0
Fluid retention	0	0	0	0	0	0	0	0	0

Metabolism and nutrition disorders (Cont'd)									
Glucose tolerance impaired	1(0.2%)	0	0	0	0	1(0.4%)	1(0.5%)	0	0
Hypercalcaemia	1(0.2%)	0	0	0	0	1(0.4%)	1(0.5%)	0	0
Hypomagnesaemia	1(0.2%)	0	0	0	0	1(0.4%)	1(0.5%)	0	0
Hyponatraemia	1(0.2%)	1(0.9%)	1(1.0%)	0	0	0	0	0	0
Impaired fasting glucose	1(0.2%)	0	0	0	0	0	0	0	1(1.5%)
Lactose intolerance	1(0.2%)	0	0	0	0	0	0	0	1(1.5%)
Lipomatosis	1(0.2%)	1(0.9%)	1(1.0%)	0	0	0	0	0	0
Magnesium deficiency	1(0.2%)	0	0	0	0	1(0.4%)	1(0.5%)	0	0
Overweight	1(0.2%)	0	0	0	0	1(0.4%)	0	1(1.9%)	0
Neoplasms benign, malignant and unspecified (incl cysts and polyps)	181(41.8%)	43(37.4%)	37(36.3%)	2(33.3%)	4(57.1%)	104(41.4%)	85(42.9%)	19(35.8%)	34(50.7%)
Seborrhoeic keratosis	56(12.9%)	9(7.8%)	6(5.9%)	1(16.7%)	2(28.6%)	40(15.9%)	30(15.2%)	10(18.9%)	7(10.4%)
Squamous cell carcinoma	56(12.9%)	17(14.8%)	13(12.7%)	2(33.3%)	2(28.6%)	29(11.6%)	28(14.1%)	1(1.9%)	10(14.9%)
Basal cell carcinoma	27(6.2%)	6(5.2%)	6(5.9%)	0	0	16(6.4%)	13(6.6%)	3(5.7%)	5(7.5%)
Malignant melanoma	19(4.4%)	3(2.6%)	3(2.9%)	0	0	10(4.0%)	9(4.5%)	1(1.9%)	6(9.0%)
					Cohort 1: Newly Determined Advanced BCC Patients (Non-BCCNS) Metastatic BCC (mBCC)				
			Vismo (N=2)		No Vismo (N=1)				
	All (N=4)	All (N=2)	Vismo Only (N=2)	Surgery Only (N=0)	Other Therapy (N=0)	All (N=1)	Surgery Only (N=1)	Other Therapy (N=0)	No Treatment (N=1)

					Cohort 1: Newly Determined Advanced BCC Patients (Non-BCCNS) Locally Advanced BCC (laBCC)				
			Vismo (N=115)			No Vismo (N=251)			
	All (N=433)	All (N=115)	Vismo Only (N=102)	Surgery Only (N=6)	Other Therapy (N=7)	All (N=251)	Surgery Only (N=198)	Other Therapy (N=53)	No Treatment (N=67)
Metabolism and nutrition disorders (Cont'd)									
Glucose tolerance impaired	0	0	0	0	0	0	0	0	0
Hypercalcaemia	0	0	0	0	0	0	0	0	0
Hypomagnesaemia	0	0	0	0	0	0	0	0	0
Hyponatraemia	0	0	0	0	0	0	0	0	0
Impaired fasting glucose	0	0	0	0	0	0	0	0	0
Lactose intolerance	0	0	0	0	0	0	0	0	0
Lipomatosis	0	0	0	0	0	0	0	0	0
Magnesium deficiency	0	0	0	0	0	0	0	0	0
Overweight	0	0	0	0	0	0	0	0	0
Neoplasms benign, malignant and unspecified (incl cysts and polyps)	0	0	0	0	0	0	0	0	0
Seborrhoeic keratosis	0	0	0	0	0	0	0	0	0
Squamous cell carcinoma	0	0	0	0	0	0	0	0	0
Basal cell carcinoma	0	0	0	0	0	0	0	0	0
Malignant melanoma	0	0	0	0	0	0	0	0	0

Neoplasms benign, malignant and unspecified (incl cysts and polyps) (Cont'd)									
Prostate cancer	19(4.4%)	2(1.7%)	1(1.0%)	1(16.7%)	0	9(3.6%)	8(4.0%)	1(1.9%)	8(11.9%)
Melanocytic naevus	15(3.5%)	3(2.6%)	3(2.9%)	0	0	9(3.6%)	5(2.5%)	4(7.5%)	3(4.5%)
Breast cancer	10(2.3%)	2(1.7%)	1(1.0%)	0	1(14.3%)	8(3.2%)	8(4.0%)	0	0
Squamous cell carcinoma of skin	9(2.1%)	3(2.6%)	3(2.9%)	0	0	4(1.6%)	3(1.5%)	1(1.9%)	2(3.0%)
Haemangioma of skin	8(1.8%)	3(2.6%)	2(2.0%)	0	1(14.3%)	3(1.2%)	2(1.0%)	1(1.9%)	2(3.0%)
Colon cancer	6(1.4%)	2(1.7%)	2(2.0%)	0	0	3(1.2%)	0	3(5.7%)	1(1.5%)
Haemangioma	6(1.4%)	0	0	0	0	6(2.4%)	4(2.0%)	2(3.8%)	0
Acrochordon	4(0.9%)	0	0	0	0	4(1.6%)	4(2.0%)	0	0
Bladder cancer	3(0.7%)	0	0	0	0	2(0.8%)	2(1.0%)	0	1(1.5%)
Bowen's disease	3(0.7%)	0	0	0	0	3(1.2%)	3(1.5%)	0	0
Malignant melanoma in situ	3(0.7%)	1(0.9%)	1(1.0%)	0	0	2(0.8%)	2(1.0%)	0	0
Chronic lymphocytic leukaemia	2(0.5%)	0	0	0	0	1(0.4%)	1(0.5%)	0	1(1.5%)
Keratoacanthoma	2(0.5%)	0	0	0	0	2(0.8%)	1(0.5%)	1(1.9%)	0
Lung neoplasm malignant	2(0.5%)	0	0	0	0	1(0.4%)	1(0.5%)	0	1(1.5%)
					Cohort 1: Newly Determined Advanced BCC Patients (Non-BCCNS) Metastatic BCC (mBCC)				
			Vismo (N=2)		No Vismo (N=1)				
	All (N=4)	All (N=2)	Vismo Only (N=2)	Surgery Only (N=0)	Other Therapy (N=0)	All (N=1)	Surgery Only (N=1)	Other Therapy (N=0)	No Treatment (N=1)

Neoplasms benign, malignant and unspecified (incl cysts and polyps) (Cont'd)									
Prostate cancer	0	0	0	0	0	0	0	0	0
Melanocytic naevus	0	0	0	0	0	0	0	0	0
Breast cancer	0	0	0	0	0	0	0	0	0
Squamous cell carcinoma of skin	0	0	0	0	0	0	0	0	0
Haemangioma of skin	0	0	0	0	0	0	0	0	0
Colon cancer	0	0	0	0	0	0	0	0	0
Haemangioma	0	0	0	0	0	0	0	0	0
Acrochordon	0	0	0	0	0	0	0	0	0
Bladder cancer	0	0	0	0	0	0	0	0	0
Bowen's disease	0	0	0	0	0	0	0	0	0
Malignant melanoma in situ	0	0	0	0	0	0	0	0	0
Chronic lymphocytic leukaemia	0	0	0	0	0	0	0	0	0
Keratoacanthoma	0	0	0	0	0	0	0	0	0
Lung neoplasm malignant	0	0	0	0	0	0	0	0	0
					Cohort 1: Newly Determined Advanced BCC Patients (Non-BCCNS) Locally Advanced BCC (laBCC)				
		Vismo (N=115)			No Vismo (N=251)				
	All (N=433)	All (N=115)	Vismo Only (N=102)	Surgery Only (N=6)	Other Therapy (N=7)	All (N=251)	Surgery Only (N=198)	Other Therapy (N=53)	No Treatment (N=67)

Neoplasms benign, malignant and unspecified (incl cysts and polyps) (Cont'd)									
Skin cancer	2(0.5%)	0	0	0	0	2(0.8%)	2(1.0%)	0	0
Throat cancer	2(0.5%)	1(0.9%)	1(1.0%)	0	0	0	0	0	1(1.5%)
Acoustic neuroma	1(0.2%)	0	0	0	0	1(0.4%)	1(0.5%)	0	0
Atypical fibroxanth oma	1(0.2%)	1(0.9%)	1(1.0%)	0	0	0	0	0	0
Benign neoplasm of thyroid gland	1(0.2%)	1(0.9%)	1(1.0%)	0	0	0	0	0	0
Breast cancer metastatic	1(0.2%)	0	0	0	0	1(0.4%)	0	1(1.9%)	0
Carcinoid tumour of the small bowel	1(0.2%)	0	0	0	0	0	0	0	1(1.5%)
Cervix carcinoma	1(0.2%)	1(0.9%)	1(1.0%)	0	0	0	0	0	0
Dysplastic naevus	1(0.2%)	0	0	0	0	0	0	0	1(1.5%)
Ewing's sarcoma	1(0.2%)	1(0.9%)	1(1.0%)	0	0	0	0	0	0
Fibrous histiocyto ma	1(0.2%)	0	0	0	0	1(0.4%)	1(0.5%)	0	0
Hodgkin's disease	1(0.2%)	0	0	0	0	0	0	0	1(1.5%)
Lentigo maligna	1(0.2%)	1(0.9%)	1(1.0%)	0	0	0	0	0	0
Leukaemia	1(0.2%)	0	0	0	0	0	0	0	1(1.5%)
					Cohort 1: Newly Determined Advanced BCC Patients (Non-BCCNS) Metastatic BCC (mBCC)				
			Vismo (N=2)		No Vismo (N=1)				
	All (N=4)	All (N=2)	Vismo Only (N=2)	Surgery Only (N=0)	Other Therapy (N=0)	All (N=1)	Surgery Only (N=1)	Other Therapy (N=0)	No Treatment (N=1)

					Cohort 1: Newly Determined Advanced BCC Patients (Non-BCCNS) Locally Advanced BCC (laBCC)				
		Vismo (N=115)			No Vismo (N=251)				
	All (N=433)	All (N=115)	Vismo Only (N=102)	Surgery Only (N=6)	Other Therapy (N=7)	All (N=251)	Surgery Only (N=198)	Other Therapy (N=53)	No Treatment (N=67)
Neoplasms benign, malignant and unspecified (incl cysts and polyps) (Cont'd)									
Skin cancer	0	0	0	0	0	0	0	0	0
Throat cancer	0	0	0	0	0	0	0	0	0
Acoustic neuroma	0	0	0	0	0	0	0	0	0
Atypical fibroxanthoma	0	0	0	0	0	0	0	0	0
Benign neoplasm of thyroid gland	0	0	0	0	0	0	0	0	0
Breast cancer metastatic	0	0	0	0	0	0	0	0	0
Carcinoid tumour of the small bowel	0	0	0	0	0	0	0	0	0
Cervix carcinoma	0	0	0	0	0	0	0	0	0
Dysplastic naevus	0	0	0	0	0	0	0	0	0
Ewing's sarcoma	0	0	0	0	0	0	0	0	0
Fibrous histiocytoma	0	0	0	0	0	0	0	0	0
Hodgkin's disease	0	0	0	0	0	0	0	0	0
Lentigo maligna	0	0	0	0	0	0	0	0	0
Leukaemia	0	0	0	0	0	0	0	0	0

					Cohort 1: Newly Determined Advanced BCC Patients (Non-BCCNS) Metastatic BCC (mBCC)				
			Vismo (N=2)			No Vismo (N=1)			
	All (N=4)	All (N=2)	Vismo Only (N=2)	Surgery Only (N=0)	Other Therapy (N=0)	All (N=1)	Surgery Only (N=1)	Other Therapy (N=0)	No Treatment (N=1)
Neoplasms benign, malignant and unspecified (incl cysts and polyps) (Cont'd)									
Lip and/or oral cavity cancer	1(0.2%)	1(0.9%)	1(1.0%)	0	0	0	0	0	0
Lipoma	1(0.2%)	0	0	0	0	1(0.4%)	1(0.5%)	0	0
Lymphocytic leukaemia	1(0.2%)	1(0.9%)	1(1.0%)	0	0	0	0	0	0
Malignant neoplasm of eyelid	1(0.2%)	1(0.9%)	1(1.0%)	0	0	0	0	0	0
Meningioma	1(0.2%)	0	0	0	0	1(0.4%)	0	1(1.9%)	0
Metastatic malignant melanoma	1(0.2%)	0	0	0	0	0	0	0	1(1.5%)
Neoplasm malignant	1(0.2%)	1(0.9%)	1(1.0%)	0	0	0	0	0	0
Neuroectodermal neoplasm	1(0.2%)	0	0	0	0	1(0.4%)	1(0.5%)	0	0
Neuroendocrine carcinoma of the skin	1(0.2%)	1(0.9%)	1(1.0%)	0	0	0	0	0	0
Ovarian cancer	1(0.2%)	0	0	0	0	1(0.4%)	1(0.5%)	0	0
Pituitary tumour	1(0.2%)	0	0	0	0	1(0.4%)	1(0.5%)	0	0
Squamous cell carcinoma of the tongue	1(0.2%)	0	0	0	0	1(0.4%)	1(0.5%)	0	0

Neoplasms benign, malignant and unspecified (incl cysts and polyps) (Cont'd)									
Lip and/or oral cavity cancer	0	0	0	0	0	0	0	0	0
Lipoma	0	0	0	0	0	0	0	0	0
Lymphocytic leukaemia	0	0	0	0	0	0	0	0	0
Malignant neoplasm of eyelid	0	0	0	0	0	0	0	0	0
Meningioma	0	0	0	0	0	0	0	0	0
Metastatic malignant melanoma	0	0	0	0	0	0	0	0	0
Neoplasm malignant	0	0	0	0	0	0	0	0	0
Neuroectodermal neoplasm	0	0	0	0	0	0	0	0	0
Neuroendocrine carcinoma of the skin	0	0	0	0	0	0	0	0	0
Ovarian cancer	0	0	0	0	0	0	0	0	0
Pituitary tumour	0	0	0	0	0	0	0	0	0
Squamous cell carcinoma of the tongue	0	0	0	0	0	0	0	0	0
					Cohort 1: Newly Determined Advanced BCC Patients (Non-BCCNS) Locally Advanced BCC (laBCC)				
			Vismo (N=115)			No Vismo (N=251)			
	All (N=433)	All (N=115)	Vismo Only (N=102)	Surgery Only (N=6)	Other Therapy (N=7)	All (N=251)	Surgery Only (N=198)	Other Therapy (N=53)	No Treatment (N=67)

Neoplasms benign, malignant and unspecified (incl cysts and polyps) (Cont'd)									
Tongue neoplasm malignant stage unspecified	1(0.2%)	0	0	0	0	1(0.4%)	1(0.5%)	0	0
Uterine cancer	1(0.2%)	0	0	0	0	1(0.4%)	1(0.5%)	0	0
Uterine leiomyoma	1(0.2%)	0	0	0	0	1(0.4%)	1(0.5%)	0	0
Skin and subcutaneous tissue disorders	152(35.1%)	33(28.7%)	31(30.4%)	1(16.7%)	1(14.3%)	99(39.4%)	75(37.9%)	24(45.3%)	20(29.9%)
Actinic keratosis	102(23.6%)	14(12.2%)	13(12.7%)	1(16.7%)	0	72(28.7%)	52(26.3%)	20(37.7%)	16(23.9%)
Lentigo	27(6.2%)	2(1.7%)	2(2.0%)	0	0	23(9.2%)	18(9.1%)	5(9.4%)	2(3.0%)
Rosacea	14(3.2%)	2(1.7%)	1(1.0%)	0	1(14.3%)	9(3.6%)	5(2.5%)	4(7.5%)	3(4.5%)
Seborrhoeic dermatitis	10(2.3%)	1(0.9%)	1(1.0%)	0	0	7(2.8%)	4(2.0%)	3(5.7%)	2(3.0%)
Chloasma	8(1.8%)	0	0	0	0	7(2.8%)	5(2.5%)	2(3.8%)	1(1.5%)
Dermatitis contact	8(1.8%)	2(1.7%)	2(2.0%)	0	0	4(1.6%)	4(2.0%)	0	2(3.0%)
Pruritus	8(1.8%)	1(0.9%)	1(1.0%)	0	0	7(2.8%)	7(3.5%)	0	0
Purpura	8(1.8%)	0	0	0	0	6(2.4%)	3(1.5%)	3(5.7%)	2(3.0%)
Alopecia	6(1.4%)	4(3.5%)	4(3.9%)	0	0	2(0.8%)	1(0.5%)	1(1.9%)	0

					Cohort 1: Newly Determined Advanced BCC Patients (Non-BCCNS) Metastatic BCC (mBCC)				
			Vismo (N=2)		No Vismo (N=1)				
	All (N=4)	All (N=2)	Vismo Only (N=2)	Surgery Only (N=0)	Other Therapy (N=0)	All (N=1)	Surgery Only (N=1)	Other Therapy (N=0)	No Treatment (N=1)

Neoplasms benign, malignant and unspecified (incl cysts and polyps) (Cont'd)									
Tongue neoplasm malignant stage unspecified	0	0	0	0	0	0	0	0	0
Uterine cancer	0	0	0	0	0	0	0	0	0
Uterine leiomyoma	0	0	0	0	0	0	0	0	0
Skin and subcutaneous tissue disorders	1(25.0%)	0	0	0	0	0	0	0	1(100%)
Actinic keratosis	0	0	0	0	0	0	0	0	0
Lentigo	0	0	0	0	0	0	0	0	0
Rosacea	0	0	0	0	0	0	0	0	0
Seborrhoeic dermatitis	0	0	0	0	0	0	0	0	0
Chloasma	0	0	0	0	0	0	0	0	0
Dermatitis contact	0	0	0	0	0	0	0	0	0
Pruritus	0	0	0	0	0	0	0	0	0
Purpura	0	0	0	0	0	0	0	0	0
Alopecia	0	0	0	0	0	0	0	0	0

					Cohort 1: Newly Determined Advanced BCC Patients (Non-BCCNS) Locally Advanced BCC (laBCC)				
		Vismo (N=115)			No Vismo (N=251)				
	All (N=433)	All (N=115)	Vismo Only (N=102)	Surgery Only (N=6)	Other Therapy (N=7)	All (N=251)	Surgery Only (N=198)	Other Therapy (N=53)	No Treatment (N=67)
Skin and subcutaneous tissue disorders (Cont'd)									
Acne	5(1.2%)	1(0.9%)	1(1.0%)	0	0	4(1.6%)	4(2.0%)	0	0

Eczema	5(1.2%)	1(0.9%)	1(1.0%)	0	0	3(1.2%)	3(1.5%)	0	1(1.5%)
Photodermatosis	4(0.9%)	2(1.7%)	1(1.0%)	1(16.7%)	0	2(0.8%)	2(1.0%)	0	0
Psoriasis	4(0.9%)	1(0.9%)	1(1.0%)	0	0	1(0.4%)	1(0.5%)	0	2(3.0%)
Skin exfoliation	4(0.9%)	0	0	0	0	4(1.6%)	4(2.0%)	0	0
Skin ulcer	4(0.9%)	4(3.5%)	4(3.9%)	0	0	0	0	0	0
Androgenetic alopecia	3(0.7%)	3(2.6%)	3(2.9%)	0	0	0	0	0	0
Keloid scar	3(0.7%)	0	0	0	0	3(1.2%)	3(1.5%)	0	0
Rash	3(0.7%)	1(0.9%)	1(1.0%)	0	0	1(0.4%)	1(0.5%)	0	1(1.5%)
Sebaceous hyperplasia	3(0.7%)	0	0	0	0	3(1.2%)	1(0.5%)	2(3.8%)	0
Stasis dermatitis	3(0.7%)	1(0.9%)	1(1.0%)	0	0	2(0.8%)	1(0.5%)	1(1.9%)	0
Dermatitis	2(0.5%)	1(0.9%)	1(1.0%)	0	0	1(0.4%)	1(0.5%)	0	0
Dry skin	2(0.5%)	0	0	0	0	2(0.8%)	2(1.0%)	0	0
Lichenoid keratosis	2(0.5%)	1(0.9%)	1(1.0%)	0	0	0	0	0	1(1.5%)
Melanoderma	2(0.5%)	0	0	0	0	1(0.4%)	1(0.5%)	0	1(1.5%)
Solar lentigo	2(0.5%)	0	0	0	0	1(0.4%)	1(0.5%)	0	1(1.5%)

					Cohort 1: Newly Determined Advanced BCC Patients (Non-BCCNS) Metastatic BCC (mBCC)				
			Vismo (N=2)			No Vismo (N=1)			
	All (N=4)	All (N=2)	Vismo Only (N=2)	Surgery Only (N=0)	Other Therapy (N=0)	All (N=1)	Surgery Only (N=1)	Other Therapy (N=0)	No Treatment (N=1)
Skin and subcutaneous tissue disorders (Cont'd)									
Acne	0	0	0	0	0	0	0	0	0
Eczema	0	0	0	0	0	0	0	0	0
Photodermatosis	0	0	0	0	0	0	0	0	0
Psoriasis	0	0	0	0	0	0	0	0	0
Skin exfoliation	0	0	0	0	0	0	0	0	0
Skin ulcer	0	0	0	0	0	0	0	0	0
Androgenetic alopecia	0	0	0	0	0	0	0	0	0

Keloid scar	0	0	0	0	0	0	0	0	0
Rash	0	0	0	0	0	0	0	0	0
Sebaceous hyperplasia	0	0	0	0	0	0	0	0	0
Stasis dermatitis	0	0	0	0	0	0	0	0	0
Dermatitis	0	0	0	0	0	0	0	0	0
Dry skin	0	0	0	0	0	0	0	0	0
Lichenoid keratosis	0	0	0	0	0	0	0	0	0
Melanoderma	0	0	0	0	0	0	0	0	0
Solar lentigo	0	0	0	0	0	0	0	0	0

					Cohort 1: Newly Determined Advanced BCC Patients (Non-BCCNS) Locally Advanced BCC (laBCC)				
		Vismo (N=115)			No Vismo (N=251)				
	All (N=433)	All (N=115)	Vismo Only (N=102)	Surgery Only (N=6)	Other Therapy (N=7)	All (N=251)	Surgery Only (N=198)	Other Therapy (N=53)	No Treatment (N=67)
Skin and subcutaneous tissue disorders (Cont'd)									
Transient acantholytic dermatosis	2(0.5%)	1(0.9%)	1(1.0%)	0	0	0	0	0	1(1.5%)
Actinic elastosis	1(0.2%)	0	0	0	0	0	0	0	1(1.5%)
Blister	1(0.2%)	0	0	0	0	1(0.4%)	0	1(1.9%)	0
Decubitus ulcer	1(0.2%)	1(0.9%)	1(1.0%)	0	0	0	0	0	0
Dermal cyst	1(0.2%)	1(0.9%)	1(1.0%)	0	0	0	0	0	0
Dermatitis atopic	1(0.2%)	0	0	0	0	1(0.4%)	1(0.5%)	0	0
Dermatomyositis	1(0.2%)	0	0	0	0	1(0.4%)	0	1(1.9%)	0
Diabetic foot	1(0.2%)	1(0.9%)	1(1.0%)	0	0	0	0	0	0
Dyshidrotic eczema	1(0.2%)	0	0	0	0	1(0.4%)	0	1(1.9%)	0
Ecchymosis	1(0.2%)	0	0	0	0	1(0.4%)	1(0.5%)	0	0
Hyperkeratosis	1(0.2%)	1(0.9%)	1(1.0%)	0	0	0	0	0	0
Intertrigo	1(0.2%)	0	0	0	0	1(0.4%)	1(0.5%)	0	0

Keratosis pilaris	1(0.2%)	1(0.9%)	0	0	1(14.3%)	0	0	0	0
Poikiloderma	1(0.2%)	1(0.9%)	1(1.0%)	0	0	0	0	0	0
Rash maculo-papular	1(0.2%)	1(0.9%)	1(1.0%)	0	0	0	0	0	0
Skin erosion	1(0.2%)	1(0.9%)	1(1.0%)	0	0	0	0	0	0

					Cohort 1: Newly Determined Advanced BCC Patients (Non-BCCNS) Metastatic BCC (mBCC)				
		Vismo (N=2)			No Vismo (N=1)				
	All (N=4)	All (N=2)	Vismo Only (N=2)	Surgery Only (N=0)	Other Therapy (N=0)	All (N=1)	Surgery Only (N=1)	Other Therapy (N=0)	No Treatment (N=1)
Skin and subcutaneous tissue disorders (Cont'd)									
Transient acantholytic dermatosis	0	0	0	0	0	0	0	0	0
Actinic elastosis	0	0	0	0	0	0	0	0	0
Blister	0	0	0	0	0	0	0	0	0
Decubitus ulcer	0	0	0	0	0	0	0	0	0
Dermal cyst	0	0	0	0	0	0	0	0	0
Dermatitis atopic	0	0	0	0	0	0	0	0	0
Dermatomyositis	0	0	0	0	0	0	0	0	0
Diabetic foot	0	0	0	0	0	0	0	0	0
Dyshidrotic eczema	0	0	0	0	0	0	0	0	0
Ecchymosis	0	0	0	0	0	0	0	0	0
Hyperkeratosis	0	0	0	0	0	0	0	0	0
Intertrigo	0	0	0	0	0	0	0	0	0
Keratosis pilaris	0	0	0	0	0	0	0	0	0
Poikiloderma	0	0	0	0	0	0	0	0	0

Rash maculo-papular	0	0	0	0	0	0	0	0	0
Skin erosion	0	0	0	0	0	0	0	0	0
					Cohort 1: Newly Determined Advanced BCC Patients (Non-BCCNS) Locally Advanced BCC (laBCC)				
		Vismo (N=115)			No Vismo (N=251)				
	All (N=433)	All (N=115)	Vismo Only (N=102)	Surgery Only (N=6)	Other Therapy (N=7)	All (N=251)	Surgery Only (N=198)	Other Therapy (N=53)	No Treatment (N=67)
Skin and subcutaneous tissue disorders (Cont'd)									
Skin hyperpigmentation	1(0.2%)	0	0	0	0	1(0.4%)	0	1(1.9%)	0
Skin hypopigmentation	1(0.2%)	0	0	0	0	1(0.4%)	0	1(1.9%)	0
Skin irritation	1(0.2%)	0	0	0	0	0	0	0	1(1.5%)
Skin wrinkling	1(0.2%)	0	0	0	0	1(0.4%)	1(0.5%)	0	0
Spider naevus	1(0.2%)	0	0	0	0	1(0.4%)	1(0.5%)	0	0
Telangiectasia	0	0	0	0	0	0	0	0	0
Xeroderma	1(0.2%)	0	0	0	0	1(0.4%)	1(0.5%)	0	0
Musculoskeletal and connective tissue disorders	132(30.5%)	34(29.6%)	31(30.4%)	1(16.7%)	2(28.6%)	77(30.7%)	59(29.8%)	18(34.0%)	21(31.3%)
Arthritis	41(9.5%)	11(9.6%)	10(9.8%)	1(16.7%)	0	23(9.2%)	21(10.6%)	2(3.8%)	7(10.4%)
Osteoarthritis	33(7.6%)	6(5.2%)	5(4.9%)	0	1(14.3%)	23(9.2%)	12(6.1%)	11(20.8%)	4(6.0%)
Back pain	17(3.9%)	4(3.5%)	4(3.9%)	0	0	9(3.6%)	7(3.5%)	2(3.8%)	4(6.0%)
Osteoporosis	17(3.9%)	4(3.5%)	4(3.9%)	0	0	11(4.4%)	11(5.6%)	0	2(3.0%)
Muscle spasms	9(2.1%)	3(2.6%)	3(2.9%)	0	0	3(1.2%)	2(1.0%)	1(1.9%)	3(4.5%)
Rheumatoid arthritis	8(1.8%)	1(0.9%)	1(1.0%)	0	0	6(2.4%)	3(1.5%)	3(5.7%)	1(1.5%)

Arthralgia	6(1.4%)	3(2.6%)	2(2.0%)	0	1(14.3%)	2(0.8%)	2(1.0%)	0	1(1.5%)
					Cohort 1: Newly Determined Advanced BCC Patients (Non-BCCNS) Metastatic BCC (mBCC)				
			Vismo (N=2)			No Vismo (N=1)			
	All (N=4)	All (N=2)	Vismo Only (N=2)	Surgery Only (N=0)	Other Therapy (N=0)	All (N=1)	Surgery Only (N=1)	Other Therapy (N=0)	No Treatment (N=1)
Skin and subcutaneous tissue disorders (Cont'd)									
Skin hyperpigmentation	0	0	0	0	0	0	0	0	0
Skin hypopigmentation	0	0	0	0	0	0	0	0	0
Skin irritation	0	0	0	0	0	0	0	0	0
Skin wrinkling	0	0	0	0	0	0	0	0	0
Spider naevus	0	0	0	0	0	0	0	0	0
Telangiectasia	1(25.0%)	0	0	0	0	0	0	0	1(100%)
Xeroderma	0	0	0	0	0	0	0	0	0
Musculoskeletal and connective tissue disorders	2(50.0%)	1(50.0%)	1(50.0%)	0	0	0	0	0	1(100%)
Arthritis	0	0	0	0	0	0	0	0	0
Osteoarthritis	1(25.0%)	0	0	0	0	0	0	0	1(100%)
Back pain	0	0	0	0	0	0	0	0	0
Osteoporosis	0	0	0	0	0	0	0	0	0
Muscle spasms	0	0	0	0	0	0	0	0	0
Rheumatoid arthritis	0	0	0	0	0	0	0	0	0
Arthralgia	1(25.0%)	1(50.0%)	1(50.0%)	0	0	0	0	0	0
					Cohort 1: Newly Determined Advanced BCC Patients (Non-BCCNS) Locally Advanced BCC (laBCC)				
			Vismo (N=115)			No Vismo (N=251)			

	All (N=433)	All (N=115)	Vismo Only (N=102)	Surgery Only (N=6)	Other Therapy (N=7)	All (N=251)	Surgery Only (N=198)	Other Therapy (N=53)	No Treatment (N=67)
Musculoskeletal and connective tissue disorders (Cont'd)									
Osteopenia	7(1.6%)	0	0	0	0	4(1.6%)	4(2.0%)	0	3(4.5%)
Fibromyalgia	6(1.4%)	3(2.6%)	2(2.0%)	0	1(14.3%)	3(1.2%)	1(0.5%)	2(3.8%)	0
Intervertebral disc protrusion	5(1.2%)	2(1.7%)	2(2.0%)	0	0	2(0.8%)	2(1.0%)	0	1(1.5%)
Musculoskeletal pain	4(0.9%)	0	0	0	0	1(0.4%)	1(0.5%)	0	3(4.5%)
Neck pain	3(0.7%)	0	0	0	0	1(0.4%)	1(0.5%)	0	2(3.0%)
Intervertebral disc degeneration	2(0.5%)	0	0	0	0	1(0.4%)	1(0.5%)	0	1(1.5%)
Muscular weakness	2(0.5%)	1(0.9%)	1(1.0%)	0	0	0	0	0	1(1.5%)
Psoriatic arthropathy	2(0.5%)	1(0.9%)	1(1.0%)	0	0	1(0.4%)	1(0.5%)	0	0
Spinal osteoarthritis	2(0.5%)	0	0	0	0	1(0.4%)	1(0.5%)	0	1(1.5%)
Cervical spinal stenosis	1(0.2%)	0	0	0	0	1(0.4%)	0	1(1.9%)	0
Exostosis	1(0.2%)	0	0	0	0	1(0.4%)	1(0.5%)	0	0
Joint contracture	1(0.2%)	0	0	0	0	1(0.4%)	1(0.5%)	0	0
Joint swelling	1(0.2%)	1(0.9%)	1(1.0%)	0	0	0	0	0	0
Lumbar spinal stenosis	1(0.2%)	0	0	0	0	1(0.4%)	0	1(1.9%)	0
Muscle twitching	1(0.2%)	1(0.9%)	1(1.0%)	0	0	0	0	0	0

		Cohort 1: Newly Determined Advanced BCC Patients (Non-BCCNS) Metastatic BCC (mBCC)	
	Vismo (N=2)	No Vismo (N=1)	

	All (N=4)	All (N=2)	Vismo Only (N=2)	Surgery Only (N=0)	Other Therapy (N=0)	All (N=1)	Surgery Only (N=1)	Other Therapy (N=0)	No Treatment (N=1)
Musculoskeletal and connective tissue disorders (Cont'd)									
Osteopenia	0	0	0	0	0	0	0	0	0
Fibromyalgia	0	0	0	0	0	0	0	0	0
Intervertebral disc protrusion	0	0	0	0	0	0	0	0	0
Musculoskeletal pain	0	0	0	0	0	0	0	0	0
Neck pain	0	0	0	0	0	0	0	0	0
Intervertebral disc degeneration	0	0	0	0	0	0	0	0	0
Muscular weakness	0	0	0	0	0	0	0	0	0
Psoriatic arthropathy	0	0	0	0	0	0	0	0	0
Spinal osteoarthritis	0	0	0	0	0	0	0	0	0
Cervical spinal stenosis	0	0	0	0	0	0	0	0	0
Exostosis	0	0	0	0	0	0	0	0	0
Joint contracture	0	0	0	0	0	0	0	0	0
Joint swelling	0	0	0	0	0	0	0	0	0
Lumbar spinal stenosis	0	0	0	0	0	0	0	0	0
Muscle twitching	0	0	0	0	0	0	0	0	0

		Cohort 1: Newly Determined Advanced BCC Patients (Non-BCCNS) Locally Advanced BCC (laBCC)	
	Vismo (N=115)	No Vismo (N=251)	

	All (N=433)	All (N=115)	Vismo Only (N=102)	Surgery Only (N=6)	Other Therapy (N=7)	All (N=251)	Surgery Only (N=198)	Other Therapy (N=53)	No Treatment (N=67)
Musculoskeletal and connective tissue disorders (Cont'd)									
Musculoskeletal stiffness	1(0.2%)	1(0.9%)	1(1.0%)	0	0	0	0	0	0
Pain in extremity	1(0.2%)	1(0.9%)	1(1.0%)	0	0	0	0	0	0
Plantar fasciitis	1(0.2%)	1(0.9%)	0	0	1(14.3%)	0	0	0	0
Rheumatic fever	1(0.2%)	0	0	0	0	0	0	0	1(1.5%)
Rotator cuff syndrome	1(0.2%)	0	0	0	0	1(0.4%)	1(0.5%)	0	0
Scoliosis	1(0.2%)	0	0	0	0	1(0.4%)	1(0.5%)	0	0
Soft tissue necrosis	1(0.2%)	0	0	0	0	1(0.4%)	1(0.5%)	0	0
Spinal column stenosis	1(0.2%)	1(0.9%)	1(1.0%)	0	0	0	0	0	0
Spinal disorder	1(0.2%)	0	0	0	0	1(0.4%)	0	1(1.9%)	0
Spondylitis	1(0.2%)	0	0	0	0	1(0.4%)	1(0.5%)	0	0
Systemic lupus erythematosus	1(0.2%)	0	0	0	0	1(0.4%)	1(0.5%)	0	0
Temporomandibular joint syndrome	1(0.2%)	0	0	0	0	1(0.4%)	0	1(1.9%)	0
Gastrointestinal disorders	121(27.9%)	33(28.7%)	29(28.4%)	0	4(57.1%)	69(27.5%)	48(24.2%)	21(39.6%)	19(28.4%)
Gastrooesophageal reflux disease	79(18.2%)	17(14.8%)	15(14.7%)	0	2(28.6%)	47(18.7%)	30(15.2%)	17(32.1%)	15(22.4%)

					Cohort 1: Newly Determined Advanced BCC Patients (Non-BCCNS) Metastatic BCC (mBCC)				
		Vismo (N=2)			No Vismo (N=1)				
	All (N=4)	All (N=2)	Vismo Only (N=2)	Surgery Only (N=0)	Other Therapy (N=0)	All (N=1)	Surgery Only (N=1)	Other Therapy (N=0)	No Treatment (N=1)
Musculoskeletal and connective tissue disorders (Cont'd)									
Musculoskeletal stiffness	0	0	0	0	0	0	0	0	0
Pain in extremity	0	0	0	0	0	0	0	0	0
Plantar fasciitis	0	0	0	0	0	0	0	0	0
Rheumatic fever	0	0	0	0	0	0	0	0	0
Rotator cuff syndrome	0	0	0	0	0	0	0	0	0
Scoliosis	0	0	0	0	0	0	0	0	0
Soft tissue necrosis	0	0	0	0	0	0	0	0	0
Spinal column stenosis	0	0	0	0	0	0	0	0	0
Spinal disorder	0	0	0	0	0	0	0	0	0
Spondylitis	0	0	0	0	0	0	0	0	0
Systemic lupus erythematosus	0	0	0	0	0	0	0	0	0
Temporomandibular joint syndrome	0	0	0	0	0	0	0	0	0
Gastrointestinal disorders	2(50.0%)	1(50.0%)	1(50.0%)	0	0	0	0	0	1(100%)

Gastrooesophageal reflux disease	0	0	0	0	0	0	0	0	0
					Cohort 1: Newly Determined Advanced BCC Patients (Non-BCCNS) Locally Advanced BCC (laBCC)				
		Vismo (N=115)			No Vismo (N=251)				
	All (N=433)	All (N=115)	Vismo Only (N=102)	Surgery Only (N=6)	Other Therapy (N=7)	All (N=251)	Surgery Only (N=198)	Other Therapy (N=53)	No Treatment (N=67)
Gastrointestinal disorders (Cont'd)									
Constipation	16(3.7%)	6(5.2%)	6(5.9%)	0	0	8(3.2%)	8(4.0%)	0	2(3.0%)
Nausea	11(2.5%)	4(3.5%)	2(2.0%)	0	2(28.6%)	7(2.8%)	5(2.5%)	2(3.8%)	0
Dyschezia	6(1.4%)	3(2.6%)	0	0	3(42.9%)	3(1.2%)	0	3(5.7%)	0
Dyspepsia	6(1.4%)	3(2.6%)	3(2.9%)	0	0	3(1.2%)	3(1.5%)	0	0
Haemorrhoids	4(0.9%)	2(1.7%)	2(2.0%)	0	0	1(0.4%)	1(0.5%)	0	1(1.5%)
Hiatus hernia	4(0.9%)	2(1.7%)	0	0	2(28.6%)	1(0.4%)	1(0.5%)	0	1(1.5%)
Irritable bowel syndrome	4(0.9%)	0	0	0	0	4(1.6%)	2(1.0%)	2(3.8%)	0
Diarrhoea	2(0.5%)	0	0	0	0	1(0.4%)	1(0.5%)	0	1(1.5%)
Diverticulum	3(0.7%)	0	0	0	0	1(0.4%)	1(0.5%)	0	2(3.0%)
Large intestine polyp	3(0.7%)	1(0.9%)	1(1.0%)	0	0	2(0.8%)	1(0.5%)	1(1.9%)	0
Umbilical hernia	3(0.7%)	0	0	0	0	3(1.2%)	2(1.0%)	1(1.9%)	0
Abdominal hernia	2(0.5%)	0	0	0	0	2(0.8%)	2(1.0%)	0	0
Abdominal pain	2(0.5%)	0	0	0	0	2(0.8%)	1(0.5%)	1(1.9%)	0
Gastric ulcer	2(0.5%)	1(0.9%)	1(1.0%)	0	0	1(0.4%)	0	1(1.9%)	0
Gastritis	2(0.5%)	0	0	0	0	1(0.4%)	0	1(1.9%)	1(1.5%)
Pancreatitis	2(0.5%)	1(0.9%)	1(1.0%)	0	0	1(0.4%)	1(0.5%)	0	0
Barrett's oesophagus	1(0.2%)	0	0	0	0	1(0.4%)	0	1(1.9%)	0
					Cohort 1: Newly Determined Advanced BCC Patients (Non-BCCNS) Metastatic BCC (mBCC)				

		Vismo (N=2)			No Vismo (N=1)				
	All (N=4)	All (N=2)	Vismo Only (N=2)	Surgery Only (N=0)	Other Therapy (N=0)	All (N=1)	Surgery Only (N=1)	Other Therapy (N=0)	No Treatment (N=1)
Gastrointestinal disorders (Cont'd)									
Constipation	1(25.0%)	1(50.0%)	1(50.0%)	0	0	0	0	0	0
Nausea	0	0	0	0	0	0	0	0	0
Dyschezia	0	0	0	0	0	0	0	0	0
Dyspepsia	0	0	0	0	0	0	0	0	0
Haemorrhoids	0	0	0	0	0	0	0	0	0
Hiatus hernia	0	0	0	0	0	0	0	0	0
Irritable bowel syndrome	0	0	0	0	0	0	0	0	0
Diarrhoea	1(25.0%)	0	0	0	0	0	0	0	1(100%)
Diverticulum	0	0	0	0	0	0	0	0	0
Large intestine polyp	0	0	0	0	0	0	0	0	0
Umbilical hernia	0	0	0	0	0	0	0	0	0
Abdominal hernia	0	0	0	0	0	0	0	0	0
Abdominal pain	0	0	0	0	0	0	0	0	0
Gastric ulcer	0	0	0	0	0	0	0	0	0
Gastritis	0	0	0	0	0	0	0	0	0
Pancreatitis	0	0	0	0	0	0	0	0	0
Barrett's oesophagus	0	0	0	0	0	0	0	0	0
					Cohort 1: Newly Determined Advanced BCC Patients (Non-BCCNS) Locally Advanced BCC (laBCC)				
		Vismo (N=115)			No Vismo (N=251)				
	All (N=433)	All (N=115)	Vismo Only (N=102)	Surgery Only (N=6)	Other Therapy (N=7)	All (N=251)	Surgery Only (N=198)	Other Therapy (N=53)	No Treatment (N=67)

Gastrointestinal disorders (Cont'd)									
Colitis	1(0.2%)	1(0.9%)	1(1.0%)	0	0	0	0	0	0
Dry mouth	1(0.2%)	0	0	0	0	1(0.4%)	0	1(1.9%)	0
Duodenal ulcer	1(0.2%)	1(0.9%)	1(1.0%)	0	0	0	0	0	0
Dysphagia	1(0.2%)	0	0	0	0	1(0.4%)	0	1(1.9%)	0
Faeces hard	1(0.2%)	0	0	0	0	0	0	0	1(1.5%)
Gastric ulcer haemorrhage	1(0.2%)	0	0	0	0	1(0.4%)	0	1(1.9%)	0
Malpositioned teeth	1(0.2%)	1(0.9%)	1(1.0%)	0	0	0	0	0	0
Pancreatitis chronic	1(0.2%)	0	0	0	0	1(0.4%)	1(0.5%)	0	0
Peptic ulcer	1(0.2%)	0	0	0	0	1(0.4%)	1(0.5%)	0	0
Peptic ulcer haemorrhage	1(0.2%)	0	0	0	0	1(0.4%)	0	1(1.9%)	0
Poor dental condition	1(0.2%)	0	0	0	0	0	0	0	1(1.5%)
Rectal haemorrhage	0	0	0	0	0	0	0	0	0
Rectal polyp	1(0.2%)	0	0	0	0	1(0.4%)	0	1(1.9%)	0
Upper gastrointestinal haemorrhage	1(0.2%)	0	0	0	0	1(0.4%)	0	1(1.9%)	0

					Cohort 1: Newly Determined Advanced BCC Patients (Non-BCCNS) Metastatic BCC (mBCC)				
			Vismo (N=2)			No Vismo (N=1)			
	All (N=4)	All (N=2)	Vismo Only (N=2)	Surgery Only (N=0)	Other Therapy (N=0)	All (N=1)	Surgery Only (N=1)	Other Therapy (N=0)	No Treatment (N=1)
Gastrointestinal disorders (Cont'd)									
Colitis	0	0	0	0	0	0	0	0	0

Dry mouth	0	0	0	0	0	0	0	0	0
Duodenal ulcer	0	0	0	0	0	0	0	0	0
Dysphagia	0	0	0	0	0	0	0	0	0
Faeces hard	0	0	0	0	0	0	0	0	0
Gastric ulcer haemorrhage	0	0	0	0	0	0	0	0	0
Malpositioned teeth	0	0	0	0	0	0	0	0	0
Pancreatitis chronic	0	0	0	0	0	0	0	0	0
Peptic ulcer	0	0	0	0	0	0	0	0	0
Peptic ulcer haemorrhage	0	0	0	0	0	0	0	0	0
Poor dental condition	0	0	0	0	0	0	0	0	0
Rectal haemorrhage	1(25.0%)	0	0	0	0	0	0	0	1(100%)
Rectal polyp	0	0	0	0	0	0	0	0	0
Upper gastrointestinal haemorrhage	0	0	0	0	0	0	0	0	0

					Cohort 1: Newly Determined Advanced BCC Patients (Non-BCCNS) Locally Advanced BCC (laBCC)				
			Vismo (N=115)			No Vismo (N=251)			
	All (N=433)	All (N=115)	Vismo Only (N=102)	Surgery Only (N=6)	Other Therapy (N=7)	All (N=251)	Surgery Only (N=198)	Other Therapy (N=53)	No Treatment (N=67)
Psychiatric disorders	98(22.6%)	24(20.9%)	18(17.6%)	1(16.7%)	5(71.4%)	55(21.9%)	38(19.2%)	17(32.1%)	19(28.4%)
Depression	50(11.5%)	12(10.4%)	10(9.8%)	0	2(28.6%)	28(11.2%)	18(9.1%)	10(18.9%)	10(14.9%)
Anxiety	38(8.8%)	16(13.9%)	10(9.8%)	1(16.7%)	5(71.4%)	18(7.2%)	12(6.1%)	6(11.3%)	4(6.0%)
Insomnia	21(4.8%)	3(2.6%)	2(2.0%)	0	1(14.3%)	13(5.2%)	11(5.6%)	2(3.8%)	5(7.5%)
Bipolar disorder	5(1.2%)	1(0.9%)	1(1.0%)	0	0	3(1.2%)	2(1.0%)	1(1.9%)	1(1.5%)
Claustrophobia	2(0.5%)	1(0.9%)	1(1.0%)	0	0	1(0.4%)	1(0.5%)	0	0

Psychotic disorder	2(0.5%)	1(0.9%)	1(1.0%)	0	0	1(0.4%)	1(0.5%)	0	0
Alcohol abuse	1(0.2%)	0	0	0	0	0	0	0	1(1.5%)
Anxiety disorder	1(0.2%)	0	0	0	0	1(0.4%)	1(0.5%)	0	0
Bulimia nervosa	1(0.2%)	1(0.9%)	1(1.0%)	0	0	0	0	0	0
Drug use disorder	1(0.2%)	0	0	0	0	0	0	0	1(1.5%)
Generalised anxiety disorder	1(0.2%)	0	0	0	0	0	0	0	1(1.5%)
Major depression	1(0.2%)	0	0	0	0	0	0	0	1(1.5%)
Nicotine dependence	1(0.2%)	0	0	0	0	1(0.4%)	1(0.5%)	0	0
Post-traumatic stress disorder	1(0.2%)	0	0	0	0	0	0	0	1(1.5%)
Sleep disorder	1(0.2%)	0	0	0	0	1(0.4%)	1(0.5%)	0	0
Substance use disorder	1(0.2%)	1(0.9%)	1(1.0%)	0	0	0	0	0	0
Tobacco abuse	1(0.2%)	0	0	0	0	0	0	0	1(1.5%)

					Cohort 1: Newly Determined Advanced BCC Patients (Non-BCCNS) Metastatic BCC (mBCC)				
			Vismo (N=2)		No Vismo (N=1)				
	All (N=4)	All (N=2)	Vismo Only (N=2)	Surgery Only (N=0)	Other Therapy (N=0)	All (N=1)	Surgery Only (N=1)	Other Therapy (N=0)	No Treatment (N=1)
Psychiatric disorders	0	0	0	0	0	0	0	0	0
Depression	0	0	0	0	0	0	0	0	0
Anxiety	0	0	0	0	0	0	0	0	0
Insomnia	0	0	0	0	0	0	0	0	0
Bipolar disorder	0	0	0	0	0	0	0	0	0
Claustrophobia	0	0	0	0	0	0	0	0	0
Psychotic disorder	0	0	0	0	0	0	0	0	0
Alcohol abuse	0	0	0	0	0	0	0	0	0

Anxiety disorder	0	0	0	0	0	0	0	0	0
Bulimia nervosa	0	0	0	0	0	0	0	0	0
Drug use disorder	0	0	0	0	0	0	0	0	0
Generalised anxiety disorder	0	0	0	0	0	0	0	0	0
Major depression	0	0	0	0	0	0	0	0	0
Nicotine dependence	0	0	0	0	0	0	0	0	0
Post-traumatic stress disorder	0	0	0	0	0	0	0	0	0
Sleep disorder	0	0	0	0	0	0	0	0	0
Substance use disorder	0	0	0	0	0	0	0	0	0
Tobacco abuse	0	0	0	0	0	0	0	0	0
					Cohort 1: Newly Determined Advanced BCC Patients (Non-BCCNS) Locally Advanced BCC (laBCC)				
		Vismo (N=115)			No Vismo (N=251)				
	All (N=433)	All (N=115)	Vismo Only (N=102)	Surgery Only (N=6)	Other Therapy (N=7)	All (N=251)	Surgery Only (N=198)	Other Therapy (N=53)	No Treatment (N=67)
Social circumstances	94(21.7%)	24(20.9%)	20(19.6%)	0	4(57.1%)	54(21.5%)	41(20.7%)	13(24.5%)	16(23.9%)
Menopause	68(15.7%)	15(13.0%)	12(11.8%)	0	3(42.9%)	44(17.5%)	34(17.2%)	10(18.9%)	9(13.4%)
Postmenopause	15(3.5%)	7(6.1%)	6(5.9%)	0	1(14.3%)	6(2.4%)	4(2.0%)	2(3.8%)	2(3.0%)
Vascular device user	7(1.6%)	2(1.7%)	1(1.0%)	0	1(14.3%)	3(1.2%)	3(1.5%)	0	2(3.0%)
Cardiac assistance device user	2(0.5%)	0	0	0	0	1(0.4%)	0	1(1.9%)	1(1.5%)
Corrective lens user	2(0.5%)	0	0	0	0	0	0	0	2(3.0%)
Tobacco user	1(0.2%)	1(0.9%)	1(1.0%)	0	0	0	0	0	0

Cardiac disorders	90(20.8%)	21(18.3%)	15(14.7%)	0	6(85.7%)	47(18.7%)	35(17.7%)	12(22.6%)	22(32.8%)
Atrial fibrillation	22(5.1%)	3(2.6%)	2(2.0%)	0	1(14.3%)	14(5.6%)	13(6.6%)	1(1.9%)	5(7.5%)
Myocardial infarction	21(4.8%)	4(3.5%)	4(3.9%)	0	0	9(3.6%)	6(3.0%)	3(5.7%)	8(11.9%)
Coronary artery disease	20(4.6%)	8(7.0%)	4(3.9%)	0	4(57.1%)	8(3.2%)	5(2.5%)	3(5.7%)	4(6.0%)
Cardiac failure congestive	14(3.2%)	4(3.5%)	3(2.9%)	0	1(14.3%)	6(2.4%)	5(2.5%)	1(1.9%)	4(6.0%)
Cardiac disorder	9(2.1%)	1(0.9%)	1(1.0%)	0	0	7(2.8%)	3(1.5%)	4(7.5%)	1(1.5%)
Angina pectoris	8(1.8%)	1(0.9%)	1(1.0%)	0	0	5(2.0%)	4(2.0%)	1(1.9%)	2(3.0%)
Arrhythmia	6(1.4%)	0	0	0	0	3(1.2%)	3(1.5%)	0	3(4.5%)
Arteriosclerosis coronary artery	3(0.7%)	1(0.9%)	1(1.0%)	0	0	1(0.4%)	1(0.5%)	0	1(1.5%)

					Cohort 1: Newly Determined Advanced BCC Patients (Non-BCCNS) Metastatic BCC (mBCC)				
			Vismo (N=2)			No Vismo (N=1)			
	All (N=4)	All (N=2)	Vismo Only (N=2)	Surgery Only (N=0)	Other Therapy (N=0)	All (N=1)	Surgery Only (N=1)	Other Therapy (N=0)	No Treatment (N=1)
Social circumstances	0	0	0	0	0	0	0	0	0
Menopause	0	0	0	0	0	0	0	0	0
Postmenopause	0	0	0	0	0	0	0	0	0
Vascular device user	0	0	0	0	0	0	0	0	0
Cardiac assistance device user	0	0	0	0	0	0	0	0	0
Corrective lens user	0	0	0	0	0	0	0	0	0
Tobacco user	0	0	0	0	0	0	0	0	0

Cardiac disorders	1(25.0%)	0	0	0	0	0	0	0	1(100%)
Atrial fibrillation	0	0	0	0	0	0	0	0	0
Myocardial infarction	1(25.0%)	0	0	0	0	0	0	0	1(100%)
Coronary artery disease	0	0	0	0	0	0	0	0	0
Cardiac failure congestive	0	0	0	0	0	0	0	0	0
Cardiac disorder	0	0	0	0	0	0	0	0	0
Angina pectoris	0	0	0	0	0	0	0	0	0
Arrhythmia	0	0	0	0	0	0	0	0	0
Arteriosclerosis coronary artery	1(25.0%)	0	0	0	0	0	0	0	1(100%)

					Cohort 1: Newly Determined Advanced BCC Patients (Non-BCCNS) Locally Advanced BCC (laBCC)				
			Vismo (N=115)			No Vismo (N=251)			
	All (N=433)	All (N=115)	Vismo Only (N=102)	Surgery Only (N=6)	Other Therapy (N=7)	All (N=251)	Surgery Only (N=198)	Other Therapy (N=53)	No Treatment (N=67)
Cardiac disorders (Cont'd)									
Aortic valve stenosis	2(0.5%)	2(1.7%)	1(1.0%)	0	1(14.3%)	0	0	0	0
Cardiac valve disease	2(0.5%)	0	0	0	0	2(0.8%)	2(1.0%)	0	0
Cardiomyopathy	2(0.5%)	0	0	0	0	2(0.8%)	2(1.0%)	0	0
Mitral valve prolapse	2(0.5%)	0	0	0	0	2(0.8%)	2(1.0%)	0	0
Acute myocardial infarction	1(0.2%)	0	0	0	0	1(0.4%)	1(0.5%)	0	0

Bundle branch block left	1(0.2%)	0	0	0	0	1(0.4%)	1(0.5%)	0	0
Cardiovascular disorder	1(0.2%)	0	0	0	0	1(0.4%)	0	1(1.9%)	0
Hypertensive heart disease	1(0.2%)	0	0	0	0	1(0.4%)	1(0.5%)	0	0
Ischaemic cardiomyopathy	1(0.2%)	1(0.9%)	1(1.0%)	0	0	0	0	0	0
Left ventricular hypertrophy	1(0.2%)	1(0.9%)	1(1.0%)	0	0	0	0	0	0
Mitral valve disease	1(0.2%)	1(0.9%)	0	0	1(14.3%)	0	0	0	0
Palpitations	1(0.2%)	0	0	0	0	0	0	0	1(1.5%)
Pericarditis	1(0.2%)	0	0	0	0	1(0.4%)	1(0.5%)	0	0
Stress cardiomyopathy	1(0.2%)	0	0	0	0	1(0.4%)	1(0.5%)	0	0
Supraventricular tachycardia	1(0.2%)	0	0	0	0	0	0	0	1(1.5%)
Tachycardia	1(0.2%)	1(0.9%)	1(1.0%)	0	0	0	0	0	0
Ventricular tachycardia	1(0.2%)	0	0	0	0	1(0.4%)	1(0.5%)	0	0

					Cohort 1: Newly Determined Advanced BCC Patients (Non-BCCNS) Metastatic BCC (mBCC)				
			Vismo (N=2)		No Vismo (N=1)				
	All (N=4)	All (N=2)	Vismo Only (N=2)	Surgery Only (N=0)	Other Therapy (N=0)	All (N=1)	Surgery Only (N=1)	Other Therapy (N=0)	No Treatment (N=1)
Cardiac disorders (Cont'd)									
Aortic valve stenosis	0	0	0	0	0	0	0	0	0

Cardiac valve disease	0	0	0	0	0	0	0	0	0
Cardiomyopathy	0	0	0	0	0	0	0	0	0
Mitral valve prolapse	0	0	0	0	0	0	0	0	0
Acute myocardial infarction	0	0	0	0	0	0	0	0	0
Bundle branch block left	0	0	0	0	0	0	0	0	0
Cardiovascular disorder	0	0	0	0	0	0	0	0	0
Hypertensive heart disease	0	0	0	0	0	0	0	0	0
Ischaemic cardiomyopathy	0	0	0	0	0	0	0	0	0
Left ventricular hypertrophy	0	0	0	0	0	0	0	0	0
Mitral valve disease	0	0	0	0	0	0	0	0	0
Palpitations	0	0	0	0	0	0	0	0	0
Pericarditis	0	0	0	0	0	0	0	0	0
Stress cardiomyopathy	0	0	0	0	0	0	0	0	0
Supraventricular tachycardia	0	0	0	0	0	0	0	0	0
Tachycardia	0	0	0	0	0	0	0	0	0
Ventricular tachycardia	0	0	0	0	0	0	0	0	0

Cohort 1: Newly Determined Advanced BCC Patients (Non-BCCNS) Locally Advanced BCC (laBCC)

		Vismo (N=115)				No Vismo (N=251)			
	All (N=433)	All (N=115)	Vismo Only (N=102)	Surgery Only (N=6)	Other Therapy (N=7)	All (N=251)	Surgery Only (N=198)	Other Therapy (N=53)	No Treatment (N=67)
Nervous system disorders	88(20.3%)	27(23.5%)	20(19.6%)	1(16.7%)	6(85.7%)	46(18.3%)	30(15.2%)	16(30.2%)	15(22.4%)
Cerebrovascular accident	16(3.7%)	4(3.5%)	2(2.0%)	1(16.7%)	1(14.3%)	6(2.4%)	4(2.0%)	2(3.8%)	6(9.0%)
Neuropathy peripheral	14(3.2%)	3(2.6%)	1(1.0%)	0	2(28.6%)	10(4.0%)	5(2.5%)	5(9.4%)	1(1.5%)
Migraine	10(2.3%)	6(5.2%)	4(3.9%)	0	2(28.6%)	4(1.6%)	3(1.5%)	1(1.9%)	0
Dementia Alzheimer's type	8(1.8%)	3(2.6%)	1(1.0%)	0	2(28.6%)	4(1.6%)	1(0.5%)	3(5.7%)	1(1.5%)
Headache	8(1.8%)	3(2.6%)	3(2.9%)	0	0	2(0.8%)	2(1.0%)	0	3(4.5%)
Transient ischaemic attack	8(1.8%)	1(0.9%)	1(1.0%)	0	0	3(1.2%)	2(1.0%)	1(1.9%)	4(6.0%)
Dementia	7(1.6%)	3(2.6%)	1(1.0%)	0	2(28.6%)	4(1.6%)	2(1.0%)	2(3.8%)	0
Parkinson's disease	6(1.4%)	0	0	0	0	5(2.0%)	2(1.0%)	3(5.7%)	1(1.5%)
Carpal tunnel syndrome	4(0.9%)	1(0.9%)	0	0	1(14.3%)	3(1.2%)	3(1.5%)	0	0
Dizziness	4(0.9%)	4(3.5%)	2(2.0%)	0	2(28.6%)	0	0	0	0
Dysgeusia	4(0.9%)	3(2.6%)	3(2.9%)	0	0	1(0.4%)	0	1(1.9%)	0
Neuralgia	4(0.9%)	1(0.9%)	1(1.0%)	0	0	3(1.2%)	3(1.5%)	0	0
Seizure	4(0.9%)	1(0.9%)	1(1.0%)	0	0	2(0.8%)	1(0.5%)	1(1.9%)	1(1.5%)
Diabetic neuropathy	3(0.7%)	2(1.7%)	1(1.0%)	0	1(14.3%)	1(0.4%)	1(0.5%)	0	0
Epilepsy	3(0.7%)	0	0	0	0	3(1.2%)	2(1.0%)	1(1.9%)	0
Polyneuropathy	3(0.7%)	0	0	0	0	3(1.2%)	3(1.5%)	0	0
Restless legs syndrome	3(0.7%)	0	0	0	0	3(1.2%)	3(1.5%)	0	0
Syncope	3(0.7%)	1(0.9%)	1(1.0%)	0	0	0	0	0	2(3.0%)

					Cohort 1: Newly Determined Advanced BCC Patients (Non-BCCNS) Metastatic BCC (mBCC)				
		Vismo (N=2)				No Vismo (N=1)			
	All (N=4)	All (N=2)	Vismo Only (N=2)	Surgery Only (N=0)	Other Therapy (N=0)	All (N=1)	Surgery Only (N=1)	Other Therapy (N=0)	No Treatment (N=1)

Nervous system disorders	1(25.0%)	0	0	0	0	0	0	0	1(100%)
Cerebrovascular accident	0	0	0	0	0	0	0	0	0
Neuropathy peripheral	0	0	0	0	0	0	0	0	0
Migraine	0	0	0	0	0	0	0	0	0
Dementia Alzheimer's type	0	0	0	0	0	0	0	0	0
Headache	0	0	0	0	0	0	0	0	0
Transient ischaemic attack	0	0	0	0	0	0	0	0	0
Dementia	0	0	0	0	0	0	0	0	0
Parkinson's disease	0	0	0	0	0	0	0	0	0
Carpal tunnel syndrome	1(25.0%)	0	0	0	0	0	0	0	1(100%)
Dizziness	0	0	0	0	0	0	0	0	0
Dysgeusia	0	0	0	0	0	0	0	0	0
Neuralgia	0	0	0	0	0	0	0	0	0
Seizure	0	0	0	0	0	0	0	0	0
Diabetic neuropathy	0	0	0	0	0	0	0	0	0
Epilepsy	0	0	0	0	0	0	0	0	0
Polyneuropathy	0	0	0	0	0	0	0	0	0
Restless legs syndrome	0	0	0	0	0	0	0	0	0
Syncope	0	0	0	0	0	0	0	0	0
					Cohort 1: Newly Determined Advanced BCC Patients (Non-BCCNS) Locally Advanced BCC (laBCC)				
			Vismo (N=115)			No Vismo (N=251)			
	All (N=433)	All (N=115)	Vismo Only (N=102)	Surgery Only (N=6)	Other Therapy (N=7)	All (N=251)	Surgery Only (N=198)	Other Therapy (N=53)	No Treatment (N=67)
Nervous system disorders (Cont'd)									
Tremor	3(0.7%)	2(1.7%)	2(2.0%)	0	0	1(0.4%)	1(0.5%)	0	0

Carotid artery stenosis	2(0.5%)	1(0.9%)	1(1.0%)	0	0	0	0	0	1(1.5%)
Hemiparesis	2(0.5%)	1(0.9%)	1(1.0%)	0	0	1(0.4%)	1(0.5%)	0	0
Autonomic nervous system imbalance	1(0.2%)	0	0	0	0	1(0.4%)	1(0.5%)	0	0
Carotid artery disease	1(0.2%)	0	0	0	0	1(0.4%)	0	1(1.9%)	0
Carotid artery occlusion	1(0.2%)	0	0	0	0	0	0	0	1(1.5%)
Cerebral haemorrhage	1(0.2%)	0	0	0	0	0	0	0	1(1.5%)
Cervical myelopathy	1(0.2%)	0	0	0	0	1(0.4%)	0	1(1.9%)	0
Encephalopathy	1(0.2%)	0	0	0	0	1(0.4%)	1(0.5%)	0	0
Facial paralysis	0	0	0	0	0	0	0	0	0
Hypoaesthesia	1(0.2%)	1(0.9%)	1(1.0%)	0	0	0	0	0	0
Hypogeusia	1(0.2%)	1(0.9%)	1(1.0%)	0	0	0	0	0	0
Intracranial aneurysm	1(0.2%)	0	0	0	0	1(0.4%)	1(0.5%)	0	0
Multiple sclerosis	1(0.2%)	0	0	0	0	0	0	0	1(1.5%)
Parkinsonism	1(0.2%)	0	0	0	0	1(0.4%)	0	1(1.9%)	0
Radiculopathy	1(0.2%)	0	0	0	0	0	0	0	1(1.5%)

					Cohort 1: Newly Determined Advanced BCC Patients (Non-BCCNS) Metastatic BCC (mBCC)				
			Vismo (N=2)		No Vismo (N=1)				
	All (N=4)	All (N=2)	Vismo Only (N=2)	Surgery Only (N=0)	Other Therapy (N=0)	All (N=1)	Surgery Only (N=1)	Other Therapy (N=0)	No Treatment (N=1)
Nervous system disorders (Cont'd)									
Tremor	0	0	0	0	0	0	0	0	0

Carotid artery stenosis	0	0	0	0	0	0	0	0	0
Hemiparesis	0	0	0	0	0	0	0	0	0
Autonomic nervous system imbalance	0	0	0	0	0	0	0	0	0
Carotid artery disease	0	0	0	0	0	0	0	0	0
Carotid artery occlusion	0	0	0	0	0	0	0	0	0
Cerebral haemorrhage	0	0	0	0	0	0	0	0	0
Cervical myelopathy	0	0	0	0	0	0	0	0	0
Encephalopathy	0	0	0	0	0	0	0	0	0
Facial paralysis	1(25.0%)	0	0	0	0	0	0	0	1(100%)
Hypoaesthesia	0	0	0	0	0	0	0	0	0
Hypogeusia	0	0	0	0	0	0	0	0	0
Intracranial aneurysm	0	0	0	0	0	0	0	0	0
Multiple sclerosis	0	0	0	0	0	0	0	0	0
Parkinsonism	0	0	0	0	0	0	0	0	0
Radiculopathy	0	0	0	0	0	0	0	0	0

					Cohort 1: Newly Determined Advanced BCC Patients (Non-BCCNS) Locally Advanced BCC (laBCC)				
		Vismo (N=115)			No Vismo (N=251)				
	All (N=433)	All (N=115)	Vismo Only (N=102)	Surgery Only (N=6)	Other Therapy (N=7)	All (N=251)	Surgery Only (N=198)	Other Therapy (N=53)	No Treatment (N=67)
Nervous system disorders (Cont'd)									
Sinus headache	1(0.2%)	0	0	0	0	0	0	0	1(1.5%)

Tension headache	1(0.2%)	0	0	0	0	1(0.4%)	1(0.5%)	0	0
Trigeminal neuralgia	1(0.2%)	0	0	0	0	1(0.4%)	1(0.5%)	0	0
Upper motor neurone lesion	1(0.2%)	1(0.9%)	1(1.0%)	0	0	0	0	0	0
Infections and infestations	70(16.2%)	21(18.3%)	18(17.6%)	1(16.7%)	2(28.6%)	37(14.7%)	27(13.6%)	10(18.9%)	12(17.9%)
Hepatitis C	9(2.1%)	2(1.7%)	2(2.0%)	0	0	5(2.0%)	4(2.0%)	1(1.9%)	2(3.0%)
Pneumonia	9(2.1%)	3(2.6%)	3(2.9%)	0	0	5(2.0%)	5(2.5%)	0	1(1.5%)
Cellulitis	5(1.2%)	0	0	0	0	4(1.6%)	1(0.5%)	3(5.7%)	1(1.5%)
Osteomyelitis	4(0.9%)	0	0	0	0	4(1.6%)	3(1.5%)	1(1.9%)	0
Sinusitis	4(0.9%)	1(0.9%)	0	0	1(14.3%)	2(0.8%)	2(1.0%)	0	1(1.5%)
Staphylococcal infection	4(0.9%)	2(1.7%)	2(2.0%)	0	0	2(0.8%)	1(0.5%)	1(1.9%)	0
Infection	2(0.5%)	1(0.9%)	1(1.0%)	0	0	1(0.4%)	1(0.5%)	0	0
Measles	3(0.7%)	3(2.6%)	3(2.9%)	0	0	0	0	0	0
Mumps	3(0.7%)	3(2.6%)	3(2.9%)	0	0	0	0	0	0
Onychomycosis	3(0.7%)	1(0.9%)	1(1.0%)	0	0	1(0.4%)	1(0.5%)	0	1(1.5%)
Rhinitis	3(0.7%)	1(0.9%)	1(1.0%)	0	0	2(0.8%)	2(1.0%)	0	0

					Cohort 1: Newly Determined Advanced BCC Patients (Non-BCCNS) Metastatic BCC (mBCC)				
			Vismo (N=2)		No Vismo (N=1)				
	All (N=4)	All (N=2)	Vismo Only (N=2)	Surgery Only (N=0)	Other Therapy (N=0)	All (N=1)	Surgery Only (N=1)	Other Therapy (N=0)	No Treatment (N=1)
Nervous system disorders (Cont'd)									
Sinus headache	0	0	0	0	0	0	0	0	0
Tension headache	0	0	0	0	0	0	0	0	0
Trigeminal neuralgia	0	0	0	0	0	0	0	0	0
Upper motor neurone lesion	0	0	0	0	0	0	0	0	0

Infections and infestations	2(50.0%)	1(50.0%)	1(50.0%)	0	0	1(100%)	1(100%)	0	0
Hepatitis C	0	0	0	0	0	0	0	0	0
Pneumonia	0	0	0	0	0	0	0	0	0
Cellulitis	1(25.0%)	1(50.0%)	1(50.0%)	0	0	0	0	0	0
Osteomyelitis	0	0	0	0	0	0	0	0	0
Sinusitis	0	0	0	0	0	0	0	0	0
Staphylococcal infection	0	0	0	0	0	0	0	0	0
Infection	1(25.0%)	0	0	0	0	1(100%)	1(100%)	0	0
Measles	0	0	0	0	0	0	0	0	0
Mumps	0	0	0	0	0	0	0	0	0
Onychomycosis	0	0	0	0	0	0	0	0	0
Rhinitis	0	0	0	0	0	0	0	0	0
					Cohort 1: Newly Determined Advanced BCC Patients (Non-BCCNS) Locally Advanced BCC (laBCC)				
			Vismo (N=115)			No Vismo (N=251)			
	All (N=433)	All (N=115)	Vismo Only (N=102)	Surgery Only (N=6)	Other Therapy (N=7)	All (N=251)	Surgery Only (N=198)	Other Therapy (N=53)	No Treatment (N=67)
Infections and infestations (Cont'd)									
Urinary tract infection	3(0.7%)	0	0	0	0	3(1.2%)	2(1.0%)	1(1.9%)	0
Bacterial infection	2(0.5%)	1(0.9%)	1(1.0%)	0	0	1(0.4%)	1(0.5%)	0	0
Bronchitis	2(0.5%)	0	0	0	0	1(0.4%)	1(0.5%)	0	1(1.5%)
Conjunctivitis	2(0.5%)	1(0.9%)	1(1.0%)	0	0	1(0.4%)	0	1(1.9%)	0
Diverticulitis	2(0.5%)	0	0	0	0	1(0.4%)	0	1(1.9%)	1(1.5%)
Fungal infection	2(0.5%)	2(1.7%)	1(1.0%)	0	1(14.3%)	0	0	0	0
Poliomyelitis	2(0.5%)	0	0	0	0	0	0	0	2(3.0%)
Varicella	2(0.5%)	0	0	0	0	2(0.8%)	2(1.0%)	0	0
Appendicitis	1(0.2%)	1(0.9%)	1(1.0%)	0	0	0	0	0	0

Chronic hepatitis C	1(0.2%)	1(0.9%)	1(1.0%)	0	0	0	0	0	0
Chronic sinusitis	1(0.2%)	1(0.9%)	1(1.0%)	0	0	0	0	0	0
Clostridium difficile colitis	1(0.2%)	0	0	0	0	1(0.4%)	1(0.5%)	0	0
Coccidioidomycosis	1(0.2%)	0	0	0	0	1(0.4%)	1(0.5%)	0	0
Cytomegalovirus infection	1(0.2%)	0	0	0	0	1(0.4%)	0	1(1.9%)	0
Dermatophytosis of nail	1(0.2%)	0	0	0	0	1(0.4%)	1(0.5%)	0	0
Endocarditis bacterial	1(0.2%)	0	0	0	0	0	0	0	1(1.5%)

					Cohort 1: Newly Determined Advanced BCC Patients (Non-BCCNS) Metastatic BCC (mBCC)				
			Vismo (N=2)			No Vismo (N=1)			
	All (N=4)	All (N=2)	Vismo Only (N=2)	Surgery Only (N=0)	Other Therapy (N=0)	All (N=1)	Surgery Only (N=1)	Other Therapy (N=0)	No Treatment (N=1)
Infections and infestations (Cont'd)									
Urinary tract infection	0	0	0	0	0	0	0	0	0
Bacterial infection	0	0	0	0	0	0	0	0	0
Bronchitis	0	0	0	0	0	0	0	0	0
Conjunctivitis	0	0	0	0	0	0	0	0	0
Diverticulitis	0	0	0	0	0	0	0	0	0
Fungal infection	0	0	0	0	0	0	0	0	0
Poliomyelitis	0	0	0	0	0	0	0	0	0
Varicella	0	0	0	0	0	0	0	0	0
Appendicitis	0	0	0	0	0	0	0	0	0

Chronic hepatitis C	0	0	0	0	0	0	0	0	0
Chronic sinusitis	0	0	0	0	0	0	0	0	0
Clostridium difficile colitis	0	0	0	0	0	0	0	0	0
Coccidioidomycosis	0	0	0	0	0	0	0	0	0
Cytomegalovirus infection	0	0	0	0	0	0	0	0	0
Dermatophytosis of nail	0	0	0	0	0	0	0	0	0
Endocarditis bacterial	0	0	0	0	0	0	0	0	0
					Cohort 1: Newly Determined Advanced BCC Patients (Non-BCCNS) Locally Advanced BCC (laBCC)				
			Vismo (N=115)			No Vismo (N=251)			
	All (N=433)	All (N=115)	Vismo Only (N=102)	Surgery Only (N=6)	Other Therapy (N=7)	All (N=251)	Surgery Only (N=198)	Other Therapy (N=53)	No Treatment (N=67)
Infections and infestations (Cont'd)									
Epstein-Barr virus infection	1(0.2%)	0	0	0	0	1(0.4%)	0	1(1.9%)	0
Extradural abscess	1(0.2%)	0	0	0	0	1(0.4%)	0	1(1.9%)	0
Folliculitis	1(0.2%)	0	0	0	0	1(0.4%)	0	1(1.9%)	0
Fungal skin infection	1(0.2%)	1(0.9%)	1(1.0%)	0	0	0	0	0	0
Gangrene	1(0.2%)	1(0.9%)	0	0	1(14.3%)	0	0	0	0
Gastrointestinal candidiasis	1(0.2%)	0	0	0	0	1(0.4%)	0	1(1.9%)	0
Genital herpes	1(0.2%)	1(0.9%)	1(1.0%)	0	0	0	0	0	0
HIV infection	1(0.2%)	1(0.9%)	1(1.0%)	0	0	0	0	0	0

Hepatitis A	1(0.2%)	0	0	0	0	0	0	0	1(1.5%)
Herpes simplex	1(0.2%)	0	0	0	0	1(0.4%)	0	1(1.9%)	0
Herpes zoster	1(0.2%)	0	0	0	0	1(0.4%)	1(0.5%)	0	0
Impetigo	1(0.2%)	1(0.9%)	1(1.0%)	0	0	0	0	0	0
Mastitis	1(0.2%)	0	0	0	0	1(0.4%)	1(0.5%)	0	0
Mastoiditis	1(0.2%)	0	0	0	0	1(0.4%)	1(0.5%)	0	0
Oral candidiasis	1(0.2%)	1(0.9%)	1(1.0%)	0	0	0	0	0	0
Oral herpes	1(0.2%)	0	0	0	0	0	0	0	1(1.5%)
Otitis media	1(0.2%)	0	0	0	0	1(0.4%)	1(0.5%)	0	0

	Cohort 1: Newly Determined Advanced BCC Patients (Non-BCCNS) Metastatic BCC (mBCC)								
		Vismo (N=2)			No Vismo (N=1)				
	All (N=4)	All (N=2)	Vismo Only (N=2)	Surgery Only (N=0)	Other Therapy (N=0)	All (N=1)	Surgery Only (N=1)	Other Therapy (N=0)	No Treatment (N=1)
Infections and infestations (Cont'd)									
Epstein-Barr virus infection	0	0	0	0	0	0	0	0	0
Extradural abscess	0	0	0	0	0	0	0	0	0
Folliculitis	0	0	0	0	0	0	0	0	0
Fungal skin infection	0	0	0	0	0	0	0	0	0
Gangrene	0	0	0	0	0	0	0	0	0
Gastrointestinal candidiasis	0	0	0	0	0	0	0	0	0
Genital herpes	0	0	0	0	0	0	0	0	0
HIV infection	0	0	0	0	0	0	0	0	0
Hepatitis A	0	0	0	0	0	0	0	0	0
Herpes simplex	0	0	0	0	0	0	0	0	0
Herpes zoster	0	0	0	0	0	0	0	0	0

Impetigo	0	0	0	0	0	0	0	0	0
Mastitis	0	0	0	0	0	0	0	0	0
Mastoiditis	0	0	0	0	0	0	0	0	0
Oral candidiasis	0	0	0	0	0	0	0	0	0
Oral herpes	0	0	0	0	0	0	0	0	0
Otitis media	0	0	0	0	0	0	0	0	0
					Cohort 1: Newly Determined Advanced BCC Patients (Non-BCCNS) Locally Advanced BCC (laBCC)				
		Vismo (N=115)			No Vismo (N=251)				
	All (N=433)	All (N=115)	Vismo Only (N=102)	Surgery Only (N=6)	Other Therapy (N=7)	All (N=251)	Surgery Only (N=198)	Other Therapy (N=53)	No Treatment (N=67)
Infections and infestations (Cont'd)									
Papilloma viral infection	1(0.2%)	0	0	0	0	1(0.4%)	1(0.5%)	0	0
Pertussis	1(0.2%)	1(0.9%)	1(1.0%)	0	0	0	0	0	0
Pilonidal cyst	1(0.2%)	1(0.9%)	0	1(16.7%)	0	0	0	0	0
Post procedural sepsis	1(0.2%)	0	0	0	0	0	0	0	1(1.5%)
Sepsis	1(0.2%)	0	0	0	0	1(0.4%)	1(0.5%)	0	0
Sialoadenitis	1(0.2%)	0	0	0	0	1(0.4%)	1(0.5%)	0	0
Syphilis	1(0.2%)	0	0	0	0	0	0	0	1(1.5%)
Tuberculosis	1(0.2%)	1(0.9%)	1(1.0%)	0	0	0	0	0	0
Respiratory, thoracic and mediastinal disorders	71(16.4%)	18(15.7%)	16(15.7%)	1(16.7%)	1(14.3%)	40(15.9%)	31(15.7%)	9(17.0%)	13(19.4%)
Asthma	26(6.0%)	7(6.1%)	5(4.9%)	1(16.7%)	1(14.3%)	15(6.0%)	12(6.1%)	3(5.7%)	4(6.0%)
Chronic obstructive pulmonary disease	20(4.6%)	6(5.2%)	6(5.9%)	0	0	10(4.0%)	7(3.5%)	3(5.7%)	4(6.0%)

Sleep apnoea syndrome	10(2.3%)	2(1.7%)	2(2.0%)	0	0	6(2.4%)	4(2.0%)	2(3.8%)	2(3.0%)
Emphysema	7(1.6%)	2(1.7%)	2(2.0%)	0	0	4(1.6%)	4(2.0%)	0	1(1.5%)
Rhinitis allergic	5(1.2%)	1(0.9%)	1(1.0%)	0	0	4(1.6%)	3(1.5%)	1(1.9%)	0

					Cohort 1: Newly Determined Advanced BCC Patients (Non-BCCNS) Metastatic BCC (mBCC)				
			Vismo (N=2)			No Vismo (N=1)			
	All (N=4)	All (N=2)	Vismo Only (N=2)	Surgery Only (N=0)	Other Therapy (N=0)	All (N=1)	Surgery Only (N=1)	Other Therapy (N=0)	No Treatment (N=1)
Infections and infestations (Cont'd)									
Papilloma viral infection	0	0	0	0	0	0	0	0	0
Pertussis	0	0	0	0	0	0	0	0	0
Pilonidal cyst	0	0	0	0	0	0	0	0	0
Post procedural sepsis	0	0	0	0	0	0	0	0	0
Sepsis	0	0	0	0	0	0	0	0	0
Sialoadenitis	0	0	0	0	0	0	0	0	0
Syphilis	0	0	0	0	0	0	0	0	0
Tuberculosis	0	0	0	0	0	0	0	0	0
Respiratory, thoracic and mediastinal disorders	1(25.0%)	0	0	0	0	0	0	0	1(100%)
Asthma	0	0	0	0	0	0	0	0	0
Chronic obstructive pulmonary disease	0	0	0	0	0	0	0	0	0
Sleep apnoea syndrome	0	0	0	0	0	0	0	0	0
Emphysema	0	0	0	0	0	0	0	0	0

Rhinitis allergic	1(25.0%)	0	0	0	0	0	0	0	1(100%)
					Cohort 1: Newly Determined Advanced BCC Patients (Non-BCCNS) Locally Advanced BCC (laBCC)				
		Vismo (N=115)			No Vismo (N=251)				
	All (N=433)	All (N=115)	Vismo Only (N=102)	Surgery Only (N=6)	Other Therapy (N=7)	All (N=251)	Surgery Only (N=198)	Other Therapy (N=53)	No Treatment (N=67)
Respiratory, thoracic and mediastinal disorders (Cont'd)									
Dyspnoea	5(1.2%)	1(0.9%)	1(1.0%)	0	0	1(0.4%)	1(0.5%)	0	3(4.5%)
Bronchitis chronic	3(0.7%)	1(0.9%)	1(1.0%)	0	0	2(0.8%)	1(0.5%)	1(1.9%)	0
Cough	1(0.2%)	0	0	0	0	1(0.4%)	1(0.5%)	0	0
Hypoxia	2(0.5%)	1(0.9%)	1(1.0%)	0	0	1(0.4%)	0	1(1.9%)	0
Pulmonary embolism	2(0.5%)	0	0	0	0	2(0.8%)	1(0.5%)	1(1.9%)	0
Wheezing	2(0.5%)	1(0.9%)	0	0	1(14.3%)	0	0	0	1(1.5%)
Bronchiectasis	1(0.2%)	0	0	0	0	1(0.4%)	1(0.5%)	0	0
Nasal congestion	1(0.2%)	0	0	0	0	1(0.4%)	1(0.5%)	0	0
Nasal disorder	1(0.2%)	1(0.9%)	1(1.0%)	0	0	0	0	0	0
Nasal septum deviation	1(0.2%)	0	0	0	0	1(0.4%)	1(0.5%)	0	0
Paranasal sinus hypersecretion	1(0.2%)	0	0	0	0	0	0	0	1(1.5%)
Pulmonary arterial hypertension	1(0.2%)	1(0.9%)	1(1.0%)	0	0	0	0	0	0
Pulmonary hypertension	1(0.2%)	0	0	0	0	1(0.4%)	0	1(1.9%)	0
Pulmonary mass	1(0.2%)	0	0	0	0	1(0.4%)	1(0.5%)	0	0
Respiratory failure	1(0.2%)	0	0	0	0	1(0.4%)	1(0.5%)	0	0
					Cohort 1: Newly Determined Advanced BCC Patients (Non-BCCNS) Metastatic BCC (mBCC)				

		Vismo (N=2)			No Vismo (N=1)			
	All (N=4)	All (N=2)	Vismo Only (N=2)	Surgery Only (N=0)	Other Therapy (N=0)	All (N=1)	Surgery Only (N=1)	Other Therapy (N=0)	No Treatment (N=1)
Respiratory, thoracic and mediastinal disorders (Cont'd)									
Dyspnoea	0	0	0	0	0	0	0	0	0
Bronchitis chronic	0	0	0	0	0	0	0	0	0
Cough	1(25.0%)	0	0	0	0	0	0	0	1(100%)
Hypoxia	0	0	0	0	0	0	0	0	0
Pulmonary embolism	0	0	0	0	0	0	0	0	0
Wheezing	0	0	0	0	0	0	0	0	0
Bronchiectasis	0	0	0	0	0	0	0	0	0
Nasal congestion	0	0	0	0	0	0	0	0	0
Nasal disorder	0	0	0	0	0	0	0	0	0
Nasal septum deviation	0	0	0	0	0	0	0	0	0
Paranasal sinus hypersecretion	0	0	0	0	0	0	0	0	0
Pulmonary arterial hypertension	0	0	0	0	0	0	0	0	0
Pulmonary hypertension	0	0	0	0	0	0	0	0	0
Pulmonary mass	0	0	0	0	0	0	0	0	0
Respiratory failure	0	0	0	0	0	0	0	0	0

					Cohort 1: Newly Determined Advanced BCC Patients (Non-BCCNS) Locally Advanced BCC (laBCC)				
		Vismo (N=115)			No Vismo (N=251)				
	All (N=433)	All (N=115)	Vismo Only (N=102)	Surgery Only (N=6)	Other Therapy (N=7)	All (N=251)	Surgery Only (N=198)	Other Therapy (N=53)	No Treatment (N=67)

					Cohort 1: Newly Determined Advanced BCC Patients (Non-BCCNS) Metastatic BCC (mBCC)				
			Vismo (N=2)		No Vismo (N=1)				
	All (N=4)	All (N=2)	Vismo Only (N=2)	Surgery Only (N=0)	Other Therapy (N=0)	All (N=1)	Surgery Only (N=1)	Other Therapy (N=0)	No Treatment (N=1)
Respiratory, thoracic and mediastinal disorders (Cont'd)									
Sinus congestion	1(0.2%)	0	0	0	0	0	0	0	1(1.5%)
Investigations	71(16.4%)	22(19.1%)	19(18.6%)	2(33.3%)	1(14.3%)	37(14.7%)	25(12.6%)	12(22.6%)	12(17.9%)
Blood cholesterol increased	21(4.8%)	6(5.2%)	5(4.9%)	1(16.7%)	0	11(4.4%)	7(3.5%)	4(7.5%)	4(6.0%)
Biopsy skin	11(2.5%)	5(4.3%)	4(3.9%)	1(16.7%)	0	4(1.6%)	2(1.0%)	2(3.8%)	2(3.0%)
Biopsy	7(1.6%)	3(2.6%)	2(2.0%)	0	1(14.3%)	4(1.6%)	4(2.0%)	0	0
Cardiac murmur	7(1.6%)	3(2.6%)	3(2.9%)	0	0	3(1.2%)	2(1.0%)	1(1.9%)	1(1.5%)
Colonoscopy	6(1.4%)	2(1.7%)	1(1.0%)	0	1(14.3%)	4(1.6%)	2(1.0%)	2(3.8%)	0
Arthroscopy	4(0.9%)	3(2.6%)	3(2.9%)	0	0	1(0.4%)	1(0.5%)	0	0
Catheterisation cardiac	4(0.9%)	2(1.7%)	1(1.0%)	0	1(14.3%)	2(0.8%)	0	2(3.8%)	0
Blood triglycerides increased	3(0.7%)	0	0	0	0	2(0.8%)	2(1.0%)	0	1(1.5%)
Biopsy breast	2(0.5%)	1(0.9%)	1(1.0%)	0	0	1(0.4%)	1(0.5%)	0	0
Biopsy ear	2(0.5%)	0	0	0	0	2(0.8%)	2(1.0%)	0	0
Endoscopy	2(0.5%)	0	0	0	0	2(0.8%)	1(0.5%)	1(1.9%)	0
Oesophagogastroduodenoscopy	2(0.5%)	1(0.9%)	0	0	1(14.3%)	1(0.4%)	0	1(1.9%)	0
Weight decreased	2(0.5%)	0	0	0	0	2(0.8%)	0	2(3.8%)	0

Respiratory, thoracic and mediastinal disorders (Cont'd)									
Sinus congestion	0	0	0	0	0	0	0	0	0
Investigations	0	0	0	0	0	0	0	0	0
Blood cholesterol increased	0	0	0	0	0	0	0	0	0
Biopsy skin	0	0	0	0	0	0	0	0	0
Biopsy	0	0	0	0	0	0	0	0	0
Cardiac murmur	0	0	0	0	0	0	0	0	0
Colonoscopy	0	0	0	0	0	0	0	0	0
Arthroscopy	0	0	0	0	0	0	0	0	0
Catheterisation cardiac	0	0	0	0	0	0	0	0	0
Blood triglycerides increased	0	0	0	0	0	0	0	0	0
Biopsy breast	0	0	0	0	0	0	0	0	0
Biopsy ear	0	0	0	0	0	0	0	0	0
Endoscopy	0	0	0	0	0	0	0	0	0
Oesophagogastroduodenoscopy	0	0	0	0	0	0	0	0	0
Weight decreased	0	0	0	0	0	0	0	0	0

					Cohort 1: Newly Determined Advanced BCC Patients (Non-BCCNS) Locally Advanced BCC (laBCC)				
			Vismo (N=115)		No Vismo (N=251)				
	All (N=433)	All (N=115)	Vismo Only (N=102)	Surgery Only (N=6)	Other Therapy (N=7)	All (N=251)	Surgery Only (N=198)	Other Therapy (N=53)	No Treatment (N=67)
Investigations (Cont'd)									
Antinuclear antibody positive	1(0.2%)	0	0	0	0	0	0	0	1(1.5%)

					Cohort 1: Newly Determined Advanced BCC Patients (Non-BCCNS) Metastatic BCC (mBCC)				
		Vismo (N=2)			No Vismo (N=1)				
	All (N=4)	All (N=2)	Vismo Only (N=2)	Surgery Only (N=0)	Other Therapy (N=0)	All (N=1)	Surgery Only (N=1)	Other Therapy (N=0)	No Treatment (N=1)
Investigations (Cont'd)									
Aortic bruit	1(0.2%)	0	0	0	0	1(0.4%)	1(0.5%)	0	0
Biopsy abdominal wall	1(0.2%)	1(0.9%)	0	0	1(14.3%)	0	0	0	0
Biopsy lip	1(0.2%)	0	0	0	0	1(0.4%)	1(0.5%)	0	0
Biopsy prostate	1(0.2%)	0	0	0	0	0	0	0	1(1.5%)
Blood calcium decreased	1(0.2%)	0	0	0	0	1(0.4%)	1(0.5%)	0	0
Blood glucose increased	1(0.2%)	0	0	0	0	0	0	0	1(1.5%)
Blood parathyroid hormone abnormal	1(0.2%)	0	0	0	0	1(0.4%)	1(0.5%)	0	0
Blood testosterone decreased	1(0.2%)	0	0	0	0	1(0.4%)	1(0.5%)	0	0
Endoscopic retrograde cholangiopancreatography	1(0.2%)	0	0	0	0	1(0.4%)	0	1(1.9%)	0
Heart rate irregular	1(0.2%)	0	0	0	0	0	0	0	1(1.5%)
Hepatic enzyme increased	1(0.2%)	1(0.9%)	1(1.0%)	0	0	0	0	0	0
Laparoscopy	1(0.2%)	0	0	0	0	0	0	0	1(1.5%)
Staphylococcus test	1(0.2%)	0	0	0	0	0	0	0	1(1.5%)
Staphylococcus test positive	1(0.2%)	0	0	0	0	1(0.4%)	1(0.5%)	0	0

Antinuclear antibody positive	0	0	0	0	0	0	0	0	0
Aortic bruit	0	0	0	0	0	0	0	0	0
Biopsy abdominal wall	0	0	0	0	0	0	0	0	0
Biopsy lip	0	0	0	0	0	0	0	0	0
Biopsy prostate	0	0	0	0	0	0	0	0	0
Blood calcium decreased	0	0	0	0	0	0	0	0	0
Blood glucose increased	0	0	0	0	0	0	0	0	0
Blood parathyroid hormone abnormal	0	0	0	0	0	0	0	0	0
Blood testosterone decreased	0	0	0	0	0	0	0	0	0
Endoscopic retrograde cholangiopancreatography	0	0	0	0	0	0	0	0	0
Heart rate irregular	0	0	0	0	0	0	0	0	0
Hepatic enzyme increased	0	0	0	0	0	0	0	0	0
Laparoscopy	0	0	0	0	0	0	0	0	0
Staphylococcus test	0	0	0	0	0	0	0	0	0
Staphylococcus test positive	0	0	0	0	0	0	0	0	0
					Cohort 1: Newly Determined Advanced BCC Patients (Non-BCCNS) Locally Advanced BCC (laBCC)				
		Vismo (N=115)			No Vismo (N=251)				
	All (N=433)	All (N=115)	Vismo Only (N=102)	Surgery Only (N=6)	Other Therapy (N=7)	All (N=251)	Surgery Only (N=198)	Other Therapy (N=53)	No Treatment (N=67)

Investigations (Cont'd)									
Ureteroscopy normal	1(0.2%)	0	0	0	0	1(0.4%)	0	1(1.9%)	0
Eye disorders	67(15.5%)	24(20.9%)	23(22.5%)	0	1(14.3%)	35(13.9%)	32(16.2%)	3(5.7%)	8(11.9%)
Glaucoma	16(3.7%)	7(6.1%)	7(6.9%)	0	0	8(3.2%)	5(2.5%)	3(5.7%)	1(1.5%)
Cataract	15(3.5%)	7(6.1%)	6(5.9%)	0	1(14.3%)	7(2.8%)	6(3.0%)	1(1.9%)	1(1.5%)
Myopia	15(3.5%)	2(1.7%)	2(2.0%)	0	0	12(4.8%)	12(6.1%)	0	1(1.5%)
Dry eye	7(1.6%)	3(2.6%)	3(2.9%)	0	0	3(1.2%)	3(1.5%)	0	1(1.5%)
Macular degeneration	7(1.6%)	1(0.9%)	1(1.0%)	0	0	5(2.0%)	5(2.5%)	0	1(1.5%)
Presbyopia	5(1.2%)	2(1.7%)	2(2.0%)	0	0	3(1.2%)	3(1.5%)	0	0
Hypermetropia	3(0.7%)	0	0	0	0	3(1.2%)	3(1.5%)	0	0
Blindness unilateral	2(0.5%)	0	0	0	0	1(0.4%)	1(0.5%)	0	1(1.5%)
Dacryostenosis acquired	2(0.5%)	1(0.9%)	1(1.0%)	0	0	0	0	0	1(1.5%)
Ectropion	1(0.2%)	1(0.9%)	1(1.0%)	0	0	0	0	0	0
Lacrimation increased	2(0.5%)	1(0.9%)	1(1.0%)	0	0	0	0	0	1(1.5%)
Retinal tear	2(0.5%)	1(0.9%)	0	0	1(14.3%)	1(0.4%)	1(0.5%)	0	0
Visual impairment	2(0.5%)	2(1.7%)	2(2.0%)	0	0	0	0	0	0
Blepharitis	1(0.2%)	1(0.9%)	1(1.0%)	0	0	0	0	0	0
Blepharospasm	1(0.2%)	1(0.9%)	1(1.0%)	0	0	0	0	0	0

					Cohort 1: Newly Determined Advanced BCC Patients (Non-BCCNS) Metastatic BCC (mBCC)				
			Vismo (N=2)			No Vismo (N=1)			
	All (N=4)	All (N=2)	Vismo Only (N=2)	Surgery Only (N=0)	Other Therapy (N=0)	All (N=1)	Surgery Only (N=1)	Other Therapy (N=0)	No Treatment (N=1)
Investigations (Cont'd)									
Ureteroscopy normal	0	0	0	0	0	0	0	0	0
Eye disorders	1(25.0%)	0	0	0	0	0	0	0	1(100%)
Glaucoma	0	0	0	0	0	0	0	0	0
Cataract	0	0	0	0	0	0	0	0	0
Myopia	0	0	0	0	0	0	0	0	0

Dry eye	0	0	0	0	0	0	0	0	0
Macular degeneration	0	0	0	0	0	0	0	0	0
Presbyopia	0	0	0	0	0	0	0	0	0
Hypermetropia	0	0	0	0	0	0	0	0	0
Blindness unilateral	0	0	0	0	0	0	0	0	0
Dacryostenosis acquired	0	0	0	0	0	0	0	0	0
Ectropion	1(25.0%)	0	0	0	0	0	0	0	1(100%)
Lacrimation increased	0	0	0	0	0	0	0	0	0
Retinal tear	0	0	0	0	0	0	0	0	0
Visual impairment	0	0	0	0	0	0	0	0	0
Blepharitis	0	0	0	0	0	0	0	0	0
Blepharospasm	0	0	0	0	0	0	0	0	0
					Cohort 1: Newly Determined Advanced BCC Patients (Non-BCCNS) Locally Advanced BCC (laBCC)				
			Vismo (N=115)			No Vismo (N=251)			
	All (N=433)	All (N=115)	Vismo Only (N=102)	Surgery Only (N=6)	Other Therapy (N=7)	All (N=251)	Surgery Only (N=198)	Other Therapy (N=53)	No Treatment (N=67)
Eye disorders (Cont'd)									
Blindness	1(0.2%)	1(0.9%)	1(1.0%)	0	0	0	0	0	0
Borderline glaucoma	1(0.2%)	0	0	0	0	1(0.4%)	1(0.5%)	0	0
Conjunctival hyperaemia	1(0.2%)	1(0.9%)	1(1.0%)	0	0	0	0	0	0
Eye inflammation	1(0.2%)	0	0	0	0	1(0.4%)	1(0.5%)	0	0
Eye irritation	1(0.2%)	0	0	0	0	0	0	0	1(1.5%)
Eyelid ptosis	1(0.2%)	1(0.9%)	1(1.0%)	0	0	0	0	0	0
Ocular hypertension	1(0.2%)	0	0	0	0	1(0.4%)	1(0.5%)	0	0

Punctate keratitis	1(0.2%)	0	0	0	0	1(0.4%)	0	1(1.9%)	0
General disorders and administration site conditions	61(14.1%)	16(13.9%)	13(12.7%)	1(16.7%)	2(28.6%)	34(13.5%)	20(10.1%)	14(26.4%)	11(16.4%)
Pain	23(5.3%)	7(6.1%)	7(6.9%)	0	0	12(4.8%)	8(4.0%)	4(7.5%)	4(6.0%)
Xerosis	17(3.9%)	2(1.7%)	0	1(16.7%)	1(14.3%)	12(4.8%)	8(4.0%)	4(7.5%)	3(4.5%)
Oedema	8(1.8%)	1(0.9%)	0	0	1(14.3%)	6(2.4%)	1(0.5%)	5(9.4%)	1(1.5%)
Cyst	5(1.2%)	0	0	0	0	5(2.0%)	3(1.5%)	2(3.8%)	0
Unevaluable event	5(1.2%)	0	0	0	0	4(1.6%)	4(2.0%)	0	1(1.5%)
Chest pain	4(0.9%)	0	0	0	0	2(0.8%)	1(0.5%)	1(1.9%)	2(3.0%)
Fatigue	3(0.7%)	2(1.7%)	2(2.0%)	0	0	1(0.4%)	0	1(1.9%)	0

						Cohort 1: Newly Determined Advanced BCC Patients (Non-BCCNS) Metastatic BCC (mBCC)			
		Vismo (N=2)				No Vismo (N=1)			
	All (N=4)	All (N=2)	Vismo Only (N=2)	Surgery Only (N=0)	Other Therapy (N=0)	All (N=1)	Surgery Only (N=1)	Other Therapy (N=0)	No Treatment (N=1)
Eye disorders (Cont'd)									
Blindness	0	0	0	0	0	0	0	0	0
Borderline glaucoma	0	0	0	0	0	0	0	0	0
Conjunctival hyperaemia	0	0	0	0	0	0	0	0	0
Eye inflammation	0	0	0	0	0	0	0	0	0
Eye irritation	0	0	0	0	0	0	0	0	0
Eyelid ptosis	0	0	0	0	0	0	0	0	0
Ocular hypertension	0	0	0	0	0	0	0	0	0
Punctate keratitis	0	0	0	0	0	0	0	0	0

General disorders and administration site conditions	1(25.0%)	1(50.0%)	1(50.0%)	0	0	0	0	0	0
Pain	1(25.0%)	1(50.0%)	1(50.0%)	0	0	0	0	0	0
Xerosis	0	0	0	0	0	0	0	0	0
Oedema	0	0	0	0	0	0	0	0	0
Cyst	0	0	0	0	0	0	0	0	0
Unevaluable event	0	0	0	0	0	0	0	0	0
Chest pain	0	0	0	0	0	0	0	0	0
Fatigue	0	0	0	0	0	0	0	0	0

					Cohort 1: Newly Determined Advanced BCC Patients (Non-BCCNS) Locally Advanced BCC (laBCC)				
			Vismo (N=115)			No Vismo (N=251)			
	All (N=433)	All (N=115)	Vismo Only (N=102)	Surgery Only (N=6)	Other Therapy (N=7)	All (N=251)	Surgery Only (N=198)	Other Therapy (N=53)	No Treatment (N=67)
General disorders and administration site conditions (Cont'd)									
Facial pain	2(0.5%)	2(1.7%)	2(2.0%)	0	0	0	0	0	0
Impaired healing	2(0.5%)	1(0.9%)	1(1.0%)	0	0	1(0.4%)	0	1(1.9%)	0
Oedema peripheral	2(0.5%)	0	0	0	0	1(0.4%)	1(0.5%)	0	1(1.5%)
Chronic fatigue syndrome	1(0.2%)	0	0	0	0	0	0	0	1(1.5%)
Gait disturbance	1(0.2%)	0	0	0	0	0	0	0	1(1.5%)
Hernia	1(0.2%)	1(0.9%)	1(1.0%)	0	0	0	0	0	0
Ill-defined disorder	1(0.2%)	0	0	0	0	1(0.4%)	1(0.5%)	0	0
Inflammation	1(0.2%)	1(0.9%)	1(1.0%)	0	0	0	0	0	0
Ulcer	1(0.2%)	1(0.9%)	1(1.0%)	0	0	0	0	0	0
Immune system disorders	59(13.6%)	12(10.4%)	12(11.8%)	0	0	40(15.9%)	32(16.2%)	8(15.1%)	7(10.4%)

Seasonal allergy	29(6.7%)	5(4.3%)	5(4.9%)	0	0	21(8.4%)	19(9.6%)	2(3.8%)	3(4.5%)
Drug hypersensitivity	24(5.5%)	6(5.2%)	6(5.9%)	0	0	16(6.4%)	11(5.6%)	5(9.4%)	2(3.0%)
Hypersensitivity	11(2.5%)	3(2.6%)	3(2.9%)	0	0	7(2.8%)	6(3.0%)	1(1.9%)	1(1.5%)
Allergy to chemicals	1(0.2%)	0	0	0	0	1(0.4%)	0	1(1.9%)	0
					Cohort 1: Newly Determined Advanced BCC Patients (Non-BCCNS) Metastatic BCC (mBCC)				
			Vismo (N=2)		No Vismo (N=1)				
	All (N=4)	All (N=2)	Vismo Only (N=2)	Surgery Only (N=0)	Other Therapy (N=0)	All (N=1)	Surgery Only (N=1)	Other Therapy (N=0)	No Treatment (N=1)
General disorders and administration site conditions (Cont'd)									
Facial pain	0	0	0	0	0	0	0	0	0
Impaired healing	0	0	0	0	0	0	0	0	0
Oedema peripheral	0	0	0	0	0	0	0	0	0
Chronic fatigue syndrome	0	0	0	0	0	0	0	0	0
Gait disturbance	0	0	0	0	0	0	0	0	0
Hernia	0	0	0	0	0	0	0	0	0
Ill-defined disorder	0	0	0	0	0	0	0	0	0
Inflammation	0	0	0	0	0	0	0	0	0
Ulcer	0	0	0	0	0	0	0	0	0
Immune system disorders	0	0	0	0	0	0	0	0	0
Seasonal allergy	0	0	0	0	0	0	0	0	0
Drug hypersensitivity	0	0	0	0	0	0	0	0	0

Hypersensitivity	0	0	0	0	0	0	0	0	0
Allergy to chemicals	0	0	0	0	0	0	0	0	0
					Cohort 1: Newly Determined Advanced BCC Patients (Non-BCCNS) Locally Advanced BCC (laBCC)				
		Vismo (N=115)			No Vismo (N=251)				
	All (N=433)	All (N=115)	Vismo Only (N=102)	Surgery Only (N=6)	Other Therapy (N=7)	All (N=251)	Surgery Only (N=198)	Other Therapy (N=53)	No Treatment (N=67)
Immune system disorders (Cont'd)									
Allergy to fermented products	1(0.2%)	0	0	0	0	1(0.4%)	0	1(1.9%)	0
Allergy to plants	1(0.2%)	0	0	0	0	0	0	0	1(1.5%)
Anaphylactic reaction	1(0.2%)	0	0	0	0	0	0	0	1(1.5%)
Contrast media allergy	1(0.2%)	0	0	0	0	1(0.4%)	0	1(1.9%)	0
Endocrine disorders	58(13.4%)	20(17.4%)	17(16.7%)	0	3(42.9%)	34(13.5%)	26(13.1%)	8(15.1%)	4(6.0%)
Hypothyroidism	52(12.0%)	19(16.5%)	16(15.7%)	0	3(42.9%)	29(11.6%)	22(11.1%)	7(13.2%)	4(6.0%)
Hyperthyroidism	2(0.5%)	0	0	0	0	2(0.8%)	1(0.5%)	1(1.9%)	0
Adrenal insufficiency	1(0.2%)	0	0	0	0	1(0.4%)	1(0.5%)	0	0
Androgen deficiency	1(0.2%)	1(0.9%)	1(1.0%)	0	0	0	0	0	0
Cushing's syndrome	1(0.2%)	0	0	0	0	1(0.4%)	1(0.5%)	0	0
Hyperparathyroidism	1(0.2%)	0	0	0	0	1(0.4%)	1(0.5%)	0	0
Hypoparathyroidism	1(0.2%)	0	0	0	0	1(0.4%)	1(0.5%)	0	0
Thyroiditis	1(0.2%)	1(0.9%)	1(1.0%)	0	0	0	0	0	0
					Cohort 1: Newly Determined Advanced BCC Patients (Non-BCCNS) Metastatic BCC (mBCC)				
		Vismo (N=2)			No Vismo (N=1)				

	All (N=4)	All (N=2)	Vismo Only (N=2)	Surgery Only (N=0)	Other Therapy (N=0)	All (N=1)	Surgery Only (N=1)	Other Therapy (N=0)	No Treatment (N=1)
Immune system disorders (Cont'd)									
Allergy to fermented products	0	0	0	0	0	0	0	0	0
Allergy to plants	0	0	0	0	0	0	0	0	0
Anaphylactic reaction	0	0	0	0	0	0	0	0	0
Contrast media allergy	0	0	0	0	0	0	0	0	0
Endocrine disorders	0	0	0	0	0	0	0	0	0
Hypothyroidism	0	0	0	0	0	0	0	0	0
Hyperthyroidism	0	0	0	0	0	0	0	0	0
Adrenal insufficiency	0	0	0	0	0	0	0	0	0
Androgen deficiency	0	0	0	0	0	0	0	0	0
Cushing's syndrome	0	0	0	0	0	0	0	0	0
Hyperparathyroidism	0	0	0	0	0	0	0	0	0
Hypoparathyroidism	0	0	0	0	0	0	0	0	0
Thyroiditis	0	0	0	0	0	0	0	0	0
					Cohort 1: Newly Determined Advanced BCC Patients (Non-BCCNS) Locally Advanced BCC (laBCC)				
			Vismo (N=115)		No Vismo (N=251)				
	All (N=433)	All (N=115)	Vismo Only (N=102)	Surgery Only (N=6)	Other Therapy (N=7)	All (N=251)	Surgery Only (N=198)	Other Therapy (N=53)	No Treatment (N=67)
Reproductive system and breast disorders	46(10.6%)	6(5.2%)	5(4.9%)	0	1(14.3%)	29(11.6%)	24(12.1%)	5(9.4%)	11(16.4%)

					Cohort 1: Newly Determined Advanced BCC Patients (Non-BCCNS) Metastatic BCC (mBCC)				
			Vismo (N=2)		No Vismo (N=1)				
	All (N=4)	All (N=2)	Vismo Only (N=2)	Surgery Only (N=0)	Other Therapy (N=0)	All (N=1)	Surgery Only (N=1)	Other Therapy (N=0)	No Treatment (N=1)
Benign prostatic hyperplasia	24(5.5%)	3(2.6%)	3(2.9%)	0	0	17(6.8%)	12(6.1%)	5(9.4%)	4(6.0%)
Prostatomegaly	9(2.1%)	1(0.9%)	0	0	1(14.3%)	6(2.4%)	6(3.0%)	0	2(3.0%)
Erectile dysfunction	8(1.8%)	1(0.9%)	1(1.0%)	0	0	5(2.0%)	5(2.5%)	0	2(3.0%)
Atrophic vulvovaginitis	2(0.5%)	0	0	0	0	1(0.4%)	1(0.5%)	0	1(1.5%)
Endometriosis	2(0.5%)	1(0.9%)	1(1.0%)	0	0	1(0.4%)	1(0.5%)	0	0
Breast calcifications	1(0.2%)	0	0	0	0	0	0	0	1(1.5%)
Cervical dysplasia	1(0.2%)	0	0	0	0	0	0	0	1(1.5%)
Ovarian enlargement	1(0.2%)	0	0	0	0	1(0.4%)	1(0.5%)	0	0
Premenstrual syndrome	1(0.2%)	0	0	0	0	0	0	0	1(1.5%)
Vaginal prolapse	1(0.2%)	0	0	0	0	1(0.4%)	1(0.5%)	0	0
Injury, poisoning and procedural complications	43(9.9%)	13(11.3%)	13(12.7%)	0	0	20(8.0%)	16(8.1%)	4(7.5%)	10(14.9%)
Procedural pain	5(1.2%)	1(0.9%)	1(1.0%)	0	0	3(1.2%)	3(1.5%)	0	1(1.5%)
Foot fracture	3(0.7%)	2(1.7%)	2(2.0%)	0	0	1(0.4%)	1(0.5%)	0	0
Hip fracture	3(0.7%)	1(0.9%)	1(1.0%)	0	0	1(0.4%)	0	1(1.9%)	1(1.5%)
Sunburn	3(0.7%)	0	0	0	0	3(1.2%)	3(1.5%)	0	0

Reproductive system and breast disorders	1(25.0%)	0	0	0	0	0	0	0	1(100%)
Benign prostatic hyperplasia	0	0	0	0	0	0	0	0	0
Prostatomegaly	1(25.0%)	0	0	0	0	0	0	0	1(100%)
Erectile dysfunction	0	0	0	0	0	0	0	0	0
Atrophic vulvovaginitis	0	0	0	0	0	0	0	0	0
Endometriosis	0	0	0	0	0	0	0	0	0
Breast calcifications	0	0	0	0	0	0	0	0	0
Cervical dysplasia	0	0	0	0	0	0	0	0	0
Ovarian enlargement	0	0	0	0	0	0	0	0	0
Premenstrual syndrome	0	0	0	0	0	0	0	0	0
Vaginal prolapse	0	0	0	0	0	0	0	0	0
Injury, poisoning and procedural complications	0	0	0	0	0	0	0	0	0
Procedural pain	0	0	0	0	0	0	0	0	0
Foot fracture	0	0	0	0	0	0	0	0	0
Hip fracture	0	0	0	0	0	0	0	0	0
Sunburn	0	0	0	0	0	0	0	0	0
					Cohort 1: Newly Determined Advanced BCC Patients (Non-BCCNS) Locally Advanced BCC (laBCC)				
			Vismo (N=115)			No Vismo (N=251)			

	All (N=433)	All (N=115)	Vismo Only (N=102)	Surgery Only (N=6)	Other Therapy (N=7)	All (N=251)	Surgery Only (N=198)	Other Therapy (N=53)	No Treatment (N=67)
Injury, poisoning and procedural complications (Cont'd)									
Hand fracture	2(0.5%)	1(0.9%)	1(1.0%)	0	0	0	0	0	1(1.5%)
Humerus fracture	2(0.5%)	1(0.9%)	1(1.0%)	0	0	0	0	0	1(1.5%)
Scar	2(0.5%)	0	0	0	0	2(0.8%)	1(0.5%)	1(1.9%)	0
Upper limb fracture	2(0.5%)	1(0.9%)	1(1.0%)	0	0	0	0	0	1(1.5%)
Wound complication	2(0.5%)	1(0.9%)	1(1.0%)	0	0	1(0.4%)	1(0.5%)	0	0
Ankle fracture	1(0.2%)	0	0	0	0	1(0.4%)	1(0.5%)	0	0
Back injury	1(0.2%)	0	0	0	0	1(0.4%)	1(0.5%)	0	0
Blindness traumatic	1(0.2%)	0	0	0	0	1(0.4%)	0	1(1.9%)	0
Delayed effects of radiation	1(0.2%)	0	0	0	0	1(0.4%)	0	1(1.9%)	0
Epicondylitis	1(0.2%)	0	0	0	0	0	0	0	1(1.5%)
Exposure to chemical pollution	1(0.2%)	0	0	0	0	0	0	0	1(1.5%)
Eye injury	1(0.2%)	1(0.9%)	1(1.0%)	0	0	0	0	0	0
Fall	1(0.2%)	0	0	0	0	1(0.4%)	1(0.5%)	0	0
Femoral neck fracture	1(0.2%)	1(0.9%)	1(1.0%)	0	0	0	0	0	0
Femur fracture	1(0.2%)	0	0	0	0	0	0	0	1(1.5%)
Limb injury	1(0.2%)	1(0.9%)	1(1.0%)	0	0	0	0	0	0

					Cohort 1: Newly Determined Advanced BCC Patients (Non-BCCNS) Metastatic BCC (mBCC)				
			Vismo (N=2)		No Vismo (N=1)				
	All (N=4)	All (N=2)	Vismo Only (N=2)	Surgery Only (N=0)	Other Therapy (N=0)	All (N=1)	Surgery Only (N=1)	Other Therapy (N=0)	No Treatment (N=1)

	Cohort 1: Newly Determined Advanced BCC Patients (Non-BCCNS) Locally Advanced BCC (laBCC)								
		Vismo (N=115)			No Vismo (N=251)				
	All (N=433)	All (N=115)	Vismo Only (N=102)	Surgery Only (N=6)	Other Therapy (N=7)	All (N=251)	Surgery Only (N=198)	Other Therapy (N=53)	No Treatment (N=67)
Injury, poisoning and procedural complications (Cont'd)									
Hand fracture	0	0	0	0	0	0	0	0	0
Humerus fracture	0	0	0	0	0	0	0	0	0
Scar	0	0	0	0	0	0	0	0	0
Upper limb fracture	0	0	0	0	0	0	0	0	0
Wound complication	0	0	0	0	0	0	0	0	0
Ankle fracture	0	0	0	0	0	0	0	0	0
Back injury	0	0	0	0	0	0	0	0	0
Blindness traumatic	0	0	0	0	0	0	0	0	0
Delayed effects of radiation	0	0	0	0	0	0	0	0	0
Epicondylitis	0	0	0	0	0	0	0	0	0
Exposure to chemical pollution	0	0	0	0	0	0	0	0	0
Eye injury	0	0	0	0	0	0	0	0	0
Fall	0	0	0	0	0	0	0	0	0
Femoral neck fracture	0	0	0	0	0	0	0	0	0
Femur fracture	0	0	0	0	0	0	0	0	0
Limb injury	0	0	0	0	0	0	0	0	0

Injury, poisoning and procedural complications (Cont'd)									
Lower limb fracture	1(0.2%)	0	0	0	0	0	0	0	1(1.5%)
Mallet finger	1(0.2%)	1(0.9%)	1(1.0%)	0	0	0	0	0	0
Meniscus injury	1(0.2%)	0	0	0	0	0	0	0	1(1.5%)
Patella fracture	1(0.2%)	0	0	0	0	1(0.4%)	1(0.5%)	0	0
Pelvic fracture	1(0.2%)	0	0	0	0	0	0	0	1(1.5%)
Post procedural haemorrhage	1(0.2%)	1(0.9%)	1(1.0%)	0	0	0	0	0	0
Procedural pneumothorax	1(0.2%)	1(0.9%)	1(1.0%)	0	0	0	0	0	0
Radiation skin injury	1(0.2%)	0	0	0	0	0	0	0	1(1.5%)
Rib fracture	1(0.2%)	0	0	0	0	0	0	0	1(1.5%)
Skin abrasion	1(0.2%)	1(0.9%)	1(1.0%)	0	0	0	0	0	0
Skull fracture	1(0.2%)	0	0	0	0	1(0.4%)	1(0.5%)	0	0
Subdural haematoma	1(0.2%)	0	0	0	0	1(0.4%)	1(0.5%)	0	0
Tendon rupture	1(0.2%)	0	0	0	0	1(0.4%)	1(0.5%)	0	0
Thermal burn	1(0.2%)	1(0.9%)	1(1.0%)	0	0	0	0	0	0
Ulna fracture	1(0.2%)	1(0.9%)	1(1.0%)	0	0	0	0	0	0
Wound	1(0.2%)	0	0	0	0	1(0.4%)	0	1(1.9%)	0
Wrist fracture	1(0.2%)	0	0	0	0	1(0.4%)	1(0.5%)	0	0

		Cohort 1: Newly Determined Advanced BCC Patients (Non-BCCNS) Metastatic BCC (mBCC)	
	Vismo (N=2)	No Vismo (N=1)	

	All (N=4)	All (N=2)	Vismo Only (N=2)	Surgery Only (N=0)	Other Therapy (N=0)	All (N=1)	Surgery Only (N=1)	Other Therapy (N=0)	No Treatment (N=1)
Injury, poisoning and procedural complications (Cont'd)									
Lower limb fracture	0	0	0	0	0	0	0	0	0
Mallet finger	0	0	0	0	0	0	0	0	0
Meniscus injury	0	0	0	0	0	0	0	0	0
Patella fracture	0	0	0	0	0	0	0	0	0
Pelvic fracture	0	0	0	0	0	0	0	0	0
Post procedural haemorrhage	0	0	0	0	0	0	0	0	0
Procedural pneumothorax	0	0	0	0	0	0	0	0	0
Radiation skin injury	0	0	0	0	0	0	0	0	0
Rib fracture	0	0	0	0	0	0	0	0	0
Skin abrasion	0	0	0	0	0	0	0	0	0
Skull fracture	0	0	0	0	0	0	0	0	0
Subdural haematoma	0	0	0	0	0	0	0	0	0
Tendon rupture	0	0	0	0	0	0	0	0	0
Thermal burn	0	0	0	0	0	0	0	0	0
Ulna fracture	0	0	0	0	0	0	0	0	0
Wound	0	0	0	0	0	0	0	0	0
Wrist fracture	0	0	0	0	0	0	0	0	0

					Cohort 1: Newly Determined Advanced BCC Patients (Non-BCCNS) Locally Advanced BCC (laBCC)				
			Vismo (N=115)			No Vismo (N=251)			
	All (N=433)	All (N=115)	Vismo Only (N=102)	Surgery Only (N=6)	Other Therapy (N=7)	All (N=251)	Surgery Only (N=198)	Other Therapy (N=53)	No Treatment (N=67)
Renal and urinary disorders	37(8.5%)	9(7.8%)	7(6.9%)	0	2(28.6%)	22(8.8%)	18(9.1%)	4(7.5%)	6(9.0%)
Nephrolithiasis	10(2.3%)	4(3.5%)	3(2.9%)	0	1(14.3%)	5(2.0%)	5(2.5%)	0	1(1.5%)
Hypertonic bladder	6(1.4%)	0	0	0	0	5(2.0%)	4(2.0%)	1(1.9%)	1(1.5%)
Chronic kidney disease	4(0.9%)	2(1.7%)	2(2.0%)	0	0	2(0.8%)	2(1.0%)	0	0
Cystitis interstitial	3(0.7%)	0	0	0	0	3(1.2%)	2(1.0%)	1(1.9%)	0
Renal failure	3(0.7%)	2(1.7%)	1(1.0%)	0	1(14.3%)	0	0	0	1(1.5%)
Urinary retention	3(0.7%)	0	0	0	0	3(1.2%)	3(1.5%)	0	0
Nephropathy	2(0.5%)	1(0.9%)	1(1.0%)	0	0	1(0.4%)	1(0.5%)	0	0
Acute prerenal failure	1(0.2%)	0	0	0	0	1(0.4%)	1(0.5%)	0	0
Dysuria	1(0.2%)	0	0	0	0	0	0	0	1(1.5%)
End stage renal disease	1(0.2%)	0	0	0	0	1(0.4%)	0	1(1.9%)	0
Haematuria	1(0.2%)	0	0	0	0	1(0.4%)	0	1(1.9%)	0
Obstructive uropathy	1(0.2%)	0	0	0	0	0	0	0	1(1.5%)
Renal atrophy	1(0.2%)	0	0	0	0	1(0.4%)	1(0.5%)	0	0
Renal cortical necrosis	1(0.2%)	0	0	0	0	0	0	0	1(1.5%)
Urinary incontinence	1(0.2%)	0	0	0	0	1(0.4%)	1(0.5%)	0	0
Vesicoureteric reflux	1(0.2%)	0	0	0	0	1(0.4%)	1(0.5%)	0	0
					Cohort 1: Newly Determined Advanced BCC Patients (Non-BCCNS) Metastatic BCC (mBCC)				
			Vismo (N=2)			No Vismo (N=1)			

	All (N=4)	All (N=2)	Vismo Only (N=2)	Surgery Only (N=0)	Other Therapy (N=0)	All (N=1)	Surgery Only (N=1)	Other Therapy (N=0)	No Treatment (N=1)
Renal and urinary disorders	0	0	0	0	0	0	0	0	0
Nephrolithiasis	0	0	0	0	0	0	0	0	0
Hypertonic bladder	0	0	0	0	0	0	0	0	0
Chronic kidney disease	0	0	0	0	0	0	0	0	0
Cystitis interstitial	0	0	0	0	0	0	0	0	0
Renal failure	0	0	0	0	0	0	0	0	0
Urinary retention	0	0	0	0	0	0	0	0	0
Nephropathy	0	0	0	0	0	0	0	0	0
Acute prerenal failure	0	0	0	0	0	0	0	0	0
Dysuria	0	0	0	0	0	0	0	0	0
End stage renal disease	0	0	0	0	0	0	0	0	0
Haematuria	0	0	0	0	0	0	0	0	0
Obstructive uropathy	0	0	0	0	0	0	0	0	0
Renal atrophy	0	0	0	0	0	0	0	0	0
Renal cortical necrosis	0	0	0	0	0	0	0	0	0
Urinary incontinence	0	0	0	0	0	0	0	0	0
Vesicoureteric reflux	0	0	0	0	0	0	0	0	0

					Cohort 1: Newly Determined Advanced BCC Patients (Non-BCCNS) Locally Advanced BCC (laBCC)				
		Vismo (N=115)			No Vismo (N=251)				
	All (N=433)	All (N=115)	Vismo Only (N=102)	Surgery Only (N=6)	Other Therapy (N=7)	All (N=251)	Surgery Only (N=198)	Other Therapy (N=53)	No Treatment (N=67)

Blood and lymphatic system disorders	30(6.9%)	13(11.3%)	12(11.8%)	0	1(14.3%)	13(5.2%)	9(4.5%)	4(7.5%)	4(6.0%)
Anaemia	23(5.3%)	12(10.4%)	11(10.8%)	0	1(14.3%)	8(3.2%)	4(2.0%)	4(7.5%)	3(4.5%)
Anaemia of chronic disease	2(0.5%)	0	0	0	0	2(0.8%)	2(1.0%)	0	0
Iron deficiency anaemia	1(0.2%)	0	0	0	0	1(0.4%)	1(0.5%)	0	0
Leukocytosis	1(0.2%)	1(0.9%)	1(1.0%)	0	0	0	0	0	0
Lymphocytosis	1(0.2%)	1(0.9%)	1(1.0%)	0	0	0	0	0	0
Microcytic anaemia	1(0.2%)	0	0	0	0	1(0.4%)	1(0.5%)	0	0
Normochromic normocytic anaemia	1(0.2%)	0	0	0	0	0	0	0	1(1.5%)
Pernicious anaemia	1(0.2%)	0	0	0	0	1(0.4%)	1(0.5%)	0	0
Splenomegaly	1(0.2%)	0	0	0	0	0	0	0	1(1.5%)
Thrombocytopenia	1(0.2%)	0	0	0	0	0	0	0	1(1.5%)
Thrombocytosis	1(0.2%)	1(0.9%)	0	0	1(14.3%)	0	0	0	0
Ear and labyrinth disorders	22(5.1%)	3(2.6%)	3(2.9%)	0	0	15(6.0%)	13(6.6%)	2(3.8%)	4(6.0%)
Deafness	7(1.6%)	0	0	0	0	6(2.4%)	5(2.5%)	1(1.9%)	1(1.5%)
Deafness bilateral	2(0.5%)	0	0	0	0	1(0.4%)	1(0.5%)	0	1(1.5%)
Deafness unilateral	2(0.5%)	1(0.9%)	1(1.0%)	0	0	1(0.4%)	1(0.5%)	0	0
					Cohort 1: Newly Determined Advanced BCC Patients (Non-BCCNS) Metastatic BCC (mBCC)				
		Vismo (N=2)			No Vismo (N=1)				
	All (N=4)	All (N=2)	Vismo Only (N=2)	Surgery Only (N=0)	Other Therapy (N=0)	All (N=1)	Surgery Only (N=1)	Other Therapy (N=0)	No Treatment (N=1)

Blood and lymphatic system disorders	0	0	0	0	0	0	0	0	0
Anaemia	0	0	0	0	0	0	0	0	0
Anaemia of chronic disease	0	0	0	0	0	0	0	0	0
Iron deficiency anaemia	0	0	0	0	0	0	0	0	0
Leukocytosis	0	0	0	0	0	0	0	0	0
Lymphocytosis	0	0	0	0	0	0	0	0	0
Microcytic anaemia	0	0	0	0	0	0	0	0	0
Normochromic normocytic anaemia	0	0	0	0	0	0	0	0	0
Pernicious anaemia	0	0	0	0	0	0	0	0	0
Splenomegaly	0	0	0	0	0	0	0	0	0
Thrombocytopenia	0	0	0	0	0	0	0	0	0
Thrombocytosis	0	0	0	0	0	0	0	0	0
Ear and labyrinth disorders	1(25.0%)	1(50.0%)	1(50.0%)	0	0	0	0	0	0
Deafness	0	0	0	0	0	0	0	0	0
Deafness bilateral	0	0	0	0	0	0	0	0	0
Deafness unilateral	0	0	0	0	0	0	0	0	0
					Cohort 1: Newly Determined Advanced BCC Patients (Non-BCCNS) Locally Advanced BCC (laBCC)				
			Vismo (N=115)		No Vismo (N=251)				
	All (N=433)	All (N=115)	Vismo Only (N=102)	Surgery Only (N=6)	Other Therapy (N=7)	All (N=251)	Surgery Only (N=198)	Other Therapy (N=53)	No Treatment (N=67)

Ear and labyrinth disorders (Cont'd)									
Hypoacusis	2(0.5%)	0	0	0	0	1(0.4%)	1(0.5%)	0	1(1.5%)
Meniere's disease	2(0.5%)	0	0	0	0	1(0.4%)	1(0.5%)	0	1(1.5%)
Tinnitus	2(0.5%)	0	0	0	0	2(0.8%)	2(1.0%)	0	0
Vertigo	2(0.5%)	1(0.9%)	1(1.0%)	0	0	1(0.4%)	1(0.5%)	0	0
Deafness neurosensory	1(0.2%)	1(0.9%)	1(1.0%)	0	0	0	0	0	0
Ear pain	0	0	0	0	0	0	0	0	0
Presbyacusis	1(0.2%)	0	0	0	0	1(0.4%)	1(0.5%)	0	0
Tympanic membrane perforation	1(0.2%)	0	0	0	0	1(0.4%)	0	1(1.9%)	0
Vertigo positional	1(0.2%)	0	0	0	0	1(0.4%)	1(0.5%)	0	0
Congenital, familial and genetic disorders	13(3.0%)	2(1.7%)	2(2.0%)	0	0	8(3.2%)	5(2.5%)	3(5.7%)	3(4.5%)
Albinism	2(0.5%)	1(0.9%)	1(1.0%)	0	0	0	0	0	1(1.5%)
Congenital cystic kidney disease	2(0.5%)	0	0	0	0	2(0.8%)	1(0.5%)	1(1.9%)	0
Type V hyperlipidaemia	2(0.5%)	0	0	0	0	1(0.4%)	1(0.5%)	0	1(1.5%)

					Cohort 1: Newly Determined Advanced BCC Patients (Non-BCCNS) Metastatic BCC (mBCC)				
			Vismo (N=2)		No Vismo (N=1)				
	All (N=4)	All (N=2)	Vismo Only (N=2)	Surgery Only (N=0)	Other Therapy (N=0)	All (N=1)	Surgery Only (N=1)	Other Therapy (N=0)	No Treatment (N=1)
Ear and labyrinth disorders (Cont'd)									
Hypoacusis	0	0	0	0	0	0	0	0	0
Meniere's disease	0	0	0	0	0	0	0	0	0

Tinnitus	0	0	0	0	0	0	0	0	0
Vertigo	0	0	0	0	0	0	0	0	0
Deafness neurosensory	0	0	0	0	0	0	0	0	0
Ear pain	1(25.0%)	1(50.0%)	1(50.0%)	0	0	0	0	0	0
Presbyacusis	0	0	0	0	0	0	0	0	0
Tympanic membrane perforation	0	0	0	0	0	0	0	0	0
Vertigo positional	0	0	0	0	0	0	0	0	0
Congenital, familial and genetic disorders	0	0	0	0	0	0	0	0	0
Albinism	0	0	0	0	0	0	0	0	0
Congenital cystic kidney disease	0	0	0	0	0	0	0	0	0
Type V hyperlipidaemia	0	0	0	0	0	0	0	0	0

					Cohort 1: Newly Determined Advanced BCC Patients (Non-BCCNS) Locally Advanced BCC (laBCC)				
		Vismo (N=115)			No Vismo (N=251)				
	All (N=433)	All (N=115)	Vismo Only (N=102)	Surgery Only (N=6)	Other Therapy (N=7)	All (N=251)	Surgery Only (N=198)	Other Therapy (N=53)	No Treatment (N=67)
Congenital, familial and genetic disorders (Cont'd)									
Arnold-Chiari malformation	1(0.2%)	0	0	0	0	1(0.4%)	1(0.5%)	0	0
Cerebral palsy	1(0.2%)	0	0	0	0	1(0.4%)	1(0.5%)	0	0
Factor V Leiden mutation	1(0.2%)	0	0	0	0	1(0.4%)	0	1(1.9%)	0

Neurofibromatosis	1(0.2%)	0	0	0	0	1(0.4%)	0	1(1.9%)	0
Retinitis pigmentosa	1(0.2%)	1(0.9%)	1(1.0%)	0	0	0	0	0	0
Thalassaemia	1(0.2%)	0	0	0	0	0	0	0	1(1.5%)
Tourette's disorder	1(0.2%)	0	0	0	0	1(0.4%)	1(0.5%)	0	0
Hepatobiliary disorders	12(2.8%)	4(3.5%)	4(3.9%)	0	0	7(2.8%)	6(3.0%)	1(1.9%)	1(1.5%)
Cholelithiasis	4(0.9%)	1(0.9%)	1(1.0%)	0	0	3(1.2%)	3(1.5%)	0	0
Cholecystitis	2(0.5%)	1(0.9%)	1(1.0%)	0	0	1(0.4%)	1(0.5%)	0	0
Hepatic cirrhosis	2(0.5%)	0	0	0	0	1(0.4%)	1(0.5%)	0	1(1.5%)
Biliary cirrhosis primary	1(0.2%)	0	0	0	0	1(0.4%)	1(0.5%)	0	0
Cholecystitis acute	1(0.2%)	0	0	0	0	1(0.4%)	0	1(1.9%)	0
Hepatic cyst	1(0.2%)	0	0	0	0	1(0.4%)	1(0.5%)	0	0
Hepatic mass	1(0.2%)	0	0	0	0	1(0.4%)	1(0.5%)	0	0
Hepatitis	1(0.2%)	1(0.9%)	1(1.0%)	0	0	0	0	0	0

					Cohort 1: Newly Determined Advanced BCC Patients (Non-BCCNS) Metastatic BCC (mBCC)				
			Vismo (N=2)			No Vismo (N=1)			
	All (N=4)	All (N=2)	Vismo Only (N=2)	Surgery Only (N=0)	Other Therapy (N=0)	All (N=1)	Surgery Only (N=1)	Other Therapy (N=0)	No Treatment (N=1)
Congenital, familial and genetic disorders (Cont'd)									
Arnold-Chiari malformation	0	0	0	0	0	0	0	0	0
Cerebral palsy	0	0	0	0	0	0	0	0	0
Factor V Leiden mutation	0	0	0	0	0	0	0	0	0

Neurofibromatosis	0	0	0	0	0	0	0	0	0
Retinitis pigmentosa	0	0	0	0	0	0	0	0	0
Thalassaemia	0	0	0	0	0	0	0	0	0
Tourette's disorder	0	0	0	0	0	0	0	0	0
Hepatobiliary disorders	0	0	0	0	0	0	0	0	0
Cholelithiasis	0	0	0	0	0	0	0	0	0
Cholecystitis	0	0	0	0	0	0	0	0	0
Hepatic cirrhosis	0	0	0	0	0	0	0	0	0
Biliary cirrhosis primary	0	0	0	0	0	0	0	0	0
Cholecystitis acute	0	0	0	0	0	0	0	0	0
Hepatic cyst	0	0	0	0	0	0	0	0	0
Hepatic mass	0	0	0	0	0	0	0	0	0
Hepatitis	0	0	0	0	0	0	0	0	0

					Cohort 1: Newly Determined Advanced BCC Patients (Non-BCCNS) Locally Advanced BCC (laBCC)				
		Vismo (N=115)			No Vismo (N=251)				
	All (N=433)	All (N=115)	Vismo Only (N=102)	Surgery Only (N=6)	Other Therapy (N=7)	All (N=251)	Surgery Only (N=198)	Other Therapy (N=53)	No Treatment (N=67)
Hepatobiliary disorders (Cont'd)									
Hepatomegaly	1(0.2%)	0	0	0	0	0	0	0	1(1.5%)
Liver disorder	1(0.2%)	1(0.9%)	1(1.0%)	0	0	0	0	0	0
UNCODED	4(0.9%)	0	0	0	0	2(0.8%)	1(0.5%)	1(1.9%)	2(3.0%)
UNCODED	4(0.9%)	0	0	0	0	2(0.8%)	1(0.5%)	1(1.9%)	2(3.0%)

Pregnancy, puerperium and perinatal conditions	3(0.7%)	0	0	0	0	2(0.8%)	1(0.5%)	1(1.9%)	1(1.5%)
Previous caesarean section	2(0.5%)	0	0	0	0	1(0.4%)	0	1(1.9%)	1(1.5%)
Gestational diabetes	1(0.2%)	0	0	0	0	1(0.4%)	1(0.5%)	0	0
					Cohort 1: Newly Determined Advanced BCC Patients (Non-BCCNS) Metastatic BCC (mBCC)				
			Vismo (N=2)			No Vismo (N=1)			
	All (N=4)	All (N=2)	Vismo Only (N=2)	Surgery Only (N=0)	Other Therapy (N=0)	All (N=1)	Surgery Only (N=1)	Other Therapy (N=0)	No Treatment (N=1)
Hepatobiliary disorders (Cont'd)									
Hepatomegaly	0	0	0	0	0	0	0	0	0
Liver disorder	0	0	0	0	0	0	0	0	0
UNCODED	0	0	0	0	0	0	0	0	0
UNCODED	0	0	0	0	0	0	0	0	0
Pregnancy, puerperium and perinatal conditions	0	0	0	0	0	0	0	0	0
Previous caesarean section	0	0	0	0	0	0	0	0	0
Gestational diabetes	0	0	0	0	0	0	0	0	0

Note: “Vismo” group is defined as initiating vismodegib less than or equal to 90 days after the date of determination of locally advanced BCC (laBCC) or metastatic BCC (mBCC); “No Vismo” group is defined as not initiating vismodegib (but other BCC treatment) less than or equal to 90 days after the date of determination of laBCC or mBCC; “No Treatment” group is defined as not receiving any BCC treatment less than or equal to 90 days after the date of determination of laBCC or mBCC.

“Vismo Only” and “Surgery Only” are defined as having only 1 of these treatments less than or equal to 90 days after the date of determination of laBCC or mBCC; “Other Therapy” includes individual or combination treatments (e.g., surgery, PDT, ED&C, topical treatment, cryosurgery, systemic treatment, other) initiated less than or equal to 90 days after date of determination of laBCC or mBCC. For example, if patient receives topical treatment followed by vismo followed by surgery, then all 3 treatments must be initiated within the 90 days of the date of determination of disease.

Only the treatments that started and stopped greater than 90 days prior to enrollment are collected on the prior treatment for current disease CRF form and are included in this table.

[1] A patient may be counted in more than one category. The percentages may add up to more than 100%.

					Cohort 1: Newly Determined Advanced BCC Patients (Non-BCCNS) Locally Advanced BCC (laBCC)				
		Vismo (N=115)			No Vismo (N=251)				
	All (N=433)	All (N=115)	Vismo Only (N=102)	Surgery Only (N=6)	Other Therapy (N=7)	All (N=251)	Surgery Only (N=198)	Other Therapy (N=53)	No Treatment (N=67)
Prior Treatment for Current Disease [1]									
n	176	43	39	3	1	100	76	24	33
None	34(19.3%)	1(2.3%)	1(2.6%)	0	0	26(26.0%)	23(30.3%)	3(12.5%)	7(21.2%)
Vismodegib	1(0.6%)	1(2.3%)	1(2.6%)	0	0	0	0	0	0
Other Systemic Treatment	1(0.6%)	1(2.3%)	0	1(33.3%)	0	0	0	0	0
Surgery	132(75.0%)	39(90.7%)	35(89.7%)	3(100%)	1(100%)	71(71.0%)	52(68.4%)	19(79.2%)	22(66.7%)
Radiation	21(11.9%)	10(23.3%)	9(23.1%)	1(33.3%)	0	3(3.0%)	2(2.6%)	1(4.2%)	8(24.2%)
Photodynamic Therapy	0	0	0	0	0	0	0	0	0
Topical Treatment	19(10.8%)	6(14.0%)	6(15.4%)	0	0	8(8.0%)	4(5.3%)	4(16.7%)	5(15.2%)
Other	3(1.7%)	1(2.3%)	1(2.6%)	0	0	0	0	0	2(6.1%)
If Surgery, Number of BCC related Surgeries in the Preceding 2 Years									
n	124	34	30	3	1	68	51	17	22
1-5	110(88.7%)	31(91.2%)	27(90.0%)	3(100%)	1(100%)	62(91.2%)	46(90.2%)	16(94.1%)	17(77.3%)
6-10	12(9.7%)	3(8.8%)	3(10.0%)	0	0	5(7.4%)	4(7.8%)	1(5.9%)	4(18.2%)
>10	2(1.6%)	0	0	0	0	1(1.5%)	1(2.0%)	0	1(4.5%)

	Cohort 1: Newly Determined Advanced BCC Patients (Non-BCCNS) Metastatic BCC (mBCC)							
		Vismo (N=2)			No Vismo (N=1)			
	All (N=4)	All (N=2)	Vismo Only (N=2)	Surgery Only (N=0)	Other Therapy (N=0)	All (N=1)	Surgery Only (N=1)	Other Therapy (N=0)	No Treatment (N=1)
Prior Treatment for Current Disease [1]									
n	2	0	0	0	0	1	1	0	1
None	0	0	0	0	0	0	0	0	0
Vismodegib	0	0	0	0	0	0	0	0	0
Other Systemic Treatment	0	0	0	0	0	0	0	0	0
Surgery	2(100%)	0	0	0	0	1(100%)	1(100%)	0	1(100%)
Radiation	1(50.0%)	0	0	0	0	0	0	0	1(100%)
Photodynamic Therapy	0	0	0	0	0	0	0	0	0
Topical Treatment	0	0	0	0	0	0	0	0	0
Other	0	0	0	0	0	0	0	0	0
If Surgery, Number of BCC related Surgeries in the Preceding 2 Years									
n	2	0	0	0	0	1	1	0	1
1-5	2(100%)	0	0	0	0	1(100%)	1(100%)	0	1(100%)
6-10	0	0	0	0	0	0	0	0	0
>10	0	0	0	0	0	0	0	0	0

Note: "Vismo" group is defined as initiating vismodegib less than or equal to 90 days after the date of determination of locally advanced BCC (laBCC) or metastatic BCC (mBCC); "No Vismo" group is defined as not initiating vismodegib (but other BCC treatment) less than or equal to 90 days after the date of determination of laBCC or mBCC; "No Treatment" group is defined as not receiving any BCC treatment less than or equal to 90 days after the date of determination of laBCC or mBCC.

"Vismo Only" and "Surgery Only" are defined as having only 1 of these treatments less than or equal to 90 days after the date of determination of laBCC or mBCC; "Other Therapy" includes individual or combination treatments (e.g., surgery, PDT, ED&C, topical treatment, cryosurgery, systemic treatment, other) initiated less than or equal to 90 days after date of determination of laBCC or mBCC. For example, if patient receives topical treatment followed by vismo followed by surgery, then all 3 treatments must be initiated within the 90 days of the date of determination of disease.

[1] Optional DNA blood sample.

[2] A patient may be counted in more than one category. The percentages may add up to more than 100%.

[3] Summarized lesion locations for subjects with multiple locally advanced lesions and target lesion location for subjects with single locally advanced lesion on baseline disease assessment aCRF.

		Cohort 1: Newly Determined Advanced BCC Patients (Non-BCCNS) Locally Advanced BCC (laBCC)							
		Vismo (N=115)				No Vismo (N=251)			
Disease Assessment at Baseline: Overall	All (N=433)	All (N=115)	Vismo Only (N=102)	Surgery Only (N=6)	Other Therapy (N=7)	All (N=251)	Surgery Only (N=198)	Other Therapy (N=53)	No Treatment (N=67)
Current Blood Sample Available [1]									
n	108	50	45	3	2	39	35	4	19
Yes	29(26.9%)	23(46.0%)	18(40.0%)	3(100%)	2(100%)	2(5.1%)	1(2.9%)	1(25.0%)	4(21.1%)
No	79(73.1%)	27(54.0%)	27(60.0%)	0	0	37(94.9%)	34(97.1%)	3(75.0%)	15(78.9%)
If Yes, was Sample Submitted									
n	29	23	18	3	2	2	1	1	4
Yes	29(100%)	23(100%)	18(100%)	3(100%)	2(100%)	2(100%)	1(100%)	1(100%)	4(100%)
No	0	0	0	0	0	0	0	0	0
Current Diagnostic Images Available									
n	41	18	16	1	1	16	12	4	7
Yes	8(19.5%)	4(22.2%)	3(18.8%)	0	1(100%)	1(6.3%)	1(8.3%)	0	3(42.9%)
No	33(80.5%)	14(77.8%)	13(81.3%)	1(100%)	0	15(93.8%)	11(91.7%)	4(100%)	4(57.1%)
Current Photographs Available									

n	70	39	37	1	1	19	16	3	12
Yes	57(81.4%)	34(87.2%)	32(86.5%)	1(100%)	1(100%)	15(78.9%)	14(87.5%)	1(33.3%)	8(66.7%)
No	13(18.6%)	5(12.8%)	5(13.5%)	0	0	4(21.1%)	2(12.5%)	2(66.7%)	4(33.3%)
					Cohort 1: Newly Determined Advanced BCC Patients (Non-BCCNS) Metastatic BCC (mBCC)				
			Vismo (N=2)			No Vismo (N=1)			
Disease Assessment at Baseline: Overall	All (N=4)	All (N=2)	Vismo Only (N=2)	Surgery Only (N=0)	Other Therapy (N=0)	All (N=1)	Surgery Only (N=1)	Other Therapy (N=0)	No Treatment (N=1)
Current Blood Sample Available [1]									
n	3	1	1	0	0	1	1	0	1
Yes	2(66.7%)	1(100%)	1(100%)	0	0	1(100%)	1(100%)	0	0
No	1(33.3%)	0	0	0	0	0	0	0	1(100%)
If Yes, was Sample Submitted									
n	2	1	1	0	0	1	1	0	0
Yes	2(100%)	1(100%)	1(100%)	0	0	1(100%)	1(100%)	0	0
No	0	0	0	0	0	0	0	0	0
Current Diagnostic Images Available									
n	2	1	1	0	0	0	0	0	1
Yes	0	0	0	0	0	0	0	0	0
No	2(100%)	1(100%)	1(100%)	0	0	0	0	0	1(100%)
Current Photographs Available									
n	3	2	2	0	0	0	0	0	1
Yes	1(33.3%)	0	0	0	0	0	0	0	1(100%)
No	2(66.7%)	2(100%)	2(100%)	0	0	0	0	0	0
					Cohort 1: Newly Determined Advanced BCC Patients (Non-BCCNS) Locally Advanced BCC (laBCC)				
			Vismo (N=115)			No Vismo (N=251)			
Disease Assessment at Baseline: Overall	All (N=433)	All (N=115)	Vismo Only (N=102)	Surgery Only (N=6)	Other Therapy (N=7)	All (N=251)	Surgery Only (N=198)	Other Therapy (N=53)	No Treatment (N=67)

ECOG PS Prior to Treatment									
n	350	83	74	6	3	209	173	36	58
0	184(52.6%)	35(42.2%)	32(43.2%)	2(33.3%)	1(33.3%)	124(59.3%)	106(61.3%)	18(50.0%)	25(43.1%)
1	36(10.3%)	13(15.7%)	13(17.6%)	0	0	16(7.7%)	13(7.5%)	3(8.3%)	7(12.1%)
2	22(6.3%)	7(8.4%)	6(8.1%)	0	1(33.3%)	13(6.2%)	11(6.4%)	2(5.6%)	2(3.4%)
3	9(2.6%)	0	0	0	0	4(1.9%)	2(1.2%)	2(5.6%)	5(8.6%)
4	6(1.7%)	4(4.8%)	3(4.1%)	0	1(33.3%)	1(0.5%)	0	1(2.8%)	1(1.7%)
Unknown	93(26.6%)	24(28.9%)	20(27.0%)	4(66.7%)	0	51(24.4%)	41(23.7%)	10(27.8%)	18(31.0%)
ECOG PS Prior to Enrollment									
n	321	87	74	6	7	183	140	43	51
0	166(51.7%)	38(43.7%)	34(45.9%)	2(33.3%)	2(28.6%)	109(59.6%)	88(62.9%)	21(48.8%)	19(37.3%)
1	45(14.0%)	18(20.7%)	16(21.6%)	0	2(28.6%)	18(9.8%)	11(7.9%)	7(16.3%)	9(17.6%)
2	14(4.4%)	5(5.7%)	4(5.4%)	0	1(14.3%)	7(3.8%)	3(2.1%)	4(9.3%)	2(3.9%)
3	14(4.4%)	3(3.4%)	3(4.1%)	0	0	6(3.3%)	3(2.1%)	3(7.0%)	5(9.8%)
4	6(1.9%)	4(4.6%)	2(2.7%)	0	2(28.6%)	1(0.5%)	0	1(2.3%)	1(2.0%)
Unknown	76(23.7%)	19(21.8%)	15(20.3%)	4(66.7%)	0	42(23.0%)	35(25.0%)	7(16.3%)	15(29.4%)

					Cohort 1: Newly Determined Advanced BCC Patients (Non-BCCNS) Metastatic BCC (mBCC)				
			Vismo (N=2)			No Vismo (N=1)			
Disease Assessment at Baseline: Overall	All (N=4)	All (N=2)	Vismo Only (N=2)	Surgery Only (N=0)	Other Therapy (N=0)	All (N=1)	Surgery Only (N=1)	Other Therapy (N=0)	No Treatment (N=1)
ECOG PS Prior to Treatment									
n	3	1	1	0	0	1	1	0	1
0	2(66.7%)	1(100%)	1(100%)	0	0	1(100%)	1(100%)	0	0
1	0	0	0	0	0	0	0	0	0
2	1(33.3%)	0	0	0	0	0	0	0	1(100%)
3	0	0	0	0	0	0	0	0	0
4	0	0	0	0	0	0	0	0	0
Unknown	0	0	0	0	0	0	0	0	0
ECOG PS Prior to Enrollment									
n	4	2	2	0	0	1	1	0	1
0	2(50.0%)	1(50.0%)	1(50.0%)	0	0	1(100%)	1(100%)	0	0
1	1(25.0%)	1(50.0%)	1(50.0%)	0	0	0	0	0	0
2	1(25.0%)	0	0	0	0	0	0	0	1(100%)

3	0	0	0	0	0	0	0	0	0
4	0	0	0	0	0	0	0	0	0
Unknown	0	0	0	0	0	0	0	0	0
					Cohort 1: Newly Determined Advanced BCC Patients (Non-BCCNS) Locally Advanced BCC (laBCC)				
		Vismo (N=115)			No Vismo (N=251)				
Disease Assessment at Baseline: Locally Advanced Disease	All (N=433)	All (N=115)	Vismo Only (N=102)	Surgery Only (N=6)	Other Therapy (N=7)	All (N=251)	Surgery Only (N=198)	Other Therapy (N=53)	No Treatment (N=67)
Prior History of BCC?									
n	433	115	102	6	7	251	198	53	67
Yes	281(64.9%)	80(69.6%)	72(70.6%)	5(83.3%)	3(42.9%)	149(59.4%)	117(59.1%)	32(60.4%)	52(77.6%)
No	152(35.1%)	35(30.4%)	30(29.4%)	1(16.7%)	4(57.1%)	102(40.6%)	81(40.9%)	21(39.6%)	15(22.4%)
Is this the initial determination (de novo) of laBCC or mBCC?									
n	433	115	102	6	7	251	198	53	67
Yes	360(83.1%)	91(79.1%)	80(78.4%)	5(83.3%)	6(85.7%)	217(86.5%)	170(85.9%)	47(88.7%)	52(77.6%)
No	73(16.9%)	24(20.9%)	22(21.6%)	1(16.7%)	1(14.3%)	34(13.5%)	28(14.1%)	6(11.3%)	15(22.4%)
Does the pt have laBCC that is clinically visible?									
n	433	115	102	6	7	251	198	53	67
Yes	433(100%)	115(100%)	102(100%)	6(100%)	7(100%)	251(100%)	198(100%)	53(100%)	67(100%)
No	0	0	0	0	0	0	0	0	0
Time from Determination of laBCC to Enrollment (Months)									
n	433	115	102	6	7	251	198	53	67
Mean (SD)	1.071 (4.1972)	0.560 (0.8238)	0.575 (0.8627)	0.498 (0.4497)	0.390 (0.3963)	0.796 (0.9056)	0.853 (0.9087)	0.583 (0.8695)	2.978 (10.3244)
Median	0.394	0.099	0.000	0.329	0.394	0.460	0.460	0.000	0.559

Range	(-0.30, 75.66)	(-0.30, 3.68)	(-0.30, 3.68)	(0.00, 1.15)	(0.00, 0.85)	(0.00, 3.09)	(0.00, 3.09)	(0.00, 3.06)	(-0.20, 75.66)
					Cohort 1: Newly Determined Advanced BCC Patients (Non-BCCNS) Metastatic BCC (mBCC)				
			Vismo (N=2)			No Vismo (N=1)			
Disease Assessment at Baseline: Locally Advanced Disease	All (N=4)	All (N=2)	Vismo Only (N=2)	Surgery Only (N=0)	Other Therapy (N=0)	All (N=1)	Surgery Only (N=1)	Other Therapy (N=0)	No Treatment (N=1)
Prior History of BCC?									
n	4	2	2	0	0	1	1	0	1
Yes	3(75.0%)	1(50.0%)	1(50.0%)	0	0	1(100%)	1(100%)	0	1(100%)
No	1(25.0%)	1(50.0%)	1(50.0%)	0	0	0	0	0	0
Is this the initial determination (de novo) of laBCC or mBCC?									
n	4	2	2	0	0	1	1	0	1
Yes	3(75.0%)	1(50.0%)	1(50.0%)	0	0	1(100%)	1(100%)	0	1(100%)
No	1(25.0%)	1(50.0%)	1(50.0%)	0	0	0	0	0	0
Does the pt have laBCC that is clinically visible?									
n	4	2	2	0	0	1	1	0	1
Yes	0	0	0	0	0	0	0	0	0
No	4(100%)	2(100%)	2(100%)	0	0	1(100%)	1(100%)	0	1(100%)
Time from Determination of laBCC to Enrollment (Months)									
n	0	0	0	0	0	0	0	0	0
Mean (SD)									
Median									
Range									

					Cohort 1: Newly Determined Advanced BCC Patients (Non-BCCNS) Locally Advanced BCC (laBCC)				
			Vismo (N=115)			No Vismo (N=251)			
Disease Assessment at Baseline: Locally Advanced Disease	All (N=433)	All (N=115)	Vismo Only (N=102)	Surgery Only (N=6)	Other Therapy (N=7)	All (N=251)	Surgery Only (N=198)	Other Therapy (N=53)	No Treatment (N=67)
Time from initial diagnosis of this BCC lesion to enrollment (Months)									
n	421	109	96	6	7	247	197	50	65
Mean (SD)	8.450 (26.0605)	13.425 (33.4297)	14.750 (35.3079)	6.056 (12.3121)	1.577 (2.1880)	5.363 (20.1936)	6.005 (22.2051)	2.835 (8.1727)	11.837 (30.2167)
Median	1.577	1.610	1.659	1.265	0.854	1.478	1.708	0.805	1.906
Range	(-0.30, 235.47)	(-0.30, 168.61)	(-0.30, 168.61)	(0.26, 31.15)	(0.00, 5.42)	(0.00, 235.47)	(0.00, 235.47)	(0.00, 52.11)	(0.00, 190.55)
The Definition of laBCC was Based on [2]									
n	431	114	101	6	7	251	198	53	66
Size	343(79.6%)	83(72.8%)	72(71.3%)	5(83.3%)	6(85.7%)	217(86.5%)	173(87.4%)	44(83.0%)	43(65.2%)
Extent	211(49.0%)	60(52.6%)	51(50.5%)	4(66.7%)	5(71.4%)	122(48.6%)	93(47.0%)	29(54.7%)	29(43.9%)
Location	199(46.2%)	76(66.7%)	68(67.3%)	3(50.0%)	5(71.4%)	85(33.9%)	61(30.8%)	24(45.3%)	38(57.6%)
Curative Resection Unlikely	109(25.3%)	56(49.1%)	49(48.5%)	2(33.3%)	5(71.4%)	34(13.5%)	17(8.6%)	17(32.1%)	19(28.8%)
Medical Contraindication to Surgery	57(13.2%)	29(25.4%)	23(22.8%)	1(16.7%)	5(71.4%)	17(6.8%)	3(1.5%)	14(26.4%)	11(16.7%)
Medical Contraindication to Radiation	73(16.9%)	35(30.7%)	30(29.7%)	1(16.7%)	4(57.1%)	26(10.4%)	13(6.6%)	13(24.5%)	12(18.2%)
Recurrence	115(26.7%)	43(37.7%)	40(39.6%)	3(50.0%)	0	41(16.3%)	32(16.2%)	9(17.0%)	31(47.0%)
Histopathology	234(54.3%)	57(50.0%)	56(55.4%)	1(16.7%)	0	149(59.4%)	133(67.2%)	16(30.2%)	28(42.4%)
Other	40(9.3%)	9(7.9%)	8(7.9%)	0	1(14.3%)	19(7.6%)	16(8.1%)	3(5.7%)	12(18.2%)

					Cohort 1: Newly Determined Advanced BCC Patients (Non-BCCNS) Metastatic BCC (mBCC)				
		Vismo (N=2)			No Vismo (N=1)				
Disease Assessment at Baseline: Locally Advanced Disease	All (N=4)	All (N=2)	Vismo Only (N=2)	Surgery Only (N=0)	Other Therapy (N=0)	All (N=1)	Surgery Only (N=1)	Other Therapy (N=0)	No Treatment (N=1)
Time from initial diagnosis of this BCC lesion to enrollment (Months)									
n	0	0	0	0	0	0	0	0	0
Mean (SD)									
Median									
Range									
The Definition of laBCC was Based on [2]									
n	0	0	0	0	0	0	0	0	0
Size	0	0	0	0	0	0	0	0	0
Extent	0	0	0	0	0	0	0	0	0
Location	0	0	0	0	0	0	0	0	0
Curative Resection Unlikely	0	0	0	0	0	0	0	0	0
Medical Contraindication to Surgery	0	0	0	0	0	0	0	0	0
Medical Contraindication to Radiation	0	0	0	0	0	0	0	0	0
Recurrence	0	0	0	0	0	0	0	0	0
Histopathology	0	0	0	0	0	0	0	0	0
Other	0	0	0	0	0	0	0	0	0
					Cohort 1: Newly Determined Advanced BCC Patients (Non-BCCNS) Locally Advanced BCC (laBCC)				
		Vismo (N=115)			No Vismo (N=251)				

Disease Assessment at Baseline: Locally Advanced Disease	All (N=433)	All (N=115)	Vismo Only (N=102)	Surgery Only (N=6)	Other Therapy (N=7)	All (N=251)	Surgery Only (N=198)	Other Therapy (N=53)	No Treatment (N=67)
If Size,									
n	343	83	72	5	6	217	173	44	43
<10 mm	39(11.4%)	9(10.8%)	9(12.5%)	0	0	25(11.5%)	18(10.4%)	7(15.9%)	5(11.6%)
10-19 mm	88(25.7%)	13(15.7%)	9(12.5%)	3(60.0%)	1(16.7%)	64(29.5%)	51(29.5%)	13(29.5%)	11(25.6%)
>=20 mm	216(63.0%)	61(73.5%)	54(75.0%)	2(40.0%)	5(83.3%)	128(59.0%)	104(60.1%)	24(54.5%)	27(62.8%)
If Recurrence, Number of Recurrences									
n	115	43	40	3	0	41	32	9	31
<2	68(59.1%)	28(65.1%)	25(62.5%)	3(100%)	0	25(61.0%)	20(62.5%)	5(55.6%)	15(48.4%)
>=2	47(40.9%)	15(34.9%)	15(37.5%)	0	0	16(39.0%)	12(37.5%)	4(44.4%)	16(51.6%)
Single or Multiple Clinically Visible Advanced Lesions									
n	433	115	102	6	7	251	198	53	67
Single Locally Advanced Lesion	332(76.7%)	77(67.0%)	69(67.6%)	3(50.0%)	5(71.4%)	207(82.5%)	172(86.9%)	35(66.0%)	48(71.6%)
Multiple Locally Advanced Lesions	101(23.3%)	38(33.0%)	33(32.4%)	3(50.0%)	2(28.6%)	44(17.5%)	26(13.1%)	18(34.0%)	19(28.4%)
If Multiple, Number of Lesions									
n	100	37	32	3	2	44	26	18	19
Mean (SD)	4.7 (5.49)	6.2 (8.37)	5.9 (8.65)	10.7 (8.08)	4.0 (1.41)	3.9 (2.27)	3.7 (1.96)	4.2 (2.69)	3.8 (2.46)
Median	3.0	4.0	3.0	6.0	4.0	3.0	3.0	3.0	3.0
Range	(2, 50)	(2, 50)	(2, 50)	(6, 20)	(3, 5)	(2, 10)	(2, 10)	(2, 10)	(2, 10)

					Cohort 1: Newly Determined Advanced BCC Patients (Non-BCCNS) Metastatic BCC (mBCC)				
		Vismo (N=2)			No Vismo (N=1)				
Disease Assessment at Baseline: Locally Advanced Disease	All (N=4)	All (N=2)	Vismo Only (N=2)	Surgery Only (N=0)	Other Therapy (N=0)	All (N=1)	Surgery Only (N=1)	Other Therapy (N=0)	No Treatment (N=1)
If Size,									
n	0	0	0	0	0	0	0	0	0
<10 mm	0	0	0	0	0	0	0	0	0
10-19 mm	0	0	0	0	0	0	0	0	0
>=20 mm	0	0	0	0	0	0	0	0	0
If Recurrence, Number of Recurrences									
n	0	0	0	0	0	0	0	0	0
<2	0	0	0	0	0	0	0	0	0
>=2	0	0	0	0	0	0	0	0	0
Single or Multiple Clinically Visible Advanced Lesions									
n	0	0	0	0	0	0	0	0	0
Single Locally Advanced Lesion	0	0	0	0	0	0	0	0	0
Multiple Locally Advanced Lesions	0	0	0	0	0	0	0	0	0
If Multiple, Number of Lesions									
n	0	0	0	0	0	0	0	0	0
Mean (SD)									

Median									
Range									
					Cohort 1: Newly Determined Advanced BCC Patients (Non-BCCNS) Locally Advanced BCC (laBCC)				
		Vismo (N=115)			No Vismo (N=251)				
Disease Assessment at Baseline: Locally Advanced Disease	All (N=433)	All (N=115)	Vismo Only (N=102)	Surgery Only (N=6)	Other Therapy (N=7)	All (N=251)	Surgery Only (N=198)	Other Therapy (N=53)	No Treatment (N=67)
If Multiple, Lesion locations for laBCC [2]									
n	101	38	33	3	2	44	26	18	19
Eye	2(2.0%)	2(5.3%)	2(6.1%)	0	0	0	0	0	0
Hand	6(5.9%)	2(5.3%)	2(6.1%)	0	0	4(9.1%)	2(7.7%)	2(11.1%)	0
Foot	1(1.0%)	0	0	0	0	1(2.3%)	1(3.8%)	0	0
Shoulder	20(19.8%)	9(23.7%)	6(18.2%)	2(66.7%)	1(50.0%)	8(18.2%)	6(23.1%)	2(11.1%)	3(15.8%)
Scalp	9(8.9%)	6(15.8%)	5(15.2%)	1(33.3%)	0	3(6.8%)	1(3.8%)	2(11.1%)	0
Ear	20(19.8%)	5(13.2%)	4(12.1%)	1(33.3%)	0	12(27.3%)	7(26.9%)	5(27.8%)	3(15.8%)
Cheek	25(24.8%)	9(23.7%)	8(24.2%)	1(33.3%)	0	7(15.9%)	5(19.2%)	2(11.1%)	9(47.4%)
Nose	22(21.8%)	9(23.7%)	7(21.2%)	2(66.7%)	0	7(15.9%)	5(19.2%)	2(11.1%)	6(31.6%)
Forehead	24(23.8%)	15(39.5%)	13(39.4%)	2(66.7%)	0	3(6.8%)	2(7.7%)	1(5.6%)	6(31.6%)
Chin	7(6.9%)	3(7.9%)	3(9.1%)	0	0	1(2.3%)	0	1(5.6%)	3(15.8%)
Neck	19(18.8%)	11(28.9%)	7(21.2%)	2(66.7%)	2(100%)	5(11.4%)	4(15.4%)	1(5.6%)	3(15.8%)
Back	44(43.6%)	21(55.3%)	17(51.5%)	2(66.7%)	2(100%)	18(40.9%)	11(42.3%)	7(38.9%)	5(26.3%)
Chest	26(25.7%)	11(28.9%)	9(27.3%)	1(33.3%)	1(50.0%)	12(27.3%)	7(26.9%)	5(27.8%)	3(15.8%)
Abdomen	6(5.9%)	4(10.5%)	3(9.1%)	1(33.3%)	0	1(2.3%)	1(3.8%)	0	1(5.3%)
Arm	27(26.7%)	13(34.2%)	11(33.3%)	1(33.3%)	1(50.0%)	12(27.3%)	5(19.2%)	7(38.9%)	2(10.5%)
Leg	23(22.8%)	8(21.1%)	7(21.2%)	1(33.3%)	0	10(22.7%)	3(11.5%)	7(38.9%)	5(26.3%)
Other	6(5.9%)	2(5.3%)	2(6.1%)	0	0	4(9.1%)	4(15.4%)	0	0
					Cohort 1: Newly Determined Advanced BCC Patients (Non-BCCNS) Metastatic BCC (mBCC)				
		Vismo (N=2)			No Vismo (N=1)				
Disease Assessment at Baseline: Locally Advanced Disease	All (N=4)	All (N=2)	Vismo Only (N=2)	Surgery Only (N=0)	Other Therapy (N=0)	All (N=1)	Surgery Only (N=1)	Other Therapy (N=0)	No Treatment (N=1)

If Multiple, Lesion locations for laBCC [2]									
n	0	0	0	0	0	0	0	0	0
Eye	0	0	0	0	0	0	0	0	0
Hand	0	0	0	0	0	0	0	0	0
Foot	0	0	0	0	0	0	0	0	0
Shoulder	0	0	0	0	0	0	0	0	0
Scalp	0	0	0	0	0	0	0	0	0
Ear	0	0	0	0	0	0	0	0	0
Cheek	0	0	0	0	0	0	0	0	0
Nose	0	0	0	0	0	0	0	0	0
Forehead	0	0	0	0	0	0	0	0	0
Chin	0	0	0	0	0	0	0	0	0
Neck	0	0	0	0	0	0	0	0	0
Back	0	0	0	0	0	0	0	0	0
Chest	0	0	0	0	0	0	0	0	0
Abdomen	0	0	0	0	0	0	0	0	0
Arm	0	0	0	0	0	0	0	0	0
Leg	0	0	0	0	0	0	0	0	0
Other	0	0	0	0	0	0	0	0	0

					Cohort 1: Newly Determined Advanced BCC Patients (Non-BCCNS) Locally Advanced BCC (laBCC)				
		Vismo (N=115)			No Vismo (N=251)				
Disease Assessment at Baseline: Metastatic Disease	All (N=433)	All (N=115)	Vismo Only (N=102)	Surgery Only (N=6)	Other Therapy (N=7)	All (N=251)	Surgery Only (N=198)	Other Therapy (N=53)	No Treatment (N=67)
Does the Patient Have mBCC?									
n	433	115	102	6	7	251	198	53	67
Yes	0	0	0	0	0	0	0	0	0
No	433(100%)	115(100%)	102(100%)	6(100%)	7(100%)	251(100%)	198(100%)	53(100%)	67(100%)
Time from Diagnosis of mBCC to Enrollment (Months)									
n	0	0	0	0	0	0	0	0	0
Mean (SD)									

Median									
Range									
Time from Initial Diagnosis of This BCC to Enrollment (Months)									
n	0	0	0	0	0	0	0	0	0
Mean (SD)									
Median									
Range									
					Cohort 1: Newly Determined Advanced BCC Patients (Non-BCCNS) Metastatic BCC (mBCC)				
			Vismo (N=2)			No Vismo (N=1)			
Disease Assessment at Baseline: Metastatic Disease	All (N=4)	All (N=2)	Vismo Only (N=2)	Surgery Only (N=0)	Other Therapy (N=0)	All (N=1)	Surgery Only (N=1)	Other Therapy (N=0)	No Treatment (N=1)
Does the Patient Have mBCC?									
n	4	2	2	0	0	1	1	0	1
Yes	4(100%)	2(100%)	2(100%)	0	0	1(100%)	1(100%)	0	1(100%)
No	0	0	0	0	0	0	0	0	0
Time from Diagnosis of mBCC to Enrollment (Months)									
n	4	2	2	0	0	1	1	0	1
Mean (SD)	6.259 (11.6889)	0.476 (0.4879)	0.476 (0.4879)			0.296 (-)	0.296 (-)		23.786 (-)
Median	0.559	0.476	0.476			0.296	0.296		23.786
Range	(0.13, 23.79)	(0.13, 0.82)	(0.13, 0.82)			(0.30, 0.30)	(0.30, 0.30)		(23.79, 23.79)
Time from Initial Diagnosis of This BCC to Enrollment (Months)									
n	4	2	2	0	0	1	1	0	1

Mean (SD)	18.267 (33.6859)	0.641 (0.7202)	0.641 (0.7202)			3.023 (-)	3.023 (-)		68.764 (-)
Median	2.086	0.641	0.641			3.023	3.023		68.764
Range	(0.13, 68.76)	(0.13, 1.15)	(0.13, 1.15)			(3.02, 3.02)	(3.02, 3.02)		(68.76, 68.76)
					Cohort 1: Newly Determined Advanced BCC Patients (Non-BCCNS) Locally Advanced BCC (laBCC)				
		Vismo (N=115)			No Vismo (N=251)				
Disease Assessment at Baseline: Metastatic Disease	All (N=433)	All (N=115)	Vismo Only (N=102)	Surgery Only (N=6)	Other Therapy (N=7)	All (N=251)	Surgery Only (N=198)	Other Therapy (N=53)	No Treatment (N=67)
Histopathology of Current mBCC [2]									
n	0	0	0	0	0	0	0	0	0
Nodular	0	0	0	0	0	0	0	0	0
Superficial	0	0	0	0	0	0	0	0	0
Morpheiform / Infiltrative	0	0	0	0	0	0	0	0	0
Micronodular	0	0	0	0	0	0	0	0	0
Basosquamous	0	0	0	0	0	0	0	0	0
Other	0	0	0	0	0	0	0	0	0
Metastatic Site(s) [2]									
n	0	0	0	0	0	0	0	0	0
Bone	0	0	0	0	0	0	0	0	0
Lymph Nodes, Local	0	0	0	0	0	0	0	0	0
Lymph Nodes, Distant	0	0	0	0	0	0	0	0	0
Lung	0	0	0	0	0	0	0	0	0
Liver	0	0	0	0	0	0	0	0	0
CNS/Brain	0	0	0	0	0	0	0	0	0
Other	0	0	0	0	0	0	0	0	0
					Cohort 1: Newly Determined Advanced BCC Patients (Non-BCCNS) Metastatic BCC (mBCC)				
		Vismo (N=2)			No Vismo (N=1)				

Disease Assessment at Baseline: Metastatic Disease	All (N=4)	All (N=2)	Vismo Only (N=2)	Surgery Only (N=0)	Other Therapy (N=0)	All (N=1)	Surgery Only (N=1)	Other Therapy (N=0)	No Treatment (N=1)
Histopathology of Current mBCC [2]									
n	4	2	2	0	0	1	1	0	1
Nodular	3(75.0%)	1(50.0%)	1(50.0%)	0	0	1(100%)	1(100%)	0	1(100%)
Superficial	0	0	0	0	0	0	0	0	0
Morpheiform / Infiltrative	0	0	0	0	0	0	0	0	0
Micronodular	1(25.0%)	0	0	0	0	1(100%)	1(100%)	0	0
Basosquamous	2(50.0%)	1(50.0%)	1(50.0%)	0	0	1(100%)	1(100%)	0	0
Other	0	0	0	0	0	0	0	0	0
Metastatic Site(s) [2]									
n	3	1	1	0	0	1	1	0	1
Bone	1(33.3%)	0	0	0	0	0	0	0	1(100%)
Lymph Nodes, Local	0	0	0	0	0	0	0	0	0
Lymph Nodes, Distant	0	0	0	0	0	0	0	0	0
Lung	2(66.7%)	1(100%)	1(100%)	0	0	0	0	0	1(100%)
Liver	0	0	0	0	0	0	0	0	0
CNS/Brain	0	0	0	0	0	0	0	0	0
Other	1(33.3%)	0	0	0	0	1(100%)	1(100%)	0	0

					Cohort 1: Newly Determined Advanced BCC Patients (Non-BCCNS) Locally Advanced BCC (laBCC)				
			Vismo (N=115)		No Vismo (N=251)				
Disease Assessment at Baseline: Locally Advanced Target Lesion Assessment	All (N=433)	All (N=115)	Vismo Only (N=102)	Surgery Only (N=6)	Other Therapy (N=7)	All (N=251)	Surgery Only (N=198)	Other Therapy (N=53)	No Treatment (N=67)

Target Lesion Location on Body									
n	429	114	101	6	7	249	196	53	66
Eye	21(4.9%)	7(6.1%)	7(6.9%)	0	0	9(3.6%)	8(4.1%)	1(1.9%)	5(7.6%)
Hand	0	0	0	0	0	0	0	0	0
Foot	1(0.2%)	1(0.9%)	1(1.0%)	0	0	0	0	0	0
Shoulder	16(3.7%)	2(1.8%)	2(2.0%)	0	0	10(4.0%)	5(2.6%)	5(9.4%)	4(6.1%)
Scalp	26(6.1%)	7(6.1%)	6(5.9%)	1(16.7%)	0	15(6.0%)	12(6.1%)	3(5.7%)	4(6.1%)
Ear	38(8.9%)	8(7.0%)	7(6.9%)	1(16.7%)	0	27(10.8%)	22(11.2%)	5(9.4%)	3(4.5%)
Cheek	35(8.2%)	9(7.9%)	8(7.9%)	0	1(14.3%)	21(8.4%)	12(6.1%)	9(17.0%)	5(7.6%)
Nose	85(19.8%)	20(17.5%)	17(16.8%)	1(16.7%)	2(28.6%)	53(21.3%)	47(24.0%)	6(11.3%)	12(18.2%)
Forehead	50(11.7%)	14(12.3%)	13(12.9%)	1(16.7%)	0	31(12.4%)	26(13.3%)	5(9.4%)	5(7.6%)
Chin	6(1.4%)	0	0	0	0	4(1.6%)	4(2.0%)	0	2(3.0%)
Neck	12(2.8%)	3(2.6%)	3(3.0%)	0	0	7(2.8%)	6(3.1%)	1(1.9%)	2(3.0%)
Back	34(7.9%)	16(14.0%)	13(12.9%)	1(16.7%)	2(28.6%)	14(5.6%)	11(5.6%)	3(5.7%)	4(6.1%)
Chest	19(4.4%)	5(4.4%)	4(4.0%)	0	1(14.3%)	11(4.4%)	6(3.1%)	5(9.4%)	3(4.5%)
Abdomen	3(0.7%)	0	0	0	0	2(0.8%)	2(1.0%)	0	1(1.5%)
Arm	14(3.3%)	2(1.8%)	2(2.0%)	0	0	10(4.0%)	7(3.6%)	3(5.7%)	2(3.0%)
Leg	19(4.4%)	6(5.3%)	6(5.9%)	0	0	8(3.2%)	4(2.0%)	4(7.5%)	5(7.6%)
Other	50(11.7%)	14(12.3%)	12(11.9%)	1(16.7%)	1(14.3%)	27(10.8%)	24(12.2%)	3(5.7%)	9(13.6%)
						Cohort 1: Newly Determined Advanced BCC Patients (Non-BCCNS) Metastatic BCC (mBCC)			
			Vismo (N=2)			No Vismo (N=1)			
Disease Assessment at Baseline: Locally Advanced Target Lesion Assessment	All (N=4)	All (N=2)	Vismo Only (N=2)	Surgery Only (N=0)	Other Therapy (N=0)	All (N=1)	Surgery Only (N=1)	Other Therapy (N=0)	No Treatment (N=1)
Target Lesion Location on Body									
n	0	0	0	0	0	0	0	0	0
Eye	0	0	0	0	0	0	0	0	0
Hand	0	0	0	0	0	0	0	0	0
Foot	0	0	0	0	0	0	0	0	0
Shoulder	0	0	0	0	0	0	0	0	0
Scalp	0	0	0	0	0	0	0	0	0
Ear	0	0	0	0	0	0	0	0	0
Cheek	0	0	0	0	0	0	0	0	0
Nose	0	0	0	0	0	0	0	0	0
Forehead	0	0	0	0	0	0	0	0	0
Chin	0	0	0	0	0	0	0	0	0

Neck	0	0	0	0	0	0	0	0	0
Back	0	0	0	0	0	0	0	0	0
Chest	0	0	0	0	0	0	0	0	0
Abdomen	0	0	0	0	0	0	0	0	0
Arm	0	0	0	0	0	0	0	0	0
Leg	0	0	0	0	0	0	0	0	0
Other	0	0	0	0	0	0	0	0	0
					Cohort 1: Newly Determined Advanced BCC Patients (Non-BCCNS) Locally Advanced BCC (laBCC)				
			Vismo (N=115)			No Vismo (N=251)			
Disease Assessment at Baseline: Locally Advanced Target Lesion Assessment	All (N=433)	All (N=115)	Vismo Only (N=102)	Surgery Only (N=6)	Other Therapy (N=7)	All (N=251)	Surgery Only (N=198)	Other Therapy (N=53)	No Treatment (N=67)
Overall Lesion Location on Body [3]									
n	429	114	101	6	7	249	196	53	66
Eye	22(5.1%)	8(7.0%)	8(7.9%)	0	0	9(3.6%)	8(4.1%)	1(1.9%)	5(7.6%)
Hand	6(1.4%)	2(1.8%)	2(2.0%)	0	0	4(1.6%)	2(1.0%)	2(3.8%)	0
Foot	2(0.5%)	1(0.9%)	1(1.0%)	0	0	1(0.4%)	1(0.5%)	0	0
Shoulder	28(6.5%)	11(9.6%)	8(7.9%)	2(33.3%)	1(14.3%)	12(4.8%)	8(4.1%)	4(7.5%)	5(7.6%)
Scalp	32(7.5%)	11(9.6%)	10(9.9%)	1(16.7%)	0	17(6.8%)	13(6.6%)	4(7.5%)	4(6.1%)
Ear	50(11.7%)	11(9.6%)	10(9.9%)	1(16.7%)	0	33(13.3%)	24(12.2%)	9(17.0%)	6(9.1%)
Cheek	54(12.6%)	17(14.9%)	15(14.9%)	1(16.7%)	1(14.3%)	26(10.4%)	16(8.2%)	10(18.9%)	11(16.7%)
Nose	97(22.6%)	25(21.9%)	20(19.8%)	3(50.0%)	2(28.6%)	57(22.9%)	50(25.5%)	7(13.2%)	15(22.7%)
Forehead	60(14.0%)	23(20.2%)	20(19.8%)	3(50.0%)	0	30(12.0%)	25(12.8%)	5(9.4%)	7(10.6%)
Chin	12(2.8%)	3(2.6%)	3(3.0%)	0	0	5(2.0%)	4(2.0%)	1(1.9%)	4(6.1%)
Neck	28(6.5%)	12(10.5%)	8(7.9%)	2(33.3%)	2(28.6%)	11(4.4%)	9(4.6%)	2(3.8%)	5(7.6%)
Back	62(14.5%)	28(24.6%)	23(22.8%)	2(33.3%)	3(42.9%)	26(10.4%)	18(9.2%)	8(15.1%)	8(12.1%)
Chest	37(8.6%)	13(11.4%)	11(10.9%)	1(16.7%)	1(14.3%)	18(7.2%)	11(5.6%)	7(13.2%)	6(9.1%)
Abdomen	8(1.9%)	4(3.5%)	3(3.0%)	1(16.7%)	0	3(1.2%)	3(1.5%)	0	1(1.5%)
Arm	34(7.9%)	14(12.3%)	12(11.9%)	1(16.7%)	1(14.3%)	17(6.8%)	8(4.1%)	9(17.0%)	3(4.5%)
Leg	31(7.2%)	11(9.6%)	10(9.9%)	1(16.7%)	0	13(5.2%)	6(3.1%)	7(13.2%)	7(10.6%)
Other	52(12.1%)	12(10.5%)	10(9.9%)	1(16.7%)	1(14.3%)	31(12.4%)	28(14.3%)	3(5.7%)	9(13.6%)
					Cohort 1: Newly Determined Advanced BCC Patients (Non-BCCNS) Metastatic BCC (mBCC)				
			Vismo (N=2)			No Vismo (N=1)			

Disease Assessment at Baseline: Locally Advanced Target Lesion Assessment	All (N=4)	All (N=2)	Vismo Only (N=2)	Surgery Only (N=0)	Other Therapy (N=0)	All (N=1)	Surgery Only (N=1)	Other Therapy (N=0)	No Treatment (N=1)
Overall Lesion Location on Body [3]									
n	0	0	0	0	0	0	0	0	0
Eye	0	0	0	0	0	0	0	0	0
Hand	0	0	0	0	0	0	0	0	0
Foot	0	0	0	0	0	0	0	0	0
Shoulder	0	0	0	0	0	0	0	0	0
Scalp	0	0	0	0	0	0	0	0	0
Ear	0	0	0	0	0	0	0	0	0
Cheek	0	0	0	0	0	0	0	0	0
Nose	0	0	0	0	0	0	0	0	0
Forehead	0	0	0	0	0	0	0	0	0
Chin	0	0	0	0	0	0	0	0	0
Neck	0	0	0	0	0	0	0	0	0
Back	0	0	0	0	0	0	0	0	0
Chest	0	0	0	0	0	0	0	0	0
Abdomen	0	0	0	0	0	0	0	0	0
Arm	0	0	0	0	0	0	0	0	0
Leg	0	0	0	0	0	0	0	0	0
Other	0	0	0	0	0	0	0	0	0

					Cohort 1: Newly Determined Advanced BCC Patients (Non-BCCNS) Locally Advanced BCC (laBCC)				
		Vismo (N=115)			No Vismo (N=251)				
Disease Assessment at Baseline: Locally Advanced Target Lesion Assessment	All (N=433)	All (N=115)	Vismo Only (N=102)	Surgery Only (N=6)	Other Therapy (N=7)	All (N=251)	Surgery Only (N=198)	Other Therapy (N=53)	No Treatment (N=67)
Size (mm)									
n	415	107	94	6	7	245	193	52	63
Mean (SD)	30.4 (36.94)	38.2 (37.58)	38.7 (37.88)	21.0 (14.64)	47.0 (46.38)	28.7 (39.26)	30.2 (42.56)	23.3 (22.68)	23.3 (21.56)

Median	20.0	30.0	30.0	16.5	32.0	20.0	20.0	18.5	17.0
Range	(0, 456)	(0, 260)	(0, 260)	(10, 50)	(18, 150)	(0, 456)	(0, 456)	(2, 110)	(1, 100)
Histopathology [2]									
n	390	104	93	6	5	228	191	37	58
Nodular	252(64.6%)	66(63.5%)	60(64.5%)	4(66.7%)	2(40.0%)	144(63.2%)	122(63.9%)	22(59.5%)	42(72.4%)
Superficial	59(15.1%)	12(11.5%)	11(11.8%)	1(16.7%)	0	38(16.7%)	30(15.7%)	8(21.6%)	9(15.5%)
Morpheiform / Infiltrative	107(27.4%)	34(32.7%)	28(30.1%)	2(33.3%)	4(80.0%)	62(27.2%)	51(26.7%)	11(29.7%)	11(19.0%)
Micronodular	13(3.3%)	5(4.8%)	5(5.4%)	0	0	7(3.1%)	6(3.1%)	1(2.7%)	1(1.7%)
Basosquamous	10(2.6%)	5(4.8%)	5(5.4%)	0	0	4(1.8%)	3(1.6%)	1(2.7%)	1(1.7%)
Other	40(10.3%)	8(7.7%)	8(8.6%)	0	0	23(10.1%)	18(9.4%)	5(13.5%)	9(15.5%)

					Cohort 1: Newly Determined Advanced BCC Patients (Non-BCCNS) Metastatic BCC (mBCC)				
		Vismo (N=2)			No Vismo (N=1)				
Disease Assessment at Baseline: Locally Advanced Target Lesion Assessment	All (N=4)	All (N=2)	Vismo Only (N=2)	Surgery Only (N=0)	Other Therapy (N=0)	All (N=1)	Surgery Only (N=1)	Other Therapy (N=0)	No Treatment (N=1)
Size (mm)									
n	0	0	0	0	0	0	0	0	0
Mean (SD)									
Median									
Range									
Histopathology [2]									
n	0	0	0	0	0	0	0	0	0
Nodular	0	0	0	0	0	0	0	0	0
Superficial	0	0	0	0	0	0	0	0	0
Morpheiform / Infiltrative	0	0	0	0	0	0	0	0	0
Micronodular	0	0	0	0	0	0	0	0	0

Basosquamous	0	0	0	0	0	0	0	0	0
Other	0	0	0	0	0	0	0	0	0

Note: “Vismo” group is defined as initiating vismodegib less than or equal to 90 days after the date of determination of locally advanced BCC (laBCC) or metastatic BCC (mBCC); “No Vismo” group is defined as not initiating vismodegib (but other BCC treatment) less than or equal to 90 days after the date of determination of laBCC or mBCC; “No Treatment” group is defined as not receiving any BCC treatment less than or equal to 90 days after the date of determination of laBCC or mBCC.

“Vismo Only” and “Surgery Only” are defined as having only 1 of these treatments less than or equal to 90 days after the date of determination of laBCC or mBCC; “Other Therapy” includes individual or combination treatments (e.g., surgery, PDT, ED&C, topical treatment, cryosurgery, systemic treatment, other) initiated less than or equal to 90 days after date of determination of laBCC or mBCC. For example, if patient receives topical treatment followed by vismo followed by surgery, then all 3 treatments must be initiated within the 90 days of the date of determination of disease.

Denominator for the response rates is the number of patients with the best overall response, unless specified otherwise.

[1] Duration of response is defined as the time from the initial recorded complete or partial response to the last recorded disease assessment date prior to documented disease progression or death. For patients who do not progress or die after achieving a response, DOR will be censored at the last recorded disease assessment date. This analysis will be performed for patients who achieved a response only.

[2] Progression free survival is defined as the time from date of study enrollment to documented disease progression or death from any cause while on study. Patients who are progression-free at the end of the study period will be censored at the last recorded disease assessment date.

[3] Overall survival is defined as the time from enrollment to death of any cause. Patients not died are censored at treatment termination or cutoff date. Death after treatment termination is treated as event.

[4] Denominator consists of all patients for whom Best Response Prior to 1st PD on study is characterized.

[5] Duration of BCC treatment is defined as the time from the initial exposure to the last exposure to any BCC treatment.

[6] Duration of vismodegib is defined as the sum of the time from the initial exposure to the last exposure to each Vismodegib treatment for each subject.

[7] Any Vismodegib treatment records with completely or partially missing end dates are excluded from the calculation.

[8] Any Vismodegib treatment records with completely or partially missing end dates are imputed per section 4.7 in SAP. If patient is exposed to any BCC treatments other than vismoegib within the time above, the time on other BCC treatments will be excluded from calculation.

[9] Denominator is the number of patients in the column header.

					Cohort 1: Newly Determined Advanced BCC Patients (Non-BCCNS) Locally Advanced BCC (laBCC)				
			Vismo (N=115)			No Vismo (N=251)			
	All (N=433)	All (N=115)	Vismo Only (N=102)	Surgery Only (N=6)	Other Therapy (N=7)	All (N=251)	Surgery Only (N=198)	Other Therapy (N=53)	No Treatment (N=67)
Best Overall Response									
n	326	101	90	5	6	198	154	44	27
Complete Response (CR)	263(80.7%)	64(63.4%)	55(61.1%)	4(80.0%)	5(83.3%)	181(91.4%)	141(91.6%)	40(90.9%)	18(66.7%)
Partial Response (PR)	33(10.1%)	22(21.8%)	21(23.3%)	0	1(16.7%)	7(3.5%)	3(1.9%)	4(9.1%)	4(14.8%)
Stable Disease (SD)	27(8.3%)	14(13.9%)	13(14.4%)	1(20.0%)	0	8(4.0%)	8(5.2%)	0	5(18.5%)
Progressive Disease (PD)	1(0.3%)	1(1.0%)	1(1.1%)	0	0	0	0	0	0
Recurrence	0	0	0	0	0	0	0	0	0
Clinical Deterioration	0	0	0	0	0	0	0	0	0
Unable to Evaluate	2(0.6%)	0	0	0	0	2(1.0%)	2(1.3%)	0	0

Response Rate (CR/PR)	296(90.8%)	86(85.1%)	76(84.4%)	4(80.0%)	6(100%)	188(94.9%)	144(93.5%)	44(100%)	22(81.5%)
95% CI for Response Rate	(0.87, 0.94)	(0.77, 0.91)	(0.76, 0.91)	(0.34, 0.99)	(0.60, 1.00)	(0.91, 0.97)	(0.89, 0.97)	(0.93, 1.00)	(0.64, 0.92)
Disease Control Rate (CR/PR/SD)	323(99.1%)	100(99.0%)	89(98.9%)	5(100%)	6(100%)	196(99.0%)	152(98.7%)	44(100%)	27(100%)
95% CI for Disease Control Rate	(0.97, 1.00)	(0.95, 1.00)	(0.95, 1.00)	(0.50, 1.00)	(0.60, 1.00)	(0.96, 1.00)	(0.95, 1.00)	(0.93, 1.00)	(0.89, 1.00)
Patients with Recurrence on the Study	30(9.2%)	24(23.8%)	22(24.4%)	1(20.0%)	1(16.7%)	4(2.0%)	2(1.3%)	2(4.5%)	2(7.4%)
95% CI for Recurrence Rate	(0.06, 0.13)	(0.16, 0.33)	(0.16, 0.34)	(0.01, 0.66)	(0.01, 0.60)	(0.01, 0.05)	(0.00, 0.05)	(0.01, 0.15)	(0.01, 0.22)
Patients with Recurrence within 6 Months from Enrollment	2(0.6%)	0	0	0	0	0	0	0	2(7.4%)
95% CI for Recurrence Rate	(0.00, 0.02)	(0.00, 0.03)	(0.00, 0.04)	(0.00, 0.50)	(0.00, 0.40)	(0.00, 0.02)	(0.00, 0.02)	(0.00, 0.07)	(0.01, 0.22)
Patients with Recurrence within 1 Year from Enrollment	9(2.8%)	4(4.0%)	4(4.4%)	0	0	3(1.5%)	2(1.3%)	1(2.3%)	2(7.4%)
95% CI for Recurrence Rate	(0.01, 0.05)	(0.01, 0.09)	(0.02, 0.11)	(0.00, 0.50)	(0.00, 0.40)	(0.00, 0.04)	(0.00, 0.05)	(0.00, 0.11)	(0.01, 0.22)
					Cohort 1: Newly Determined Advanced BCC Patients (Non-BCCNS) Metastatic BCC (mBCC)				
			Vismo (N=2)			No Vismo (N=1)			
	All (N=4)	All (N=2)	Vismo Only (N=2)	Surgery Only (N=0)	Other Therapy (N=0)	All (N=1)	Surgery Only (N=1)	Other Therapy (N=0)	No Treatment (N=1)
Best Overall Response									
n	4	2	2	0	0	1	1	0	1

Complete Response (CR)	2(50.0%)	0	0	0	0	1(100%)	1(100%)	0	1(100%)
Partial Response (PR)	1(25.0%)	1(50.0%)	1(50.0%)	0	0	0	0	0	0
Stable Disease (SD)	1(25.0%)	1(50.0%)	1(50.0%)	0	0	0	0	0	0
Progressive Disease (PD)	0	0	0	0	0	0	0	0	0
Recurrence	0	0	0	0	0	0	0	0	0
Clinical Deterioration	0	0	0	0	0	0	0	0	0
Unable to Evaluate	0	0	0	0	0	0	0	0	0
Response Rate (CR/PR)	3(75.0%)	1(50.0%)	1(50.0%)	0	0	1(100%)	1(100%)	0	1(100%)
95% CI for Response Rate	(0.25, 0.99)	(0.03, 0.97)	(0.03, 0.97)			(0.05, 1.00)	(0.05, 1.00)		(0.05, 1.00)
Disease Control Rate (CR/PR/SD)	4(100%)	2(100%)	2(100%)	0	0	1(100%)	1(100%)	0	1(100%)
95% CI for Disease Control Rate	(0.47, 1.00)	(0.22, 1.00)	(0.22, 1.00)			(0.05, 1.00)	(0.05, 1.00)		(0.05, 1.00)
Patients with Recurrence on the Study	0	0	0	0	0	0	0	0	0
95% CI for Recurrence Rate	(0.00, 0.53)	(0.00, 0.78)	(0.00, 0.78)			(0.00, 0.95)	(0.00, 0.95)		(0.00, 0.95)
Patients with Recurrence within 6 Months from Enrollment	0	0	0	0	0	0	0	0	0
95% CI for Recurrence Rate	(0.00, 0.53)	(0.00, 0.78)	(0.00, 0.78)			(0.00, 0.95)	(0.00, 0.95)		(0.00, 0.95)
Patients with Recurrence within 1 Year from Enrollment	0	0	0	0	0	0	0	0	0

95% CI for Recurrence Rate	(0.00, 0.53)	(0.00, 0.78)	(0.00, 0.78)			(0.00, 0.95)	(0.00, 0.95)		(0.00, 0.95)
					Cohort 1: Newly Determined Advanced BCC Patients (Non-BCCNS) Locally Advanced BCC (laBCC)				
			Vismo (N=115)		No Vismo (N=251)				
	All (N=433)	All (N=115)	Vismo Only (N=102)	Surgery Only (N=6)	Other Therapy (N=7)	All (N=251)	Surgery Only (N=198)	Other Therapy (N=53)	No Treatment (N=67)
Patients with PD on the Study	39(12.0%)	20(19.8%)	18(20.0%)	0	2(33.3%)	15(7.6%)	8(5.2%)	7(15.9%)	4(14.8%)
Patients with Death [9]	37(8.5%)	16(13.9%)	14(13.7%)	0	2(28.6%)	14(5.6%)	9(4.5%)	5(9.4%)	7(10.4%)
Duration of Response (Months) [1]									
n	296	86	76	4	6	188	144	44	22
Mean (SD)	13.12 (11.483)	16.57 (10.740)	15.91 (10.781)	23.88 (6.976)	20.08 (11.151)	11.85 (11.702)	11.61 (11.943)	12.61 (10.972)	10.46 (9.760)
Median	11.96	17.49	15.92	26.58	20.25	8.89	7.82	10.92	9.33
Range	(0.03, 39.59)	(0.03, 35.32)	(0.03, 35.32)	(13.60, 28.75)	(6.64, 32.46)	(0.03, 39.59)	(0.03, 39.59)	(0.03, 37.98)	(0.03, 27.20)
Kaplan-Meier Median	NE	NE	NE	NE	NE	NE	NE	NE	NE
95% CI	(NE, NE)	(NE, NE)	(26.02, NE)	(NE, NE)	(6.64, NE)	(NE, NE)	(NE, NE)	(21.03, NE)	(NE, NE)
Progression Free Survival (Months) [2]									
Kaplan-Meier Median	NE	NE	NE	NE	21.39	NE	NE	NE	NE
95% CI	(NE, NE)	(23.49, NE)	(23.49, NE)	(NE, NE)	(0.16, NE)	(NE, NE)	(NE, NE)	(19.65, NE)	(30.69, NE)
Overall Survival (Months) [3]									
Kaplan-Meier Median	NE	NE	NE	NE	NE	NE	NE	NE	NE
95% CI	(NE, NE)	(NE, NE)	(NE, NE)	(NE, NE)	(0.16, NE)	(NE, NE)	(NE, NE)	(NE, NE)	(NE, NE)
					Cohort 1: Newly Determined Advanced BCC Patients (Non-BCCNS) Metastatic BCC (mBCC)				
			Vismo (N=2)		No Vismo (N=1)				

	All (N=4)	All (N=2)	Vismo Only (N=2)	Surgery Only (N=0)	Other Therapy (N=0)	All (N=1)	Surgery Only (N=1)	Other Therapy (N=0)	No Treatment (N=1)
Patients with PD on the Study	1(25.0%)	1(50.0%)	1(50.0%)	0	0	0	0	0	0
Patients with Death [9]	0	0	0	0	0	0	0	0	0
Duration of Response (Months) [1]									
n	3	1	1	0	0	1	1	0	1
Mean (SD)	6.40 (4.867)	4.04(-)	4.04(-)			3.15(-)	3.15(-)		11.99(-)
Median	4.04	4.04	4.04			3.15	3.15		11.99
Range	(3.15, 11.99)	(4.04, 4.04)	(4.04, 4.04)			(3.15, 3.15)	(3.15, 3.15)		(11.99, 11.99)
Kaplan-Meier Median	NE	4.04	4.04			NE	NE		NE
95% CI	(4.04, NE)	(NE, NE)	(NE, NE)			(NE, NE)	(NE, NE)		(NE, NE)
Progression Free Survival (Months) [2]									
Kaplan-Meier Median	NE	NE	NE			NE	NE		NE
95% CI	(6.47, NE)	(6.47, NE)	(6.47, NE)			(NE, NE)	(NE, NE)		(NE, NE)
Overall Survival (Months) [3]									
Kaplan-Meier Median	NE	NE	NE			NE	NE		NE
95% CI	(NE, NE)	(NE, NE)	(NE, NE)			(NE, NE)	(NE, NE)		(NE, NE)
					Cohort 1: Newly Determined Advanced BCC Patients (Non-BCCNS) Locally Advanced BCC (laBCC)				
			Vismo (N=115)		No Vismo (N=251)				
	All (N=433)	All (N=115)	Vismo Only (N=102)	Surgery Only (N=6)	Other Therapy (N=7)	All (N=251)	Surgery Only (N=198)	Other Therapy (N=53)	No Treatment (N=67)

Best Response Prior to 1st PD on the Study									
n	334	97	86	5	6	198	153	45	39
Complete Response (CR)	265(79.3%)	64(66.0%)	55(64.0%)	4(80.0%)	5(83.3%)	179(90.4%)	140(91.5%)	39(86.7%)	22(56.4%)
Partial Response (PR)	33(9.9%)	20(20.6%)	19(22.1%)	0	1(16.7%)	8(4.0%)	2(1.3%)	6(13.3%)	5(12.8%)
Stable Disease (SD)	33(9.9%)	12(12.4%)	11(12.8%)	1(20.0%)	0	9(4.5%)	9(5.9%)	0	12(30.8%)
Progressive Disease (PD)	1(0.3%)	1(1.0%)	1(1.2%)	0	0	0	0	0	0
Recurrence	0	0	0	0	0	0	0	0	0
Clinical Deterioration	0	0	0	0	0	0	0	0	0
Unable to Evaluate	2(0.6%)	0	0	0	0	2(1.0%)	2(1.3%)	0	0
Response Rate (CR/PR) [4]	298(89.2%)	84(86.6%)	74(86.0%)	4(80.0%)	6(100%)	187(94.4%)	142(92.8%)	45(100%)	27(69.2%)
95% CI for Response Rate	(0.85, 0.92)	(0.79, 0.92)	(0.77, 0.92)	(0.34, 0.99)	(0.60, 1.00)	(0.90, 0.97)	(0.88, 0.96)	(0.93, 1.00)	(0.53, 0.83)
Disease Control Rate (CR/PR/SD) [4]	331(99.1%)	96(99.0%)	85(98.8%)	5(100%)	6(100%)	196(99.0%)	151(98.7%)	45(100%)	39(100%)
95% CI for Disease Control Rate	(0.97, 1.00)	(0.95, 1.00)	(0.94, 1.00)	(0.50, 1.00)	(0.60, 1.00)	(0.96, 1.00)	(0.95, 1.00)	(0.93, 1.00)	(0.92, 1.00)
					Cohort 1: Newly Determined Advanced BCC Patients (Non-BCCNS) Metastatic BCC (mBCC)				
			Vismo (N=2)			No Vismo (N=1)			
	All (N=4)	All (N=2)	Vismo Only (N=2)	Surgery Only (N=0)	Other Therapy (N=0)	All (N=1)	Surgery Only (N=1)	Other Therapy (N=0)	No Treatment (N=1)
Best Response Prior to 1st PD on the Study									
n	4	2	2	0	0	1	1	0	1
Complete Response (CR)	2(50.0%)	0	0	0	0	1(100%)	1(100%)	0	1(100%)
Partial Response (PR)	1(25.0%)	1(50.0%)	1(50.0%)	0	0	0	0	0	0

Stable Disease (SD)	1(25.0%)	1(50.0%)	1(50.0%)	0	0	0	0	0	0
Progressive Disease (PD)	0	0	0	0	0	0	0	0	0
Recurrence	0	0	0	0	0	0	0	0	0
Clinical Deterioration	0	0	0	0	0	0	0	0	0
Unable to Evaluate	0	0	0	0	0	0	0	0	0
Response Rate (CR/PR) [4]	3(75.0%)	1(50.0%)	1(50.0%)	0	0	1(100%)	1(100%)	0	1(100%)
95% CI for Response Rate	(0.25, 0.99)	(0.03, 0.97)	(0.03, 0.97)			(0.05, 1.00)	(0.05, 1.00)		(0.05, 1.00)
Disease Control Rate (CR/PR/SD) [4]	4(100%)	2(100%)	2(100%)	0	0	1(100%)	1(100%)	0	1(100%)
95% CI for Disease Control Rate	(0.47, 1.00)	(0.22, 1.00)	(0.22, 1.00)			(0.05, 1.00)	(0.05, 1.00)		(0.05, 1.00)

					Cohort 1: Newly Determined Advanced BCC Patients (Non-BCCNS) Locally Advanced BCC (laBCC)				
		Vismo (N=115)			No Vismo (N=251)				
	All (N=433)	All (N=115)	Vismo Only (N=102)	Surgery Only (N=6)	Other Therapy (N=7)	All (N=251)	Surgery Only (N=198)	Other Therapy (N=53)	No Treatment (N=67)
Duration of BCC Treatment (Months) [5]									
n	401	115	102	6	7	250	198	52	36
Mean (SD)	6.89 (9.277)	13.06 (9.376)	13.23 (9.232)	7.82 (5.478)	14.99 (13.343)	4.08 (8.025)	3.74 (7.986)	5.37 (8.123)	6.66 (7.601)
Median	1.74	11.79	12.14	7.80	8.44	0.03	0.03	2.74	1.63
Range	(0.03, 37.95)	(0.03, 36.27)	(0.03, 35.48)	(0.03, 16.79)	(0.16, 36.27)	(0.03, 37.95)	(0.03, 34.73)	(0.03, 37.95)	(0.03, 20.63)
Kaplan-Meier Median	2.79	14.13	15.06	7.80	8.44	0.03	0.03	3.45	2.33
95% CI	(1.41, 4.11)	(10.58, 18.00)	(12.29, 21.32)	(0.03, 16.79)	(0.16, 27.17)	(NE, NE)	(NE, NE)	(1.41, 4.40)	(0.03, 11.40)
Duration of Vismo (Months) [6]									

Without Imputation [7]									
n	128	115	102	6	7	5	4	1	8
Mean (SD)	7.98 (6.598)	8.01 (6.614)	7.98 (6.682)	6.86 (5.649)	9.45 (7.015)	7.96 (6.238)	7.82 (7.195)	8.51(-)	7.45 (7.397)
Median	6.24	6.31	6.24	6.47	8.11	6.24	4.99	8.51	4.29
Range	(0.03, 31.97)	(0.03, 31.97)	(0.03, 31.97)	(0.03, 16.56)	(0.10, 18.86)	(2.89, 18.40)	(2.89, 18.40)	(8.51, 8.51)	(0.03, 19.12)
Kaplan-Meier Median	8.10	8.08	8.13	6.47	8.11	6.24	4.99	8.51	15.20
95% CI	(6.74, 9.56)	(6.74, 9.56)	(6.87, 10.22)	(0.03, 16.56)	(0.10, 15.54)	(2.89, 18.40)	(2.89, 18.40)	(NE, NE)	(1.84, NE)
With Imputation [8]									
n	128	115	102	6	7	5	4	1	8
Mean (SD)	7.98 (6.598)	8.01 (6.614)	7.98 (6.682)	6.86 (5.649)	9.45 (7.015)	7.96 (6.238)	7.82 (7.195)	8.51(-)	7.45 (7.397)
Median	6.24	6.31	6.24	6.47	8.11	6.24	4.99	8.51	4.29
Range	(0.03, 31.97)	(0.03, 31.97)	(0.03, 31.97)	(0.03, 16.56)	(0.10, 18.86)	(2.89, 18.40)	(2.89, 18.40)	(8.51, 8.51)	(0.03, 19.12)
Kaplan-Meier Median	8.10	8.08	8.13	6.47	8.11	6.24	4.99	8.51	15.20
95% CI	(6.74, 9.56)	(6.74, 9.56)	(6.87, 10.22)	(0.03, 16.56)	(0.10, 15.54)	(2.89, 18.40)	(2.89, 18.40)	(NE, NE)	(1.84, NE)
						Cohort 1: Newly Determined Advanced BCC Patients (Non-BCCNS) Metastatic BCC (mBCC)			
			Vismo (N=2)			No Vismo (N=1)			
	All (N=4)	All (N=2)	Vismo Only (N=2)	Surgery Only (N=0)	Other Therapy (N=0)	All (N=1)	Surgery Only (N=1)	Other Therapy (N=0)	No Treatment (N=1)
Duration of BCC Treatment (Months) [5]									
n	4	2	2	0	0	1	1	0	1
Mean (SD)	7.28 (7.189)	9.31 (9.176)	9.31 (9.176)			0.03(-)	0.03(-)		10.45(-)
Median	6.64	9.31	9.31			0.03	0.03		10.45
Range	(0.03, 15.80)	(2.83, 15.80)	(2.83, 15.80)			(0.03, 0.03)	(0.03, 0.03)		(10.45, 10.45)
Kaplan-Meier Median	13.13	NE	NE			0.03	0.03		10.45
95% CI	(0.03, NE)	(15.80, NE)	(15.80, NE)			(NE, NE)	(NE, NE)		(NE, NE)
Duration of Vismo (Months) [6]									

Without Imputation [7]									
n	3	2	2	0	0	0	0	0	1
Mean (SD)	6.67 (3.795)	4.78 (2.718)	4.78 (2.718)						10.45(-)
Median	6.70	4.78	4.78						10.45
Range	(2.86, 10.45)	(2.86, 6.70)	(2.86, 6.70)						(10.45, 10.45)
Kaplan-Meier Median	10.45	NE	NE						10.45
95% CI	(6.70, NE)	(6.70, NE)	(6.70, NE)						(NE, NE)
With Imputation [8]									
n	3	2	2	0	0	0	0	0	1
Mean (SD)	6.67 (3.795)	4.78 (2.718)	4.78 (2.718)						10.45(-)
Median	6.70	4.78	4.78						10.45
Range	(2.86, 10.45)	(2.86, 6.70)	(2.86, 6.70)						(10.45, 10.45)
Kaplan-Meier Median	10.45	NE	NE						10.45
95% CI	(6.70, NE)	(6.70, NE)	(6.70, NE)						(NE, NE)

Note: “Vismo” group is defined as initiating vismodegib less than or equal to 90 days after the date of determination of locally advanced BCC (laBCC) or metastatic BCC (mBCC); “No Vismo” group is defined as not initiating vismodegib (but other BCC treatment) less than or equal to 90 days after the date of determination of laBCC or mBCC; “No Treatment” group is defined as not receiving any BCC treatment less than or equal to 90 days after the date of determination of laBCC or mBCC.

“Vismo Only” and “Surgery Only” are defined as having only 1 of these treatments less than or equal to 90 days after the date of determination of laBCC or mBCC; “Other Therapy” includes individual or combination treatments (e.g., surgery, PDT, ED&C, topical treatment, cryosurgery, systemic treatment, other) initiated less than or equal to 90 days after date of determination of laBCC or mBCC. For example, if patient receives topical treatment followed by vismo followed by surgery, then all 3 treatments must be initiated within the 90 days of the date of determination of disease.

[1] A patient may be counted in more than one category. The percentages may add up to more than 100%. The denominator is the number of patients who had a complete response at the follow-up visit.

[2] A patient may be counted in more than one category. The percentages may add up to more than 100%. The denominator is the number of patients who have progressed to metastatic disease or developed new metastases at the follow-up visit.

					Cohort 1: Newly Determined Advanced BCC Patients (Non-BCCNS) Locally Advanced BCC (laBCC)				
		Vismo (N=115)			No Vismo (N=251)				
	All (N=433)	All (N=115)	Vismo Only (N=102)	Surgery Only (N=6)	Other Therapy (N=7)	All (N=251)	Surgery Only (N=198)	Other Therapy (N=53)	No Treatment (N=67)
Routine Follow-up 1									
Response Assessment									
n	314	93	83	4	6	185	140	45	36
Complete Response (CR)	169(53.8%)	21(22.6%)	17(20.5%)	3(75.0%)	1(16.7%)	136(73.5%)	118(84.3%)	18(40.0%)	12(33.3%)
Partial Response (PR)	66(21.0%)	42(45.2%)	38(45.8%)	0	4(66.7%)	21(11.4%)	2(1.4%)	19(42.2%)	3(8.3%)
Stable Disease (SD)	57(18.2%)	22(23.7%)	20(24.1%)	1(25.0%)	1(16.7%)	17(9.2%)	12(8.6%)	5(11.1%)	18(50.0%)
Disease Progression (PD)	9(2.9%)	5(5.4%)	5(6.0%)	0	0	3(1.6%)	1(0.7%)	2(4.4%)	1(2.8%)
Recurrence	4(1.3%)	1(1.1%)	1(1.2%)	0	0	1(0.5%)	0	1(2.2%)	2(5.6%)
Clinical Deterioration	1(0.3%)	0	0	0	0	1(0.5%)	1(0.7%)	0	0
Unable to Evaluate	8(2.5%)	2(2.2%)	2(2.4%)	0	0	6(3.2%)	6(4.3%)	0	0
If Complete Response, as Determined by[1]									
Negative Histopathology	37(21.9%)	2(9.5%)	2(11.8%)	0	0	32(23.5%)	28(23.7%)	4(22.2%)	3(25.0%)

Complete Tumor Shrinkage	24(14.2%)	13(61.9%)	11(64.7%)	1(33.3%)	1(100%)	11(8.1%)	6(5.1%)	5(27.8%)	0
No Clinically Visible Evidence of BCC	146(86.4%)	13(61.9%)	9(52.9%)	3(100%)	1(100%)	122(89.7%)	106(89.8%)	16(88.9%)	11(91.7%)
Other	6(3.6%)	2(9.5%)	2(11.8%)	0	0	3(2.2%)	3(2.5%)	0	1(8.3%)
					Cohort 1: Newly Determined Advanced BCC Patients (Non-BCCNS) Metastatic BCC (mBCC)				
		Vismo (N=2)				No Vismo (N=1)			
	All (N=4)	All (N=2)	Vismo Only (N=2)	Surgery Only (N=0)	Other Therapy (N=0)	All (N=1)	Surgery Only (N=1)	Other Therapy (N=0)	No Treatment (N=1)
Routine Follow-up 1									
Response Assessment									
n	4	2	2	0	0	1	1	0	1
Complete Response (CR)	1(25.0%)	0	0	0	0	1(100%)	1(100%)	0	0
Partial Response (PR)	2(50.0%)	1(50.0%)	1(50.0%)	0	0	0	0	0	1(100%)
Stable Disease (SD)	1(25.0%)	1(50.0%)	1(50.0%)	0	0	0	0	0	0
Disease Progression (PD)	0	0	0	0	0	0	0	0	0
Recurrence	0	0	0	0	0	0	0	0	0
Clinical Deterioration	0	0	0	0	0	0	0	0	0
Unable to Evaluate	0	0	0	0	0	0	0	0	0
If Complete Response, as Determined by[1]									
Negative Histopathology	0	0	0	0	0	0	0	0	0
Complete Tumor Shrinkage	1(100%)	0	0	0	0	1(100%)	1(100%)	0	0

No Clinically Visible Evidence of BCC	0	0	0	0	0	0	0	0	0
Other	1(100%)	0	0	0	0	1(100%)	1(100%)	0	0
					Cohort 1: Newly Determined Advanced BCC Patients (Non-BCCNS) Locally Advanced BCC (laBCC)				
			Vismo (N=115)			No Vismo (N=251)			
	All (N=433)	All (N=115)	Vismo Only (N=102)	Surgery Only (N=6)	Other Therapy (N=7)	All (N=251)	Surgery Only (N=198)	Other Therapy (N=53)	No Treatment (N=67)
Indicate How Response/ Progression was Assessed:									
n	137	61	57	2	2	62	45	17	14
Change in Size of Existing Lesions	108(78.8%)	54(88.5%)	50(87.7%)	2(100%)	2(100%)	46(74.2%)	31(68.9%)	15(88.2%)	8(57.1%)
Change in Number of Existing Lesions	17(12.4%)	9(14.8%)	9(15.8%)	0	0	5(8.1%)	4(8.9%)	1(5.9%)	3(21.4%)
Development of New BCCs	14(10.2%)	3(4.9%)	3(5.3%)	0	0	7(11.3%)	6(13.3%)	1(5.9%)	4(28.6%)
Progression from Locally Advanced Disease to Metastatic Disease	3(2.2%)	2(3.3%)	2(3.5%)	0	0	1(1.6%)	1(2.2%)	0	0
Development of New Metastases	0	0	0	0	0	0	0	0	0
Other	16(11.7%)	5(8.2%)	5(8.8%)	0	0	8(12.9%)	8(17.8%)	0	3(21.4%)
If progression to metastatic disease or new metastases, indicate site(s)[2]									
Bone	1(33.3%)	1(50.0%)	1(50.0%)	0	0	0	0	0	0
Lymph nodes, Local	0	0	0	0	0	0	0	0	0

Lymph nodes, Distant	1(33.3%)	1(50.0%)	1(50.0%)	0	0	0	0	0	0
Lung	0	0	0	0	0	0	0	0	0
Liver	1(33.3%)	1(50.0%)	1(50.0%)	0	0	0	0	0	0
CNS/Brain	0	0	0	0	0	0	0	0	0
Other	0	0	0	0	0	0	0	0	0
What Methods were Used to Assess Disease Response/ Progression									
n	314	93	83	4	6	185	140	45	36
Diagnostic Image (MRI, CT)	10(3.2%)	8(8.6%)	6(7.2%)	1(25.0%)	1(16.7%)	2(1.1%)	2(1.4%)	0	0
Histopathologic Evaluation	42(13.4%)	6(6.5%)	6(7.2%)	0	0	31(16.8%)	26(18.6%)	5(11.1%)	5(13.9%)
Photography	35(11.1%)	26(28.0%)	25(30.1%)	1(25.0%)	0	4(2.2%)	3(2.1%)	1(2.2%)	5(13.9%)
Clinical Evaluation	286(91.1%)	87(93.5%)	78(94.0%)	4(100%)	5(83.3%)	167(90.3%)	124(88.6%)	43(95.6%)	32(88.9%)
Laboratory Evaluation	9(2.9%)	4(4.3%)	3(3.6%)	1(25.0%)	0	5(2.7%)	4(2.9%)	1(2.2%)	0
Other	10(3.2%)	5(5.4%)	5(6.0%)	0	0	5(2.7%)	5(3.6%)	0	0

					Cohort 1: Newly Determined Advanced BCC Patients (Non-BCCNS) Metastatic BCC (mBCC)				
			Vismo (N=2)		No Vismo (N=1)				
	All (N=4)	All (N=2)	Vismo Only (N=2)	Surgery Only (N=0)	Other Therapy (N=0)	All (N=1)	Surgery Only (N=1)	Other Therapy (N=0)	No Treatment (N=1)
Indicate How Response/ Progression was Assessed:									
n	2	1	1	0	0	1	1	0	0
Change in Size of Existing Lesions	2(100%)	1(100%)	1(100%)	0	0	1(100%)	1(100%)	0	0
Change in Number of Existing Lesions	0	0	0	0	0	0	0	0	0

Development of New BCCs	0	0	0	0	0	0	0	0	0
Progression from Locally Advanced Disease to Metastatic Disease	0	0	0	0	0	0	0	0	0
Development of New Metastases	0	0	0	0	0	0	0	0	0
Other	0	0	0	0	0	0	0	0	0
If progression to metastatic disease or new metastases, indicate site(s)[2]									
Bone	0	0	0	0	0	0	0	0	0
Lymph nodes, Local	0	0	0	0	0	0	0	0	0
Lymph nodes, Distant	0	0	0	0	0	0	0	0	0
Lung	0	0	0	0	0	0	0	0	0
Liver	0	0	0	0	0	0	0	0	0
CNS/Brain	0	0	0	0	0	0	0	0	0
Other	0	0	0	0	0	0	0	0	0
What Methods were Used to Assess Disease Response/ Progression									
n	4	2	2	0	0	1	1	0	1
Diagnostic Image (MRI, CT)	1(25.0%)	1(50.0%)	1(50.0%)	0	0	0	0	0	0
Histopathologic Evaluation	0	0	0	0	0	0	0	0	0
Photography	1(25.0%)	0	0	0	0	0	0	0	1(100%)
Clinical Evaluation	4(100%)	2(100%)	2(100%)	0	0	1(100%)	1(100%)	0	1(100%)

Laboratory Evaluation	0	0	0	0	0	0	0	0	0
Other	0	0	0	0	0	0	0	0	0
					Cohort 1: Newly Determined Advanced BCC Patients (Non-BCCNS) Locally Advanced BCC (laBCC)				
			Vismo (N=115)		No Vismo (N=251)				
	All (N=433)	All (N=115)	Vismo Only (N=102)	Surgery Only (N=6)	Other Therapy (N=7)	All (N=251)	Surgery Only (N=198)	Other Therapy (N=53)	No Treatment (N=67)
Routine Follow-up 2									
Response Assessment									
n	265	89	79	4	6	147	109	38	29
Complete Response (CR)	160(60.4%)	31(34.8%)	26(32.9%)	3(75.0%)	2(33.3%)	117(79.6%)	92(84.4%)	25(65.8%)	12(41.4%)
Partial Response (PR)	58(21.9%)	40(44.9%)	37(46.8%)	0	3(50.0%)	12(8.2%)	3(2.8%)	9(23.7%)	6(20.7%)
Stable Disease (SD)	30(11.3%)	16(18.0%)	14(17.7%)	1(25.0%)	1(16.7%)	7(4.8%)	6(5.5%)	1(2.6%)	7(24.1%)
Disease Progression (PD)	9(3.4%)	1(1.1%)	1(1.3%)	0	0	6(4.1%)	3(2.8%)	3(7.9%)	2(6.9%)
Recurrence	2(0.8%)	1(1.1%)	1(1.3%)	0	0	0	0	0	1(3.4%)
Clinical Deterioration	0	0	0	0	0	0	0	0	0
Unable to Evaluate	6(2.3%)	0	0	0	0	5(3.4%)	5(4.6%)	0	1(3.4%)
If Complete Response, as Determined by[1]									
Negative Histopathology	27(16.9%)	4(12.9%)	4(15.4%)	0	0	22(18.8%)	16(17.4%)	6(24.0%)	1(8.3%)
Complete Tumor Shrinkage	19(11.9%)	14(45.2%)	11(42.3%)	1(33.3%)	2(100%)	5(4.3%)	1(1.1%)	4(16.0%)	0
No Clinically Visible Evidence of BCC	145(90.6%)	26(83.9%)	21(80.8%)	3(100%)	2(100%)	108(92.3%)	84(91.3%)	24(96.0%)	11(91.7%)
Other	1(0.6%)	0	0	0	0	1(0.9%)	1(1.1%)	0	0
					Cohort 1: Newly Determined Advanced BCC Patients (Non-BCCNS) Metastatic BCC (mBCC)				
			Vismo (N=2)		No Vismo (N=1)				

	All (N=4)	All (N=2)	Vismo Only (N=2)	Surgery Only (N=0)	Other Therapy (N=0)	All (N=1)	Surgery Only (N=1)	Other Therapy (N=0)	No Treatment (N=1)
Routine Follow-up 2									
Response Assessment									
n	4	2	2	0	0	1	1	0	1
Complete Response (CR)	2(50.0%)	0	0	0	0	1(100%)	1(100%)	0	1(100%)
Partial Response (PR)	0	0	0	0	0	0	0	0	0
Stable Disease (SD)	2(50.0%)	2(100%)	2(100%)	0	0	0	0	0	0
Disease Progression (PD)	0	0	0	0	0	0	0	0	0
Recurrence	0	0	0	0	0	0	0	0	0
Clinical Deterioration	0	0	0	0	0	0	0	0	0
Unable to Evaluate	0	0	0	0	0	0	0	0	0
If Complete Response, as Determined by[1]									
Negative Histopathology	0	0	0	0	0	0	0	0	0
Complete Tumor Shrinkage	0	0	0	0	0	0	0	0	0
No Clinically Visible Evidence of BCC	2(100%)	0	0	0	0	1(100%)	1(100%)	0	1(100%)
Other	0	0	0	0	0	0	0	0	0
					Cohort 1: Newly Determined Advanced BCC Patients (Non-BCCNS) Locally Advanced BCC (laBCC)				
		Vismo (N=115)			No Vismo (N=251)				
	All (N=433)	All (N=115)	Vismo Only (N=102)	Surgery Only (N=6)	Other Therapy (N=7)	All (N=251)	Surgery Only (N=198)	Other Therapy (N=53)	No Treatment (N=67)

Indicate How Response/ Progression was Assessed:									
n	112	57	51	3	3	41	26	15	14
Change in Size of Existing Lesions	90(80.4%)	53(93.0%)	47(92.2%)	3(100%)	3(100%)	27(65.9%)	15(57.7%)	12(80.0%)	10(71.4%)
Change in Number of Existing Lesions	21(18.8%)	13(22.8%)	10(19.6%)	1(33.3%)	2(66.7%)	5(12.2%)	4(15.4%)	1(6.7%)	3(21.4%)
Development of New BCCs	16(14.3%)	4(7.0%)	3(5.9%)	0	1(33.3%)	10(24.4%)	8(30.8%)	2(13.3%)	2(14.3%)
Progression from Locally Advanced Disease to Metastatic Disease	2(1.8%)	1(1.8%)	0	0	1(33.3%)	0	0	0	1(7.1%)
Development of New Metastases	0	0	0	0	0	0	0	0	0
Other	11(9.8%)	3(5.3%)	3(5.9%)	0	0	6(14.6%)	5(19.2%)	1(6.7%)	2(14.3%)
If progression to metastatic disease or new metastases, indicate site(s)[2]									
Bone	0	0	0	0	0	0	0	0	0
Lymph nodes, Local	0	0	0	0	0	0	0	0	0
Lymph nodes, Distant	0	0	0	0	0	0	0	0	0
Lung	0	0	0	0	0	0	0	0	0
Liver	0	0	0	0	0	0	0	0	0
CNS/Brain	0	0	0	0	0	0	0	0	0
Other	1(50.0%)	0	0	0	0	0	0	0	1(100%)

What Methods were Used to Assess Disease Response/ Progression									
n	265	89	79	4	6	147	109	38	29
Diagnostic Image (MRI, CT)	7(2.6%)	6(6.7%)	4(5.1%)	1(25.0%)	1(16.7%)	0	0	0	1(3.4%)
Histopathologic Evaluation	28(10.6%)	5(5.6%)	5(6.3%)	0	0	21(14.3%)	15(13.8%)	6(15.8%)	2(6.9%)
Photography	24(9.1%)	20(22.5%)	17(21.5%)	1(25.0%)	2(33.3%)	3(2.0%)	2(1.8%)	1(2.6%)	1(3.4%)
Clinical Evaluation	251(94.7%)	84(94.4%)	74(93.7%)	4(100%)	6(100%)	140(95.2%)	103(94.5%)	37(97.4%)	27(93.1%)
Laboratory Evaluation	4(1.5%)	2(2.2%)	2(2.5%)	0	0	2(1.4%)	2(1.8%)	0	0
Other	3(1.1%)	0	0	0	0	2(1.4%)	2(1.8%)	0	1(3.4%)
					Cohort 1: Newly Determined Advanced BCC Patients (Non-BCCNS) Metastatic BCC (mBCC)				
			Vismo (N=2)			No Vismo (N=1)			
	All (N=4)	All (N=2)	Vismo Only (N=2)	Surgery Only (N=0)	Other Therapy (N=0)	All (N=1)	Surgery Only (N=1)	Other Therapy (N=0)	No Treatment (N=1)
Indicate How Response/ Progression was Assessed:									
n	1	1	1	0	0	0	0	0	0
Change in Size of Existing Lesions	1(100%)	1(100%)	1(100%)	0	0	0	0	0	0
Change in Number of Existing Lesions	1(100%)	1(100%)	1(100%)	0	0	0	0	0	0
Development of New BCCs	0	0	0	0	0	0	0	0	0
Progression from Locally Advanced Disease to Metastatic Disease	0	0	0	0	0	0	0	0	0

Development of New Metastases	0	0	0	0	0	0	0	0	0
Other	0	0	0	0	0	0	0	0	0
If progression to metastatic disease or new metastases, indicate site(s)[2]									
Bone	0	0	0	0	0	0	0	0	0
Lymph nodes, Local	0	0	0	0	0	0	0	0	0
Lymph nodes, Distant	0	0	0	0	0	0	0	0	0
Lung	0	0	0	0	0	0	0	0	0
Liver	0	0	0	0	0	0	0	0	0
CNS/Brain	0	0	0	0	0	0	0	0	0
Other	0	0	0	0	0	0	0	0	0
What Methods were Used to Assess Disease Response/ Progression									
n	4	2	2	0	0	1	1	0	1
Diagnostic Image (MRI, CT)	2(50.0%)	2(100%)	2(100%)	0	0	0	0	0	0
Histopathologic Evaluation	0	0	0	0	0	0	0	0	0
Photography	1(25.0%)	0	0	0	0	0	0	0	1(100%)
Clinical Evaluation	3(75.0%)	1(50.0%)	1(50.0%)	0	0	1(100%)	1(100%)	0	1(100%)
Laboratory Evaluation	0	0	0	0	0	0	0	0	0
Other	0	0	0	0	0	0	0	0	0
					Cohort 1: Newly Determined Advanced BCC Patients (Non-BCCNS) Locally Advanced BCC (laBCC)				
		Vismo (N=115)			No Vismo (N=251)				
	All (N=433)	All (N=115)	Vismo Only (N=102)	Surgery Only (N=6)	Other Therapy (N=7)	All (N=251)	Surgery Only (N=198)	Other Therapy (N=53)	No Treatment (N=67)

Routine Follow-up 3									
Response Assessment									
n	218	81	71	4	6	110	77	33	27
Complete Response (CR)	126(57.8%)	25(30.9%)	19(26.8%)	2(50.0%)	4(66.7%)	89(80.9%)	66(85.7%)	23(69.7%)	12(44.4%)
Partial Response (PR)	47(21.6%)	34(42.0%)	32(45.1%)	1(25.0%)	1(16.7%)	9(8.2%)	2(2.6%)	7(21.2%)	4(14.8%)
Stable Disease (SD)	27(12.4%)	12(14.8%)	10(14.1%)	1(25.0%)	1(16.7%)	6(5.5%)	5(6.5%)	1(3.0%)	9(33.3%)
Disease Progression (PD)	10(4.6%)	6(7.4%)	6(8.5%)	0	0	3(2.7%)	1(1.3%)	2(6.1%)	1(3.7%)
Recurrence	4(1.8%)	2(2.5%)	2(2.8%)	0	0	2(1.8%)	2(2.6%)	0	0
Clinical Deterioration	0	0	0	0	0	0	0	0	0
Unable to Evaluate	4(1.8%)	2(2.5%)	2(2.8%)	0	0	1(0.9%)	1(1.3%)	0	1(3.7%)
If Complete Response, as Determined by[1]									
Negative Histopathology	21(16.7%)	4(16.0%)	3(15.8%)	0	1(25.0%)	16(18.0%)	11(16.7%)	5(21.7%)	1(8.3%)
Complete Tumor Shrinkage	24(19.0%)	14(56.0%)	10(52.6%)	1(50.0%)	3(75.0%)	9(10.1%)	4(6.1%)	5(21.7%)	1(8.3%)
No Clinically Visible Evidence of BCC	111(88.1%)	21(84.0%)	16(84.2%)	2(100%)	3(75.0%)	80(89.9%)	60(90.9%)	20(87.0%)	10(83.3%)
Other	2(1.6%)	0	0	0	0	2(2.2%)	2(3.0%)	0	0
					Cohort 1: Newly Determined Advanced BCC Patients (Non-BCCNS) Metastatic BCC (mBCC)				
			Vismo (N=2)			No Vismo (N=1)			
	All (N=4)	All (N=2)	Vismo Only (N=2)	Surgery Only (N=0)	Other Therapy (N=0)	All (N=1)	Surgery Only (N=1)	Other Therapy (N=0)	No Treatment (N=1)
Routine Follow-up 3									
Response Assessment									
n	3	2	2	0	0	0	0	0	1

Complete Response (CR)	1(33.3%)	0	0	0	0	0	0	0	1(100%)
Partial Response (PR)	0	0	0	0	0	0	0	0	0
Stable Disease (SD)	1(33.3%)	1(50.0%)	1(50.0%)	0	0	0	0	0	0
Disease Progression (PD)	1(33.3%)	1(50.0%)	1(50.0%)	0	0	0	0	0	0
Recurrence	0	0	0	0	0	0	0	0	0
Clinical Deterioration	0	0	0	0	0	0	0	0	0
Unable to Evaluate	0	0	0	0	0	0	0	0	0
If Complete Response, as Determined by[1]									
Negative Histopathology	0	0	0	0	0	0	0	0	0
Complete Tumor Shrinkage	0	0	0	0	0	0	0	0	0
No Clinically Visible Evidence of BCC	1(100%)	0	0	0	0	0	0	0	1(100%)
Other	0	0	0	0	0	0	0	0	0
					Cohort 1: Newly Determined Advanced BCC Patients (Non-BCCNS) Locally Advanced BCC (laBCC)				
			Vismo (N=115)			No Vismo (N=251)			
	All (N=433)	All (N=115)	Vismo Only (N=102)	Surgery Only (N=6)	Other Therapy (N=7)	All (N=251)	Surgery Only (N=198)	Other Therapy (N=53)	No Treatment (N=67)
Indicate How Response/ Progression was Assessed:									
n	99	57	52	3	2	29	15	14	13
Change in Size of Existing Lesions	77(77.8%)	47(82.5%)	42(80.8%)	3(100%)	2(100%)	22(75.9%)	9(60.0%)	13(92.9%)	8(61.5%)

Change in Number of Existing Lesions	16(16.2%)	9(15.8%)	8(15.4%)	1(33.3%)	0	5(17.2%)	4(26.7%)	1(7.1%)	2(15.4%)
Development of New BCCs	15(15.2%)	6(10.5%)	6(11.5%)	0	0	6(20.7%)	5(33.3%)	1(7.1%)	3(23.1%)
Progression from Locally Advanced Disease to Metastatic Disease	1(1.0%)	1(1.8%)	1(1.9%)	0	0	0	0	0	0
Development of New Metastases	0	0	0	0	0	0	0	0	0
Other	13(13.1%)	4(7.0%)	4(7.7%)	0	0	3(10.3%)	3(20.0%)	0	6(46.2%)
If progression to metastatic disease or new metastases, indicate site(s)[2]									
Bone	0	0	0	0	0	0	0	0	0
Lymph nodes, Local	0	0	0	0	0	0	0	0	0
Lymph nodes, Distant	0	0	0	0	0	0	0	0	0
Lung	0	0	0	0	0	0	0	0	0
Liver	0	0	0	0	0	0	0	0	0
CNS/Brain	0	0	0	0	0	0	0	0	0
Other	0	0	0	0	0	0	0	0	0
What Methods were Used to Assess Disease Response/ Progression									
n	218	81	71	4	6	110	77	33	27
Diagnostic Image (MRI, CT)	9(4.1%)	5(6.2%)	5(7.0%)	0	0	2(1.8%)	2(2.6%)	0	2(7.4%)
Histopathologic Evaluation	27(12.4%)	10(12.3%)	9(12.7%)	0	1(16.7%)	14(12.7%)	12(15.6%)	2(6.1%)	3(11.1%)

Photography	22(10.1%)	19(23.5%)	17(23.9%)	1(25.0%)	1(16.7%)	2(1.8%)	2(2.6%)	0	1(3.7%)
Clinical Evaluation	205(94.0%)	75(92.6%)	65(91.5%)	4(100%)	6(100%)	105(95.5%)	74(96.1%)	31(93.9%)	25(92.6%)
Laboratory Evaluation	3(1.4%)	1(1.2%)	1(1.4%)	0	0	2(1.8%)	0	2(6.1%)	0
Other	4(1.8%)	1(1.2%)	1(1.4%)	0	0	2(1.8%)	2(2.6%)	0	1(3.7%)

					Cohort 1: Newly Determined Advanced BCC Patients (Non-BCCNS) Metastatic BCC (mBCC)				
			Vismo (N=2)		No Vismo (N=1)				
	All (N=4)	All (N=2)	Vismo Only (N=2)	Surgery Only (N=0)	Other Therapy (N=0)	All (N=1)	Surgery Only (N=1)	Other Therapy (N=0)	No Treatment (N=1)
Indicate How Response/ Progression was Assessed:									
n	1	1	1	0	0	0	0	0	0
Change in Size of Existing Lesions	1(100%)	1(100%)	1(100%)	0	0	0	0	0	0
Change in Number of Existing Lesions	0	0	0	0	0	0	0	0	0
Development of New BCCs	0	0	0	0	0	0	0	0	0
Progression from Locally Advanced Disease to Metastatic Disease	0	0	0	0	0	0	0	0	0
Development of New Metastases	0	0	0	0	0	0	0	0	0
Other	0	0	0	0	0	0	0	0	0
If progression to metastatic disease or new metastases, indicate site(s)[2]									
Bone	0	0	0	0	0	0	0	0	0

Lymph nodes, Local	0	0	0	0	0	0	0	0	0
Lymph nodes, Distant	0	0	0	0	0	0	0	0	0
Lung	0	0	0	0	0	0	0	0	0
Liver	0	0	0	0	0	0	0	0	0
CNS/Brain	0	0	0	0	0	0	0	0	0
Other	0	0	0	0	0	0	0	0	0
What Methods were Used to Assess Disease Response/ Progression									
n	3	2	2	0	0	0	0	0	1
Diagnostic Image (MRI, CT)	0	0	0	0	0	0	0	0	0
Histopathologic Evaluation	1(33.3%)	1(50.0%)	1(50.0%)	0	0	0	0	0	0
Photography	2(66.7%)	1(50.0%)	1(50.0%)	0	0	0	0	0	1(100%)
Clinical Evaluation	3(100%)	2(100%)	2(100%)	0	0	0	0	0	1(100%)
Laboratory Evaluation	0	0	0	0	0	0	0	0	0
Other	0	0	0	0	0	0	0	0	0

					Cohort 1: Newly Determined Advanced BCC Patients (Non-BCCNS) Locally Advanced BCC (laBCC)				
			Vismo (N=115)		No Vismo (N=251)				
	All (N=433)	All (N=115)	Vismo Only (N=102)	Surgery Only (N=6)	Other Therapy (N=7)	All (N=251)	Surgery Only (N=198)	Other Therapy (N=53)	No Treatment (N=67)
Routine Follow-up 4									
Response Assessment									
n	191	79	69	4	6	90	63	27	22
Complete Response (CR)	120(62.8%)	34(43.0%)	27(39.1%)	4(100%)	3(50.0%)	76(84.4%)	54(85.7%)	22(81.5%)	10(45.5%)
Partial Response (PR)	27(14.1%)	20(25.3%)	19(27.5%)	0	1(16.7%)	6(6.7%)	2(3.2%)	4(14.8%)	1(4.5%)
Stable Disease (SD)	35(18.3%)	19(24.1%)	18(26.1%)	0	1(16.7%)	6(6.7%)	6(9.5%)	0	10(45.5%)

Disease Progression (PD)	4(2.1%)	3(3.8%)	2(2.9%)	0	1(16.7%)	1(1.1%)	0	1(3.7%)	0
Recurrence	4(2.1%)	3(3.8%)	3(4.3%)	0	0	1(1.1%)	1(1.6%)	0	0
Clinical Deterioration	0	0	0	0	0	0	0	0	0
Unable to Evaluate	1(0.5%)	0	0	0	0	0	0	0	1(4.5%)
If Complete Response, as Determined by[1]									
Negative Histopathology	18(15.0%)	2(5.9%)	2(7.4%)	0	0	14(18.4%)	9(16.7%)	5(22.7%)	2(20.0%)
Complete Tumor Shrinkage	22(18.3%)	11(32.4%)	7(25.9%)	2(50.0%)	2(66.7%)	9(11.8%)	2(3.7%)	7(31.8%)	2(20.0%)
No Clinically Visible Evidence of BCC	100(83.3%)	30(88.2%)	24(88.9%)	3(75.0%)	3(100%)	63(82.9%)	46(85.2%)	17(77.3%)	7(70.0%)
Other	2(1.7%)	1(2.9%)	1(3.7%)	0	0	1(1.3%)	0	1(4.5%)	0

					Cohort 1: Newly Determined Advanced BCC Patients (Non-BCCNS) Metastatic BCC (mBCC)				
		Vismo (N=2)			No Vismo (N=1)				
	All (N=4)	All (N=2)	Vismo Only (N=2)	Surgery Only (N=0)	Other Therapy (N=0)	All (N=1)	Surgery Only (N=1)	Other Therapy (N=0)	No Treatment (N=1)
Routine Follow-up 4									
Response Assessment									
n	2	1	1	0	0	0	0	0	1
Complete Response (CR)	0	0	0	0	0	0	0	0	0
Partial Response (PR)	1(50.0%)	1(100%)	1(100%)	0	0	0	0	0	0
Stable Disease (SD)	0	0	0	0	0	0	0	0	0
Disease Progression (PD)	0	0	0	0	0	0	0	0	0
Recurrence	0	0	0	0	0	0	0	0	0
Clinical Deterioration	0	0	0	0	0	0	0	0	0

Unable to Evaluate	1(50.0%)	0	0	0	0	0	0	0	1(100%)
If Complete Response, as Determined by[1]									
Negative Histopathology	0	0	0	0	0	0	0	0	0
Complete Tumor Shrinkage	0	0	0	0	0	0	0	0	0
No Clinically Visible Evidence of BCC	0	0	0	0	0	0	0	0	0
Other	0	0	0	0	0	0	0	0	0

					Cohort 1: Newly Determined Advanced BCC Patients (Non-BCCNS) Locally Advanced BCC (laBCC)				
			Vismo (N=115)			No Vismo (N=251)			
	All (N=433)	All (N=115)	Vismo Only (N=102)	Surgery Only (N=6)	Other Therapy (N=7)	All (N=251)	Surgery Only (N=198)	Other Therapy (N=53)	No Treatment (N=67)
Indicate How Response/ Progression was Assessed:									
n	71	54	50	1	3	14	11	3	3
Change in Size of Existing Lesions	56(78.9%)	47(87.0%)	43(86.0%)	1(100%)	3(100%)	7(50.0%)	4(36.4%)	3(100%)	2(66.7%)
Change in Number of Existing Lesions	15(21.1%)	11(20.4%)	10(20.0%)	0	1(33.3%)	2(14.3%)	2(18.2%)	0	2(66.7%)
Development of New BCCs	17(23.9%)	8(14.8%)	7(14.0%)	0	1(33.3%)	7(50.0%)	6(54.5%)	1(33.3%)	2(66.7%)
Progression from Locally Advanced Disease to Metastatic Disease	1(1.4%)	0	0	0	0	1(7.1%)	0	1(33.3%)	0
Development of New Metastases	0	0	0	0	0	0	0	0	0

Other	6(8.5%)	4(7.4%)	4(8.0%)	0	0	2(14.3%)	2(18.2%)	0	0
If progression to metastatic disease or new metastases, indicate site(s)[2]									
Bone	0	0	0	0	0	0	0	0	0
Lymph nodes, Local	0	0	0	0	0	0	0	0	0
Lymph nodes, Distant	0	0	0	0	0	0	0	0	0
Lung	0	0	0	0	0	0	0	0	0
Liver	0	0	0	0	0	0	0	0	0
CNS/Brain	0	0	0	0	0	0	0	0	0
Other	1(100%)	0	0	0	0	1(100%)	0	1(100%)	0
What Methods were Used to Assess Disease Response/ Progression									
n	191	79	69	4	6	90	63	27	22
Diagnostic Image (MRI, CT)	6(3.1%)	5(6.3%)	5(7.2%)	0	0	0	0	0	1(4.5%)
Histopathologic Evaluation	24(12.6%)	7(8.9%)	7(10.1%)	0	0	13(14.4%)	10(15.9%)	3(11.1%)	4(18.2%)
Photography	22(11.5%)	17(21.5%)	14(20.3%)	2(50.0%)	1(16.7%)	1(1.1%)	1(1.6%)	0	4(18.2%)
Clinical Evaluation	174(91.1%)	72(91.1%)	62(89.9%)	4(100%)	6(100%)	83(92.2%)	59(93.7%)	24(88.9%)	19(86.4%)
Laboratory Evaluation	5(2.6%)	0	0	0	0	4(4.4%)	2(3.2%)	2(7.4%)	1(4.5%)
Other	4(2.1%)	1(1.3%)	1(1.4%)	0	0	2(2.2%)	2(3.2%)	0	1(4.5%)

					Cohort 1: Newly Determined Advanced BCC Patients (Non-BCCNS) Metastatic BCC (mBCC)				
		Vismo (N=2)			No Vismo (N=1)				
	All (N=4)	All (N=2)	Vismo Only (N=2)	Surgery Only (N=0)	Other Therapy (N=0)	All (N=1)	Surgery Only (N=1)	Other Therapy (N=0)	No Treatment (N=1)

Indicate How Response/ Progression was Assessed:									
n	1	1	1	0	0	0	0	0	0
Change in Size of Existing Lesions	1(100%)	1(100%)	1(100%)	0	0	0	0	0	0
Change in Number of Existing Lesions	0	0	0	0	0	0	0	0	0
Development of New BCCs	0	0	0	0	0	0	0	0	0
Progression from Locally Advanced Disease to Metastatic Disease	0	0	0	0	0	0	0	0	0
Development of New Metastases	0	0	0	0	0	0	0	0	0
Other	0	0	0	0	0	0	0	0	0
If progression to metastatic disease or new metastases, indicate site(s)[2]									
Bone	0	0	0	0	0	0	0	0	0
Lymph nodes, Local	0	0	0	0	0	0	0	0	0
Lymph nodes, Distant	0	0	0	0	0	0	0	0	0
Lung	0	0	0	0	0	0	0	0	0
Liver	0	0	0	0	0	0	0	0	0
CNS/Brain	0	0	0	0	0	0	0	0	0
Other	0	0	0	0	0	0	0	0	0

What Methods were Used to Assess Disease Response/ Progression									
n	2	1	1	0	0	0	0	0	1
Diagnostic Image (MRI, CT)	1(50.0%)	1(100%)	1(100%)	0	0	0	0	0	0
Histopathologic Evaluation	0	0	0	0	0	0	0	0	0
Photography	1(50.0%)	0	0	0	0	0	0	0	1(100%)
Clinical Evaluation	2(100%)	1(100%)	1(100%)	0	0	0	0	0	1(100%)
Laboratory Evaluation	0	0	0	0	0	0	0	0	0
Other	0	0	0	0	0	0	0	0	0

					Cohort 1: Newly Determined Advanced BCC Patients (Non-BCCNS) Locally Advanced BCC (laBCC)				
		Vismo (N=115)			No Vismo (N=251)				
	All (N=433)	All (N=115)	Vismo Only (N=102)	Surgery Only (N=6)	Other Therapy (N=7)	All (N=251)	Surgery Only (N=198)	Other Therapy (N=53)	No Treatment (N=67)
Routine Follow-up 5									
Response Assessment									
n	154	68	58	4	6	69	46	23	17
Complete Response (CR)	97(63.0%)	28(41.2%)	21(36.2%)	4(100%)	3(50.0%)	61(88.4%)	41(89.1%)	20(87.0%)	8(47.1%)
Partial Response (PR)	25(16.2%)	21(30.9%)	19(32.8%)	0	2(33.3%)	3(4.3%)	1(2.2%)	2(8.7%)	1(5.9%)
Stable Disease (SD)	22(14.3%)	12(17.6%)	12(20.7%)	0	0	3(4.3%)	2(4.3%)	1(4.3%)	7(41.2%)
Disease Progression (PD)	6(3.9%)	3(4.4%)	2(3.4%)	0	1(16.7%)	2(2.9%)	2(4.3%)	0	1(5.9%)
Recurrence	3(1.9%)	3(4.4%)	3(5.2%)	0	0	0	0	0	0
Clinical Deterioration	1(0.6%)	1(1.5%)	1(1.7%)	0	0	0	0	0	0
Unable to Evaluate	0	0	0	0	0	0	0	0	0

If Complete Response, as Determined by[1]									
Negative Histopathology	13(13.4%)	3(10.7%)	3(14.3%)	0	0	9(14.8%)	4(9.8%)	5(25.0%)	1(12.5%)
Complete Tumor Shrinkage	20(20.6%)	12(42.9%)	8(38.1%)	2(50.0%)	2(66.7%)	7(11.5%)	0	7(35.0%)	1(12.5%)
No Clinically Visible Evidence of BCC	86(88.7%)	25(89.3%)	19(90.5%)	3(75.0%)	3(100%)	54(88.5%)	38(92.7%)	16(80.0%)	7(87.5%)
Other	1(1.0%)	1(3.6%)	1(4.8%)	0	0	0	0	0	0

					Cohort 1: Newly Determined Advanced BCC Patients (Non-BCCNS) Metastatic BCC (mBCC)				
		Vismo (N=2)			No Vismo (N=1)				
	All (N=4)	All (N=2)	Vismo Only (N=2)	Surgery Only (N=0)	Other Therapy (N=0)	All (N=1)	Surgery Only (N=1)	Other Therapy (N=0)	No Treatment (N=1)
Routine Follow-up 5									
Response Assessment									
n	1	0	0	0	0	0	0	0	1
Complete Response (CR)	0	0	0	0	0	0	0	0	0
Partial Response (PR)	0	0	0	0	0	0	0	0	0
Stable Disease (SD)	1(100%)	0	0	0	0	0	0	0	1(100%)
Disease Progression (PD)	0	0	0	0	0	0	0	0	0
Recurrence	0	0	0	0	0	0	0	0	0
Clinical Deterioration	0	0	0	0	0	0	0	0	0
Unable to Evaluate	0	0	0	0	0	0	0	0	0
If Complete Response, as Determined by[1]									

Negative Histopathology	0	0	0	0	0	0	0	0	0
Complete Tumor Shrinkage	0	0	0	0	0	0	0	0	0
No Clinically Visible Evidence of BCC	0	0	0	0	0	0	0	0	0
Other	0	0	0	0	0	0	0	0	0
					Cohort 1: Newly Determined Advanced BCC Patients (Non-BCCNS) Locally Advanced BCC (laBCC)				
			Vismo (N=115)			No Vismo (N=251)			
	All (N=433)	All (N=115)	Vismo Only (N=102)	Surgery Only (N=6)	Other Therapy (N=7)	All (N=251)	Surgery Only (N=198)	Other Therapy (N=53)	No Treatment (N=67)
Indicate How Response/ Progression was Assessed:									
n	60	44	40	1	3	8	5	3	8
Change in Size of Existing Lesions	46(76.7%)	36(81.8%)	32(80.0%)	1(100%)	3(100%)	5(62.5%)	2(40.0%)	3(100%)	5(62.5%)
Change in Number of Existing Lesions	16(26.7%)	12(27.3%)	12(30.0%)	0	0	2(25.0%)	2(40.0%)	0	2(25.0%)
Development of New BCCs	13(21.7%)	10(22.7%)	10(25.0%)	0	0	2(25.0%)	2(40.0%)	0	1(12.5%)
Progression from Locally Advanced Disease to Metastatic Disease	2(3.3%)	2(4.5%)	2(5.0%)	0	0	0	0	0	0
Development of New Metastases	0	0	0	0	0	0	0	0	0
Other	4(6.7%)	2(4.5%)	2(5.0%)	0	0	0	0	0	2(25.0%)

If progression to metastatic disease or new metastases, indicate site(s)[2]									
Bone	0	0	0	0	0	0	0	0	0
Lymph nodes, Local	0	0	0	0	0	0	0	0	0
Lymph nodes, Distant	0	0	0	0	0	0	0	0	0
Lung	0	0	0	0	0	0	0	0	0
Liver	0	0	0	0	0	0	0	0	0
CNS/Brain	0	0	0	0	0	0	0	0	0
Other	0	0	0	0	0	0	0	0	0
What Methods were Used to Assess Disease Response/ Progression									
n	154	68	58	4	6	69	46	23	17
Diagnostic Image (MRI, CT)	10(6.5%)	9(13.2%)	9(15.5%)	0	0	0	0	0	1(5.9%)
Histopathologic Evaluation	24(15.6%)	8(11.8%)	8(13.8%)	0	0	11(15.9%)	6(13.0%)	5(21.7%)	5(29.4%)
Photography	11(7.1%)	11(16.2%)	9(15.5%)	1(25.0%)	1(16.7%)	0	0	0	0
Clinical Evaluation	141(91.6%)	63(92.6%)	53(91.4%)	4(100%)	6(100%)	63(91.3%)	44(95.7%)	19(82.6%)	15(88.2%)
Laboratory Evaluation	3(1.9%)	2(2.9%)	2(3.4%)	0	0	1(1.4%)	1(2.2%)	0	0
Other	1(0.6%)	1(1.5%)	1(1.7%)	0	0	0	0	0	0
					Cohort 1: Newly Determined Advanced BCC Patients (Non-BCCNS) Metastatic BCC (mBCC)				
			Vismo (N=2)			No Vismo (N=1)			
	All (N=4)	All (N=2)	Vismo Only (N=2)	Surgery Only (N=0)	Other Therapy (N=0)	All (N=1)	Surgery Only (N=1)	Other Therapy (N=0)	No Treatment (N=1)

Indicate How Response/ Progression was Assessed:									
n	0	0	0	0	0	0	0	0	0
Change in Size of Existing Lesions	0	0	0	0	0	0	0	0	0
Change in Number of Existing Lesions	0	0	0	0	0	0	0	0	0
Development of New BCCs	0	0	0	0	0	0	0	0	0
Progression from Locally Advanced Disease to Metastatic Disease	0	0	0	0	0	0	0	0	0
Development of New Metastases	0	0	0	0	0	0	0	0	0
Other	0	0	0	0	0	0	0	0	0
If progression to metastatic disease or new metastases, indicate site(s)[2]									
Bone	0	0	0	0	0	0	0	0	0
Lymph nodes, Local	0	0	0	0	0	0	0	0	0
Lymph nodes, Distant	0	0	0	0	0	0	0	0	0
Lung	0	0	0	0	0	0	0	0	0
Liver	0	0	0	0	0	0	0	0	0
CNS/Brain	0	0	0	0	0	0	0	0	0
Other	0	0	0	0	0	0	0	0	0

What Methods were Used to Assess Disease Response/ Progression									
n	1	0	0	0	0	0	0	0	1
Diagnostic Image (MRI, CT)	0	0	0	0	0	0	0	0	0
Histopathologic Evaluation	0	0	0	0	0	0	0	0	0
Photography	1(100%)	0	0	0	0	0	0	0	1(100%)
Clinical Evaluation	1(100%)	0	0	0	0	0	0	0	1(100%)
Laboratory Evaluation	0	0	0	0	0	0	0	0	0
Other	0	0	0	0	0	0	0	0	0
					Cohort 1: Newly Determined Advanced BCC Patients (Non-BCCNS) Locally Advanced BCC (laBCC)				
		Vismo (N=115)			No Vismo (N=251)				
	All (N=433)	All (N=115)	Vismo Only (N=102)	Surgery Only (N=6)	Other Therapy (N=7)	All (N=251)	Surgery Only (N=198)	Other Therapy (N=53)	No Treatment (N=67)
Routine Follow-up 6									
Response Assessment									
n	127	58	50	3	5	56	40	16	13
Complete Response (CR)	86(67.7%)	26(44.8%)	21(42.0%)	2(66.7%)	3(60.0%)	52(92.9%)	37(92.5%)	15(93.8%)	8(61.5%)
Partial Response (PR)	15(11.8%)	15(25.9%)	13(26.0%)	0	2(40.0%)	0	0	0	0
Stable Disease (SD)	16(12.6%)	12(20.7%)	12(24.0%)	0	0	0	0	0	4(30.8%)
Disease Progression (PD)	2(1.6%)	0	0	0	0	2(3.6%)	2(5.0%)	0	0
Recurrence	4(3.1%)	4(6.9%)	3(6.0%)	1(33.3%)	0	0	0	0	0
Clinical Deterioration	2(1.6%)	1(1.7%)	1(2.0%)	0	0	1(1.8%)	0	1(6.3%)	0
Unable to Evaluate	2(1.6%)	0	0	0	0	1(1.8%)	1(2.5%)	0	1(7.7%)

If Complete Response, as Determined by[1]									
Negative Histopathology	13(15.1%)	3(11.5%)	3(14.3%)	0	0	10(19.2%)	7(18.9%)	3(20.0%)	0
Complete Tumor Shrinkage	20(23.3%)	10(38.5%)	9(42.9%)	0	1(33.3%)	8(15.4%)	1(2.7%)	7(46.7%)	2(25.0%)
No Clinically Visible Evidence of BCC	71(82.6%)	24(92.3%)	19(90.5%)	2(100%)	3(100%)	42(80.8%)	31(83.8%)	11(73.3%)	5(62.5%)
Other	3(3.5%)	0	0	0	0	2(3.8%)	2(5.4%)	0	1(12.5%)

					Cohort 1: Newly Determined Advanced BCC Patients (Non-BCCNS) Metastatic BCC (mBCC)				
		Vismo (N=2)			No Vismo (N=1)				
	All (N=4)	All (N=2)	Vismo Only (N=2)	Surgery Only (N=0)	Other Therapy (N=0)	All (N=1)	Surgery Only (N=1)	Other Therapy (N=0)	No Treatment (N=1)
Routine Follow-up 6									
Response Assessment									
n	1	0	0	0	0	0	0	0	1
Complete Response (CR)	1(100%)	0	0	0	0	0	0	0	1(100%)
Partial Response (PR)	0	0	0	0	0	0	0	0	0
Stable Disease (SD)	0	0	0	0	0	0	0	0	0
Disease Progression (PD)	0	0	0	0	0	0	0	0	0
Recurrence	0	0	0	0	0	0	0	0	0
Clinical Deterioration	0	0	0	0	0	0	0	0	0
Unable to Evaluate	0	0	0	0	0	0	0	0	0
If Complete Response, as Determined by[1]									

Negative Histopathology	1(100%)	0	0	0	0	0	0	0	1(100%)
Complete Tumor Shrinkage	0	0	0	0	0	0	0	0	0
No Clinically Visible Evidence of BCC	0	0	0	0	0	0	0	0	0
Other	0	0	0	0	0	0	0	0	0

					Cohort 1: Newly Determined Advanced BCC Patients (Non-BCCNS) Locally Advanced BCC (laBCC)				
		Vismo (N=115)			No Vismo (N=251)				
	All (N=433)	All (N=115)	Vismo Only (N=102)	Surgery Only (N=6)	Other Therapy (N=7)	All (N=251)	Surgery Only (N=198)	Other Therapy (N=53)	No Treatment (N=67)
Indicate How Response/ Progression was Assessed:									
n	56	40	37	1	2	12	7	5	4
Change in Size of Existing Lesions	42(75.0%)	33(82.5%)	31(83.8%)	0	2(100%)	6(50.0%)	2(28.6%)	4(80.0%)	3(75.0%)
Change in Number of Existing Lesions	11(19.6%)	8(20.0%)	7(18.9%)	0	1(50.0%)	0	0	0	3(75.0%)
Development of New BCCs	12(21.4%)	6(15.0%)	5(13.5%)	1(100%)	0	5(41.7%)	4(57.1%)	1(20.0%)	1(25.0%)
Progression from Locally Advanced Disease to Metastatic Disease	1(1.8%)	1(2.5%)	1(2.7%)	0	0	0	0	0	0
Development of New Metastases	0	0	0	0	0	0	0	0	0
Other	5(8.9%)	4(10.0%)	4(10.8%)	0	0	1(8.3%)	1(14.3%)	0	0

If progression to metastatic disease or new metastases, indicate site(s)[2]									
Bone	0	0	0	0	0	0	0	0	0
Lymph nodes, Local	0	0	0	0	0	0	0	0	0
Lymph nodes, Distant	0	0	0	0	0	0	0	0	0
Lung	0	0	0	0	0	0	0	0	0
Liver	0	0	0	0	0	0	0	0	0
CNS/Brain	0	0	0	0	0	0	0	0	0
Other	0	0	0	0	0	0	0	0	0
What Methods were Used to Assess Disease Response/ Progression									
n	127	58	50	3	5	56	40	16	13
Diagnostic Image (MRI, CT)	1(0.8%)	1(1.7%)	1(2.0%)	0	0	0	0	0	0
Histopathologic Evaluation	14(11.0%)	7(12.1%)	6(12.0%)	1(33.3%)	0	7(12.5%)	4(10.0%)	3(18.8%)	0
Photography	9(7.1%)	9(15.5%)	9(18.0%)	0	0	0	0	0	0
Clinical Evaluation	120(94.5%)	57(98.3%)	49(98.0%)	3(100%)	5(100%)	51(91.1%)	37(92.5%)	14(87.5%)	12(92.3%)
Laboratory Evaluation	4(3.1%)	2(3.4%)	2(4.0%)	0	0	1(1.8%)	1(2.5%)	0	1(7.7%)
Other	2(1.6%)	0	0	0	0	1(1.8%)	1(2.5%)	0	1(7.7%)
					Cohort 1: Newly Determined Advanced BCC Patients (Non-BCCNS) Metastatic BCC (mBCC)				
			Vismo (N=2)			No Vismo (N=1)			
	All (N=4)	All (N=2)	Vismo Only (N=2)	Surgery Only (N=0)	Other Therapy (N=0)	All (N=1)	Surgery Only (N=1)	Other Therapy (N=0)	No Treatment (N=1)

Indicate How Response/ Progression was Assessed:									
n	0	0	0	0	0	0	0	0	0
Change in Size of Existing Lesions	0	0	0	0	0	0	0	0	0
Change in Number of Existing Lesions	0	0	0	0	0	0	0	0	0
Development of New BCCs	0	0	0	0	0	0	0	0	0
Progression from Locally Advanced Disease to Metastatic Disease	0	0	0	0	0	0	0	0	0
Development of New Metastases	0	0	0	0	0	0	0	0	0
Other	0	0	0	0	0	0	0	0	0
If progression to metastatic disease or new metastases, indicate site(s)[2]									
Bone	0	0	0	0	0	0	0	0	0
Lymph nodes, Local	0	0	0	0	0	0	0	0	0
Lymph nodes, Distant	0	0	0	0	0	0	0	0	0
Lung	0	0	0	0	0	0	0	0	0
Liver	0	0	0	0	0	0	0	0	0
CNS/Brain	0	0	0	0	0	0	0	0	0
Other	0	0	0	0	0	0	0	0	0

What Methods were Used to Assess Disease Response/ Progression									
n	1	0	0	0	0	0	0	0	1
Diagnostic Image (MRI, CT)	0	0	0	0	0	0	0	0	0
Histopathologic Evaluation	1(100%)	0	0	0	0	0	0	0	1(100%)
Photography	1(100%)	0	0	0	0	0	0	0	1(100%)
Clinical Evaluation	1(100%)	0	0	0	0	0	0	0	1(100%)
Laboratory Evaluation	0	0	0	0	0	0	0	0	0
Other	1(100%)	0	0	0	0	0	0	0	1(100%)

					Cohort 1: Newly Determined Advanced BCC Patients (Non-BCCNS) Locally Advanced BCC (laBCC)				
			Vismo (N=115)			No Vismo (N=251)			
	All (N=433)	All (N=115)	Vismo Only (N=102)	Surgery Only (N=6)	Other Therapy (N=7)	All (N=251)	Surgery Only (N=198)	Other Therapy (N=53)	No Treatment (N=67)
Routine Follow-up 7									
Response Assessment									
n	104	50	43	2	5	46	34	12	8
Complete Response (CR)	71(68.3%)	26(52.0%)	21(48.8%)	1(50.0%)	4(80.0%)	41(89.1%)	30(88.2%)	11(91.7%)	4(50.0%)
Partial Response (PR)	5(4.8%)	5(10.0%)	5(11.6%)	0	0	0	0	0	0
Stable Disease (SD)	14(13.5%)	10(20.0%)	10(23.3%)	0	0	0	0	0	4(50.0%)
Disease Progression (PD)	6(5.8%)	4(8.0%)	3(7.0%)	0	1(20.0%)	2(4.3%)	2(5.9%)	0	0
Recurrence	6(5.8%)	5(10.0%)	4(9.3%)	1(50.0%)	0	1(2.2%)	0	1(8.3%)	0
Clinical Deterioration	0	0	0	0	0	0	0	0	0
Unable to Evaluate	2(1.9%)	0	0	0	0	2(4.3%)	2(5.9%)	0	0

If Complete Response, as Determined by[1]									
Negative Histopathology	9(12.7%)	4(15.4%)	3(14.3%)	0	1(25.0%)	5(12.2%)	4(13.3%)	1(9.1%)	0
Complete Tumor Shrinkage	16(22.5%)	9(34.6%)	8(38.1%)	0	1(25.0%)	6(14.6%)	1(3.3%)	5(45.5%)	1(25.0%)
No Clinically Visible Evidence of BCC	61(85.9%)	21(80.8%)	16(76.2%)	1(100%)	4(100%)	36(87.8%)	26(86.7%)	10(90.9%)	4(100%)
Other	2(2.8%)	1(3.8%)	1(4.8%)	0	0	1(2.4%)	1(3.3%)	0	0

					Cohort 1: Newly Determined Advanced BCC Patients (Non-BCCNS) Metastatic BCC (mBCC)				
			Vismo (N=2)			No Vismo (N=1)			
	All (N=4)	All (N=2)	Vismo Only (N=2)	Surgery Only (N=0)	Other Therapy (N=0)	All (N=1)	Surgery Only (N=1)	Other Therapy (N=0)	No Treatment (N=1)
Routine Follow-up 7									
Response Assessment									
n	1	0	0	0	0	0	0	0	1
Complete Response (CR)	0	0	0	0	0	0	0	0	0
Partial Response (PR)	0	0	0	0	0	0	0	0	0
Stable Disease (SD)	1(100%)	0	0	0	0	0	0	0	1(100%)
Disease Progression (PD)	0	0	0	0	0	0	0	0	0
Recurrence	0	0	0	0	0	0	0	0	0
Clinical Deterioration	0	0	0	0	0	0	0	0	0
Unable to Evaluate	0	0	0	0	0	0	0	0	0
If Complete Response, as Determined by[1]									

Negative Histopathology	0	0	0	0	0	0	0	0	0
Complete Tumor Shrinkage	0	0	0	0	0	0	0	0	0
No Clinically Visible Evidence of BCC	0	0	0	0	0	0	0	0	0
Other	0	0	0	0	0	0	0	0	0
					Cohort 1: Newly Determined Advanced BCC Patients (Non-BCCNS) Locally Advanced BCC (laBCC)				
			Vismo (N=115)			No Vismo (N=251)			
	All (N=433)	All (N=115)	Vismo Only (N=102)	Surgery Only (N=6)	Other Therapy (N=7)	All (N=251)	Surgery Only (N=198)	Other Therapy (N=53)	No Treatment (N=67)
Indicate How Response/ Progression was Assessed:									
n	36	29	25	1	3	3	1	2	4
Change in Size of Existing Lesions	33(91.7%)	27(93.1%)	23(92.0%)	1(100%)	3(100%)	3(100%)	1(100%)	2(100%)	3(75.0%)
Change in Number of Existing Lesions	11(30.6%)	7(24.1%)	7(28.0%)	0	0	2(66.7%)	1(100%)	1(50.0%)	2(50.0%)
Development of New BCCs	4(11.1%)	3(10.3%)	3(12.0%)	0	0	0	0	0	1(25.0%)
Progression from Locally Advanced Disease to Metastatic Disease	0	0	0	0	0	0	0	0	0
Development of New Metastases	0	0	0	0	0	0	0	0	0
Other	0	0	0	0	0	0	0	0	0

If progression to metastatic disease or new metastases, indicate site(s)[2]									
Bone	0	0	0	0	0	0	0	0	0
Lymph nodes, Local	0	0	0	0	0	0	0	0	0
Lymph nodes, Distant	0	0	0	0	0	0	0	0	0
Lung	0	0	0	0	0	0	0	0	0
Liver	0	0	0	0	0	0	0	0	0
CNS/Brain	0	0	0	0	0	0	0	0	0
Other	0	0	0	0	0	0	0	0	0
What Methods were Used to Assess Disease Response/ Progression									
n	104	50	43	2	5	46	34	12	8
Diagnostic Image (MRI, CT)	3(2.9%)	2(4.0%)	2(4.7%)	0	0	1(2.2%)	1(2.9%)	0	0
Histopathologic Evaluation	15(14.4%)	10(20.0%)	8(18.6%)	1(50.0%)	1(20.0%)	5(10.9%)	4(11.8%)	1(8.3%)	0
Photography	6(5.8%)	6(12.0%)	6(14.0%)	0	0	0	0	0	0
Clinical Evaluation	96(92.3%)	49(98.0%)	42(97.7%)	2(100%)	5(100%)	39(84.8%)	28(82.4%)	11(91.7%)	8(100%)
Laboratory Evaluation	2(1.9%)	2(4.0%)	2(4.7%)	0	0	0	0	0	0
Other	3(2.9%)	1(2.0%)	1(2.3%)	0	0	2(4.3%)	2(5.9%)	0	0
					Cohort 1: Newly Determined Advanced BCC Patients (Non-BCCNS) Metastatic BCC (mBCC)				
			Vismo (N=2)			No Vismo (N=1)			
	All (N=4)	All (N=2)	Vismo Only (N=2)	Surgery Only (N=0)	Other Therapy (N=0)	All (N=1)	Surgery Only (N=1)	Other Therapy (N=0)	No Treatment (N=1)

Indicate How Response/ Progression was Assessed:									
n	1	0	0	0	0	0	0	0	1
Change in Size of Existing Lesions	0	0	0	0	0	0	0	0	0
Change in Number of Existing Lesions	0	0	0	0	0	0	0	0	0
Development of New BCCs	0	0	0	0	0	0	0	0	0
Progression from Locally Advanced Disease to Metastatic Disease	0	0	0	0	0	0	0	0	0
Development of New Metastases	0	0	0	0	0	0	0	0	0
Other	1(100%)	0	0	0	0	0	0	0	1(100%)
If progression to metastatic disease or new metastases, indicate site(s)[2]									
Bone	0	0	0	0	0	0	0	0	0
Lymph nodes, Local	0	0	0	0	0	0	0	0	0
Lymph nodes, Distant	0	0	0	0	0	0	0	0	0
Lung	0	0	0	0	0	0	0	0	0
Liver	0	0	0	0	0	0	0	0	0
CNS/Brain	0	0	0	0	0	0	0	0	0
Other	0	0	0	0	0	0	0	0	0

What Methods were Used to Assess Disease Response/ Progression									
n	1	0	0	0	0	0	0	0	1
Diagnostic Image (MRI, CT)	1(100%)	0	0	0	0	0	0	0	1(100%)
Histopathologic Evaluation	0	0	0	0	0	0	0	0	0
Photography	1(100%)	0	0	0	0	0	0	0	1(100%)
Clinical Evaluation	1(100%)	0	0	0	0	0	0	0	1(100%)
Laboratory Evaluation	0	0	0	0	0	0	0	0	0
Other	0	0	0	0	0	0	0	0	0
					Cohort 1: Newly Determined Advanced BCC Patients (Non-BCCNS) Locally Advanced BCC (laBCC)				
			Vismo (N=115)			No Vismo (N=251)			
	All (N=433)	All (N=115)	Vismo Only (N=102)	Surgery Only (N=6)	Other Therapy (N=7)	All (N=251)	Surgery Only (N=198)	Other Therapy (N=53)	No Treatment (N=67)
Routine Follow-up 8									
Response Assessment									
n	83	42	37	1	4	35	25	10	6
Complete Response (CR)	53(63.9%)	20(47.6%)	17(45.9%)	1(100%)	2(50.0%)	30(85.7%)	22(88.0%)	8(80.0%)	3(50.0%)
Partial Response (PR)	6(7.2%)	6(14.3%)	5(13.5%)	0	1(25.0%)	0	0	0	0
Stable Disease (SD)	7(8.4%)	4(9.5%)	4(10.8%)	0	0	0	0	0	3(50.0%)
Disease Progression (PD)	9(10.8%)	5(11.9%)	5(13.5%)	0	0	4(11.4%)	2(8.0%)	2(20.0%)	0
Recurrence	7(8.4%)	7(16.7%)	6(16.2%)	0	1(25.0%)	0	0	0	0
Clinical Deterioration	0	0	0	0	0	0	0	0	0
Unable to Evaluate	1(1.2%)	0	0	0	0	1(2.9%)	1(4.0%)	0	0

If Complete Response, as Determined by[1]									
Negative Histopathology	2(3.8%)	0	0	0	0	2(6.7%)	2(9.1%)	0	0
Complete Tumor Shrinkage	8(15.1%)	5(25.0%)	5(29.4%)	0	0	3(10.0%)	1(4.5%)	2(25.0%)	0
No Clinically Visible Evidence of BCC	50(94.3%)	19(95.0%)	16(94.1%)	1(100%)	2(100%)	28(93.3%)	20(90.9%)	8(100%)	3(100%)
Other	0	0	0	0	0	0	0	0	0

					Cohort 1: Newly Determined Advanced BCC Patients (Non-BCCNS) Metastatic BCC (mBCC)				
		Vismo (N=2)			No Vismo (N=1)				
	All (N=4)	All (N=2)	Vismo Only (N=2)	Surgery Only (N=0)	Other Therapy (N=0)	All (N=1)	Surgery Only (N=1)	Other Therapy (N=0)	No Treatment (N=1)
Routine Follow-up 8									
Response Assessment									
n	0	0	0	0	0	0	0	0	0
Complete Response (CR)	0	0	0	0	0	0	0	0	0
Partial Response (PR)	0	0	0	0	0	0	0	0	0
Stable Disease (SD)	0	0	0	0	0	0	0	0	0
Disease Progression (PD)	0	0	0	0	0	0	0	0	0
Recurrence	0	0	0	0	0	0	0	0	0
Clinical Deterioration	0	0	0	0	0	0	0	0	0
Unable to Evaluate	0	0	0	0	0	0	0	0	0
If Complete Response, as Determined by[1]									

Negative Histopathology	0	0	0	0	0	0	0	0	0
Complete Tumor Shrinkage	0	0	0	0	0	0	0	0	0
No Clinically Visible Evidence of BCC	0	0	0	0	0	0	0	0	0
Other	0	0	0	0	0	0	0	0	0
					Cohort 1: Newly Determined Advanced BCC Patients (Non-BCCNS) Locally Advanced BCC (laBCC)				
			Vismo (N=115)			No Vismo (N=251)			
	All (N=433)	All (N=115)	Vismo Only (N=102)	Surgery Only (N=6)	Other Therapy (N=7)	All (N=251)	Surgery Only (N=198)	Other Therapy (N=53)	No Treatment (N=67)
Indicate How Response/ Progression was Assessed:									
n	41	30	28	0	2	8	4	4	3
Change in Size of Existing Lesions	28(68.3%)	21(70.0%)	20(71.4%)	0	1(50.0%)	4(50.0%)	1(25.0%)	3(75.0%)	3(100%)
Change in Number of Existing Lesions	13(31.7%)	10(33.3%)	9(32.1%)	0	1(50.0%)	2(25.0%)	0	2(50.0%)	1(33.3%)
Development of New BCCs	14(34.1%)	9(30.0%)	9(32.1%)	0	0	4(50.0%)	2(50.0%)	2(50.0%)	1(33.3%)
Progression from Locally Advanced Disease to Metastatic Disease	3(7.3%)	2(6.7%)	2(7.1%)	0	0	1(12.5%)	1(25.0%)	0	0
Development of New Metastases	0	0	0	0	0	0	0	0	0
Other	0	0	0	0	0	0	0	0	0

If progression to metastatic disease or new metastases, indicate site(s)[2]									
Bone	1(33.3%)	1(50.0%)	1(50.0%)	0	0	0	0	0	0
Lymph nodes, Local	0	0	0	0	0	0	0	0	0
Lymph nodes, Distant	1(33.3%)	1(50.0%)	1(50.0%)	0	0	0	0	0	0
Lung	0	0	0	0	0	0	0	0	0
Liver	0	0	0	0	0	0	0	0	0
CNS/Brain	0	0	0	0	0	0	0	0	0
Other	2(66.7%)	1(50.0%)	1(50.0%)	0	0	1(100%)	1(100%)	0	0
What Methods were Used to Assess Disease Response/ Progression									
n	83	42	37	1	4	35	25	10	6
Diagnostic Image (MRI, CT)	3(3.6%)	1(2.4%)	1(2.7%)	0	0	1(2.9%)	1(4.0%)	0	1(16.7%)
Histopathologic Evaluation	8(9.6%)	4(9.5%)	4(10.8%)	0	0	4(11.4%)	4(16.0%)	0	0
Photography	4(4.8%)	4(9.5%)	4(10.8%)	0	0	0	0	0	0
Clinical Evaluation	78(94.0%)	42(100%)	37(100%)	1(100%)	4(100%)	30(85.7%)	20(80.0%)	10(100%)	6(100%)
Laboratory Evaluation	1(1.2%)	0	0	0	0	1(2.9%)	1(4.0%)	0	0
Other	1(1.2%)	0	0	0	0	1(2.9%)	1(4.0%)	0	0
					Cohort 1: Newly Determined Advanced BCC Patients (Non-BCCNS) Metastatic BCC (mBCC)				
		Vismo (N=2)			No Vismo (N=1)				
	All (N=4)	All (N=2)	Vismo Only (N=2)	Surgery Only (N=0)	Other Therapy (N=0)	All (N=1)	Surgery Only (N=1)	Other Therapy (N=0)	No Treatment (N=1)

Indicate How Response/ Progression was Assessed:									
n	0	0	0	0	0	0	0	0	0
Change in Size of Existing Lesions	0	0	0	0	0	0	0	0	0
Change in Number of Existing Lesions	0	0	0	0	0	0	0	0	0
Development of New BCCs	0	0	0	0	0	0	0	0	0
Progression from Locally Advanced Disease to Metastatic Disease	0	0	0	0	0	0	0	0	0
Development of New Metastases	0	0	0	0	0	0	0	0	0
Other	0	0	0	0	0	0	0	0	0
If progression to metastatic disease or new metastases, indicate site(s)[2]									
Bone	0	0	0	0	0	0	0	0	0
Lymph nodes, Local	0	0	0	0	0	0	0	0	0
Lymph nodes, Distant	0	0	0	0	0	0	0	0	0
Lung	0	0	0	0	0	0	0	0	0
Liver	0	0	0	0	0	0	0	0	0
CNS/Brain	0	0	0	0	0	0	0	0	0
Other	0	0	0	0	0	0	0	0	0

What Methods were Used to Assess Disease Response/ Progression									
n	0	0	0	0	0	0	0	0	0
Diagnostic Image (MRI, CT)	0	0	0	0	0	0	0	0	0
Histopathologic Evaluation	0	0	0	0	0	0	0	0	0
Photography	0	0	0	0	0	0	0	0	0
Clinical Evaluation	0	0	0	0	0	0	0	0	0
Laboratory Evaluation	0	0	0	0	0	0	0	0	0
Other	0	0	0	0	0	0	0	0	0
					Cohort 1: Newly Determined Advanced BCC Patients (Non-BCCNS) Locally Advanced BCC (laBCC)				
			Vismo (N=115)			No Vismo (N=251)			
	All (N=433)	All (N=115)	Vismo Only (N=102)	Surgery Only (N=6)	Other Therapy (N=7)	All (N=251)	Surgery Only (N=198)	Other Therapy (N=53)	No Treatment (N=67)
Routine Follow-up 9									
Response Assessment									
n	62	29	28	0	1	28	20	8	5
Complete Response (CR)	40(64.5%)	14(48.3%)	14(50.0%)	0	0	24(85.7%)	17(85.0%)	7(87.5%)	2(40.0%)
Partial Response (PR)	6(9.7%)	5(17.2%)	4(14.3%)	0	1(100%)	1(3.6%)	1(5.0%)	0	0
Stable Disease (SD)	8(12.9%)	5(17.2%)	5(17.9%)	0	0	1(3.6%)	1(5.0%)	0	2(40.0%)
Disease Progression (PD)	4(6.5%)	3(10.3%)	3(10.7%)	0	0	1(3.6%)	0	1(12.5%)	0
Recurrence	2(3.2%)	2(6.9%)	2(7.1%)	0	0	0	0	0	0
Clinical Deterioration	0	0	0	0	0	0	0	0	0
Unable to Evaluate	2(3.2%)	0	0	0	0	1(3.6%)	1(5.0%)	0	1(20.0%)

If Complete Response, as Determined by[1]									
Negative Histopathology	5(12.5%)	2(14.3%)	2(14.3%)	0	0	3(12.5%)	2(11.8%)	1(14.3%)	0
Complete Tumor Shrinkage	8(20.0%)	3(21.4%)	3(21.4%)	0	0	4(16.7%)	1(5.9%)	3(42.9%)	1(50.0%)
No Clinically Visible Evidence of BCC	35(87.5%)	13(92.9%)	13(92.9%)	0	0	21(87.5%)	15(88.2%)	6(85.7%)	1(50.0%)
Other	0	0	0	0	0	0	0	0	0

					Cohort 1: Newly Determined Advanced BCC Patients (Non-BCCNS) Metastatic BCC (mBCC)				
			Vismo (N=2)			No Vismo (N=1)			
	All (N=4)	All (N=2)	Vismo Only (N=2)	Surgery Only (N=0)	Other Therapy (N=0)	All (N=1)	Surgery Only (N=1)	Other Therapy (N=0)	No Treatment (N=1)
Routine Follow-up 9									
Response Assessment									
n	0	0	0	0	0	0	0	0	0
Complete Response (CR)	0	0	0	0	0	0	0	0	0
Partial Response (PR)	0	0	0	0	0	0	0	0	0
Stable Disease (SD)	0	0	0	0	0	0	0	0	0
Disease Progression (PD)	0	0	0	0	0	0	0	0	0
Recurrence	0	0	0	0	0	0	0	0	0
Clinical Deterioration	0	0	0	0	0	0	0	0	0
Unable to Evaluate	0	0	0	0	0	0	0	0	0
If Complete Response, as Determined by[1]									

Negative Histopathology	0	0	0	0	0	0	0	0	0
Complete Tumor Shrinkage	0	0	0	0	0	0	0	0	0
No Clinically Visible Evidence of BCC	0	0	0	0	0	0	0	0	0
Other	0	0	0	0	0	0	0	0	0
					Cohort 1: Newly Determined Advanced BCC Patients (Non-BCCNS) Locally Advanced BCC (laBCC)				
			Vismo (N=115)		No Vismo (N=251)				
	All (N=433)	All (N=115)	Vismo Only (N=102)	Surgery Only (N=6)	Other Therapy (N=7)	All (N=251)	Surgery Only (N=198)	Other Therapy (N=53)	No Treatment (N=67)
Indicate How Response/ Progression was Assessed:									
n	26	17	17	0	0	6	3	3	3
Change in Size of Existing Lesions	18(69.2%)	11(64.7%)	11(64.7%)	0	0	5(83.3%)	2(66.7%)	3(100%)	2(66.7%)
Change in Number of Existing Lesions	3(11.5%)	3(17.6%)	3(17.6%)	0	0	0	0	0	0
Development of New BCCs	9(34.6%)	7(41.2%)	7(41.2%)	0	0	1(16.7%)	1(33.3%)	0	1(33.3%)
Progression from Locally Advanced Disease to Metastatic Disease	0	0	0	0	0	0	0	0	0
Development of New Metastases	0	0	0	0	0	0	0	0	0
Other	2(7.7%)	2(11.8%)	2(11.8%)	0	0	0	0	0	0

If progression to metastatic disease or new metastases, indicate site(s)[2]									
Bone	0	0	0	0	0	0	0	0	0
Lymph nodes, Local	0	0	0	0	0	0	0	0	0
Lymph nodes, Distant	0	0	0	0	0	0	0	0	0
Lung	0	0	0	0	0	0	0	0	0
Liver	0	0	0	0	0	0	0	0	0
CNS/Brain	0	0	0	0	0	0	0	0	0
Other	0	0	0	0	0	0	0	0	0
What Methods were Used to Assess Disease Response/ Progression									
n	62	29	28	0	1	28	20	8	5
Diagnostic Image (MRI, CT)	2(3.2%)	1(3.4%)	1(3.6%)	0	0	1(3.6%)	1(5.0%)	0	0
Histopathologic Evaluation	6(9.7%)	4(13.8%)	4(14.3%)	0	0	2(7.1%)	2(10.0%)	0	0
Photography	4(6.5%)	4(13.8%)	4(14.3%)	0	0	0	0	0	0
Clinical Evaluation	55(88.7%)	27(93.1%)	26(92.9%)	0	1(100%)	24(85.7%)	16(80.0%)	8(100%)	4(80.0%)
Laboratory Evaluation	0	0	0	0	0	0	0	0	0
Other	3(4.8%)	1(3.4%)	1(3.6%)	0	0	2(7.1%)	2(10.0%)	0	0
					Cohort 1: Newly Determined Advanced BCC Patients (Non-BCCNS) Metastatic BCC (mBCC)				
			Vismo (N=2)			No Vismo (N=1)			
	All (N=4)	All (N=2)	Vismo Only (N=2)	Surgery Only (N=0)	Other Therapy (N=0)	All (N=1)	Surgery Only (N=1)	Other Therapy (N=0)	No Treatment (N=1)

Indicate How Response/ Progression was Assessed:									
n	0	0	0	0	0	0	0	0	0
Change in Size of Existing Lesions	0	0	0	0	0	0	0	0	0
Change in Number of Existing Lesions	0	0	0	0	0	0	0	0	0
Development of New BCCs	0	0	0	0	0	0	0	0	0
Progression from Locally Advanced Disease to Metastatic Disease	0	0	0	0	0	0	0	0	0
Development of New Metastases	0	0	0	0	0	0	0	0	0
Other	0	0	0	0	0	0	0	0	0
If progression to metastatic disease or new metastases, indicate site(s)[2]									
Bone	0	0	0	0	0	0	0	0	0
Lymph nodes, Local	0	0	0	0	0	0	0	0	0
Lymph nodes, Distant	0	0	0	0	0	0	0	0	0
Lung	0	0	0	0	0	0	0	0	0
Liver	0	0	0	0	0	0	0	0	0
CNS/Brain	0	0	0	0	0	0	0	0	0
Other	0	0	0	0	0	0	0	0	0

What Methods were Used to Assess Disease Response/ Progression									
n	0	0	0	0	0	0	0	0	0
Diagnostic Image (MRI, CT)	0	0	0	0	0	0	0	0	0
Histopathologic Evaluation	0	0	0	0	0	0	0	0	0
Photography	0	0	0	0	0	0	0	0	0
Clinical Evaluation	0	0	0	0	0	0	0	0	0
Laboratory Evaluation	0	0	0	0	0	0	0	0	0
Other	0	0	0	0	0	0	0	0	0
					Cohort 1: Newly Determined Advanced BCC Patients (Non-BCCNS) Locally Advanced BCC (laBCC)				
			Vismo (N=115)			No Vismo (N=251)			
	All (N=433)	All (N=115)	Vismo Only (N=102)	Surgery Only (N=6)	Other Therapy (N=7)	All (N=251)	Surgery Only (N=198)	Other Therapy (N=53)	No Treatment (N=67)
Routine Follow-up 10									
Response Assessment									
n	52	24	23	0	1	24	17	7	4
Complete Response (CR)	31(59.6%)	9(37.5%)	9(39.1%)	0	0	20(83.3%)	14(82.4%)	6(85.7%)	2(50.0%)
Partial Response (PR)	4(7.7%)	3(12.5%)	3(13.0%)	0	0	1(4.2%)	0	1(14.3%)	0
Stable Disease (SD)	9(17.3%)	5(20.8%)	5(21.7%)	0	0	2(8.3%)	2(11.8%)	0	2(50.0%)
Disease Progression (PD)	2(3.8%)	2(8.3%)	1(4.3%)	0	1(100%)	0	0	0	0
Recurrence	5(9.6%)	5(20.8%)	5(21.7%)	0	0	0	0	0	0
Clinical Deterioration	0	0	0	0	0	0	0	0	0
Unable to Evaluate	1(1.9%)	0	0	0	0	1(4.2%)	1(5.9%)	0	0

If Complete Response, as Determined by[1]									
Negative Histopathology	5(16.1%)	1(11.1%)	1(11.1%)	0	0	4(20.0%)	2(14.3%)	2(33.3%)	0
Complete Tumor Shrinkage	7(22.6%)	1(11.1%)	1(11.1%)	0	0	5(25.0%)	1(7.1%)	4(66.7%)	1(50.0%)
No Clinically Visible Evidence of BCC	26(83.9%)	9(100%)	9(100%)	0	0	16(80.0%)	12(85.7%)	4(66.7%)	1(50.0%)
Other	0	0	0	0	0	0	0	0	0

					Cohort 1: Newly Determined Advanced BCC Patients (Non-BCCNS) Metastatic BCC (mBCC)				
			Vismo (N=2)			No Vismo (N=1)			
	All (N=4)	All (N=2)	Vismo Only (N=2)	Surgery Only (N=0)	Other Therapy (N=0)	All (N=1)	Surgery Only (N=1)	Other Therapy (N=0)	No Treatment (N=1)
Routine Follow-up 10									
Response Assessment									
n	0	0	0	0	0	0	0	0	0
Complete Response (CR)	0	0	0	0	0	0	0	0	0
Partial Response (PR)	0	0	0	0	0	0	0	0	0
Stable Disease (SD)	0	0	0	0	0	0	0	0	0
Disease Progression (PD)	0	0	0	0	0	0	0	0	0
Recurrence	0	0	0	0	0	0	0	0	0
Clinical Deterioration	0	0	0	0	0	0	0	0	0
Unable to Evaluate	0	0	0	0	0	0	0	0	0
If Complete Response, as Determined by[1]									

Negative Histopathology	0	0	0	0	0	0	0	0	0
Complete Tumor Shrinkage	0	0	0	0	0	0	0	0	0
No Clinically Visible Evidence of BCC	0	0	0	0	0	0	0	0	0
Other	0	0	0	0	0	0	0	0	0

					Cohort 1: Newly Determined Advanced BCC Patients (Non-BCCNS) Locally Advanced BCC (laBCC)				
		Vismo (N=115)			No Vismo (N=251)				
	All (N=433)	All (N=115)	Vismo Only (N=102)	Surgery Only (N=6)	Other Therapy (N=7)	All (N=251)	Surgery Only (N=198)	Other Therapy (N=53)	No Treatment (N=67)
Indicate How Response/ Progression was Assessed:									
n	18	14	13	0	1	2	1	1	2
Change in Size of Existing Lesions	15(83.3%)	12(85.7%)	11(84.6%)	0	1(100%)	1(50.0%)	0	1(100%)	2(100%)
Change in Number of Existing Lesions	3(16.7%)	2(14.3%)	2(15.4%)	0	0	0	0	0	1(50.0%)
Development of New BCCs	5(27.8%)	4(28.6%)	4(30.8%)	0	0	1(50.0%)	1(100%)	0	0
Progression from Locally Advanced Disease to Metastatic Disease	0	0	0	0	0	0	0	0	0
Development of New Metastases	0	0	0	0	0	0	0	0	0
Other	1(5.6%)	1(7.1%)	0	0	1(100%)	0	0	0	0

If progression to metastatic disease or new metastases, indicate site(s)[2]									
Bone	0	0	0	0	0	0	0	0	0
Lymph nodes, Local	0	0	0	0	0	0	0	0	0
Lymph nodes, Distant	0	0	0	0	0	0	0	0	0
Lung	0	0	0	0	0	0	0	0	0
Liver	0	0	0	0	0	0	0	0	0
CNS/Brain	0	0	0	0	0	0	0	0	0
Other	0	0	0	0	0	0	0	0	0
What Methods were Used to Assess Disease Response/ Progression									
n	52	24	23	0	1	24	17	7	4
Diagnostic Image (MRI, CT)	3(5.8%)	1(4.2%)	1(4.3%)	0	0	2(8.3%)	2(11.8%)	0	0
Histopathologic Evaluation	6(11.5%)	3(12.5%)	3(13.0%)	0	0	2(8.3%)	1(5.9%)	1(14.3%)	1(25.0%)
Photography	4(7.7%)	3(12.5%)	3(13.0%)	0	0	1(4.2%)	1(5.9%)	0	0
Clinical Evaluation	43(82.7%)	21(87.5%)	20(87.0%)	0	1(100%)	18(75.0%)	12(70.6%)	6(85.7%)	4(100%)
Laboratory Evaluation	1(1.9%)	1(4.2%)	1(4.3%)	0	0	0	0	0	0
Other	1(1.9%)	0	0	0	0	1(4.2%)	1(5.9%)	0	0
					Cohort 1: Newly Determined Advanced BCC Patients (Non-BCCNS) Metastatic BCC (mBCC)				
			Vismo (N=2)			No Vismo (N=1)			
	All (N=4)	All (N=2)	Vismo Only (N=2)	Surgery Only (N=0)	Other Therapy (N=0)	All (N=1)	Surgery Only (N=1)	Other Therapy (N=0)	No Treatment (N=1)

Indicate How Response/ Progression was Assessed:									
n	0	0	0	0	0	0	0	0	0
Change in Size of Existing Lesions	0	0	0	0	0	0	0	0	0
Change in Number of Existing Lesions	0	0	0	0	0	0	0	0	0
Development of New BCCs	0	0	0	0	0	0	0	0	0
Progression from Locally Advanced Disease to Metastatic Disease	0	0	0	0	0	0	0	0	0
Development of New Metastases	0	0	0	0	0	0	0	0	0
Other	0	0	0	0	0	0	0	0	0
If progression to metastatic disease or new metastases, indicate site(s)[2]									
Bone	0	0	0	0	0	0	0	0	0
Lymph nodes, Local	0	0	0	0	0	0	0	0	0
Lymph nodes, Distant	0	0	0	0	0	0	0	0	0
Lung	0	0	0	0	0	0	0	0	0
Liver	0	0	0	0	0	0	0	0	0
CNS/Brain	0	0	0	0	0	0	0	0	0
Other	0	0	0	0	0	0	0	0	0

What Methods were Used to Assess Disease Response/ Progression									
n	0	0	0	0	0	0	0	0	0
Diagnostic Image (MRI, CT)	0	0	0	0	0	0	0	0	0
Histopathologic Evaluation	0	0	0	0	0	0	0	0	0
Photography	0	0	0	0	0	0	0	0	0
Clinical Evaluation	0	0	0	0	0	0	0	0	0
Laboratory Evaluation	0	0	0	0	0	0	0	0	0
Other	0	0	0	0	0	0	0	0	0

					Cohort 1: Newly Determined Advanced BCC Patients (Non-BCCNS) Locally Advanced BCC (laBCC)				
		Vismo (N=115)			No Vismo (N=251)				
	All (N=433)	All (N=115)	Vismo Only (N=102)	Surgery Only (N=6)	Other Therapy (N=7)	All (N=251)	Surgery Only (N=198)	Other Therapy (N=53)	No Treatment (N=67)
Routine Follow-up 11									
Response Assessment									
n	40	17	16	0	1	19	14	5	4
Complete Response (CR)	27(67.5%)	8(47.1%)	8(50.0%)	0	0	17(89.5%)	12(85.7%)	5(100%)	2(50.0%)
Partial Response (PR)	4(10.0%)	4(23.5%)	4(25.0%)	0	0	0	0	0	0
Stable Disease (SD)	6(15.0%)	3(17.6%)	3(18.8%)	0	0	1(5.3%)	1(7.1%)	0	2(50.0%)
Disease Progression (PD)	1(2.5%)	1(5.9%)	0	0	1(100%)	0	0	0	0
Recurrence	1(2.5%)	1(5.9%)	1(6.3%)	0	0	0	0	0	0
Clinical Deterioration	0	0	0	0	0	0	0	0	0
Unable to Evaluate	1(2.5%)	0	0	0	0	1(5.3%)	1(7.1%)	0	0

If Complete Response, as Determined by[1]									
Negative Histopathology	4(14.8%)	0	0	0	0	4(23.5%)	2(16.7%)	2(40.0%)	0
Complete Tumor Shrinkage	3(11.1%)	1(12.5%)	1(12.5%)	0	0	2(11.8%)	0	2(40.0%)	0
No Clinically Visible Evidence of BCC	24(88.9%)	8(100%)	8(100%)	0	0	14(82.4%)	11(91.7%)	3(60.0%)	2(100%)
Other	0	0	0	0	0	0	0	0	0

					Cohort 1: Newly Determined Advanced BCC Patients (Non-BCCNS) Metastatic BCC (mBCC)				
		Vismo (N=2)			No Vismo (N=1)				
	All (N=4)	All (N=2)	Vismo Only (N=2)	Surgery Only (N=0)	Other Therapy (N=0)	All (N=1)	Surgery Only (N=1)	Other Therapy (N=0)	No Treatment (N=1)
Routine Follow-up 11									
Response Assessment									
n	0	0	0	0	0	0	0	0	0
Complete Response (CR)	0	0	0	0	0	0	0	0	0
Partial Response (PR)	0	0	0	0	0	0	0	0	0
Stable Disease (SD)	0	0	0	0	0	0	0	0	0
Disease Progression (PD)	0	0	0	0	0	0	0	0	0
Recurrence	0	0	0	0	0	0	0	0	0
Clinical Deterioration	0	0	0	0	0	0	0	0	0
Unable to Evaluate	0	0	0	0	0	0	0	0	0
If Complete Response, as Determined by[1]									

Negative Histopathology	0	0	0	0	0	0	0	0	0
Complete Tumor Shrinkage	0	0	0	0	0	0	0	0	0
No Clinically Visible Evidence of BCC	0	0	0	0	0	0	0	0	0
Other	0	0	0	0	0	0	0	0	0
					Cohort 1: Newly Determined Advanced BCC Patients (Non-BCCNS) Locally Advanced BCC (laBCC)				
			Vismo (N=115)			No Vismo (N=251)			
	All (N=433)	All (N=115)	Vismo Only (N=102)	Surgery Only (N=6)	Other Therapy (N=7)	All (N=251)	Surgery Only (N=198)	Other Therapy (N=53)	No Treatment (N=67)
Indicate How Response/ Progression was Assessed:									
n	12	8	7	0	1	3	1	2	1
Change in Size of Existing Lesions	9(75.0%)	7(87.5%)	7(100%)	0	0	1(33.3%)	0	1(50.0%)	1(100%)
Change in Number of Existing Lesions	3(25.0%)	2(25.0%)	2(28.6%)	0	0	0	0	0	1(100%)
Development of New BCCs	2(16.7%)	1(12.5%)	1(14.3%)	0	0	1(33.3%)	1(100%)	0	0
Progression from Locally Advanced Disease to Metastatic Disease	0	0	0	0	0	0	0	0	0
Development of New Metastases	0	0	0	0	0	0	0	0	0
Other	2(16.7%)	1(12.5%)	0	0	1(100%)	1(33.3%)	0	1(50.0%)	0

If progression to metastatic disease or new metastases, indicate site(s)[2]									
Bone	0	0	0	0	0	0	0	0	0
Lymph nodes, Local	0	0	0	0	0	0	0	0	0
Lymph nodes, Distant	0	0	0	0	0	0	0	0	0
Lung	0	0	0	0	0	0	0	0	0
Liver	0	0	0	0	0	0	0	0	0
CNS/Brain	0	0	0	0	0	0	0	0	0
Other	0	0	0	0	0	0	0	0	0
What Methods were Used to Assess Disease Response/ Progression									
n	40	17	16	0	1	19	14	5	4
Diagnostic Image (MRI, CT)	1(2.5%)	1(5.9%)	1(6.3%)	0	0	0	0	0	0
Histopathologic Evaluation	3(7.5%)	1(5.9%)	1(6.3%)	0	0	2(10.5%)	2(14.3%)	0	0
Photography	1(2.5%)	1(5.9%)	1(6.3%)	0	0	0	0	0	0
Clinical Evaluation	38(95.0%)	17(100%)	16(100%)	0	1(100%)	17(89.5%)	12(85.7%)	5(100%)	4(100%)
Laboratory Evaluation	0	0	0	0	0	0	0	0	0
Other	1(2.5%)	0	0	0	0	1(5.3%)	1(7.1%)	0	0
					Cohort 1: Newly Determined Advanced BCC Patients (Non-BCCNS) Metastatic BCC (mBCC)				
			Vismo (N=2)			No Vismo (N=1)			
	All (N=4)	All (N=2)	Vismo Only (N=2)	Surgery Only (N=0)	Other Therapy (N=0)	All (N=1)	Surgery Only (N=1)	Other Therapy (N=0)	No Treatment (N=1)

Indicate How Response/ Progression was Assessed:									
n	0	0	0	0	0	0	0	0	0
Change in Size of Existing Lesions	0	0	0	0	0	0	0	0	0
Change in Number of Existing Lesions	0	0	0	0	0	0	0	0	0
Development of New BCCs	0	0	0	0	0	0	0	0	0
Progression from Locally Advanced Disease to Metastatic Disease	0	0	0	0	0	0	0	0	0
Development of New Metastases	0	0	0	0	0	0	0	0	0
Other	0	0	0	0	0	0	0	0	0
If progression to metastatic disease or new metastases, indicate site(s)[2]									
Bone	0	0	0	0	0	0	0	0	0
Lymph nodes, Local	0	0	0	0	0	0	0	0	0
Lymph nodes, Distant	0	0	0	0	0	0	0	0	0
Lung	0	0	0	0	0	0	0	0	0
Liver	0	0	0	0	0	0	0	0	0
CNS/Brain	0	0	0	0	0	0	0	0	0
Other	0	0	0	0	0	0	0	0	0

What Methods were Used to Assess Disease Response/ Progression									
n	0	0	0	0	0	0	0	0	0
Diagnostic Image (MRI, CT)	0	0	0	0	0	0	0	0	0
Histopathologic Evaluation	0	0	0	0	0	0	0	0	0
Photography	0	0	0	0	0	0	0	0	0
Clinical Evaluation	0	0	0	0	0	0	0	0	0
Laboratory Evaluation	0	0	0	0	0	0	0	0	0
Other	0	0	0	0	0	0	0	0	0

					Cohort 1: Newly Determined Advanced BCC Patients (Non-BCCNS) Locally Advanced BCC (laBCC)				
		Vismo (N=115)			No Vismo (N=251)				
	All (N=433)	All (N=115)	Vismo Only (N=102)	Surgery Only (N=6)	Other Therapy (N=7)	All (N=251)	Surgery Only (N=198)	Other Therapy (N=53)	No Treatment (N=67)
Routine Follow-up 12									
Response Assessment									
n	22	8	8	0	0	13	9	4	1
Complete Response (CR)	16(72.7%)	4(50.0%)	4(50.0%)	0	0	12(92.3%)	8(88.9%)	4(100%)	0
Partial Response (PR)	1(4.5%)	1(12.5%)	1(12.5%)	0	0	0	0	0	0
Stable Disease (SD)	2(9.1%)	1(12.5%)	1(12.5%)	0	0	0	0	0	1(100%)
Disease Progression (PD)	1(4.5%)	1(12.5%)	1(12.5%)	0	0	0	0	0	0
Recurrence	1(4.5%)	1(12.5%)	1(12.5%)	0	0	0	0	0	0
Clinical Deterioration	0	0	0	0	0	0	0	0	0
Unable to Evaluate	1(4.5%)	0	0	0	0	1(7.7%)	1(11.1%)	0	0

If Complete Response, as Determined by[1]									
Negative Histopathology	1(6.3%)	0	0	0	0	1(8.3%)	0	1(25.0%)	0
Complete Tumor Shrinkage	2(12.5%)	0	0	0	0	2(16.7%)	0	2(50.0%)	0
No Clinically Visible Evidence of BCC	15(93.8%)	4(100%)	4(100%)	0	0	11(91.7%)	8(100%)	3(75.0%)	0
Other	0	0	0	0	0	0	0	0	0
					Cohort 1: Newly Determined Advanced BCC Patients (Non-BCCNS) Metastatic BCC (mBCC)				
			Vismo (N=2)		No Vismo (N=1)				
	All (N=4)	All (N=2)	Vismo Only (N=2)	Surgery Only (N=0)	Other Therapy (N=0)	All (N=1)	Surgery Only (N=1)	Other Therapy (N=0)	No Treatment (N=1)
Routine Follow-up 12									
Response Assessment									
n	0	0	0	0	0	0	0	0	0
Complete Response (CR)	0	0	0	0	0	0	0	0	0
Partial Response (PR)	0	0	0	0	0	0	0	0	0
Stable Disease (SD)	0	0	0	0	0	0	0	0	0
Disease Progression (PD)	0	0	0	0	0	0	0	0	0
Recurrence	0	0	0	0	0	0	0	0	0
Clinical Deterioration	0	0	0	0	0	0	0	0	0
Unable to Evaluate	0	0	0	0	0	0	0	0	0
If Complete Response, as Determined by[1]									

Negative Histopathology	0	0	0	0	0	0	0	0	0
Complete Tumor Shrinkage	0	0	0	0	0	0	0	0	0
No Clinically Visible Evidence of BCC	0	0	0	0	0	0	0	0	0
Other	0	0	0	0	0	0	0	0	0
					Cohort 1: Newly Determined Advanced BCC Patients (Non-BCCNS) Locally Advanced BCC (laBCC)				
			Vismo (N=115)			No Vismo (N=251)			
	All (N=433)	All (N=115)	Vismo Only (N=102)	Surgery Only (N=6)	Other Therapy (N=7)	All (N=251)	Surgery Only (N=198)	Other Therapy (N=53)	No Treatment (N=67)
Indicate How Response/ Progression was Assessed:									
n	10	5	5	0	0	4	2	2	1
Change in Size of Existing Lesions	7(70.0%)	4(80.0%)	4(80.0%)	0	0	2(50.0%)	0	2(100%)	1(100%)
Change in Number of Existing Lesions	2(20.0%)	1(20.0%)	1(20.0%)	0	0	0	0	0	1(100%)
Development of New BCCs	3(30.0%)	1(20.0%)	1(20.0%)	0	0	2(50.0%)	2(100%)	0	0
Progression from Locally Advanced Disease to Metastatic Disease	0	0	0	0	0	0	0	0	0
Development of New Metastases	0	0	0	0	0	0	0	0	0
Other	0	0	0	0	0	0	0	0	0

If progression to metastatic disease or new metastases, indicate site(s)[2]									
Bone	0	0	0	0	0	0	0	0	0
Lymph nodes, Local	0	0	0	0	0	0	0	0	0
Lymph nodes, Distant	0	0	0	0	0	0	0	0	0
Lung	0	0	0	0	0	0	0	0	0
Liver	0	0	0	0	0	0	0	0	0
CNS/Brain	0	0	0	0	0	0	0	0	0
Other	0	0	0	0	0	0	0	0	0
What Methods were Used to Assess Disease Response/ Progression									
n	22	8	8	0	0	13	9	4	1
Diagnostic Image (MRI, CT)	1(4.5%)	1(12.5%)	1(12.5%)	0	0	0	0	0	0
Histopathologic Evaluation	0	0	0	0	0	0	0	0	0
Photography	1(4.5%)	1(12.5%)	1(12.5%)	0	0	0	0	0	0
Clinical Evaluation	21(95.5%)	8(100%)	8(100%)	0	0	12(92.3%)	8(88.9%)	4(100%)	1(100%)
Laboratory Evaluation	0	0	0	0	0	0	0	0	0
Other	1(4.5%)	0	0	0	0	1(7.7%)	1(11.1%)	0	0
					Cohort 1: Newly Determined Advanced BCC Patients (Non-BCCNS) Metastatic BCC (mBCC)				
			Vismo (N=2)		No Vismo (N=1)				
	All (N=4)	All (N=2)	Vismo Only (N=2)	Surgery Only (N=0)	Other Therapy (N=0)	All (N=1)	Surgery Only (N=1)	Other Therapy (N=0)	No Treatment (N=1)

Indicate How Response/ Progression was Assessed:									
n	0	0	0	0	0	0	0	0	0
Change in Size of Existing Lesions	0	0	0	0	0	0	0	0	0
Change in Number of Existing Lesions	0	0	0	0	0	0	0	0	0
Development of New BCCs	0	0	0	0	0	0	0	0	0
Progression from Locally Advanced Disease to Metastatic Disease	0	0	0	0	0	0	0	0	0
Development of New Metastases	0	0	0	0	0	0	0	0	0
Other	0	0	0	0	0	0	0	0	0
If progression to metastatic disease or new metastases, indicate site(s)[2]									
Bone	0	0	0	0	0	0	0	0	0
Lymph nodes, Local	0	0	0	0	0	0	0	0	0
Lymph nodes, Distant	0	0	0	0	0	0	0	0	0
Lung	0	0	0	0	0	0	0	0	0
Liver	0	0	0	0	0	0	0	0	0
CNS/Brain	0	0	0	0	0	0	0	0	0
Other	0	0	0	0	0	0	0	0	0

What Methods were Used to Assess Disease Response/ Progression									
n	0	0	0	0	0	0	0	0	0
Diagnostic Image (MRI, CT)	0	0	0	0	0	0	0	0	0
Histopathologic Evaluation	0	0	0	0	0	0	0	0	0
Photography	0	0	0	0	0	0	0	0	0
Clinical Evaluation	0	0	0	0	0	0	0	0	0
Laboratory Evaluation	0	0	0	0	0	0	0	0	0
Other	0	0	0	0	0	0	0	0	0
					Cohort 1: Newly Determined Advanced BCC Patients (Non-BCCNS) Locally Advanced BCC (laBCC)				
			Vismo (N=115)			No Vismo (N=251)			
	All (N=433)	All (N=115)	Vismo Only (N=102)	Surgery Only (N=6)	Other Therapy (N=7)	All (N=251)	Surgery Only (N=198)	Other Therapy (N=53)	No Treatment (N=67)
Routine Follow-up 13									
Response Assessment									
n	14	6	6	0	0	8	7	1	0
Complete Response (CR)	11(78.6%)	3(50.0%)	3(50.0%)	0	0	8(100%)	7(100%)	1(100%)	0
Partial Response (PR)	1(7.1%)	1(16.7%)	1(16.7%)	0	0	0	0	0	0
Stable Disease (SD)	1(7.1%)	1(16.7%)	1(16.7%)	0	0	0	0	0	0
Disease Progression (PD)	0	0	0	0	0	0	0	0	0
Recurrence	1(7.1%)	1(16.7%)	1(16.7%)	0	0	0	0	0	0
Clinical Deterioration	0	0	0	0	0	0	0	0	0
Unable to Evaluate	0	0	0	0	0	0	0	0	0

If Complete Response, as Determined by[1]									
Negative Histopathology	0	0	0	0	0	0	0	0	0
Complete Tumor Shrinkage	1(9.1%)	0	0	0	0	1(12.5%)	0	1(100%)	0
No Clinically Visible Evidence of BCC	11(100%)	3(100%)	3(100%)	0	0	8(100%)	7(100%)	1(100%)	0
Other	0	0	0	0	0	0	0	0	0

					Cohort 1: Newly Determined Advanced BCC Patients (Non-BCCNS) Metastatic BCC (mBCC)				
		Vismo (N=2)			No Vismo (N=1)				
	All (N=4)	All (N=2)	Vismo Only (N=2)	Surgery Only (N=0)	Other Therapy (N=0)	All (N=1)	Surgery Only (N=1)	Other Therapy (N=0)	No Treatment (N=1)
Routine Follow-up 13									
Response Assessment									
n	0	0	0	0	0	0	0	0	0
Complete Response (CR)	0	0	0	0	0	0	0	0	0
Partial Response (PR)	0	0	0	0	0	0	0	0	0
Stable Disease (SD)	0	0	0	0	0	0	0	0	0
Disease Progression (PD)	0	0	0	0	0	0	0	0	0
Recurrence	0	0	0	0	0	0	0	0	0
Clinical Deterioration	0	0	0	0	0	0	0	0	0
Unable to Evaluate	0	0	0	0	0	0	0	0	0
If Complete Response, as Determined by[1]									

Negative Histopathology	0	0	0	0	0	0	0	0	0
Complete Tumor Shrinkage	0	0	0	0	0	0	0	0	0
No Clinically Visible Evidence of BCC	0	0	0	0	0	0	0	0	0
Other	0	0	0	0	0	0	0	0	0
					Cohort 1: Newly Determined Advanced BCC Patients (Non-BCCNS) Locally Advanced BCC (laBCC)				
		Vismo (N=115)			No Vismo (N=251)				
	All (N=433)	All (N=115)	Vismo Only (N=102)	Surgery Only (N=6)	Other Therapy (N=7)	All (N=251)	Surgery Only (N=198)	Other Therapy (N=53)	No Treatment (N=67)
Indicate How Response/ Progression was Assessed:									
n	3	3	3	0	0	0	0	0	0
Change in Size of Existing Lesions	3(100%)	3(100%)	3(100%)	0	0	0	0	0	0
Change in Number of Existing Lesions	1(33.3%)	1(33.3%)	1(33.3%)	0	0	0	0	0	0
Development of New BCCs	1(33.3%)	1(33.3%)	1(33.3%)	0	0	0	0	0	0
Progression from Locally Advanced Disease to Metastatic Disease	0	0	0	0	0	0	0	0	0
Development of New Metastases	0	0	0	0	0	0	0	0	0
Other	0	0	0	0	0	0	0	0	0

If progression to metastatic disease or new metastases, indicate site(s)[2]									
Bone	0	0	0	0	0	0	0	0	0
Lymph nodes, Local	0	0	0	0	0	0	0	0	0
Lymph nodes, Distant	0	0	0	0	0	0	0	0	0
Lung	0	0	0	0	0	0	0	0	0
Liver	0	0	0	0	0	0	0	0	0
CNS/Brain	0	0	0	0	0	0	0	0	0
Other	0	0	0	0	0	0	0	0	0
What Methods were Used to Assess Disease Response/ Progression									
n	14	6	6	0	0	8	7	1	0
Diagnostic Image (MRI, CT)	1(7.1%)	1(16.7%)	1(16.7%)	0	0	0	0	0	0
Histopathologic Evaluation	0	0	0	0	0	0	0	0	0
Photography	1(7.1%)	1(16.7%)	1(16.7%)	0	0	0	0	0	0
Clinical Evaluation	13(92.9%)	5(83.3%)	5(83.3%)	0	0	8(100%)	7(100%)	1(100%)	0
Laboratory Evaluation	0	0	0	0	0	0	0	0	0
Other	1(7.1%)	1(16.7%)	1(16.7%)	0	0	0	0	0	0
					Cohort 1: Newly Determined Advanced BCC Patients (Non-BCCNS) Metastatic BCC (mBCC)				
			Vismo (N=2)			No Vismo (N=1)			
	All (N=4)	All (N=2)	Vismo Only (N=2)	Surgery Only (N=0)	Other Therapy (N=0)	All (N=1)	Surgery Only (N=1)	Other Therapy (N=0)	No Treatment (N=1)

Indicate How Response/ Progression was Assessed:									
n	0	0	0	0	0	0	0	0	0
Change in Size of Existing Lesions	0	0	0	0	0	0	0	0	0
Change in Number of Existing Lesions	0	0	0	0	0	0	0	0	0
Development of New BCCs	0	0	0	0	0	0	0	0	0
Progression from Locally Advanced Disease to Metastatic Disease	0	0	0	0	0	0	0	0	0
Development of New Metastases	0	0	0	0	0	0	0	0	0
Other	0	0	0	0	0	0	0	0	0
If progression to metastatic disease or new metastases, indicate site(s)[2]									
Bone	0	0	0	0	0	0	0	0	0
Lymph nodes, Local	0	0	0	0	0	0	0	0	0
Lymph nodes, Distant	0	0	0	0	0	0	0	0	0
Lung	0	0	0	0	0	0	0	0	0
Liver	0	0	0	0	0	0	0	0	0
CNS/Brain	0	0	0	0	0	0	0	0	0
Other	0	0	0	0	0	0	0	0	0

What Methods were Used to Assess Disease Response/ Progression									
n	0	0	0	0	0	0	0	0	0
Diagnostic Image (MRI, CT)	0	0	0	0	0	0	0	0	0
Histopathologic Evaluation	0	0	0	0	0	0	0	0	0
Photography	0	0	0	0	0	0	0	0	0
Clinical Evaluation	0	0	0	0	0	0	0	0	0
Laboratory Evaluation	0	0	0	0	0	0	0	0	0
Other	0	0	0	0	0	0	0	0	0

					Cohort 1: Newly Determined Advanced BCC Patients (Non-BCCNS) Locally Advanced BCC (laBCC)				
		Vismo (N=115)			No Vismo (N=251)				
	All (N=433)	All (N=115)	Vismo Only (N=102)	Surgery Only (N=6)	Other Therapy (N=7)	All (N=251)	Surgery Only (N=198)	Other Therapy (N=53)	No Treatment (N=67)
Routine Follow-up 14									
Response Assessment									
n	12	5	5	0	0	7	6	1	0
Complete Response (CR)	9(75.0%)	2(40.0%)	2(40.0%)	0	0	7(100%)	6(100%)	1(100%)	0
Partial Response (PR)	0	0	0	0	0	0	0	0	0
Stable Disease (SD)	1(8.3%)	1(20.0%)	1(20.0%)	0	0	0	0	0	0
Disease Progression (PD)	0	0	0	0	0	0	0	0	0
Recurrence	2(16.7%)	2(40.0%)	2(40.0%)	0	0	0	0	0	0
Clinical Deterioration	0	0	0	0	0	0	0	0	0
Unable to Evaluate	0	0	0	0	0	0	0	0	0

If Complete Response, as Determined by[1]									
Negative Histopathology	0	0	0	0	0	0	0	0	0
Complete Tumor Shrinkage	1(11.1%)	0	0	0	0	1(14.3%)	0	1(100%)	0
No Clinically Visible Evidence of BCC	9(100%)	2(100%)	2(100%)	0	0	7(100%)	6(100%)	1(100%)	0
Other	0	0	0	0	0	0	0	0	0
					Cohort 1: Newly Determined Advanced BCC Patients (Non-BCCNS) Metastatic BCC (mBCC)				
			Vismo (N=2)			No Vismo (N=1)			
	All (N=4)	All (N=2)	Vismo Only (N=2)	Surgery Only (N=0)	Other Therapy (N=0)	All (N=1)	Surgery Only (N=1)	Other Therapy (N=0)	No Treatment (N=1)
Routine Follow-up 14									
Response Assessment									
n	0	0	0	0	0	0	0	0	0
Complete Response (CR)	0	0	0	0	0	0	0	0	0
Partial Response (PR)	0	0	0	0	0	0	0	0	0
Stable Disease (SD)	0	0	0	0	0	0	0	0	0
Disease Progression (PD)	0	0	0	0	0	0	0	0	0
Recurrence	0	0	0	0	0	0	0	0	0
Clinical Deterioration	0	0	0	0	0	0	0	0	0
Unable to Evaluate	0	0	0	0	0	0	0	0	0
If Complete Response, as Determined by[1]									

Negative Histopathology	0	0	0	0	0	0	0	0	0
Complete Tumor Shrinkage	0	0	0	0	0	0	0	0	0
No Clinically Visible Evidence of BCC	0	0	0	0	0	0	0	0	0
Other	0	0	0	0	0	0	0	0	0

					Cohort 1: Newly Determined Advanced BCC Patients (Non-BCCNS) Locally Advanced BCC (laBCC)				
		Vismo (N=115)			No Vismo (N=251)				
	All (N=433)	All (N=115)	Vismo Only (N=102)	Surgery Only (N=6)	Other Therapy (N=7)	All (N=251)	Surgery Only (N=198)	Other Therapy (N=53)	No Treatment (N=67)
Indicate How Response/ Progression was Assessed:									
n	3	2	2	0	0	1	0	1	0
Change in Size of Existing Lesions	1(33.3%)	0	0	0	0	1(100%)	0	1(100%)	0
Change in Number of Existing Lesions	2(66.7%)	2(100%)	2(100%)	0	0	0	0	0	0
Development of New BCCs	0	0	0	0	0	0	0	0	0
Progression from Locally Advanced Disease to Metastatic Disease	0	0	0	0	0	0	0	0	0
Development of New Metastases	0	0	0	0	0	0	0	0	0
Other	0	0	0	0	0	0	0	0	0

If progression to metastatic disease or new metastases, indicate site(s)[2]									
Bone	0	0	0	0	0	0	0	0	0
Lymph nodes, Local	0	0	0	0	0	0	0	0	0
Lymph nodes, Distant	0	0	0	0	0	0	0	0	0
Lung	0	0	0	0	0	0	0	0	0
Liver	0	0	0	0	0	0	0	0	0
CNS/Brain	0	0	0	0	0	0	0	0	0
Other	0	0	0	0	0	0	0	0	0
What Methods were Used to Assess Disease Response/ Progression									
n	12	5	5	0	0	7	6	1	0
Diagnostic Image (MRI, CT)	1(8.3%)	1(20.0%)	1(20.0%)	0	0	0	0	0	0
Histopathologic Evaluation	1(8.3%)	1(20.0%)	1(20.0%)	0	0	0	0	0	0
Photography	0	0	0	0	0	0	0	0	0
Clinical Evaluation	10(83.3%)	3(60.0%)	3(60.0%)	0	0	7(100%)	6(100%)	1(100%)	0
Laboratory Evaluation	1(8.3%)	1(20.0%)	1(20.0%)	0	0	0	0	0	0
Other	0	0	0	0	0	0	0	0	0
					Cohort 1: Newly Determined Advanced BCC Patients (Non-BCCNS) Metastatic BCC (mBCC)				
			Vismo (N=2)			No Vismo (N=1)			
	All (N=4)	All (N=2)	Vismo Only (N=2)	Surgery Only (N=0)	Other Therapy (N=0)	All (N=1)	Surgery Only (N=1)	Other Therapy (N=0)	No Treatment (N=1)

Indicate How Response/ Progression was Assessed:									
n	0	0	0	0	0	0	0	0	0
Change in Size of Existing Lesions	0	0	0	0	0	0	0	0	0
Change in Number of Existing Lesions	0	0	0	0	0	0	0	0	0
Development of New BCCs	0	0	0	0	0	0	0	0	0
Progression from Locally Advanced Disease to Metastatic Disease	0	0	0	0	0	0	0	0	0
Development of New Metastases	0	0	0	0	0	0	0	0	0
Other	0	0	0	0	0	0	0	0	0
If progression to metastatic disease or new metastases, indicate site(s)[2]									
Bone	0	0	0	0	0	0	0	0	0
Lymph nodes, Local	0	0	0	0	0	0	0	0	0
Lymph nodes, Distant	0	0	0	0	0	0	0	0	0
Lung	0	0	0	0	0	0	0	0	0
Liver	0	0	0	0	0	0	0	0	0
CNS/Brain	0	0	0	0	0	0	0	0	0
Other	0	0	0	0	0	0	0	0	0

What Methods were Used to Assess Disease Response/ Progression									
n	0	0	0	0	0	0	0	0	0
Diagnostic Image (MRI, CT)	0	0	0	0	0	0	0	0	0
Histopathologic Evaluation	0	0	0	0	0	0	0	0	0
Photography	0	0	0	0	0	0	0	0	0
Clinical Evaluation	0	0	0	0	0	0	0	0	0
Laboratory Evaluation	0	0	0	0	0	0	0	0	0
Other	0	0	0	0	0	0	0	0	0
					Cohort 1: Newly Determined Advanced BCC Patients (Non-BCCNS) Locally Advanced BCC (laBCC)				
			Vismo (N=115)		No Vismo (N=251)				
	All (N=433)	All (N=115)	Vismo Only (N=102)	Surgery Only (N=6)	Other Therapy (N=7)	All (N=251)	Surgery Only (N=198)	Other Therapy (N=53)	No Treatment (N=67)
Routine Follow-up 15									
Response Assessment									
n	8	4	4	0	0	4	4	0	0
Complete Response (CR)	6(75.0%)	2(50.0%)	2(50.0%)	0	0	4(100%)	4(100%)	0	0
Partial Response (PR)	0	0	0	0	0	0	0	0	0
Stable Disease (SD)	1(12.5%)	1(25.0%)	1(25.0%)	0	0	0	0	0	0
Disease Progression (PD)	0	0	0	0	0	0	0	0	0
Recurrence	1(12.5%)	1(25.0%)	1(25.0%)	0	0	0	0	0	0
Clinical Deterioration	0	0	0	0	0	0	0	0	0
Unable to Evaluate	0	0	0	0	0	0	0	0	0

If Complete Response, as Determined by[1]									
Negative Histopathology	2(33.3%)	1(50.0%)	1(50.0%)	0	0	1(25.0%)	1(25.0%)	0	0
Complete Tumor Shrinkage	0	0	0	0	0	0	0	0	0
No Clinically Visible Evidence of BCC	5(83.3%)	1(50.0%)	1(50.0%)	0	0	4(100%)	4(100%)	0	0
Other	0	0	0	0	0	0	0	0	0
					Cohort 1: Newly Determined Advanced BCC Patients (Non-BCCNS) Metastatic BCC (mBCC)				
			Vismo (N=2)			No Vismo (N=1)			
	All (N=4)	All (N=2)	Vismo Only (N=2)	Surgery Only (N=0)	Other Therapy (N=0)	All (N=1)	Surgery Only (N=1)	Other Therapy (N=0)	No Treatment (N=1)
Routine Follow-up 15									
Response Assessment									
n	0	0	0	0	0	0	0	0	0
Complete Response (CR)	0	0	0	0	0	0	0	0	0
Partial Response (PR)	0	0	0	0	0	0	0	0	0
Stable Disease (SD)	0	0	0	0	0	0	0	0	0
Disease Progression (PD)	0	0	0	0	0	0	0	0	0
Recurrence	0	0	0	0	0	0	0	0	0
Clinical Deterioration	0	0	0	0	0	0	0	0	0
Unable to Evaluate	0	0	0	0	0	0	0	0	0
If Complete Response, as Determined by[1]									

Negative Histopathology	0	0	0	0	0	0	0	0	0
Complete Tumor Shrinkage	0	0	0	0	0	0	0	0	0
No Clinically Visible Evidence of BCC	0	0	0	0	0	0	0	0	0
Other	0	0	0	0	0	0	0	0	0

					Cohort 1: Newly Determined Advanced BCC Patients (Non-BCCNS) Locally Advanced BCC (laBCC)				
		Vismo (N=115)			No Vismo (N=251)				
	All (N=433)	All (N=115)	Vismo Only (N=102)	Surgery Only (N=6)	Other Therapy (N=7)	All (N=251)	Surgery Only (N=198)	Other Therapy (N=53)	No Treatment (N=67)
Indicate How Response/ Progression was Assessed:									
n	3	3	3	0	0	0	0	0	0
Change in Size of Existing Lesions	2(66.7%)	2(66.7%)	2(66.7%)	0	0	0	0	0	0
Change in Number of Existing Lesions	2(66.7%)	2(66.7%)	2(66.7%)	0	0	0	0	0	0
Development of New BCCs	0	0	0	0	0	0	0	0	0
Progression from Locally Advanced Disease to Metastatic Disease	0	0	0	0	0	0	0	0	0
Development of New Metastases	0	0	0	0	0	0	0	0	0
Other	0	0	0	0	0	0	0	0	0

If progression to metastatic disease or new metastases, indicate site(s)[2]									
Bone	0	0	0	0	0	0	0	0	0
Lymph nodes, Local	0	0	0	0	0	0	0	0	0
Lymph nodes, Distant	0	0	0	0	0	0	0	0	0
Lung	0	0	0	0	0	0	0	0	0
Liver	0	0	0	0	0	0	0	0	0
CNS/Brain	0	0	0	0	0	0	0	0	0
Other	0	0	0	0	0	0	0	0	0
What Methods were Used to Assess Disease Response/ Progression									
n	8	4	4	0	0	4	4	0	0
Diagnostic Image (MRI, CT)	0	0	0	0	0	0	0	0	0
Histopathologic Evaluation	2(25.0%)	1(25.0%)	1(25.0%)	0	0	1(25.0%)	1(25.0%)	0	0
Photography	0	0	0	0	0	0	0	0	0
Clinical Evaluation	7(87.5%)	3(75.0%)	3(75.0%)	0	0	4(100%)	4(100%)	0	0
Laboratory Evaluation	0	0	0	0	0	0	0	0	0
Other	0	0	0	0	0	0	0	0	0
					Cohort 1: Newly Determined Advanced BCC Patients (Non-BCCNS) Metastatic BCC (mBCC)				
			Vismo (N=2)			No Vismo (N=1)			
	All (N=4)	All (N=2)	Vismo Only (N=2)	Surgery Only (N=0)	Other Therapy (N=0)	All (N=1)	Surgery Only (N=1)	Other Therapy (N=0)	No Treatment (N=1)

Indicate How Response/ Progression was Assessed:									
n	0	0	0	0	0	0	0	0	0
Change in Size of Existing Lesions	0	0	0	0	0	0	0	0	0
Change in Number of Existing Lesions	0	0	0	0	0	0	0	0	0
Development of New BCCs	0	0	0	0	0	0	0	0	0
Progression from Locally Advanced Disease to Metastatic Disease	0	0	0	0	0	0	0	0	0
Development of New Metastases	0	0	0	0	0	0	0	0	0
Other	0	0	0	0	0	0	0	0	0
If progression to metastatic disease or new metastases, indicate site(s)[2]									
Bone	0	0	0	0	0	0	0	0	0
Lymph nodes, Local	0	0	0	0	0	0	0	0	0
Lymph nodes, Distant	0	0	0	0	0	0	0	0	0
Lung	0	0	0	0	0	0	0	0	0
Liver	0	0	0	0	0	0	0	0	0
CNS/Brain	0	0	0	0	0	0	0	0	0
Other	0	0	0	0	0	0	0	0	0

What Methods were Used to Assess Disease Response/ Progression									
n	0	0	0	0	0	0	0	0	0
Diagnostic Image (MRI, CT)	0	0	0	0	0	0	0	0	0
Histopathologic Evaluation	0	0	0	0	0	0	0	0	0
Photography	0	0	0	0	0	0	0	0	0
Clinical Evaluation	0	0	0	0	0	0	0	0	0
Laboratory Evaluation	0	0	0	0	0	0	0	0	0
Other	0	0	0	0	0	0	0	0	0

					Cohort 1: Newly Determined Advanced BCC Patients (Non-BCCNS) Locally Advanced BCC (laBCC)				
		Vismo (N=115)			No Vismo (N=251)				
	All (N=433)	All (N=115)	Vismo Only (N=102)	Surgery Only (N=6)	Other Therapy (N=7)	All (N=251)	Surgery Only (N=198)	Other Therapy (N=53)	No Treatment (N=67)
Routine Follow-up 16									
Response Assessment									
n	7	3	3	0	0	4	4	0	0
Complete Response (CR)	6(85.7%)	2(66.7%)	2(66.7%)	0	0	4(100%)	4(100%)	0	0
Partial Response (PR)	0	0	0	0	0	0	0	0	0
Stable Disease (SD)	1(14.3%)	1(33.3%)	1(33.3%)	0	0	0	0	0	0
Disease Progression (PD)	0	0	0	0	0	0	0	0	0
Recurrence	0	0	0	0	0	0	0	0	0
Clinical Deterioration	0	0	0	0	0	0	0	0	0
Unable to Evaluate	0	0	0	0	0	0	0	0	0

If Complete Response, as Determined by[1]									
Negative Histopathology	1(16.7%)	1(50.0%)	1(50.0%)	0	0	0	0	0	0
Complete Tumor Shrinkage	0	0	0	0	0	0	0	0	0
No Clinically Visible Evidence of BCC	6(100%)	2(100%)	2(100%)	0	0	4(100%)	4(100%)	0	0
Other	0	0	0	0	0	0	0	0	0

					Cohort 1: Newly Determined Advanced BCC Patients (Non-BCCNS) Metastatic BCC (mBCC)				
		Vismo (N=2)			No Vismo (N=1)				
	All (N=4)	All (N=2)	Vismo Only (N=2)	Surgery Only (N=0)	Other Therapy (N=0)	All (N=1)	Surgery Only (N=1)	Other Therapy (N=0)	No Treatment (N=1)
Routine Follow-up 16									
Response Assessment									
n	0	0	0	0	0	0	0	0	0
Complete Response (CR)	0	0	0	0	0	0	0	0	0
Partial Response (PR)	0	0	0	0	0	0	0	0	0
Stable Disease (SD)	0	0	0	0	0	0	0	0	0
Disease Progression (PD)	0	0	0	0	0	0	0	0	0
Recurrence	0	0	0	0	0	0	0	0	0
Clinical Deterioration	0	0	0	0	0	0	0	0	0
Unable to Evaluate	0	0	0	0	0	0	0	0	0
If Complete Response, as Determined by[1]									

Negative Histopathology	0	0	0	0	0	0	0	0	0
Complete Tumor Shrinkage	0	0	0	0	0	0	0	0	0
No Clinically Visible Evidence of BCC	0	0	0	0	0	0	0	0	0
Other	0	0	0	0	0	0	0	0	0
					Cohort 1: Newly Determined Advanced BCC Patients (Non-BCCNS) Locally Advanced BCC (laBCC)				
			Vismo (N=115)		No Vismo (N=251)				
	All (N=433)	All (N=115)	Vismo Only (N=102)	Surgery Only (N=6)	Other Therapy (N=7)	All (N=251)	Surgery Only (N=198)	Other Therapy (N=53)	No Treatment (N=67)
Indicate How Response/ Progression was Assessed:									
n	3	3	3	0	0	0	0	0	0
Change in Size of Existing Lesions	2(66.7%)	2(66.7%)	2(66.7%)	0	0	0	0	0	0
Change in Number of Existing Lesions	2(66.7%)	2(66.7%)	2(66.7%)	0	0	0	0	0	0
Development of New BCCs	1(33.3%)	1(33.3%)	1(33.3%)	0	0	0	0	0	0
Progression from Locally Advanced Disease to Metastatic Disease	0	0	0	0	0	0	0	0	0
Development of New Metastases	0	0	0	0	0	0	0	0	0
Other	0	0	0	0	0	0	0	0	0

If progression to metastatic disease or new metastases, indicate site(s)[2]									
Bone	0	0	0	0	0	0	0	0	0
Lymph nodes, Local	0	0	0	0	0	0	0	0	0
Lymph nodes, Distant	0	0	0	0	0	0	0	0	0
Lung	0	0	0	0	0	0	0	0	0
Liver	0	0	0	0	0	0	0	0	0
CNS/Brain	0	0	0	0	0	0	0	0	0
Other	0	0	0	0	0	0	0	0	0
What Methods were Used to Assess Disease Response/ Progression									
n	7	3	3	0	0	4	4	0	0
Diagnostic Image (MRI, CT)	0	0	0	0	0	0	0	0	0
Histopathologic Evaluation	1(14.3%)	1(33.3%)	1(33.3%)	0	0	0	0	0	0
Photography	0	0	0	0	0	0	0	0	0
Clinical Evaluation	6(85.7%)	2(66.7%)	2(66.7%)	0	0	4(100%)	4(100%)	0	0
Laboratory Evaluation	0	0	0	0	0	0	0	0	0
Other	0	0	0	0	0	0	0	0	0
					Cohort 1: Newly Determined Advanced BCC Patients (Non-BCCNS) Metastatic BCC (mBCC)				
			Vismo (N=2)			No Vismo (N=1)			
	All (N=4)	All (N=2)	Vismo Only (N=2)	Surgery Only (N=0)	Other Therapy (N=0)	All (N=1)	Surgery Only (N=1)	Other Therapy (N=0)	No Treatment (N=1)

Indicate How Response/ Progression was Assessed:									
n	0	0	0	0	0	0	0	0	0
Change in Size of Existing Lesions	0	0	0	0	0	0	0	0	0
Change in Number of Existing Lesions	0	0	0	0	0	0	0	0	0
Development of New BCCs	0	0	0	0	0	0	0	0	0
Progression from Locally Advanced Disease to Metastatic Disease	0	0	0	0	0	0	0	0	0
Development of New Metastases	0	0	0	0	0	0	0	0	0
Other	0	0	0	0	0	0	0	0	0
If progression to metastatic disease or new metastases, indicate site(s)[2]									
Bone	0	0	0	0	0	0	0	0	0
Lymph nodes, Local	0	0	0	0	0	0	0	0	0
Lymph nodes, Distant	0	0	0	0	0	0	0	0	0
Lung	0	0	0	0	0	0	0	0	0
Liver	0	0	0	0	0	0	0	0	0
CNS/Brain	0	0	0	0	0	0	0	0	0
Other	0	0	0	0	0	0	0	0	0

What Methods were Used to Assess Disease Response/ Progression									
n	0	0	0	0	0	0	0	0	0
Diagnostic Image (MRI, CT)	0	0	0	0	0	0	0	0	0
Histopathologic Evaluation	0	0	0	0	0	0	0	0	0
Photography	0	0	0	0	0	0	0	0	0
Clinical Evaluation	0	0	0	0	0	0	0	0	0
Laboratory Evaluation	0	0	0	0	0	0	0	0	0
Other	0	0	0	0	0	0	0	0	0
					Cohort 1: Newly Determined Advanced BCC Patients (Non-BCCNS) Locally Advanced BCC (laBCC)				
		Vismo (N=115)			No Vismo (N=251)				
	All (N=433)	All (N=115)	Vismo Only (N=102)	Surgery Only (N=6)	Other Therapy (N=7)	All (N=251)	Surgery Only (N=198)	Other Therapy (N=53)	No Treatment (N=67)
Routine Follow-up 17									
Response Assessment									
n	7	3	3	0	0	4	4	0	0
Complete Response (CR)	5(71.4%)	1(33.3%)	1(33.3%)	0	0	4(100%)	4(100%)	0	0
Partial Response (PR)	0	0	0	0	0	0	0	0	0
Stable Disease (SD)	1(14.3%)	1(33.3%)	1(33.3%)	0	0	0	0	0	0
Disease Progression (PD)	0	0	0	0	0	0	0	0	0
Recurrence	1(14.3%)	1(33.3%)	1(33.3%)	0	0	0	0	0	0
Clinical Deterioration	0	0	0	0	0	0	0	0	0
Unable to Evaluate	0	0	0	0	0	0	0	0	0

If Complete Response, as Determined by[1]									
Negative Histopathology	0	0	0	0	0	0	0	0	0
Complete Tumor Shrinkage	0	0	0	0	0	0	0	0	0
No Clinically Visible Evidence of BCC	5(100%)	1(100%)	1(100%)	0	0	4(100%)	4(100%)	0	0
Other	0	0	0	0	0	0	0	0	0
					Cohort 1: Newly Determined Advanced BCC Patients (Non-BCCNS) Metastatic BCC (mBCC)				
			Vismo (N=2)			No Vismo (N=1)			
	All (N=4)	All (N=2)	Vismo Only (N=2)	Surgery Only (N=0)	Other Therapy (N=0)	All (N=1)	Surgery Only (N=1)	Other Therapy (N=0)	No Treatment (N=1)
Routine Follow-up 17									
Response Assessment									
n	0	0	0	0	0	0	0	0	0
Complete Response (CR)	0	0	0	0	0	0	0	0	0
Partial Response (PR)	0	0	0	0	0	0	0	0	0
Stable Disease (SD)	0	0	0	0	0	0	0	0	0
Disease Progression (PD)	0	0	0	0	0	0	0	0	0
Recurrence	0	0	0	0	0	0	0	0	0
Clinical Deterioration	0	0	0	0	0	0	0	0	0
Unable to Evaluate	0	0	0	0	0	0	0	0	0
If Complete Response, as Determined by[1]									

Negative Histopathology	0	0	0	0	0	0	0	0	0
Complete Tumor Shrinkage	0	0	0	0	0	0	0	0	0
No Clinically Visible Evidence of BCC	0	0	0	0	0	0	0	0	0
Other	0	0	0	0	0	0	0	0	0
					Cohort 1: Newly Determined Advanced BCC Patients (Non-BCCNS) Locally Advanced BCC (laBCC)				
			Vismo (N=115)		No Vismo (N=251)				
	All (N=433)	All (N=115)	Vismo Only (N=102)	Surgery Only (N=6)	Other Therapy (N=7)	All (N=251)	Surgery Only (N=198)	Other Therapy (N=53)	No Treatment (N=67)
Indicate How Response/ Progression was Assessed:									
n	3	3	3	0	0	0	0	0	0
Change in Size of Existing Lesions	1(33.3%)	1(33.3%)	1(33.3%)	0	0	0	0	0	0
Change in Number of Existing Lesions	3(100%)	3(100%)	3(100%)	0	0	0	0	0	0
Development of New BCCs	0	0	0	0	0	0	0	0	0
Progression from Locally Advanced Disease to Metastatic Disease	0	0	0	0	0	0	0	0	0
Development of New Metastases	0	0	0	0	0	0	0	0	0
Other	1(33.3%)	1(33.3%)	1(33.3%)	0	0	0	0	0	0

					Cohort 1: Newly Determined Advanced BCC Patients (Non-BCCNS) Metastatic BCC (mBCC)				
		Vismo (N=2)			No Vismo (N=1)				
	All (N=4)	All (N=2)	Vismo Only (N=2)	Surgery Only (N=0)	Other Therapy (N=0)	All (N=1)	Surgery Only (N=1)	Other Therapy (N=0)	No Treatment (N=1)
If progression to metastatic disease or new metastases, indicate site(s)[2]									
Bone	0	0	0	0	0	0	0	0	0
Lymph nodes, Local	0	0	0	0	0	0	0	0	0
Lymph nodes, Distant	0	0	0	0	0	0	0	0	0
Lung	0	0	0	0	0	0	0	0	0
Liver	0	0	0	0	0	0	0	0	0
CNS/Brain	0	0	0	0	0	0	0	0	0
Other	0	0	0	0	0	0	0	0	0
What Methods were Used to Assess Disease Response/ Progression									
n	7	3	3	0	0	4	4	0	0
Diagnostic Image (MRI, CT)	0	0	0	0	0	0	0	0	0
Histopathologic Evaluation	2(28.6%)	2(66.7%)	2(66.7%)	0	0	0	0	0	0
Photography	1(14.3%)	1(33.3%)	1(33.3%)	0	0	0	0	0	0
Clinical Evaluation	7(100%)	3(100%)	3(100%)	0	0	4(100%)	4(100%)	0	0
Laboratory Evaluation	0	0	0	0	0	0	0	0	0
Other	0	0	0	0	0	0	0	0	0

Indicate How Response/ Progression was Assessed:									
n	0	0	0	0	0	0	0	0	0
Change in Size of Existing Lesions	0	0	0	0	0	0	0	0	0
Change in Number of Existing Lesions	0	0	0	0	0	0	0	0	0
Development of New BCCs	0	0	0	0	0	0	0	0	0
Progression from Locally Advanced Disease to Metastatic Disease	0	0	0	0	0	0	0	0	0
Development of New Metastases	0	0	0	0	0	0	0	0	0
Other	0	0	0	0	0	0	0	0	0
If progression to metastatic disease or new metastases, indicate site(s)[2]									
Bone	0	0	0	0	0	0	0	0	0
Lymph nodes, Local	0	0	0	0	0	0	0	0	0
Lymph nodes, Distant	0	0	0	0	0	0	0	0	0
Lung	0	0	0	0	0	0	0	0	0
Liver	0	0	0	0	0	0	0	0	0
CNS/Brain	0	0	0	0	0	0	0	0	0
Other	0	0	0	0	0	0	0	0	0

What Methods were Used to Assess Disease Response/ Progression									
n	0	0	0	0	0	0	0	0	0
Diagnostic Image (MRI, CT)	0	0	0	0	0	0	0	0	0
Histopathologic Evaluation	0	0	0	0	0	0	0	0	0
Photography	0	0	0	0	0	0	0	0	0
Clinical Evaluation	0	0	0	0	0	0	0	0	0
Laboratory Evaluation	0	0	0	0	0	0	0	0	0
Other	0	0	0	0	0	0	0	0	0

					Cohort 1: Newly Determined Advanced BCC Patients (Non-BCCNS) Locally Advanced BCC (laBCC)				
		Vismo (N=115)			No Vismo (N=251)				
	All (N=433)	All (N=115)	Vismo Only (N=102)	Surgery Only (N=6)	Other Therapy (N=7)	All (N=251)	Surgery Only (N=198)	Other Therapy (N=53)	No Treatment (N=67)
Routine Follow-up 18									
Response Assessment									
n	6	3	3	0	0	3	3	0	0
Complete Response (CR)	5(83.3%)	2(66.7%)	2(66.7%)	0	0	3(100%)	3(100%)	0	0
Partial Response (PR)	0	0	0	0	0	0	0	0	0
Stable Disease (SD)	1(16.7%)	1(33.3%)	1(33.3%)	0	0	0	0	0	0
Disease Progression (PD)	0	0	0	0	0	0	0	0	0
Recurrence	0	0	0	0	0	0	0	0	0
Clinical Deterioration	0	0	0	0	0	0	0	0	0
Unable to Evaluate	0	0	0	0	0	0	0	0	0

If Complete Response, as Determined by[1]									
Negative Histopathology	0	0	0	0	0	0	0	0	0
Complete Tumor Shrinkage	0	0	0	0	0	0	0	0	0
No Clinically Visible Evidence of BCC	5(100%)	2(100%)	2(100%)	0	0	3(100%)	3(100%)	0	0
Other	0	0	0	0	0	0	0	0	0

					Cohort 1: Newly Determined Advanced BCC Patients (Non-BCCNS) Metastatic BCC (mBCC)				
		Vismo (N=2)			No Vismo (N=1)				
	All (N=4)	All (N=2)	Vismo Only (N=2)	Surgery Only (N=0)	Other Therapy (N=0)	All (N=1)	Surgery Only (N=1)	Other Therapy (N=0)	No Treatment (N=1)
Routine Follow-up 18									
Response Assessment									
n	0	0	0	0	0	0	0	0	0
Complete Response (CR)	0	0	0	0	0	0	0	0	0
Partial Response (PR)	0	0	0	0	0	0	0	0	0
Stable Disease (SD)	0	0	0	0	0	0	0	0	0
Disease Progression (PD)	0	0	0	0	0	0	0	0	0
Recurrence	0	0	0	0	0	0	0	0	0
Clinical Deterioration	0	0	0	0	0	0	0	0	0
Unable to Evaluate	0	0	0	0	0	0	0	0	0
If Complete Response, as Determined by[1]									

Negative Histopathology	0	0	0	0	0	0	0	0	0
Complete Tumor Shrinkage	0	0	0	0	0	0	0	0	0
No Clinically Visible Evidence of BCC	0	0	0	0	0	0	0	0	0
Other	0	0	0	0	0	0	0	0	0

					Cohort 1: Newly Determined Advanced BCC Patients (Non-BCCNS) Locally Advanced BCC (laBCC)				
		Vismo (N=115)			No Vismo (N=251)				
	All (N=433)	All (N=115)	Vismo Only (N=102)	Surgery Only (N=6)	Other Therapy (N=7)	All (N=251)	Surgery Only (N=198)	Other Therapy (N=53)	No Treatment (N=67)
Indicate How Response/ Progression was Assessed:									
n	3	3	3	0	0	0	0	0	0
Change in Size of Existing Lesions	1(33.3%)	1(33.3%)	1(33.3%)	0	0	0	0	0	0
Change in Number of Existing Lesions	2(66.7%)	2(66.7%)	2(66.7%)	0	0	0	0	0	0
Development of New BCCs	1(33.3%)	1(33.3%)	1(33.3%)	0	0	0	0	0	0
Progression from Locally Advanced Disease to Metastatic Disease	0	0	0	0	0	0	0	0	0
Development of New Metastases	0	0	0	0	0	0	0	0	0
Other	0	0	0	0	0	0	0	0	0

If progression to metastatic disease or new metastases, indicate site(s)[2]									
Bone	0	0	0	0	0	0	0	0	0
Lymph nodes, Local	0	0	0	0	0	0	0	0	0
Lymph nodes, Distant	0	0	0	0	0	0	0	0	0
Lung	0	0	0	0	0	0	0	0	0
Liver	0	0	0	0	0	0	0	0	0
CNS/Brain	0	0	0	0	0	0	0	0	0
Other	0	0	0	0	0	0	0	0	0
What Methods were Used to Assess Disease Response/ Progression									
n	6	3	3	0	0	3	3	0	0
Diagnostic Image (MRI, CT)	0	0	0	0	0	0	0	0	0
Histopathologic Evaluation	0	0	0	0	0	0	0	0	0
Photography	1(16.7%)	1(33.3%)	1(33.3%)	0	0	0	0	0	0
Clinical Evaluation	6(100%)	3(100%)	3(100%)	0	0	3(100%)	3(100%)	0	0
Laboratory Evaluation	0	0	0	0	0	0	0	0	0
Other	0	0	0	0	0	0	0	0	0
					Cohort 1: Newly Determined Advanced BCC Patients (Non-BCCNS) Metastatic BCC (mBCC)				
		Vismo (N=2)			No Vismo (N=1)				
	All (N=4)	All (N=2)	Vismo Only (N=2)	Surgery Only (N=0)	Other Therapy (N=0)	All (N=1)	Surgery Only (N=1)	Other Therapy (N=0)	No Treatment (N=1)

Indicate How Response/ Progression was Assessed:									
n	0	0	0	0	0	0	0	0	0
Change in Size of Existing Lesions	0	0	0	0	0	0	0	0	0
Change in Number of Existing Lesions	0	0	0	0	0	0	0	0	0
Development of New BCCs	0	0	0	0	0	0	0	0	0
Progression from Locally Advanced Disease to Metastatic Disease	0	0	0	0	0	0	0	0	0
Development of New Metastases	0	0	0	0	0	0	0	0	0
Other	0	0	0	0	0	0	0	0	0
If progression to metastatic disease or new metastases, indicate site(s)[2]									
Bone	0	0	0	0	0	0	0	0	0
Lymph nodes, Local	0	0	0	0	0	0	0	0	0
Lymph nodes, Distant	0	0	0	0	0	0	0	0	0
Lung	0	0	0	0	0	0	0	0	0
Liver	0	0	0	0	0	0	0	0	0
CNS/Brain	0	0	0	0	0	0	0	0	0
Other	0	0	0	0	0	0	0	0	0

What Methods were Used to Assess Disease Response/ Progression									
n	0	0	0	0	0	0	0	0	0
Diagnostic Image (MRI, CT)	0	0	0	0	0	0	0	0	0
Histopathologic Evaluation	0	0	0	0	0	0	0	0	0
Photography	0	0	0	0	0	0	0	0	0
Clinical Evaluation	0	0	0	0	0	0	0	0	0
Laboratory Evaluation	0	0	0	0	0	0	0	0	0
Other	0	0	0	0	0	0	0	0	0

					Cohort 1: Newly Determined Advanced BCC Patients (Non-BCCNS) Locally Advanced BCC (laBCC)				
		Vismo (N=115)			No Vismo (N=251)				
	All (N=433)	All (N=115)	Vismo Only (N=102)	Surgery Only (N=6)	Other Therapy (N=7)	All (N=251)	Surgery Only (N=198)	Other Therapy (N=53)	No Treatment (N=67)
Routine Follow-up 19									
Response Assessment									
n	4	2	2	0	0	2	2	0	0
Complete Response (CR)	3(75.0%)	1(50.0%)	1(50.0%)	0	0	2(100%)	2(100%)	0	0
Partial Response (PR)	0	0	0	0	0	0	0	0	0
Stable Disease (SD)	1(25.0%)	1(50.0%)	1(50.0%)	0	0	0	0	0	0
Disease Progression (PD)	0	0	0	0	0	0	0	0	0
Recurrence	0	0	0	0	0	0	0	0	0
Clinical Deterioration	0	0	0	0	0	0	0	0	0
Unable to Evaluate	0	0	0	0	0	0	0	0	0

If Complete Response, as Determined by[1]									
Negative Histopathology	0	0	0	0	0	0	0	0	0
Complete Tumor Shrinkage	0	0	0	0	0	0	0	0	0
No Clinically Visible Evidence of BCC	3(100%)	1(100%)	1(100%)	0	0	2(100%)	2(100%)	0	0
Other	0	0	0	0	0	0	0	0	0

					Cohort 1: Newly Determined Advanced BCC Patients (Non-BCCNS) Metastatic BCC (mBCC)				
		Vismo (N=2)			No Vismo (N=1)				
	All (N=4)	All (N=2)	Vismo Only (N=2)	Surgery Only (N=0)	Other Therapy (N=0)	All (N=1)	Surgery Only (N=1)	Other Therapy (N=0)	No Treatment (N=1)
Routine Follow-up 19									
Response Assessment									
n	0	0	0	0	0	0	0	0	0
Complete Response (CR)	0	0	0	0	0	0	0	0	0
Partial Response (PR)	0	0	0	0	0	0	0	0	0
Stable Disease (SD)	0	0	0	0	0	0	0	0	0
Disease Progression (PD)	0	0	0	0	0	0	0	0	0
Recurrence	0	0	0	0	0	0	0	0	0
Clinical Deterioration	0	0	0	0	0	0	0	0	0
Unable to Evaluate	0	0	0	0	0	0	0	0	0
If Complete Response, as Determined by[1]									

Negative Histopathology	0	0	0	0	0	0	0	0	0
Complete Tumor Shrinkage	0	0	0	0	0	0	0	0	0
No Clinically Visible Evidence of BCC	0	0	0	0	0	0	0	0	0
Other	0	0	0	0	0	0	0	0	0
					Cohort 1: Newly Determined Advanced BCC Patients (Non-BCCNS) Locally Advanced BCC (laBCC)				
			Vismo (N=115)		No Vismo (N=251)				
	All (N=433)	All (N=115)	Vismo Only (N=102)	Surgery Only (N=6)	Other Therapy (N=7)	All (N=251)	Surgery Only (N=198)	Other Therapy (N=53)	No Treatment (N=67)
Indicate How Response/ Progression was Assessed:									
n	2	2	2	0	0	0	0	0	0
Change in Size of Existing Lesions	2(100%)	2(100%)	2(100%)	0	0	0	0	0	0
Change in Number of Existing Lesions	1(50.0%)	1(50.0%)	1(50.0%)	0	0	0	0	0	0
Development of New BCCs	0	0	0	0	0	0	0	0	0
Progression from Locally Advanced Disease to Metastatic Disease	0	0	0	0	0	0	0	0	0
Development of New Metastases	0	0	0	0	0	0	0	0	0
Other	0	0	0	0	0	0	0	0	0

If progression to metastatic disease or new metastases, indicate site(s)[2]									
Bone	0	0	0	0	0	0	0	0	0
Lymph nodes, Local	0	0	0	0	0	0	0	0	0
Lymph nodes, Distant	0	0	0	0	0	0	0	0	0
Lung	0	0	0	0	0	0	0	0	0
Liver	0	0	0	0	0	0	0	0	0
CNS/Brain	0	0	0	0	0	0	0	0	0
Other	0	0	0	0	0	0	0	0	0
What Methods were Used to Assess Disease Response/ Progression									
n	4	2	2	0	0	2	2	0	0
Diagnostic Image (MRI, CT)	0	0	0	0	0	0	0	0	0
Histopathologic Evaluation	1(25.0%)	1(50.0%)	1(50.0%)	0	0	0	0	0	0
Photography	0	0	0	0	0	0	0	0	0
Clinical Evaluation	4(100%)	2(100%)	2(100%)	0	0	2(100%)	2(100%)	0	0
Laboratory Evaluation	0	0	0	0	0	0	0	0	0
Other	0	0	0	0	0	0	0	0	0
					Cohort 1: Newly Determined Advanced BCC Patients (Non-BCCNS) Metastatic BCC (mBCC)				
		Vismo (N=2)			No Vismo (N=1)				
	All (N=4)	All (N=2)	Vismo Only (N=2)	Surgery Only (N=0)	Other Therapy (N=0)	All (N=1)	Surgery Only (N=1)	Other Therapy (N=0)	No Treatment (N=1)

Indicate How Response/ Progression was Assessed:									
n	0	0	0	0	0	0	0	0	0
Change in Size of Existing Lesions	0	0	0	0	0	0	0	0	0
Change in Number of Existing Lesions	0	0	0	0	0	0	0	0	0
Development of New BCCs	0	0	0	0	0	0	0	0	0
Progression from Locally Advanced Disease to Metastatic Disease	0	0	0	0	0	0	0	0	0
Development of New Metastases	0	0	0	0	0	0	0	0	0
Other	0	0	0	0	0	0	0	0	0
If progression to metastatic disease or new metastases, indicate site(s)[2]									
Bone	0	0	0	0	0	0	0	0	0
Lymph nodes, Local	0	0	0	0	0	0	0	0	0
Lymph nodes, Distant	0	0	0	0	0	0	0	0	0
Lung	0	0	0	0	0	0	0	0	0
Liver	0	0	0	0	0	0	0	0	0
CNS/Brain	0	0	0	0	0	0	0	0	0
Other	0	0	0	0	0	0	0	0	0

What Methods were Used to Assess Disease Response/ Progression									
n	0	0	0	0	0	0	0	0	0
Diagnostic Image (MRI, CT)	0	0	0	0	0	0	0	0	0
Histopathologic Evaluation	0	0	0	0	0	0	0	0	0
Photography	0	0	0	0	0	0	0	0	0
Clinical Evaluation	0	0	0	0	0	0	0	0	0
Laboratory Evaluation	0	0	0	0	0	0	0	0	0
Other	0	0	0	0	0	0	0	0	0
					Cohort 1: Newly Determined Advanced BCC Patients (Non-BCCNS) Locally Advanced BCC (laBCC)				
		Vismo (N=115)			No Vismo (N=251)				
	All (N=433)	All (N=115)	Vismo Only (N=102)	Surgery Only (N=6)	Other Therapy (N=7)	All (N=251)	Surgery Only (N=198)	Other Therapy (N=53)	No Treatment (N=67)
Routine Follow-up 20									
Response Assessment									
n	4	2	2	0	0	2	2	0	0
Complete Response (CR)	3(75.0%)	1(50.0%)	1(50.0%)	0	0	2(100%)	2(100%)	0	0
Partial Response (PR)	0	0	0	0	0	0	0	0	0
Stable Disease (SD)	1(25.0%)	1(50.0%)	1(50.0%)	0	0	0	0	0	0
Disease Progression (PD)	0	0	0	0	0	0	0	0	0
Recurrence	0	0	0	0	0	0	0	0	0
Clinical Deterioration	0	0	0	0	0	0	0	0	0
Unable to Evaluate	0	0	0	0	0	0	0	0	0

If Complete Response, as Determined by[1]									
Negative Histopathology	0	0	0	0	0	0	0	0	0
Complete Tumor Shrinkage	0	0	0	0	0	0	0	0	0
No Clinically Visible Evidence of BCC	3(100%)	1(100%)	1(100%)	0	0	2(100%)	2(100%)	0	0
Other	0	0	0	0	0	0	0	0	0

					Cohort 1: Newly Determined Advanced BCC Patients (Non-BCCNS) Metastatic BCC (mBCC)				
		Vismo (N=2)			No Vismo (N=1)				
	All (N=4)	All (N=2)	Vismo Only (N=2)	Surgery Only (N=0)	Other Therapy (N=0)	All (N=1)	Surgery Only (N=1)	Other Therapy (N=0)	No Treatment (N=1)
Routine Follow-up 20									
Response Assessment									
n	0	0	0	0	0	0	0	0	0
Complete Response (CR)	0	0	0	0	0	0	0	0	0
Partial Response (PR)	0	0	0	0	0	0	0	0	0
Stable Disease (SD)	0	0	0	0	0	0	0	0	0
Disease Progression (PD)	0	0	0	0	0	0	0	0	0
Recurrence	0	0	0	0	0	0	0	0	0
Clinical Deterioration	0	0	0	0	0	0	0	0	0
Unable to Evaluate	0	0	0	0	0	0	0	0	0
If Complete Response, as Determined by[1]									

Negative Histopathology	0	0	0	0	0	0	0	0	0
Complete Tumor Shrinkage	0	0	0	0	0	0	0	0	0
No Clinically Visible Evidence of BCC	0	0	0	0	0	0	0	0	0
Other	0	0	0	0	0	0	0	0	0

					Cohort 1: Newly Determined Advanced BCC Patients (Non-BCCNS) Locally Advanced BCC (laBCC)				
			Vismo (N=115)		No Vismo (N=251)				
	All (N=433)	All (N=115)	Vismo Only (N=102)	Surgery Only (N=6)	Other Therapy (N=7)	All (N=251)	Surgery Only (N=198)	Other Therapy (N=53)	No Treatment (N=67)
Indicate How Response/ Progression was Assessed:									
n	2	2	2	0	0	0	0	0	0
Change in Size of Existing Lesions	1(50.0%)	1(50.0%)	1(50.0%)	0	0	0	0	0	0
Change in Number of Existing Lesions	2(100%)	2(100%)	2(100%)	0	0	0	0	0	0
Development of New BCCs	0	0	0	0	0	0	0	0	0
Progression from Locally Advanced Disease to Metastatic Disease	0	0	0	0	0	0	0	0	0
Development of New Metastases	0	0	0	0	0	0	0	0	0
Other	0	0	0	0	0	0	0	0	0

If progression to metastatic disease or new metastases, indicate site(s)[2]									
Bone	0	0	0	0	0	0	0	0	0
Lymph nodes, Local	0	0	0	0	0	0	0	0	0
Lymph nodes, Distant	0	0	0	0	0	0	0	0	0
Lung	0	0	0	0	0	0	0	0	0
Liver	0	0	0	0	0	0	0	0	0
CNS/Brain	0	0	0	0	0	0	0	0	0
Other	0	0	0	0	0	0	0	0	0
What Methods were Used to Assess Disease Response/ Progression									
n	4	2	2	0	0	2	2	0	0
Diagnostic Image (MRI, CT)	0	0	0	0	0	0	0	0	0
Histopathologic Evaluation	0	0	0	0	0	0	0	0	0
Photography	0	0	0	0	0	0	0	0	0
Clinical Evaluation	4(100%)	2(100%)	2(100%)	0	0	2(100%)	2(100%)	0	0
Laboratory Evaluation	0	0	0	0	0	0	0	0	0
Other	0	0	0	0	0	0	0	0	0
					Cohort 1: Newly Determined Advanced BCC Patients (Non-BCCNS) Metastatic BCC (mBCC)				
			Vismo (N=2)			No Vismo (N=1)			
	All (N=4)	All (N=2)	Vismo Only (N=2)	Surgery Only (N=0)	Other Therapy (N=0)	All (N=1)	Surgery Only (N=1)	Other Therapy (N=0)	No Treatment (N=1)

Indicate How Response/ Progression was Assessed:									
n	0	0	0	0	0	0	0	0	0
Change in Size of Existing Lesions	0	0	0	0	0	0	0	0	0
Change in Number of Existing Lesions	0	0	0	0	0	0	0	0	0
Development of New BCCs	0	0	0	0	0	0	0	0	0
Progression from Locally Advanced Disease to Metastatic Disease	0	0	0	0	0	0	0	0	0
Development of New Metastases	0	0	0	0	0	0	0	0	0
Other	0	0	0	0	0	0	0	0	0
If progression to metastatic disease or new metastases, indicate site(s)[2]									
Bone	0	0	0	0	0	0	0	0	0
Lymph nodes, Local	0	0	0	0	0	0	0	0	0
Lymph nodes, Distant	0	0	0	0	0	0	0	0	0
Lung	0	0	0	0	0	0	0	0	0
Liver	0	0	0	0	0	0	0	0	0
CNS/Brain	0	0	0	0	0	0	0	0	0
Other	0	0	0	0	0	0	0	0	0

What Methods were Used to Assess Disease Response/ Progression									
n	0	0	0	0	0	0	0	0	0
Diagnostic Image (MRI, CT)	0	0	0	0	0	0	0	0	0
Histopathologic Evaluation	0	0	0	0	0	0	0	0	0
Photography	0	0	0	0	0	0	0	0	0
Clinical Evaluation	0	0	0	0	0	0	0	0	0
Laboratory Evaluation	0	0	0	0	0	0	0	0	0
Other	0	0	0	0	0	0	0	0	0

					Cohort 1: Newly Determined Advanced BCC Patients (Non-BCCNS) Locally Advanced BCC (laBCC)				
		Vismo (N=115)			No Vismo (N=251)				
	All (N=433)	All (N=115)	Vismo Only (N=102)	Surgery Only (N=6)	Other Therapy (N=7)	All (N=251)	Surgery Only (N=198)	Other Therapy (N=53)	No Treatment (N=67)
Routine Follow-up 21									
Response Assessment									
n	4	2	2	0	0	2	2	0	0
Complete Response (CR)	3(75.0%)	1(50.0%)	1(50.0%)	0	0	2(100%)	2(100%)	0	0
Partial Response (PR)	0	0	0	0	0	0	0	0	0
Stable Disease (SD)	1(25.0%)	1(50.0%)	1(50.0%)	0	0	0	0	0	0
Disease Progression (PD)	0	0	0	0	0	0	0	0	0
Recurrence	0	0	0	0	0	0	0	0	0
Clinical Deterioration	0	0	0	0	0	0	0	0	0
Unable to Evaluate	0	0	0	0	0	0	0	0	0

If Complete Response, as Determined by[1]									
Negative Histopathology	0	0	0	0	0	0	0	0	0
Complete Tumor Shrinkage	0	0	0	0	0	0	0	0	0
No Clinically Visible Evidence of BCC	3(100%)	1(100%)	1(100%)	0	0	2(100%)	2(100%)	0	0
Other	0	0	0	0	0	0	0	0	0

					Cohort 1: Newly Determined Advanced BCC Patients (Non-BCCNS) Metastatic BCC (mBCC)				
		Vismo (N=2)			No Vismo (N=1)				
	All (N=4)	All (N=2)	Vismo Only (N=2)	Surgery Only (N=0)	Other Therapy (N=0)	All (N=1)	Surgery Only (N=1)	Other Therapy (N=0)	No Treatment (N=1)
Routine Follow-up 21									
Response Assessment									
n	0	0	0	0	0	0	0	0	0
Complete Response (CR)	0	0	0	0	0	0	0	0	0
Partial Response (PR)	0	0	0	0	0	0	0	0	0
Stable Disease (SD)	0	0	0	0	0	0	0	0	0
Disease Progression (PD)	0	0	0	0	0	0	0	0	0
Recurrence	0	0	0	0	0	0	0	0	0
Clinical Deterioration	0	0	0	0	0	0	0	0	0
Unable to Evaluate	0	0	0	0	0	0	0	0	0
If Complete Response, as Determined by[1]									

Negative Histopathology	0	0	0	0	0	0	0	0	0
Complete Tumor Shrinkage	0	0	0	0	0	0	0	0	0
No Clinically Visible Evidence of BCC	0	0	0	0	0	0	0	0	0
Other	0	0	0	0	0	0	0	0	0

					Cohort 1: Newly Determined Advanced BCC Patients (Non-BCCNS) Locally Advanced BCC (laBCC)				
			Vismo (N=115)			No Vismo (N=251)			
	All (N=433)	All (N=115)	Vismo Only (N=102)	Surgery Only (N=6)	Other Therapy (N=7)	All (N=251)	Surgery Only (N=198)	Other Therapy (N=53)	No Treatment (N=67)
Indicate How Response/ Progression was Assessed:									
n	1	1	1	0	0	0	0	0	0
Change in Size of Existing Lesions	1(100%)	1(100%)	1(100%)	0	0	0	0	0	0
Change in Number of Existing Lesions	0	0	0	0	0	0	0	0	0
Development of New BCCs	0	0	0	0	0	0	0	0	0
Progression from Locally Advanced Disease to Metastatic Disease	0	0	0	0	0	0	0	0	0
Development of New Metastases	0	0	0	0	0	0	0	0	0
Other	0	0	0	0	0	0	0	0	0

If progression to metastatic disease or new metastases, indicate site(s)[2]									
Bone	0	0	0	0	0	0	0	0	0
Lymph nodes, Local	0	0	0	0	0	0	0	0	0
Lymph nodes, Distant	0	0	0	0	0	0	0	0	0
Lung	0	0	0	0	0	0	0	0	0
Liver	0	0	0	0	0	0	0	0	0
CNS/Brain	0	0	0	0	0	0	0	0	0
Other	0	0	0	0	0	0	0	0	0
What Methods were Used to Assess Disease Response/ Progression									
n	4	2	2	0	0	2	2	0	0
Diagnostic Image (MRI, CT)	0	0	0	0	0	0	0	0	0
Histopathologic Evaluation	1(25.0%)	1(50.0%)	1(50.0%)	0	0	0	0	0	0
Photography	0	0	0	0	0	0	0	0	0
Clinical Evaluation	4(100%)	2(100%)	2(100%)	0	0	2(100%)	2(100%)	0	0
Laboratory Evaluation	0	0	0	0	0	0	0	0	0
Other	0	0	0	0	0	0	0	0	0
					Cohort 1: Newly Determined Advanced BCC Patients (Non-BCCNS) Metastatic BCC (mBCC)				
			Vismo (N=2)			No Vismo (N=1)			
	All (N=4)	All (N=2)	Vismo Only (N=2)	Surgery Only (N=0)	Other Therapy (N=0)	All (N=1)	Surgery Only (N=1)	Other Therapy (N=0)	No Treatment (N=1)

Indicate How Response/ Progression was Assessed:									
n	0	0	0	0	0	0	0	0	0
Change in Size of Existing Lesions	0	0	0	0	0	0	0	0	0
Change in Number of Existing Lesions	0	0	0	0	0	0	0	0	0
Development of New BCCs	0	0	0	0	0	0	0	0	0
Progression from Locally Advanced Disease to Metastatic Disease	0	0	0	0	0	0	0	0	0
Development of New Metastases	0	0	0	0	0	0	0	0	0
Other	0	0	0	0	0	0	0	0	0
If progression to metastatic disease or new metastases, indicate site(s)[2]									
Bone	0	0	0	0	0	0	0	0	0
Lymph nodes, Local	0	0	0	0	0	0	0	0	0
Lymph nodes, Distant	0	0	0	0	0	0	0	0	0
Lung	0	0	0	0	0	0	0	0	0
Liver	0	0	0	0	0	0	0	0	0
CNS/Brain	0	0	0	0	0	0	0	0	0
Other	0	0	0	0	0	0	0	0	0

What Methods were Used to Assess Disease Response/ Progression									
n	0	0	0	0	0	0	0	0	0
Diagnostic Image (MRI, CT)	0	0	0	0	0	0	0	0	0
Histopathologic Evaluation	0	0	0	0	0	0	0	0	0
Photography	0	0	0	0	0	0	0	0	0
Clinical Evaluation	0	0	0	0	0	0	0	0	0
Laboratory Evaluation	0	0	0	0	0	0	0	0	0
Other	0	0	0	0	0	0	0	0	0

					Cohort 1: Newly Determined Advanced BCC Patients (Non-BCCNS) Locally Advanced BCC (laBCC)				
		Vismo (N=115)			No Vismo (N=251)				
	All (N=433)	All (N=115)	Vismo Only (N=102)	Surgery Only (N=6)	Other Therapy (N=7)	All (N=251)	Surgery Only (N=198)	Other Therapy (N=53)	No Treatment (N=67)
Routine Follow-up 22									
Response Assessment									
n	4	2	2	0	0	2	2	0	0
Complete Response (CR)	3(75.0%)	1(50.0%)	1(50.0%)	0	0	2(100%)	2(100%)	0	0
Partial Response (PR)	0	0	0	0	0	0	0	0	0
Stable Disease (SD)	1(25.0%)	1(50.0%)	1(50.0%)	0	0	0	0	0	0
Disease Progression (PD)	0	0	0	0	0	0	0	0	0
Recurrence	0	0	0	0	0	0	0	0	0
Clinical Deterioration	0	0	0	0	0	0	0	0	0
Unable to Evaluate	0	0	0	0	0	0	0	0	0

If Complete Response, as Determined by[1]									
Negative Histopathology	0	0	0	0	0	0	0	0	0
Complete Tumor Shrinkage	0	0	0	0	0	0	0	0	0
No Clinically Visible Evidence of BCC	3(100%)	1(100%)	1(100%)	0	0	2(100%)	2(100%)	0	0
Other	0	0	0	0	0	0	0	0	0

					Cohort 1: Newly Determined Advanced BCC Patients (Non-BCCNS) Metastatic BCC (mBCC)				
			Vismo (N=2)			No Vismo (N=1)			
	All (N=4)	All (N=2)	Vismo Only (N=2)	Surgery Only (N=0)	Other Therapy (N=0)	All (N=1)	Surgery Only (N=1)	Other Therapy (N=0)	No Treatment (N=1)
Routine Follow-up 22									
Response Assessment									
n	0	0	0	0	0	0	0	0	0
Complete Response (CR)	0	0	0	0	0	0	0	0	0
Partial Response (PR)	0	0	0	0	0	0	0	0	0
Stable Disease (SD)	0	0	0	0	0	0	0	0	0
Disease Progression (PD)	0	0	0	0	0	0	0	0	0
Recurrence	0	0	0	0	0	0	0	0	0
Clinical Deterioration	0	0	0	0	0	0	0	0	0
Unable to Evaluate	0	0	0	0	0	0	0	0	0
If Complete Response, as Determined by[1]									

Negative Histopathology	0	0	0	0	0	0	0	0	0
Complete Tumor Shrinkage	0	0	0	0	0	0	0	0	0
No Clinically Visible Evidence of BCC	0	0	0	0	0	0	0	0	0
Other	0	0	0	0	0	0	0	0	0
					Cohort 1: Newly Determined Advanced BCC Patients (Non-BCCNS) Locally Advanced BCC (laBCC)				
			Vismo (N=115)			No Vismo (N=251)			
	All (N=433)	All (N=115)	Vismo Only (N=102)	Surgery Only (N=6)	Other Therapy (N=7)	All (N=251)	Surgery Only (N=198)	Other Therapy (N=53)	No Treatment (N=67)
Indicate How Response/ Progression was Assessed:									
n	2	2	2	0	0	0	0	0	0
Change in Size of Existing Lesions	2(100%)	2(100%)	2(100%)	0	0	0	0	0	0
Change in Number of Existing Lesions	1(50.0%)	1(50.0%)	1(50.0%)	0	0	0	0	0	0
Development of New BCCs	0	0	0	0	0	0	0	0	0
Progression from Locally Advanced Disease to Metastatic Disease	0	0	0	0	0	0	0	0	0
Development of New Metastases	0	0	0	0	0	0	0	0	0
Other	0	0	0	0	0	0	0	0	0

					Cohort 1: Newly Determined Advanced BCC Patients (Non-BCCNS) Metastatic BCC (mBCC)				
			Vismo (N=2)			No Vismo (N=1)			
	All (N=4)	All (N=2)	Vismo Only (N=2)	Surgery Only (N=0)	Other Therapy (N=0)	All (N=1)	Surgery Only (N=1)	Other Therapy (N=0)	No Treatment (N=1)
If progression to metastatic disease or new metastases, indicate site(s)[2]									
Bone	0	0	0	0	0	0	0	0	0
Lymph nodes, Local	0	0	0	0	0	0	0	0	0
Lymph nodes, Distant	0	0	0	0	0	0	0	0	0
Lung	0	0	0	0	0	0	0	0	0
Liver	0	0	0	0	0	0	0	0	0
CNS/Brain	0	0	0	0	0	0	0	0	0
Other	0	0	0	0	0	0	0	0	0
What Methods were Used to Assess Disease Response/ Progression									
n	4	2	2	0	0	2	2	0	0
Diagnostic Image (MRI, CT)	0	0	0	0	0	0	0	0	0
Histopathologic Evaluation	0	0	0	0	0	0	0	0	0
Photography	0	0	0	0	0	0	0	0	0
Clinical Evaluation	4(100%)	2(100%)	2(100%)	0	0	2(100%)	2(100%)	0	0
Laboratory Evaluation	0	0	0	0	0	0	0	0	0
Other	0	0	0	0	0	0	0	0	0

Indicate How Response/ Progression was Assessed:									
n	0	0	0	0	0	0	0	0	0
Change in Size of Existing Lesions	0	0	0	0	0	0	0	0	0
Change in Number of Existing Lesions	0	0	0	0	0	0	0	0	0
Development of New BCCs	0	0	0	0	0	0	0	0	0
Progression from Locally Advanced Disease to Metastatic Disease	0	0	0	0	0	0	0	0	0
Development of New Metastases	0	0	0	0	0	0	0	0	0
Other	0	0	0	0	0	0	0	0	0
If progression to metastatic disease or new metastases, indicate site(s)[2]									
Bone	0	0	0	0	0	0	0	0	0
Lymph nodes, Local	0	0	0	0	0	0	0	0	0
Lymph nodes, Distant	0	0	0	0	0	0	0	0	0
Lung	0	0	0	0	0	0	0	0	0
Liver	0	0	0	0	0	0	0	0	0
CNS/Brain	0	0	0	0	0	0	0	0	0
Other	0	0	0	0	0	0	0	0	0

What Methods were Used to Assess Disease Response/ Progression									
n	0	0	0	0	0	0	0	0	0
Diagnostic Image (MRI, CT)	0	0	0	0	0	0	0	0	0
Histopathologic Evaluation	0	0	0	0	0	0	0	0	0
Photography	0	0	0	0	0	0	0	0	0
Clinical Evaluation	0	0	0	0	0	0	0	0	0
Laboratory Evaluation	0	0	0	0	0	0	0	0	0
Other	0	0	0	0	0	0	0	0	0

					Cohort 1: Newly Determined Advanced BCC Patients (Non-BCCNS) Locally Advanced BCC (laBCC)				
			Vismo (N=115)			No Vismo (N=251)			
	All (N=433)	All (N=115)	Vismo Only (N=102)	Surgery Only (N=6)	Other Therapy (N=7)	All (N=251)	Surgery Only (N=198)	Other Therapy (N=53)	No Treatment (N=67)
Routine Follow-up 23									
Response Assessment									
n	3	1	1	0	0	2	2	0	0
Complete Response (CR)	3(100%)	1(100%)	1(100%)	0	0	2(100%)	2(100%)	0	0
Partial Response (PR)	0	0	0	0	0	0	0	0	0
Stable Disease (SD)	0	0	0	0	0	0	0	0	0
Disease Progression (PD)	0	0	0	0	0	0	0	0	0
Recurrence	0	0	0	0	0	0	0	0	0
Clinical Deterioration	0	0	0	0	0	0	0	0	0
Unable to Evaluate	0	0	0	0	0	0	0	0	0

If Complete Response, as Determined by[1]									
Negative Histopathology	0	0	0	0	0	0	0	0	0
Complete Tumor Shrinkage	0	0	0	0	0	0	0	0	0
No Clinically Visible Evidence of BCC	3(100%)	1(100%)	1(100%)	0	0	2(100%)	2(100%)	0	0
Other	0	0	0	0	0	0	0	0	0

					Cohort 1: Newly Determined Advanced BCC Patients (Non-BCCNS) Metastatic BCC (mBCC)				
			Vismo (N=2)		No Vismo (N=1)				
	All (N=4)	All (N=2)	Vismo Only (N=2)	Surgery Only (N=0)	Other Therapy (N=0)	All (N=1)	Surgery Only (N=1)	Other Therapy (N=0)	No Treatment (N=1)
Routine Follow-up 23									
Response Assessment									
n	0	0	0	0	0	0	0	0	0
Complete Response (CR)	0	0	0	0	0	0	0	0	0
Partial Response (PR)	0	0	0	0	0	0	0	0	0
Stable Disease (SD)	0	0	0	0	0	0	0	0	0
Disease Progression (PD)	0	0	0	0	0	0	0	0	0
Recurrence	0	0	0	0	0	0	0	0	0
Clinical Deterioration	0	0	0	0	0	0	0	0	0
Unable to Evaluate	0	0	0	0	0	0	0	0	0
If Complete Response, as Determined by[1]									

Negative Histopathology	0	0	0	0	0	0	0	0	0
Complete Tumor Shrinkage	0	0	0	0	0	0	0	0	0
No Clinically Visible Evidence of BCC	0	0	0	0	0	0	0	0	0
Other	0	0	0	0	0	0	0	0	0

					Cohort 1: Newly Determined Advanced BCC Patients (Non-BCCNS) Locally Advanced BCC (laBCC)				
			Vismo (N=115)		No Vismo (N=251)				
	All (N=433)	All (N=115)	Vismo Only (N=102)	Surgery Only (N=6)	Other Therapy (N=7)	All (N=251)	Surgery Only (N=198)	Other Therapy (N=53)	No Treatment (N=67)
Indicate How Response/ Progression was Assessed:									
n	1	1	1	0	0	0	0	0	0
Change in Size of Existing Lesions	1(100%)	1(100%)	1(100%)	0	0	0	0	0	0
Change in Number of Existing Lesions	0	0	0	0	0	0	0	0	0
Development of New BCCs	0	0	0	0	0	0	0	0	0
Progression from Locally Advanced Disease to Metastatic Disease	0	0	0	0	0	0	0	0	0
Development of New Metastases	0	0	0	0	0	0	0	0	0
Other	0	0	0	0	0	0	0	0	0

If progression to metastatic disease or new metastases, indicate site(s)[2]									
Bone	0	0	0	0	0	0	0	0	0
Lymph nodes, Local	0	0	0	0	0	0	0	0	0
Lymph nodes, Distant	0	0	0	0	0	0	0	0	0
Lung	0	0	0	0	0	0	0	0	0
Liver	0	0	0	0	0	0	0	0	0
CNS/Brain	0	0	0	0	0	0	0	0	0
Other	0	0	0	0	0	0	0	0	0
What Methods were Used to Assess Disease Response/ Progression									
n	3	1	1	0	0	2	2	0	0
Diagnostic Image (MRI, CT)	0	0	0	0	0	0	0	0	0
Histopathologic Evaluation	0	0	0	0	0	0	0	0	0
Photography	0	0	0	0	0	0	0	0	0
Clinical Evaluation	3(100%)	1(100%)	1(100%)	0	0	2(100%)	2(100%)	0	0
Laboratory Evaluation	0	0	0	0	0	0	0	0	0
Other	0	0	0	0	0	0	0	0	0
					Cohort 1: Newly Determined Advanced BCC Patients (Non-BCCNS) Metastatic BCC (mBCC)				
			Vismo (N=2)		No Vismo (N=1)				
	All (N=4)	All (N=2)	Vismo Only (N=2)	Surgery Only (N=0)	Other Therapy (N=0)	All (N=1)	Surgery Only (N=1)	Other Therapy (N=0)	No Treatment (N=1)

Indicate How Response/ Progression was Assessed:									
n	0	0	0	0	0	0	0	0	0
Change in Size of Existing Lesions	0	0	0	0	0	0	0	0	0
Change in Number of Existing Lesions	0	0	0	0	0	0	0	0	0
Development of New BCCs	0	0	0	0	0	0	0	0	0
Progression from Locally Advanced Disease to Metastatic Disease	0	0	0	0	0	0	0	0	0
Development of New Metastases	0	0	0	0	0	0	0	0	0
Other	0	0	0	0	0	0	0	0	0
If progression to metastatic disease or new metastases, indicate site(s)[2]									
Bone	0	0	0	0	0	0	0	0	0
Lymph nodes, Local	0	0	0	0	0	0	0	0	0
Lymph nodes, Distant	0	0	0	0	0	0	0	0	0
Lung	0	0	0	0	0	0	0	0	0
Liver	0	0	0	0	0	0	0	0	0
CNS/Brain	0	0	0	0	0	0	0	0	0
Other	0	0	0	0	0	0	0	0	0

What Methods were Used to Assess Disease Response/ Progression									
n	0	0	0	0	0	0	0	0	0
Diagnostic Image (MRI, CT)	0	0	0	0	0	0	0	0	0
Histopathologic Evaluation	0	0	0	0	0	0	0	0	0
Photography	0	0	0	0	0	0	0	0	0
Clinical Evaluation	0	0	0	0	0	0	0	0	0
Laboratory Evaluation	0	0	0	0	0	0	0	0	0
Other	0	0	0	0	0	0	0	0	0
					Cohort 1: Newly Determined Advanced BCC Patients (Non-BCCNS) Locally Advanced BCC (laBCC)				
			Vismo (N=115)		No Vismo (N=251)				
	All (N=433)	All (N=115)	Vismo Only (N=102)	Surgery Only (N=6)	Other Therapy (N=7)	All (N=251)	Surgery Only (N=198)	Other Therapy (N=53)	No Treatment (N=67)
Routine Follow-up 24									
Response Assessment									
n	3	1	1	0	0	2	2	0	0
Complete Response (CR)	3(100%)	1(100%)	1(100%)	0	0	2(100%)	2(100%)	0	0
Partial Response (PR)	0	0	0	0	0	0	0	0	0
Stable Disease (SD)	0	0	0	0	0	0	0	0	0
Disease Progression (PD)	0	0	0	0	0	0	0	0	0
Recurrence	0	0	0	0	0	0	0	0	0
Clinical Deterioration	0	0	0	0	0	0	0	0	0
Unable to Evaluate	0	0	0	0	0	0	0	0	0

If Complete Response, as Determined by[1]									
Negative Histopathology	0	0	0	0	0	0	0	0	0
Complete Tumor Shrinkage	0	0	0	0	0	0	0	0	0
No Clinically Visible Evidence of BCC	3(100%)	1(100%)	1(100%)	0	0	2(100%)	2(100%)	0	0
Other	0	0	0	0	0	0	0	0	0
					Cohort 1: Newly Determined Advanced BCC Patients (Non-BCCNS) Metastatic BCC (mBCC)				
		Vismo (N=2)			No Vismo (N=1)				
	All (N=4)	All (N=2)	Vismo Only (N=2)	Surgery Only (N=0)	Other Therapy (N=0)	All (N=1)	Surgery Only (N=1)	Other Therapy (N=0)	No Treatment (N=1)
Routine Follow-up 24									
Response Assessment									
n	0	0	0	0	0	0	0	0	0
Complete Response (CR)	0	0	0	0	0	0	0	0	0
Partial Response (PR)	0	0	0	0	0	0	0	0	0
Stable Disease (SD)	0	0	0	0	0	0	0	0	0
Disease Progression (PD)	0	0	0	0	0	0	0	0	0
Recurrence	0	0	0	0	0	0	0	0	0
Clinical Deterioration	0	0	0	0	0	0	0	0	0
Unable to Evaluate	0	0	0	0	0	0	0	0	0
If Complete Response, as Determined by[1]									

Negative Histopathology	0	0	0	0	0	0	0	0	0
Complete Tumor Shrinkage	0	0	0	0	0	0	0	0	0
No Clinically Visible Evidence of BCC	0	0	0	0	0	0	0	0	0
Other	0	0	0	0	0	0	0	0	0
					Cohort 1: Newly Determined Advanced BCC Patients (Non-BCCNS) Locally Advanced BCC (laBCC)				
		Vismo (N=115)			No Vismo (N=251)				
	All (N=433)	All (N=115)	Vismo Only (N=102)	Surgery Only (N=6)	Other Therapy (N=7)	All (N=251)	Surgery Only (N=198)	Other Therapy (N=53)	No Treatment (N=67)
Indicate How Response/ Progression was Assessed:									
n	1	1	1	0	0	0	0	0	0
Change in Size of Existing Lesions	1(100%)	1(100%)	1(100%)	0	0	0	0	0	0
Change in Number of Existing Lesions	0	0	0	0	0	0	0	0	0
Development of New BCCs	0	0	0	0	0	0	0	0	0
Progression from Locally Advanced Disease to Metastatic Disease	0	0	0	0	0	0	0	0	0
Development of New Metastases	0	0	0	0	0	0	0	0	0
Other	0	0	0	0	0	0	0	0	0

					Cohort 1: Newly Determined Advanced BCC Patients (Non-BCCNS) Metastatic BCC (mBCC)				
		Vismo (N=2)			No Vismo (N=1)				
	All (N=4)	All (N=2)	Vismo Only (N=2)	Surgery Only (N=0)	Other Therapy (N=0)	All (N=1)	Surgery Only (N=1)	Other Therapy (N=0)	No Treatment (N=1)
If progression to metastatic disease or new metastases, indicate site(s)[2]									
Bone	0	0	0	0	0	0	0	0	0
Lymph nodes, Local	0	0	0	0	0	0	0	0	0
Lymph nodes, Distant	0	0	0	0	0	0	0	0	0
Lung	0	0	0	0	0	0	0	0	0
Liver	0	0	0	0	0	0	0	0	0
CNS/Brain	0	0	0	0	0	0	0	0	0
Other	0	0	0	0	0	0	0	0	0
What Methods were Used to Assess Disease Response/ Progression									
n	3	1	1	0	0	2	2	0	0
Diagnostic Image (MRI, CT)	0	0	0	0	0	0	0	0	0
Histopathologic Evaluation	0	0	0	0	0	0	0	0	0
Photography	0	0	0	0	0	0	0	0	0
Clinical Evaluation	3(100%)	1(100%)	1(100%)	0	0	2(100%)	2(100%)	0	0
Laboratory Evaluation	0	0	0	0	0	0	0	0	0
Other	0	0	0	0	0	0	0	0	0

Indicate How Response/ Progression was Assessed:									
n	0	0	0	0	0	0	0	0	0
Change in Size of Existing Lesions	0	0	0	0	0	0	0	0	0
Change in Number of Existing Lesions	0	0	0	0	0	0	0	0	0
Development of New BCCs	0	0	0	0	0	0	0	0	0
Progression from Locally Advanced Disease to Metastatic Disease	0	0	0	0	0	0	0	0	0
Development of New Metastases	0	0	0	0	0	0	0	0	0
Other	0	0	0	0	0	0	0	0	0
If progression to metastatic disease or new metastases, indicate site(s)[2]									
Bone	0	0	0	0	0	0	0	0	0
Lymph nodes, Local	0	0	0	0	0	0	0	0	0
Lymph nodes, Distant	0	0	0	0	0	0	0	0	0
Lung	0	0	0	0	0	0	0	0	0
Liver	0	0	0	0	0	0	0	0	0
CNS/Brain	0	0	0	0	0	0	0	0	0
Other	0	0	0	0	0	0	0	0	0

What Methods were Used to Assess Disease Response/ Progression									
n	0	0	0	0	0	0	0	0	0
Diagnostic Image (MRI, CT)	0	0	0	0	0	0	0	0	0
Histopathologic Evaluation	0	0	0	0	0	0	0	0	0
Photography	0	0	0	0	0	0	0	0	0
Clinical Evaluation	0	0	0	0	0	0	0	0	0
Laboratory Evaluation	0	0	0	0	0	0	0	0	0
Other	0	0	0	0	0	0	0	0	0

					Cohort 1: Newly Determined Advanced BCC Patients (Non-BCCNS) Locally Advanced BCC (laBCC)				
		Vismo (N=115)			No Vismo (N=251)				
	All (N=433)	All (N=115)	Vismo Only (N=102)	Surgery Only (N=6)	Other Therapy (N=7)	All (N=251)	Surgery Only (N=198)	Other Therapy (N=53)	No Treatment (N=67)
Routine Follow-up 25									
Response Assessment									
n	2	1	1	0	0	1	1	0	0
Complete Response (CR)	2(100%)	1(100%)	1(100%)	0	0	1(100%)	1(100%)	0	0
Partial Response (PR)	0	0	0	0	0	0	0	0	0
Stable Disease (SD)	0	0	0	0	0	0	0	0	0
Disease Progression (PD)	0	0	0	0	0	0	0	0	0
Recurrence	0	0	0	0	0	0	0	0	0
Clinical Deterioration	0	0	0	0	0	0	0	0	0
Unable to Evaluate	0	0	0	0	0	0	0	0	0

If Complete Response, as Determined by[1]									
Negative Histopathology	0	0	0	0	0	0	0	0	0
Complete Tumor Shrinkage	0	0	0	0	0	0	0	0	0
No Clinically Visible Evidence of BCC	2(100%)	1(100%)	1(100%)	0	0	1(100%)	1(100%)	0	0
Other	0	0	0	0	0	0	0	0	0

					Cohort 1: Newly Determined Advanced BCC Patients (Non-BCCNS) Metastatic BCC (mBCC)				
		Vismo (N=2)			No Vismo (N=1)				
	All (N=4)	All (N=2)	Vismo Only (N=2)	Surgery Only (N=0)	Other Therapy (N=0)	All (N=1)	Surgery Only (N=1)	Other Therapy (N=0)	No Treatment (N=1)
Routine Follow-up 25									
Response Assessment									
n	0	0	0	0	0	0	0	0	0
Complete Response (CR)	0	0	0	0	0	0	0	0	0
Partial Response (PR)	0	0	0	0	0	0	0	0	0
Stable Disease (SD)	0	0	0	0	0	0	0	0	0
Disease Progression (PD)	0	0	0	0	0	0	0	0	0
Recurrence	0	0	0	0	0	0	0	0	0
Clinical Deterioration	0	0	0	0	0	0	0	0	0
Unable to Evaluate	0	0	0	0	0	0	0	0	0
If Complete Response, as Determined by[1]									

Negative Histopathology	0	0	0	0	0	0	0	0	0
Complete Tumor Shrinkage	0	0	0	0	0	0	0	0	0
No Clinically Visible Evidence of BCC	0	0	0	0	0	0	0	0	0
Other	0	0	0	0	0	0	0	0	0
					Cohort 1: Newly Determined Advanced BCC Patients (Non-BCCNS) Locally Advanced BCC (laBCC)				
		Vismo (N=115)			No Vismo (N=251)				
	All (N=433)	All (N=115)	Vismo Only (N=102)	Surgery Only (N=6)	Other Therapy (N=7)	All (N=251)	Surgery Only (N=198)	Other Therapy (N=53)	No Treatment (N=67)
Indicate How Response/ Progression was Assessed:									
n	1	1	1	0	0	0	0	0	0
Change in Size of Existing Lesions	1(100%)	1(100%)	1(100%)	0	0	0	0	0	0
Change in Number of Existing Lesions	0	0	0	0	0	0	0	0	0
Development of New BCCs	0	0	0	0	0	0	0	0	0
Progression from Locally Advanced Disease to Metastatic Disease	0	0	0	0	0	0	0	0	0
Development of New Metastases	0	0	0	0	0	0	0	0	0
Other	0	0	0	0	0	0	0	0	0

If progression to metastatic disease or new metastases, indicate site(s)[2]									
Bone	0	0	0	0	0	0	0	0	0
Lymph nodes, Local	0	0	0	0	0	0	0	0	0
Lymph nodes, Distant	0	0	0	0	0	0	0	0	0
Lung	0	0	0	0	0	0	0	0	0
Liver	0	0	0	0	0	0	0	0	0
CNS/Brain	0	0	0	0	0	0	0	0	0
Other	0	0	0	0	0	0	0	0	0
What Methods were Used to Assess Disease Response/ Progression									
n	2	1	1	0	0	1	1	0	0
Diagnostic Image (MRI, CT)	0	0	0	0	0	0	0	0	0
Histopathologic Evaluation	0	0	0	0	0	0	0	0	0
Photography	0	0	0	0	0	0	0	0	0
Clinical Evaluation	2(100%)	1(100%)	1(100%)	0	0	1(100%)	1(100%)	0	0
Laboratory Evaluation	0	0	0	0	0	0	0	0	0
Other	0	0	0	0	0	0	0	0	0
					Cohort 1: Newly Determined Advanced BCC Patients (Non-BCCNS) Metastatic BCC (mBCC)				
		Vismo (N=2)			No Vismo (N=1)				
	All (N=4)	All (N=2)	Vismo Only (N=2)	Surgery Only (N=0)	Other Therapy (N=0)	All (N=1)	Surgery Only (N=1)	Other Therapy (N=0)	No Treatment (N=1)

Indicate How Response/ Progression was Assessed:									
n	0	0	0	0	0	0	0	0	0
Change in Size of Existing Lesions	0	0	0	0	0	0	0	0	0
Change in Number of Existing Lesions	0	0	0	0	0	0	0	0	0
Development of New BCCs	0	0	0	0	0	0	0	0	0
Progression from Locally Advanced Disease to Metastatic Disease	0	0	0	0	0	0	0	0	0
Development of New Metastases	0	0	0	0	0	0	0	0	0
Other	0	0	0	0	0	0	0	0	0
If progression to metastatic disease or new metastases, indicate site(s)[2]									
Bone	0	0	0	0	0	0	0	0	0
Lymph nodes, Local	0	0	0	0	0	0	0	0	0
Lymph nodes, Distant	0	0	0	0	0	0	0	0	0
Lung	0	0	0	0	0	0	0	0	0
Liver	0	0	0	0	0	0	0	0	0
CNS/Brain	0	0	0	0	0	0	0	0	0
Other	0	0	0	0	0	0	0	0	0

What Methods were Used to Assess Disease Response/ Progression									
n	0	0	0	0	0	0	0	0	0
Diagnostic Image (MRI, CT)	0	0	0	0	0	0	0	0	0
Histopathologic Evaluation	0	0	0	0	0	0	0	0	0
Photography	0	0	0	0	0	0	0	0	0
Clinical Evaluation	0	0	0	0	0	0	0	0	0
Laboratory Evaluation	0	0	0	0	0	0	0	0	0
Other	0	0	0	0	0	0	0	0	0
					Cohort 1: Newly Determined Advanced BCC Patients (Non-BCCNS) Locally Advanced BCC (laBCC)				
		Vismo (N=115)			No Vismo (N=251)				
	All (N=433)	All (N=115)	Vismo Only (N=102)	Surgery Only (N=6)	Other Therapy (N=7)	All (N=251)	Surgery Only (N=198)	Other Therapy (N=53)	No Treatment (N=67)
Routine Follow-up 26									
Response Assessment									
n	2	1	1	0	0	1	1	0	0
Complete Response (CR)	2(100%)	1(100%)	1(100%)	0	0	1(100%)	1(100%)	0	0
Partial Response (PR)	0	0	0	0	0	0	0	0	0
Stable Disease (SD)	0	0	0	0	0	0	0	0	0
Disease Progression (PD)	0	0	0	0	0	0	0	0	0
Recurrence	0	0	0	0	0	0	0	0	0
Clinical Deterioration	0	0	0	0	0	0	0	0	0
Unable to Evaluate	0	0	0	0	0	0	0	0	0

If Complete Response, as Determined by[1]									
Negative Histopathology	0	0	0	0	0	0	0	0	0
Complete Tumor Shrinkage	0	0	0	0	0	0	0	0	0
No Clinically Visible Evidence of BCC	2(100%)	1(100%)	1(100%)	0	0	1(100%)	1(100%)	0	0
Other	0	0	0	0	0	0	0	0	0

					Cohort 1: Newly Determined Advanced BCC Patients (Non-BCCNS) Metastatic BCC (mBCC)				
			Vismo (N=2)			No Vismo (N=1)			
	All (N=4)	All (N=2)	Vismo Only (N=2)	Surgery Only (N=0)	Other Therapy (N=0)	All (N=1)	Surgery Only (N=1)	Other Therapy (N=0)	No Treatment (N=1)
Routine Follow-up 26									
Response Assessment									
n	0	0	0	0	0	0	0	0	0
Complete Response (CR)	0	0	0	0	0	0	0	0	0
Partial Response (PR)	0	0	0	0	0	0	0	0	0
Stable Disease (SD)	0	0	0	0	0	0	0	0	0
Disease Progression (PD)	0	0	0	0	0	0	0	0	0
Recurrence	0	0	0	0	0	0	0	0	0
Clinical Deterioration	0	0	0	0	0	0	0	0	0
Unable to Evaluate	0	0	0	0	0	0	0	0	0
If Complete Response, as Determined by[1]									

Negative Histopathology	0	0	0	0	0	0	0	0	0
Complete Tumor Shrinkage	0	0	0	0	0	0	0	0	0
No Clinically Visible Evidence of BCC	0	0	0	0	0	0	0	0	0
Other	0	0	0	0	0	0	0	0	0

	Cohort 1: Newly Determined Advanced BCC Patients (Non-BCCNS) Locally Advanced BCC (laBCC)								
		Vismo (N=115)			No Vismo (N=251)				
	All (N=433)	All (N=115)	Vismo Only (N=102)	Surgery Only (N=6)	Other Therapy (N=7)	All (N=251)	Surgery Only (N=198)	Other Therapy (N=53)	No Treatment (N=67)
Indicate How Response/ Progression was Assessed:									
n	1	1	1	0	0	0	0	0	0
Change in Size of Existing Lesions	0	0	0	0	0	0	0	0	0
Change in Number of Existing Lesions	1(100%)	1(100%)	1(100%)	0	0	0	0	0	0
Development of New BCCs	0	0	0	0	0	0	0	0	0
Progression from Locally Advanced Disease to Metastatic Disease	0	0	0	0	0	0	0	0	0
Development of New Metastases	0	0	0	0	0	0	0	0	0
Other	0	0	0	0	0	0	0	0	0

					Cohort 1: Newly Determined Advanced BCC Patients (Non-BCCNS) Metastatic BCC (mBCC)				
		Vismo (N=2)			No Vismo (N=1)				
	All (N=4)	All (N=2)	Vismo Only (N=2)	Surgery Only (N=0)	Other Therapy (N=0)	All (N=1)	Surgery Only (N=1)	Other Therapy (N=0)	No Treatment (N=1)
If progression to metastatic disease or new metastases, indicate site(s)[2]									
Bone	0	0	0	0	0	0	0	0	0
Lymph nodes, Local	0	0	0	0	0	0	0	0	0
Lymph nodes, Distant	0	0	0	0	0	0	0	0	0
Lung	0	0	0	0	0	0	0	0	0
Liver	0	0	0	0	0	0	0	0	0
CNS/Brain	0	0	0	0	0	0	0	0	0
Other	0	0	0	0	0	0	0	0	0
What Methods were Used to Assess Disease Response/ Progression									
n	2	1	1	0	0	1	1	0	0
Diagnostic Image (MRI, CT)	0	0	0	0	0	0	0	0	0
Histopathologic Evaluation	0	0	0	0	0	0	0	0	0
Photography	0	0	0	0	0	0	0	0	0
Clinical Evaluation	2(100%)	1(100%)	1(100%)	0	0	1(100%)	1(100%)	0	0
Laboratory Evaluation	0	0	0	0	0	0	0	0	0
Other	0	0	0	0	0	0	0	0	0

Indicate How Response/ Progression was Assessed:									
n	0	0	0	0	0	0	0	0	0
Change in Size of Existing Lesions	0	0	0	0	0	0	0	0	0
Change in Number of Existing Lesions	0	0	0	0	0	0	0	0	0
Development of New BCCs	0	0	0	0	0	0	0	0	0
Progression from Locally Advanced Disease to Metastatic Disease	0	0	0	0	0	0	0	0	0
Development of New Metastases	0	0	0	0	0	0	0	0	0
Other	0	0	0	0	0	0	0	0	0
If progression to metastatic disease or new metastases, indicate site(s)[2]									
Bone	0	0	0	0	0	0	0	0	0
Lymph nodes, Local	0	0	0	0	0	0	0	0	0
Lymph nodes, Distant	0	0	0	0	0	0	0	0	0
Lung	0	0	0	0	0	0	0	0	0
Liver	0	0	0	0	0	0	0	0	0
CNS/Brain	0	0	0	0	0	0	0	0	0
Other	0	0	0	0	0	0	0	0	0

What Methods were Used to Assess Disease Response/ Progression									
n	0	0	0	0	0	0	0	0	0
Diagnostic Image (MRI, CT)	0	0	0	0	0	0	0	0	0
Histopathologic Evaluation	0	0	0	0	0	0	0	0	0
Photography	0	0	0	0	0	0	0	0	0
Clinical Evaluation	0	0	0	0	0	0	0	0	0
Laboratory Evaluation	0	0	0	0	0	0	0	0	0
Other	0	0	0	0	0	0	0	0	0

					Cohort 1: Newly Determined Advanced BCC Patients (Non-BCCNS) Locally Advanced BCC (laBCC)				
		Vismo (N=115)			No Vismo (N=251)				
	All (N=433)	All (N=115)	Vismo Only (N=102)	Surgery Only (N=6)	Other Therapy (N=7)	All (N=251)	Surgery Only (N=198)	Other Therapy (N=53)	No Treatment (N=67)
Routine Follow-up 27									
Response Assessment									
n	1	1	1	0	0	0	0	0	0
Complete Response (CR)	1(100%)	1(100%)	1(100%)	0	0	0	0	0	0
Partial Response (PR)	0	0	0	0	0	0	0	0	0
Stable Disease (SD)	0	0	0	0	0	0	0	0	0
Disease Progression (PD)	0	0	0	0	0	0	0	0	0
Recurrence	0	0	0	0	0	0	0	0	0
Clinical Deterioration	0	0	0	0	0	0	0	0	0
Unable to Evaluate	0	0	0	0	0	0	0	0	0

If Complete Response, as Determined by[1]									
Negative Histopathology	0	0	0	0	0	0	0	0	0
Complete Tumor Shrinkage	0	0	0	0	0	0	0	0	0
No Clinically Visible Evidence of BCC	1(100%)	1(100%)	1(100%)	0	0	0	0	0	0
Other	0	0	0	0	0	0	0	0	0

					Cohort 1: Newly Determined Advanced BCC Patients (Non-BCCNS) Metastatic BCC (mBCC)				
		Vismo (N=2)			No Vismo (N=1)				
	All (N=4)	All (N=2)	Vismo Only (N=2)	Surgery Only (N=0)	Other Therapy (N=0)	All (N=1)	Surgery Only (N=1)	Other Therapy (N=0)	No Treatment (N=1)
Routine Follow-up 27									
Response Assessment									
n	0	0	0	0	0	0	0	0	0
Complete Response (CR)	0	0	0	0	0	0	0	0	0
Partial Response (PR)	0	0	0	0	0	0	0	0	0
Stable Disease (SD)	0	0	0	0	0	0	0	0	0
Disease Progression (PD)	0	0	0	0	0	0	0	0	0
Recurrence	0	0	0	0	0	0	0	0	0
Clinical Deterioration	0	0	0	0	0	0	0	0	0
Unable to Evaluate	0	0	0	0	0	0	0	0	0
If Complete Response, as Determined by[1]									

Negative Histopathology	0	0	0	0	0	0	0	0	0
Complete Tumor Shrinkage	0	0	0	0	0	0	0	0	0
No Clinically Visible Evidence of BCC	0	0	0	0	0	0	0	0	0
Other	0	0	0	0	0	0	0	0	0

					Cohort 1: Newly Determined Advanced BCC Patients (Non-BCCNS) Locally Advanced BCC (laBCC)				
		Vismo (N=115)			No Vismo (N=251)				
	All (N=433)	All (N=115)	Vismo Only (N=102)	Surgery Only (N=6)	Other Therapy (N=7)	All (N=251)	Surgery Only (N=198)	Other Therapy (N=53)	No Treatment (N=67)
Indicate How Response/ Progression was Assessed:									
n	1	1	1	0	0	0	0	0	0
Change in Size of Existing Lesions	0	0	0	0	0	0	0	0	0
Change in Number of Existing Lesions	1(100%)	1(100%)	1(100%)	0	0	0	0	0	0
Development of New BCCs	0	0	0	0	0	0	0	0	0
Progression from Locally Advanced Disease to Metastatic Disease	0	0	0	0	0	0	0	0	0
Development of New Metastases	0	0	0	0	0	0	0	0	0
Other	0	0	0	0	0	0	0	0	0

If progression to metastatic disease or new metastases, indicate site(s)[2]									
Bone	0	0	0	0	0	0	0	0	0
Lymph nodes, Local	0	0	0	0	0	0	0	0	0
Lymph nodes, Distant	0	0	0	0	0	0	0	0	0
Lung	0	0	0	0	0	0	0	0	0
Liver	0	0	0	0	0	0	0	0	0
CNS/Brain	0	0	0	0	0	0	0	0	0
Other	0	0	0	0	0	0	0	0	0
What Methods were Used to Assess Disease Response/ Progression									
n	1	1	1	0	0	0	0	0	0
Diagnostic Image (MRI, CT)	0	0	0	0	0	0	0	0	0
Histopathologic Evaluation	0	0	0	0	0	0	0	0	0
Photography	0	0	0	0	0	0	0	0	0
Clinical Evaluation	1(100%)	1(100%)	1(100%)	0	0	0	0	0	0
Laboratory Evaluation	0	0	0	0	0	0	0	0	0
Other	0	0	0	0	0	0	0	0	0
					Cohort 1: Newly Determined Advanced BCC Patients (Non-BCCNS) Metastatic BCC (mBCC)				
			Vismo (N=2)			No Vismo (N=1)			
	All (N=4)	All (N=2)	Vismo Only (N=2)	Surgery Only (N=0)	Other Therapy (N=0)	All (N=1)	Surgery Only (N=1)	Other Therapy (N=0)	No Treatment (N=1)

Indicate How Response/ Progression was Assessed:									
n	0	0	0	0	0	0	0	0	0
Change in Size of Existing Lesions	0	0	0	0	0	0	0	0	0
Change in Number of Existing Lesions	0	0	0	0	0	0	0	0	0
Development of New BCCs	0	0	0	0	0	0	0	0	0
Progression from Locally Advanced Disease to Metastatic Disease	0	0	0	0	0	0	0	0	0
Development of New Metastases	0	0	0	0	0	0	0	0	0
Other	0	0	0	0	0	0	0	0	0
If progression to metastatic disease or new metastases, indicate site(s)[2]									
Bone	0	0	0	0	0	0	0	0	0
Lymph nodes, Local	0	0	0	0	0	0	0	0	0
Lymph nodes, Distant	0	0	0	0	0	0	0	0	0
Lung	0	0	0	0	0	0	0	0	0
Liver	0	0	0	0	0	0	0	0	0
CNS/Brain	0	0	0	0	0	0	0	0	0
Other	0	0	0	0	0	0	0	0	0

What Methods were Used to Assess Disease Response/ Progression									
n	0	0	0	0	0	0	0	0	0
Diagnostic Image (MRI, CT)	0	0	0	0	0	0	0	0	0
Histopathologic Evaluation	0	0	0	0	0	0	0	0	0
Photography	0	0	0	0	0	0	0	0	0
Clinical Evaluation	0	0	0	0	0	0	0	0	0
Laboratory Evaluation	0	0	0	0	0	0	0	0	0
Other	0	0	0	0	0	0	0	0	0

					Cohort 1: Newly Determined Advanced BCC Patients (Non-BCCNS) Locally Advanced BCC (laBCC)				
			Vismo (N=115)			No Vismo (N=251)			
	All (N=433)	All (N=115)	Vismo Only (N=102)	Surgery Only (N=6)	Other Therapy (N=7)	All (N=251)	Surgery Only (N=198)	Other Therapy (N=53)	No Treatment (N=67)
Routine Follow-up 28									
Response Assessment									
n	1	1	1	0	0	0	0	0	0
Complete Response (CR)	1(100%)	1(100%)	1(100%)	0	0	0	0	0	0
Partial Response (PR)	0	0	0	0	0	0	0	0	0
Stable Disease (SD)	0	0	0	0	0	0	0	0	0
Disease Progression (PD)	0	0	0	0	0	0	0	0	0
Recurrence	0	0	0	0	0	0	0	0	0
Clinical Deterioration	0	0	0	0	0	0	0	0	0
Unable to Evaluate	0	0	0	0	0	0	0	0	0

If Complete Response, as Determined by[1]									
Negative Histopathology	0	0	0	0	0	0	0	0	0
Complete Tumor Shrinkage	0	0	0	0	0	0	0	0	0
No Clinically Visible Evidence of BCC	1(100%)	1(100%)	1(100%)	0	0	0	0	0	0
Other	0	0	0	0	0	0	0	0	0

					Cohort 1: Newly Determined Advanced BCC Patients (Non-BCCNS) Metastatic BCC (mBCC)				
		Vismo (N=2)			No Vismo (N=1)				
	All (N=4)	All (N=2)	Vismo Only (N=2)	Surgery Only (N=0)	Other Therapy (N=0)	All (N=1)	Surgery Only (N=1)	Other Therapy (N=0)	No Treatment (N=1)
Routine Follow-up 28									
Response Assessment									
n	0	0	0	0	0	0	0	0	0
Complete Response (CR)	0	0	0	0	0	0	0	0	0
Partial Response (PR)	0	0	0	0	0	0	0	0	0
Stable Disease (SD)	0	0	0	0	0	0	0	0	0
Disease Progression (PD)	0	0	0	0	0	0	0	0	0
Recurrence	0	0	0	0	0	0	0	0	0
Clinical Deterioration	0	0	0	0	0	0	0	0	0
Unable to Evaluate	0	0	0	0	0	0	0	0	0
If Complete Response, as Determined by[1]									

Negative Histopathology	0	0	0	0	0	0	0	0	0
Complete Tumor Shrinkage	0	0	0	0	0	0	0	0	0
No Clinically Visible Evidence of BCC	0	0	0	0	0	0	0	0	0
Other	0	0	0	0	0	0	0	0	0

					Cohort 1: Newly Determined Advanced BCC Patients (Non-BCCNS) Locally Advanced BCC (laBCC)				
		Vismo (N=115)			No Vismo (N=251)				
	All (N=433)	All (N=115)	Vismo Only (N=102)	Surgery Only (N=6)	Other Therapy (N=7)	All (N=251)	Surgery Only (N=198)	Other Therapy (N=53)	No Treatment (N=67)
Indicate How Response/ Progression was Assessed:									
n	1	1	1	0	0	0	0	0	0
Change in Size of Existing Lesions	0	0	0	0	0	0	0	0	0
Change in Number of Existing Lesions	1(100%)	1(100%)	1(100%)	0	0	0	0	0	0
Development of New BCCs	0	0	0	0	0	0	0	0	0
Progression from Locally Advanced Disease to Metastatic Disease	0	0	0	0	0	0	0	0	0
Development of New Metastases	0	0	0	0	0	0	0	0	0
Other	0	0	0	0	0	0	0	0	0

If progression to metastatic disease or new metastases, indicate site(s)[2]									
Bone	0	0	0	0	0	0	0	0	0
Lymph nodes, Local	0	0	0	0	0	0	0	0	0
Lymph nodes, Distant	0	0	0	0	0	0	0	0	0
Lung	0	0	0	0	0	0	0	0	0
Liver	0	0	0	0	0	0	0	0	0
CNS/Brain	0	0	0	0	0	0	0	0	0
Other	0	0	0	0	0	0	0	0	0
What Methods were Used to Assess Disease Response/ Progression									
n	1	1	1	0	0	0	0	0	0
Diagnostic Image (MRI, CT)	0	0	0	0	0	0	0	0	0
Histopathologic Evaluation	0	0	0	0	0	0	0	0	0
Photography	0	0	0	0	0	0	0	0	0
Clinical Evaluation	1(100%)	1(100%)	1(100%)	0	0	0	0	0	0
Laboratory Evaluation	0	0	0	0	0	0	0	0	0
Other	0	0	0	0	0	0	0	0	0
					Cohort 1: Newly Determined Advanced BCC Patients (Non-BCCNS) Metastatic BCC (mBCC)				
			Vismo (N=2)		No Vismo (N=1)				
	All (N=4)	All (N=2)	Vismo Only (N=2)	Surgery Only (N=0)	Other Therapy (N=0)	All (N=1)	Surgery Only (N=1)	Other Therapy (N=0)	No Treatment (N=1)

Indicate How Response/ Progression was Assessed:									
n	0	0	0	0	0	0	0	0	0
Change in Size of Existing Lesions	0	0	0	0	0	0	0	0	0
Change in Number of Existing Lesions	0	0	0	0	0	0	0	0	0
Development of New BCCs	0	0	0	0	0	0	0	0	0
Progression from Locally Advanced Disease to Metastatic Disease	0	0	0	0	0	0	0	0	0
Development of New Metastases	0	0	0	0	0	0	0	0	0
Other	0	0	0	0	0	0	0	0	0
If progression to metastatic disease or new metastases, indicate site(s)[2]									
Bone	0	0	0	0	0	0	0	0	0
Lymph nodes, Local	0	0	0	0	0	0	0	0	0
Lymph nodes, Distant	0	0	0	0	0	0	0	0	0
Lung	0	0	0	0	0	0	0	0	0
Liver	0	0	0	0	0	0	0	0	0
CNS/Brain	0	0	0	0	0	0	0	0	0
Other	0	0	0	0	0	0	0	0	0

What Methods were Used to Assess Disease Response/ Progression									
n	0	0	0	0	0	0	0	0	0
Diagnostic Image (MRI, CT)	0	0	0	0	0	0	0	0	0
Histopathologic Evaluation	0	0	0	0	0	0	0	0	0
Photography	0	0	0	0	0	0	0	0	0
Clinical Evaluation	0	0	0	0	0	0	0	0	0
Laboratory Evaluation	0	0	0	0	0	0	0	0	0
Other	0	0	0	0	0	0	0	0	0

					Cohort 1: Newly Determined Advanced BCC Patients (Non-BCCNS) Locally Advanced BCC (laBCC)				
		Vismo (N=115)			No Vismo (N=251)				
	All (N=433)	All (N=115)	Vismo Only (N=102)	Surgery Only (N=6)	Other Therapy (N=7)	All (N=251)	Surgery Only (N=198)	Other Therapy (N=53)	No Treatment (N=67)
Routine Follow-up 29									
Response Assessment									
n	1	1	1	0	0	0	0	0	0
Complete Response (CR)	0	0	0	0	0	0	0	0	0
Partial Response (PR)	0	0	0	0	0	0	0	0	0
Stable Disease (SD)	0	0	0	0	0	0	0	0	0
Disease Progression (PD)	0	0	0	0	0	0	0	0	0
Recurrence	1(100%)	1(100%)	1(100%)	0	0	0	0	0	0
Clinical Deterioration	0	0	0	0	0	0	0	0	0
Unable to Evaluate	0	0	0	0	0	0	0	0	0

If Complete Response, as Determined by[1]									
Negative Histopathology	0	0	0	0	0	0	0	0	0
Complete Tumor Shrinkage	0	0	0	0	0	0	0	0	0
No Clinically Visible Evidence of BCC	0	0	0	0	0	0	0	0	0
Other	0	0	0	0	0	0	0	0	0

	Cohort 1: Newly Determined Advanced BCC Patients (Non-BCCNS) Metastatic BCC (mBCC)								
		Vismo (N=2)			No Vismo (N=1)				
	All (N=4)	All (N=2)	Vismo Only (N=2)	Surgery Only (N=0)	Other Therapy (N=0)	All (N=1)	Surgery Only (N=1)	Other Therapy (N=0)	No Treatment (N=1)
Routine Follow-up 29									
Response Assessment									
n	0	0	0	0	0	0	0	0	0
Complete Response (CR)	0	0	0	0	0	0	0	0	0
Partial Response (PR)	0	0	0	0	0	0	0	0	0
Stable Disease (SD)	0	0	0	0	0	0	0	0	0
Disease Progression (PD)	0	0	0	0	0	0	0	0	0
Recurrence	0	0	0	0	0	0	0	0	0
Clinical Deterioration	0	0	0	0	0	0	0	0	0
Unable to Evaluate	0	0	0	0	0	0	0	0	0
If Complete Response, as Determined by[1]									

Negative Histopathology	0	0	0	0	0	0	0	0	0
Complete Tumor Shrinkage	0	0	0	0	0	0	0	0	0
No Clinically Visible Evidence of BCC	0	0	0	0	0	0	0	0	0
Other	0	0	0	0	0	0	0	0	0

					Cohort 1: Newly Determined Advanced BCC Patients (Non-BCCNS) Locally Advanced BCC (laBCC)				
		Vismo (N=115)			No Vismo (N=251)				
	All (N=433)	All (N=115)	Vismo Only (N=102)	Surgery Only (N=6)	Other Therapy (N=7)	All (N=251)	Surgery Only (N=198)	Other Therapy (N=53)	No Treatment (N=67)
Indicate How Response/ Progression was Assessed:									
n	1	1	1	0	0	0	0	0	0
Change in Size of Existing Lesions	0	0	0	0	0	0	0	0	0
Change in Number of Existing Lesions	1(100%)	1(100%)	1(100%)	0	0	0	0	0	0
Development of New BCCs	0	0	0	0	0	0	0	0	0
Progression from Locally Advanced Disease to Metastatic Disease	0	0	0	0	0	0	0	0	0
Development of New Metastases	0	0	0	0	0	0	0	0	0
Other	0	0	0	0	0	0	0	0	0

If progression to metastatic disease or new metastases, indicate site(s)[2]									
Bone	0	0	0	0	0	0	0	0	0
Lymph nodes, Local	0	0	0	0	0	0	0	0	0
Lymph nodes, Distant	0	0	0	0	0	0	0	0	0
Lung	0	0	0	0	0	0	0	0	0
Liver	0	0	0	0	0	0	0	0	0
CNS/Brain	0	0	0	0	0	0	0	0	0
Other	0	0	0	0	0	0	0	0	0
What Methods were Used to Assess Disease Response/ Progression									
n	1	1	1	0	0	0	0	0	0
Diagnostic Image (MRI, CT)	0	0	0	0	0	0	0	0	0
Histopathologic Evaluation	0	0	0	0	0	0	0	0	0
Photography	0	0	0	0	0	0	0	0	0
Clinical Evaluation	1(100%)	1(100%)	1(100%)	0	0	0	0	0	0
Laboratory Evaluation	0	0	0	0	0	0	0	0	0
Other	0	0	0	0	0	0	0	0	0
					Cohort 1: Newly Determined Advanced BCC Patients (Non-BCCNS) Metastatic BCC (mBCC)				
			Vismo (N=2)		No Vismo (N=1)				
	All (N=4)	All (N=2)	Vismo Only (N=2)	Surgery Only (N=0)	Other Therapy (N=0)	All (N=1)	Surgery Only (N=1)	Other Therapy (N=0)	No Treatment (N=1)

Indicate How Response/ Progression was Assessed:									
n	0	0	0	0	0	0	0	0	0
Change in Size of Existing Lesions	0	0	0	0	0	0	0	0	0
Change in Number of Existing Lesions	0	0	0	0	0	0	0	0	0
Development of New BCCs	0	0	0	0	0	0	0	0	0
Progression from Locally Advanced Disease to Metastatic Disease	0	0	0	0	0	0	0	0	0
Development of New Metastases	0	0	0	0	0	0	0	0	0
Other	0	0	0	0	0	0	0	0	0
If progression to metastatic disease or new metastases, indicate site(s)[2]									
Bone	0	0	0	0	0	0	0	0	0
Lymph nodes, Local	0	0	0	0	0	0	0	0	0
Lymph nodes, Distant	0	0	0	0	0	0	0	0	0
Lung	0	0	0	0	0	0	0	0	0
Liver	0	0	0	0	0	0	0	0	0
CNS/Brain	0	0	0	0	0	0	0	0	0
Other	0	0	0	0	0	0	0	0	0

What Methods were Used to Assess Disease Response/ Progression									
n	0	0	0	0	0	0	0	0	0
Diagnostic Image (MRI, CT)	0	0	0	0	0	0	0	0	0
Histopathologic Evaluation	0	0	0	0	0	0	0	0	0
Photography	0	0	0	0	0	0	0	0	0
Clinical Evaluation	0	0	0	0	0	0	0	0	0
Laboratory Evaluation	0	0	0	0	0	0	0	0	0
Other	0	0	0	0	0	0	0	0	0

					Cohort 1: Newly Determined Advanced BCC Patients (Non-BCCNS) Locally Advanced BCC (laBCC)				
		Vismo (N=115)			No Vismo (N=251)				
	All (N=433)	All (N=115)	Vismo Only (N=102)	Surgery Only (N=6)	Other Therapy (N=7)	All (N=251)	Surgery Only (N=198)	Other Therapy (N=53)	No Treatment (N=67)
Routine Follow-up 30									
Response Assessment									
n	1	1	1	0	0	0	0	0	0
Complete Response (CR)	0	0	0	0	0	0	0	0	0
Partial Response (PR)	0	0	0	0	0	0	0	0	0
Stable Disease (SD)	1(100%)	1(100%)	1(100%)	0	0	0	0	0	0
Disease Progression (PD)	0	0	0	0	0	0	0	0	0
Recurrence	0	0	0	0	0	0	0	0	0
Clinical Deterioration	0	0	0	0	0	0	0	0	0
Unable to Evaluate	0	0	0	0	0	0	0	0	0

If Complete Response, as Determined by[1]									
Negative Histopathology	0	0	0	0	0	0	0	0	0
Complete Tumor Shrinkage	0	0	0	0	0	0	0	0	0
No Clinically Visible Evidence of BCC	0	0	0	0	0	0	0	0	0
Other	0	0	0	0	0	0	0	0	0

					Cohort 1: Newly Determined Advanced BCC Patients (Non-BCCNS) Metastatic BCC (mBCC)				
		Vismo (N=2)			No Vismo (N=1)				
	All (N=4)	All (N=2)	Vismo Only (N=2)	Surgery Only (N=0)	Other Therapy (N=0)	All (N=1)	Surgery Only (N=1)	Other Therapy (N=0)	No Treatment (N=1)
Routine Follow-up 30									
Response Assessment									
n	0	0	0	0	0	0	0	0	0
Complete Response (CR)	0	0	0	0	0	0	0	0	0
Partial Response (PR)	0	0	0	0	0	0	0	0	0
Stable Disease (SD)	0	0	0	0	0	0	0	0	0
Disease Progression (PD)	0	0	0	0	0	0	0	0	0
Recurrence	0	0	0	0	0	0	0	0	0
Clinical Deterioration	0	0	0	0	0	0	0	0	0
Unable to Evaluate	0	0	0	0	0	0	0	0	0
If Complete Response, as Determined by[1]									

Negative Histopathology	0	0	0	0	0	0	0	0	0
Complete Tumor Shrinkage	0	0	0	0	0	0	0	0	0
No Clinically Visible Evidence of BCC	0	0	0	0	0	0	0	0	0
Other	0	0	0	0	0	0	0	0	0

		Cohort 1: Newly Determined Advanced BCC Patients (Non-BCCNS) Locally Advanced BCC (laBCC)							
		Vismo (N=115)			No Vismo (N=251)				
	All (N=433)	All (N=115)	Vismo Only (N=102)	Surgery Only (N=6)	Other Therapy (N=7)	All (N=251)	Surgery Only (N=198)	Other Therapy (N=53)	No Treatment (N=67)
Indicate How Response/ Progression was Assessed:									
n	1	1	1	0	0	0	0	0	0
Change in Size of Existing Lesions	0	0	0	0	0	0	0	0	0
Change in Number of Existing Lesions	1(100%)	1(100%)	1(100%)	0	0	0	0	0	0
Development of New BCCs	0	0	0	0	0	0	0	0	0
Progression from Locally Advanced Disease to Metastatic Disease	0	0	0	0	0	0	0	0	0
Development of New Metastases	0	0	0	0	0	0	0	0	0
Other	0	0	0	0	0	0	0	0	0

If progression to metastatic disease or new metastases, indicate site(s)[2]									
Bone	0	0	0	0	0	0	0	0	0
Lymph nodes, Local	0	0	0	0	0	0	0	0	0
Lymph nodes, Distant	0	0	0	0	0	0	0	0	0
Lung	0	0	0	0	0	0	0	0	0
Liver	0	0	0	0	0	0	0	0	0
CNS/Brain	0	0	0	0	0	0	0	0	0
Other	0	0	0	0	0	0	0	0	0
What Methods were Used to Assess Disease Response/ Progression									
n	1	1	1	0	0	0	0	0	0
Diagnostic Image (MRI, CT)	0	0	0	0	0	0	0	0	0
Histopathologic Evaluation	0	0	0	0	0	0	0	0	0
Photography	0	0	0	0	0	0	0	0	0
Clinical Evaluation	1(100%)	1(100%)	1(100%)	0	0	0	0	0	0
Laboratory Evaluation	0	0	0	0	0	0	0	0	0
Other	0	0	0	0	0	0	0	0	0
					Cohort 1: Newly Determined Advanced BCC Patients (Non-BCCNS) Metastatic BCC (mBCC)				
			Vismo (N=2)			No Vismo (N=1)			
	All (N=4)	All (N=2)	Vismo Only (N=2)	Surgery Only (N=0)	Other Therapy (N=0)	All (N=1)	Surgery Only (N=1)	Other Therapy (N=0)	No Treatment (N=1)

Indicate How Response/ Progression was Assessed:									
n	0	0	0	0	0	0	0	0	0
Change in Size of Existing Lesions	0	0	0	0	0	0	0	0	0
Change in Number of Existing Lesions	0	0	0	0	0	0	0	0	0
Development of New BCCs	0	0	0	0	0	0	0	0	0
Progression from Locally Advanced Disease to Metastatic Disease	0	0	0	0	0	0	0	0	0
Development of New Metastases	0	0	0	0	0	0	0	0	0
Other	0	0	0	0	0	0	0	0	0
If progression to metastatic disease or new metastases, indicate site(s)[2]									
Bone	0	0	0	0	0	0	0	0	0
Lymph nodes, Local	0	0	0	0	0	0	0	0	0
Lymph nodes, Distant	0	0	0	0	0	0	0	0	0
Lung	0	0	0	0	0	0	0	0	0
Liver	0	0	0	0	0	0	0	0	0
CNS/Brain	0	0	0	0	0	0	0	0	0
Other	0	0	0	0	0	0	0	0	0

What Methods were Used to Assess Disease Response/ Progression									
n	0	0	0	0	0	0	0	0	0
Diagnostic Image (MRI, CT)	0	0	0	0	0	0	0	0	0
Histopathologic Evaluation	0	0	0	0	0	0	0	0	0
Photography	0	0	0	0	0	0	0	0	0
Clinical Evaluation	0	0	0	0	0	0	0	0	0
Laboratory Evaluation	0	0	0	0	0	0	0	0	0
Other	0	0	0	0	0	0	0	0	0

Note: “Vismo” group is defined as initiating vismodegib less than or equal to 90 days after the date of determination of locally advanced BCC (laBCC) or metastatic BCC (mBCC); “No Vismo” group is defined as not initiating vismodegib (but other BCC treatment) less than or equal to 90 days after the date of determination of laBCC or mBCC; “No Treatment” group is defined as not receiving any BCC treatment less than or equal to 90 days after the date of determination of laBCC or mBCC.

“Vismo Only” and “Surgery Only” are defined as having only 1 of these treatments less than or equal to 90 days after the date of determination of laBCC or mBCC; “Other Therapy” includes individual or combination treatments (e.g., surgery, PDT, ED&C, topical treatment, cryosurgery, systemic treatment, other) initiated less than or equal to 90 days after date of determination of laBCC or mBCC. For example, if patient receives topical treatment followed by vismo followed by surgery, then all 3 treatments must be initiated within the 90 days of the date of determination of disease.

[1] Denominator is based on the number of patients with at least one change of treatment.

[2] Denominator is based on the number of patients with 2 or more changes of treatment.

					Cohort 1: Newly Determined Advanced BCC Patients (Non-BCCNS) Locally Advanced BCC (laBCC)				
		Vismo (N=115)			No Vismo (N=251)				
	All (N=433)	All (N=115)	Vismo Only (N=102)	Surgery Only (N=6)	Other Therapy (N=7)	All (N=251)	Surgery Only (N=198)	Other Therapy (N=53)	No Treatment (N=67)
Patients with at least 1 Stopped or Change in Treatment	389(89.8%)	108(93.9%)	95(93.1%)	6(100%)	7(100%)	247(98.4%)	197(99.5%)	50(94.3%)	34(50.7%)
Patients Had AE Prior to the 1st Stop or Change of Treatment [1]	79(20.3%)	67(62.0%)	67(70.5%)	0	0	4(1.6%)	1(0.5%)	3(6.0%)	8(23.5%)
Reasons for Stopping or Changing First Treatment [1]									
Adverse Event	39(10.0%)	36(33.3%)	35(36.8%)	0	1(14.3%)	0	0	0	3(8.8%)
Treatment Holiday	23(5.9%)	14(13.0%)	13(13.7%)	0	1(14.3%)	9(3.6%)	0	9(18.0%)	0
Disease Progression	1(0.3%)	1(0.9%)	1(1.1%)	0	0	0	0	0	0
Disease Recurrence	2(0.5%)	1(0.9%)	1(1.1%)	0	0	0	0	0	1(2.9%)
Maximum Benefit Achieved	236(60.7%)	21(19.4%)	13(13.7%)	5(83.3%)	3(42.9%)	196(79.4%)	162(82.2%)	34(68.0%)	19(55.9%)

Lost to Follow Up	7(1.8%)	5(4.6%)	5(5.3%)	0	0	1(0.4%)	0	1(2.0%)	1(2.9%)
Physician Decision	57(14.7%)	11(10.2%)	9(9.5%)	1(16.7%)	1(14.3%)	39(15.8%)	34(17.3%)	5(10.0%)	7(20.6%)
Patient Decision	25(6.4%)	19(17.6%)	18(18.9%)	0	1(14.3%)	3(1.2%)	1(0.5%)	2(4.0%)	3(8.8%)
Pregnancy	0	0	0	0	0	0	0	0	0
					Cohort 1: Newly Determined Advanced BCC Patients (Non-BCCNS) Metastatic BCC (mBCC)				
			Vismo (N=2)			No Vismo (N=1)			
	All (N=4)	All (N=2)	Vismo Only (N=2)	Surgery Only (N=0)	Other Therapy (N=0)	All (N=1)	Surgery Only (N=1)	Other Therapy (N=0)	No Treatment (N=1)
Patients with at least 1 Stopped or Change in Treatment	4(100%)	2(100%)	2(100%)	0	0	1(100%)	1(100%)	0	1(100%)
Patients Had AE Prior to the 1st Stop or Change of Treatment [1]	3(75.0%)	2(100%)	2(100%)	0	0	0	0	0	1(100%)
Reasons for Stopping or Changing First Treatment [1]									
Adverse Event	0	0	0	0	0	0	0	0	0
Treatment Holiday	0	0	0	0	0	0	0	0	0
Disease Progression	1(25.0%)	1(50.0%)	1(50.0%)	0	0	0	0	0	0
Disease Recurrence	0	0	0	0	0	0	0	0	0
Maximum Benefit Achieved	1(25.0%)	0	0	0	0	1(100%)	1(100%)	0	0
Lost to Follow Up	0	0	0	0	0	0	0	0	0
Physician Decision	2(50.0%)	1(50.0%)	1(50.0%)	0	0	0	0	0	1(100%)

Patient Decision	0	0	0	0	0	0	0	0	0
Pregnancy	0	0	0	0	0	0	0	0	0
					Cohort 1: Newly Determined Advanced BCC Patients (Non-BCCNS) Locally Advanced BCC (laBCC)				
		Vismo (N=115)			No Vismo (N=251)				
	All (N=433)	All (N=115)	Vismo Only (N=102)	Surgery Only (N=6)	Other Therapy (N=7)	All (N=251)	Surgery Only (N=198)	Other Therapy (N=53)	No Treatment (N=67)
Patients with 2 or more Stops or Changes in Treatment	165(38.1%)	71(61.7%)	59(57.8%)	5(83.3%)	7(100%)	81(32.3%)	58(29.3%)	23(43.4%)	13(19.4%)
Reasons for Stopping or Changing Second Treatment, if Any [2]									
Adverse Event	19(11.5%)	15(21.1%)	12(20.3%)	0	3(42.9%)	3(3.7%)	3(5.2%)	0	1(7.7%)
Treatment Holiday	12(7.3%)	8(11.3%)	7(11.9%)	0	1(14.3%)	4(4.9%)	0	4(17.4%)	0
Disease Progression	4(2.4%)	4(5.6%)	4(6.8%)	0	0	0	0	0	0
Disease Recurrence	2(1.2%)	1(1.4%)	1(1.7%)	0	0	0	0	0	1(7.7%)
Maximum Benefit Achieved	90(54.5%)	23(32.4%)	19(32.2%)	4(80.0%)	0	60(74.1%)	43(74.1%)	17(73.9%)	7(53.8%)
Lost to Follow Up	3(1.8%)	3(4.2%)	3(5.1%)	0	0	0	0	0	0
Physician Decision	30(18.2%)	14(19.7%)	12(20.3%)	1(20.0%)	1(14.3%)	12(14.8%)	10(17.2%)	2(8.7%)	4(30.8%)
Patient Decision	7(4.2%)	4(5.6%)	2(3.4%)	0	2(28.6%)	3(3.7%)	2(3.4%)	1(4.3%)	0
Pregnancy	0	0	0	0	0	0	0	0	0
					Cohort 1: Newly Determined Advanced BCC Patients (Non-BCCNS) Metastatic BCC (mBCC)				
		Vismo (N=2)			No Vismo (N=1)				
	All (N=4)	All (N=2)	Vismo Only (N=2)	Surgery Only (N=0)	Other Therapy (N=0)	All (N=1)	Surgery Only (N=1)	Other Therapy (N=0)	No Treatment (N=1)

Patients with 2 or more Stops or Changes in Treatment	1(25.0%)	1(50.0%)	1(50.0%)	0	0	0	0	0	0
Reasons for Stopping or Changing Second Treatment, if Any [2]									
Adverse Event	0	0	0	0	0	0	0	0	0
Treatment Holiday	0	0	0	0	0	0	0	0	0
Disease Progressio n	0	0	0	0	0	0	0	0	0
Disease Recurrence	0	0	0	0	0	0	0	0	0
Maximum Benefit Achieved	1(100%)	1(100%)	1(100%)	0	0	0	0	0	0
Lost to Follow Up	0	0	0	0	0	0	0	0	0
Physician Decision	0	0	0	0	0	0	0	0	0
Patient Decision	0	0	0	0	0	0	0	0	0
Pregnancy	0	0	0	0	0	0	0	0	0

Note: “Vismo” group is defined as initiating vismodegib less than or equal to 90 days after the date of determination of locally advanced BCC (laBCC) or metastatic BCC (mBCC); “No Vismo” group is defined as not initiating vismodegib (but other BCC treatment) less than or equal to 90 days after the date of determination of laBCC or mBCC; “No Treatment” group is defined as not receiving any BCC treatment less than or equal to 90 days after the date of determination of laBCC or mBCC.

“Vismo Only” and “Surgery Only” are defined as having only 1 of these treatments less than or equal to 90 days after the date of determination of laBCC or mBCC; “Other Therapy” includes individual or combination treatments (e.g., surgery, PDT, ED&C, topical treatment, cryosurgery, systemic treatment, other) initiated less than or equal to 90 days after date of determination of laBCC or mBCC. For example, if patient receives topical treatment followed by vismo followed by surgery, then all 3 treatments must be initiated within the 90 days of the date of determination of disease.

If a patient has more than one event in the same category then the patient is only counted once in that row. Denominator is the big N in the column header. Exposure-adjusted incidence rate (EAIR) per subject-years is number(n) of subjects reporting the event divided by subject-years [patient’s AE start date (use initial aBCC diagnosis for Cohort 1 and Cohort 3 patients not previously enrolled in GNE; use ICD for Cohort 2 and Cohort 3 patients previously enrolled in GNE) to the first event start date for subjects reporting the event, up to termination or data cut for other subjects].

[1] The AEs with 'Action taken with suspected causal treatment' as permanently discontinued.

[2] Other should only contain non-AEs of Interest that either resulted in a treatment change/stop or were SAE.

							Cohort 1: Newly Determined Advanced BCC Patients (Non-BCCNS)							
							Locally Advanced BCC (laBCC)							
							Vismo (N=115)							
	All (N=433)			All (N=115)			Vismo Only (N=102)			Surgery Only (N=6)			Other Therapy (N=7)	
	n (%)	Exposure Adjusted Incidence Rate (Subj/yr)		n (%)	Exposure Adjusted Incidence Rate (Subj/yr)		n (%)	Exposure Adjusted Incidence Rate (Subj/yr)		n (%)	Exposure Adjusted Incidence Rate (Subj/yr)		n (%)	Exposure Adjusted Incidence Rate (Subj/yr)
Any AE/ SAE														
All AEs/SAEs	193 (44.6%)	0.36		104 (90.4%)	2.35		93 (91.2%)	2.69		4 (66.7%)	0.68		7 (100.0%)	1.87
SAEs	78 (18.0%)	0.10		29 (25.2%)	0.13		27 (26.5%)	0.15		0	0.00		2 (28.6%)	0.13
Any AE Leading to Early Treatment Discontinuation [1]														
All AEs/SAEs	31 (7.2%)	0.04		24 (20.9%)	0.12		22 (21.6%)	0.12		0	0.00		2 (28.6%)	0.19
SAEs	8 (1.8%)	0.01		3 (2.6%)	0.01		3 (2.9%)	0.01		0	0.00		0	0.00

Ageusia or dysgeusia														
All AEs/SAEs	79 (18.2%)	0.11		71 (61.7%)	0.74		64 (62.7%)	0.78		3 (50.0%)	0.36		4 (57.1%)	0.64
SAEs	0	0.00		0	0.00		0	0.00		0	0.00		0	0.00
Ageusia														
All AEs/SAEs	21 (4.8%)	0.03		20 (17.4%)	0.10		19 (18.6%)	0.11		1 (16.7%)	0.07		0	0.00
SAEs	0	0.00		0	0.00		0	0.00		0	0.00		0	0.00
Dysgeusia														
All AEs/SAEs	63 (14.5%)	0.09		56 (48.7%)	0.44		50 (49.0%)	0.45		2 (33.3%)	0.18		4 (57.1%)	0.64
SAEs	0	0.00		0	0.00		0	0.00		0	0.00		0	0.00

						Cohort 1: Newly Determined Advanced BCC Patients (Non-BCCNS)					
						Locally Advanced BCC (laBCC)					
				No Vismo (N=251)							
	All (N=251)			Surgery Only (N=198)			Other Therapy (N=53)			No Treatment (N=67)	
	n (%)	Exposure Adjusted Incidence Rate (Subj/yr)		n (%)	Exposure Adjusted Incidence Rate (Subj/yr)		n (%)	Exposure Adjusted Incidence Rate (Subj/yr)		n (%)	Exposure Adjusted Incidence Rate (Subj/yr)
Any AE/SAE											
All AEs/ SAEs	65 (25.9%)	0.16		49 (24.7%)	0.16		16 (30.2%)	0.17		24 (35.8%)	0.28
SAEs	35 (13.9%)	0.08		27 (13.6%)	0.08		8 (15.1%)	0.07		14 (20.9%)	0.13
Any AE Leading to Early Treatment Discontinuation [1]											
All AEs/ SAEs	6 (2.4%)	0.01		6 (3.0%)	0.02		0	0.00		1 (1.5%)	0.01
SAEs	4 (1.6%)	0.01		4 (2.0%)	0.01		0	0.00		1 (1.5%)	0.01
Ageusia or dysgeusia											

All AEs/ SAEs	4 (1.6%)	0.01		3 (1.5%)	0.01		1 (1.9%)	0.01		4 (6.0%)	0.04
SAEs	0	0.00		0	0.00		0	0.00		0	0.00
Ageusia											
All AEs/SAEs	1 (0.4%)	0.00		1 (0.5%)	0.00		0	0.00		0	0.00
SAEs	0	0.00		0	0.00		0	0.00		0	0.00
Dysgeusia											
All AEs/SAEs	3 (1.2%)	0.01		2 (1.0%)	0.01		1 (1.9%)	0.01		4 (6.0%)	0.04
SAEs	0	0.00		0	0.00		0	0.00		0	0.00

							Cohort 1: Newly Determined Advanced BCC Patients (Non-BCCNS)							
							Metastatic BCC (mBCC)							
							Vismo (N=2)							
	All (N=4)			All (N=2)			Vismo Only (N=2)			Surgery Only (N=0)			Other Therapy (N=0)	
	n (%)	Exposure Adjusted Incidence Rate (Subj/ yr)		n (%)	Exposure Adjusted Incidence Rate (Subj/ yr)		n (%)	Exposure Adjusted Incidence Rate (Subj/ yr)		n (%)	Exposure Adjusted Incidence Rate (Subj/ yr)		n (%)	Exposure Adjusted Incidence Rate (Subj/ yr)
Any AE/ SAE														
All AEs/SAEs	3 (75.0%)	0.64		2 (100.0%)	8.80		2 (100.0%)	8.80		0	0.00		0	0.00
SAEs	1 (25.0%)	0.12		0	0.00		0	0.00		0	0.00		0	0.00
Any AE Leading to Early Treatment Discontinuation [1]														
All AEs/SAEs	0	0.00		0	0.00		0	0.00		0	0.00		0	0.00
SAEs	0	0.00		0	0.00		0	0.00		0	0.00		0	0.00
Ageusia or dysgeusia														

All AEs/SAEs	3 (75.0%)	0.64		2 (100.0%)	8.80		2 (100.0%)	8.80		0	0.00		0	0.00
SAEs	0	0.00		0	0.00		0	0.00		0	0.00		0	0.00
Ageusia														
All AEs/SAEs	0	0.00		0	0.00		0	0.00		0	0.00		0	0.00
SAEs	0	0.00		0	0.00		0	0.00		0	0.00		0	0.00
Dysgeusia														
All AEs/SAEs	3 (75.0%)	0.64		2 (100.0%)	8.80		2 (100.0%)	8.80		0	0.00		0	0.00
SAEs	0	0.00		0	0.00		0	0.00		0	0.00		0	0.00

						Cohort 1: Newly Determined Advanced BCC Patients (Non-BCCNS)					
						Metastatic BCC (mBCC)					
				No Vismo (N=1)							
	All (N=1)			Surgery Only (N=1)			Other Therapy (N=0)			No Treatment (N=1)	
	n (%)	Exposure Adjusted Incidence Rate (Subj/yr)		n (%)	Exposure Adjusted Incidence Rate (Subj/yr)		n (%)	Exposure Adjusted Incidence Rate (Subj/yr)		n (%)	Exposure Adjusted Incidence Rate (Subj/yr)
Any AE/SAE											
All AEs/ SAEs	0	0.00		0	0.00		0	0.00		1 (100.0%)	0.49
SAEs	0	0.00		0	0.00		0	0.00		1 (100.0%)	0.35
Any AE Leading to Early Treatment Discontinuation [1]											
All AEs/ SAEs	0	0.00		0	0.00		0	0.00		0	0.00
SAEs	0	0.00		0	0.00		0	0.00		0	0.00
Ageusia or dysgeusia											
All AEs/ SAEs	0	0.00		0	0.00		0	0.00		1 (100.0%)	0.49
SAEs	0	0.00		0	0.00		0	0.00		0	0.00
Ageusia											

All AEs/SAEs	0	0.00		0	0.00		0	0.00		0	0.00
SAEs	0	0.00		0	0.00		0	0.00		0	0.00
Dysgeusia											
All AEs/SAEs	0	0.00		0	0.00		0	0.00		1 (100.0%)	0.49
SAEs	0	0.00		0	0.00		0	0.00		0	0.00

							Cohort 1: Newly Determined Advanced BCC Patients (Non-BCCNS)							
							Locally Advanced BCC (laBCC)							
							Vismo (N=115)							
	All (N=433)			All (N=115)			Vismo Only (N=102)			Surgery Only (N=6)			Other Therapy (N=7)	
	n (%)	Exposure Adjusted Incidence Rate (Subj/yr)		n (%)	Exposure Adjusted Incidence Rate (Subj/yr)		n (%)	Exposure Adjusted Incidence Rate (Subj/yr)		n (%)	Exposure Adjusted Incidence Rate (Subj/yr)		n (%)	Exposure Adjusted Incidence Rate (Subj/yr)
GI Event														
All AEs/SAEs	15 (3.5%)	0.02		11 (9.6%)	0.05		11 (10.8%)	0.06		0	0.00		0	0.00
SAEs	3 (0.7%)	0.00		1 (0.9%)	0.00		1 (1.0%)	0.00		0	0.00		0	0.00
Abdominal Pain (Grade >=2)														
All AEs/SAEs	3 (0.7%)	0.00		1 (0.9%)	0.00		1 (1.0%)	0.00		0	0.00		0	0.00
SAEs	1 (0.2%)	0.00		0	0.00		0	0.00		0	0.00		0	0.00
Constipation (Grade >=2)														
All AEs/SAEs	3 (0.7%)	0.00		3 (2.6%)	0.01		3 (2.9%)	0.01		0	0.00		0	0.00
SAEs	0	0.00		0	0.00		0	0.00		0	0.00		0	0.00
Diarrhea (Grade >=2)														
All AEs/SAEs	3 (0.7%)	0.00		2 (1.7%)	0.01		2 (2.0%)	0.01		0	0.00		0	0.00

SAEs	1 (0.2%)	0.00		1 (0.9%)	0.00		1 (1.0%)	0.00		0	0.00		0	0.00
Elevated Liver Enzymes (Grade >=2)														
All AEs/SAEs	1 (0.2%)	0.00		1 (0.9%)	0.00		1 (1.0%)	0.00		0	0.00		0	0.00
SAEs	0	0.00		0	0.00		0	0.00		0	0.00		0	0.00
Nausea (Grade >=2)														
All AEs/SAEs	7 (1.6%)	0.01		7 (6.1%)	0.03		7 (6.9%)	0.04		0	0.00		0	0.00
SAEs	0	0.00		0	0.00		0	0.00		0	0.00		0	0.00
Vomiting (Grade >=2)														
All AEs/SAEs	5 (1.2%)	0.01		4 (3.5%)	0.02		4 (3.9%)	0.02		0	0.00		0	0.00
SAEs	1 (0.2%)	0.00		0	0.00		0	0.00		0	0.00		0	0.00

						Cohort 1: Newly Determined Advanced BCC Patients (Non-BCCNS)					
						Locally Advanced BCC (laBCC)					
				No Vismo (N=251)							
	All (N=251)				Surgery Only (N=198)			Other Therapy (N=53)			No Treatment (N=67)
	n (%)	Exposure Adjusted Incidence Rate (Subj/yr)		n (%)	Exposure Adjusted Incidence Rate (Subj/yr)		n (%)	Exposure Adjusted Incidence Rate (Subj/yr)		n (%)	Exposure Adjusted Incidence Rate (Subj/yr)
GI Event											
All AEs/ SAEs	3 (1.2%)	0.01		2 (1.0%)	0.01		1 (1.9%)	0.01		1 (1.5%)	0.01
SAEs	2 (0.8%)	0.00		2 (1.0%)	0.01		0	0.00		0	0.00
Abdominal Pain (Grade >=2)											
All AEs/SAEs	2 (0.8%)	0.00		1 (0.5%)	0.00		1 (1.9%)	0.01		0	0.00
SAEs	1 (0.4%)	0.00		1 (0.5%)	0.00		0	0.00		0	0.00

Constipation (Grade >=2)											
All AEs/SAEs	0	0.00		0	0.00		0	0.00		0	0.00
SAEs	0	0.00		0	0.00		0	0.00		0	0.00
Diarrhea (Grade >=2)											
All AEs/SAEs	0	0.00		0	0.00		0	0.00		1 (1.5%)	0.01
SAEs	0	0.00		0	0.00		0	0.00		0	0.00
Elevated Liver Enzymes (Grade >=2)											
All AEs/SAEs	0	0.00		0	0.00		0	0.00		0	0.00
SAEs	0	0.00		0	0.00		0	0.00		0	0.00
Nausea (Grade >=2)											
All AEs/SAEs	0	0.00		0	0.00		0	0.00		0	0.00
SAEs	0	0.00		0	0.00		0	0.00		0	0.00
Vomiting (Grade >=2)											
All AEs/SAEs	1 (0.4%)	0.00		1 (0.5%)	0.00		0	0.00		0	0.00
SAEs	1 (0.4%)	0.00		1 (0.5%)	0.00		0	0.00		0	0.00

							Cohort 1: Newly Determined Advanced BCC Patients (Non-BCCNS)							
							Metastatic BCC (mBCC)							
							Vismo (N=2)							
	All (N=4)			All (N=2)			Vismo Only (N=2)			Surgery Only (N=0)			Other Therapy (N=0)	
	n (%)	Exposure Adjusted Incidence Rate (Subj/yr)		n (%)	Exposure Adjusted Incidence Rate (Subj/yr)		n (%)	Exposure Adjusted Incidence Rate (Subj/yr)		n (%)	Exposure Adjusted Incidence Rate (Subj/yr)		n (%)	Exposure Adjusted Incidence Rate (Subj/yr)
GI Event														
All AEs/SAEs	0	0.00		0	0.00		0	0.00		0	0.00		0	0.00
SAEs	0	0.00		0	0.00		0	0.00		0	0.00		0	0.00

Abdominal Pain (Grade >=2)														
All AEs/SAEs	0	0.00		0	0.00		0	0.00		0	0.00		0	0.00
SAEs	0	0.00		0	0.00		0	0.00		0	0.00		0	0.00
Constipation (Grade >=2)														
All AEs/SAEs	0	0.00		0	0.00		0	0.00		0	0.00		0	0.00
SAEs	0	0.00		0	0.00		0	0.00		0	0.00		0	0.00
Diarrhea (Grade >=2)														
All AEs/SAEs	0	0.00		0	0.00		0	0.00		0	0.00		0	0.00
SAEs	0	0.00		0	0.00		0	0.00		0	0.00		0	0.00
Elevated Liver Enzymes (Grade >=2)														
All AEs/SAEs	0	0.00		0	0.00		0	0.00		0	0.00		0	0.00
SAEs	0	0.00		0	0.00		0	0.00		0	0.00		0	0.00
Nausea (Grade >=2)														
All AEs/SAEs	0	0.00		0	0.00		0	0.00		0	0.00		0	0.00
SAEs	0	0.00		0	0.00		0	0.00		0	0.00		0	0.00
Vomiting (Grade >=2)														
All AEs/SAEs	0	0.00		0	0.00		0	0.00		0	0.00		0	0.00
SAEs	0	0.00		0	0.00		0	0.00		0	0.00		0	0.00
							Cohort 1: Newly Determined Advanced BCC Patients (Non-BCCNS)							
							Metastatic BCC (mBCC)							

					No Vismo (N=1)						
	All (N=1)				Surgery Only (N=1)			Other Therapy (N=0)			No Treatment (N=1)
	n (%)	Exposure Adjusted Incidence Rate (Subj/yr)		n (%)	Exposure Adjusted Incidence Rate (Subj/yr)		n (%)	Exposure Adjusted Incidence Rate (Subj/yr)		n (%)	Exposure Adjusted Incidence Rate (Subj/yr)
GI Event											
All AEs/ SAEs	0	0.00		0	0.00		0	0.00		0	0.00
SAEs	0	0.00		0	0.00		0	0.00		0	0.00
Abdominal Pain (Grade >=2)											
All AEs/SAEs	0	0.00		0	0.00		0	0.00		0	0.00
SAEs	0	0.00		0	0.00		0	0.00		0	0.00
Constipation (Grade >=2)											
All AEs/SAEs	0	0.00		0	0.00		0	0.00		0	0.00
SAEs	0	0.00		0	0.00		0	0.00		0	0.00
Diarrhea (Grade >=2)											
All AEs/SAEs	0	0.00		0	0.00		0	0.00		0	0.00
SAEs	0	0.00		0	0.00		0	0.00		0	0.00
Elevated Liver Enzymes (Grade >=2)											
All AEs/SAEs	0	0.00		0	0.00		0	0.00		0	0.00
SAEs	0	0.00		0	0.00		0	0.00		0	0.00
Nausea (Grade >=2)											
All AEs/SAEs	0	0.00		0	0.00		0	0.00		0	0.00
SAEs	0	0.00		0	0.00		0	0.00		0	0.00
Vomiting (Grade >=2)											
All AEs/SAEs	0	0.00		0	0.00		0	0.00		0	0.00

SAEs	0	0.00		0	0.00		0	0.00		0	0.00

							Cohort 1: Newly Determined Advanced BCC Patients (Non-BCCNS)							
							Locally Advanced BCC (laBCC)							
							Vismo (N=115)							
	All (N=433)			All (N=115)			Vismo Only (N=102)			Surgery Only (N=6)			Other Therapy (N=7)	
	n (%)	Exposure Adjusted Incidence Rate (Subj/ yr)		n (%)	Exposure Adjusted Incidence Rate (Subj/ yr)		n (%)	Exposure Adjusted Incidence Rate (Subj/ yr)		n (%)	Exposure Adjusted Incidence Rate (Subj/ yr)		n (%)	Exposure Adjusted Incidence Rate (Subj/ yr)
Alopecia														
All AEs/SAEs	64 (14.8%)	0.09		58 (50.4%)	0.45		52 (51.0%)	0.46		3 (50.0%)	0.35		3 (42.9%)	0.38
SAEs	0	0.00		0	0.00		0	0.00		0	0.00		0	0.00
Amenorrhea														
All AEs/SAEs	1 (0.2%)	0.00		1 (0.9%)	0.00		1 (1.0%)	0.00		0	0.00		0	0.00
SAEs	0	0.00		0	0.00		0	0.00		0	0.00		0	0.00
CVD Event														
All AEs/SAEs	1 (0.2%)	0.00		0	0.00		0	0.00		0	0.00		0	0.00
SAEs	0	0.00		0	0.00		0	0.00		0	0.00		0	0.00
Congestive Heart Failure														
All AEs/SAEs	0	0.00		0	0.00		0	0.00		0	0.00		0	0.00
SAEs	0	0.00		0	0.00		0	0.00		0	0.00		0	0.00
Deep Vein Thrombosis														
All AEs/SAEs	0	0.00		0	0.00		0	0.00		0	0.00		0	0.00
SAEs	0	0.00		0	0.00		0	0.00		0	0.00		0	0.00
Pulmonary Emboli														

All AEs/SAEs	1 (0.2%)	0.00		0	0.00		0	0.00		0	0.00		0	0.00
SAEs	0	0.00		0	0.00		0	0.00		0	0.00		0	0.00
Stroke														
All AEs/SAEs	0	0.00		0	0.00		0	0.00		0	0.00		0	0.00
SAEs	0	0.00		0	0.00		0	0.00		0	0.00		0	0.00
Fatigue (Grade >=2)														
All AEs/SAEs	12 (2.8%)	0.01		10 (8.7%)	0.05		10 (9.8%)	0.05		0	0.00		0	0.00
SAEs	0	0.00		0	0.00		0	0.00		0	0.00		0	0.00

	Cohort 1: Newly Determined Advanced BCC Patients (Non-BCCNS)										
	Locally Advanced BCC (laBCC)										
	No Vismo (N=251)										
	All (N=251)			Surgery Only (N=198)			Other Therapy (N=53)			No Treatment (N=67)	
	n (%)	Exposure Adjusted Incidence Rate (Subj/yr)		n (%)	Exposure Adjusted Incidence Rate (Subj/yr)		n (%)	Exposure Adjusted Incidence Rate (Subj/yr)		n (%)	Exposure Adjusted Incidence Rate (Subj/yr)
Alopecia											
All AEs/ SAEs	2 (0.8%)	0.00		2 (1.0%)	0.01		0	0.00		4 (6.0%)	0.03
SAEs	0	0.00		0	0.00		0	0.00		0	0.00
Amenorrhea											
All AEs/ SAEs	0	0.00		0	0.00		0	0.00		0	0.00
SAEs	0	0.00		0	0.00		0	0.00		0	0.00
CVD Event											
All AEs/ SAEs	1 (0.4%)	0.00		1 (0.5%)	0.00		0	0.00		0	0.00
SAEs	0	0.00		0	0.00		0	0.00		0	0.00
Congestive Heart Failure											
All AEs/SAEs	0	0.00		0	0.00		0	0.00		0	0.00
SAEs	0	0.00		0	0.00		0	0.00		0	0.00

Deep Vein Thrombosis											
All AEs/SAEs	0	0.00		0	0.00		0	0.00		0	0.00
SAEs	0	0.00		0	0.00		0	0.00		0	0.00
Pulmonary Emboli											
All AEs/SAEs	1 (0.4%)	0.00		1 (0.5%)	0.00		0	0.00		0	0.00
SAEs	0	0.00		0	0.00		0	0.00		0	0.00
Stroke											
All AEs/SAEs	0	0.00		0	0.00		0	0.00		0	0.00
SAEs	0	0.00		0	0.00		0	0.00		0	0.00
Fatigue (Grade >=2)											
All AEs/ SAEs	2 (0.8%)	0.00		0	0.00		2 (3.8%)	0.02		0	0.00
SAEs	0	0.00		0	0.00		0	0.00		0	0.00

							Cohort 1: Newly Determined Advanced BCC Patients (Non-BCCNS)							
							Metastatic BCC (mBCC)							
							Vismo (N=2)							
	All (N=4)			All (N=2)			Vismo Only (N=2)			Surgery Only (N=0)			Other Therapy (N=0)	
	n (%)	Exposure Adjusted Incidence Rate (Subj/ yr)		n (%)	Exposure Adjusted Incidence Rate (Subj/ yr)		n (%)	Exposure Adjusted Incidence Rate (Subj/ yr)		n (%)	Exposure Adjusted Incidence Rate (Subj/ yr)		n (%)	Exposure Adjusted Incidence Rate (Subj/ yr)
Alopecia														
All AEs/SAEs	1 (25.0%)	0.11		1 (50.0%)	0.44		1 (50.0%)	0.44		0	0.00		0	0.00
SAEs	0	0.00		0	0.00		0	0.00		0	0.00		0	0.00
Amenorrhea														
All AEs/SAEs	0	0.00		0	0.00		0	0.00		0	0.00		0	0.00
SAEs	0	0.00		0	0.00		0	0.00		0	0.00		0	0.00
CVD Event														

All AEs/SAEs	0	0.00		0	0.00		0	0.00		0	0.00		0	0.00
SAEs	0	0.00		0	0.00		0	0.00		0	0.00		0	0.00
Congestive Heart Failure														
All AEs/SAEs	0	0.00		0	0.00		0	0.00		0	0.00		0	0.00
SAEs	0	0.00		0	0.00		0	0.00		0	0.00		0	0.00
Deep Vein Thrombosis														
All AEs/SAEs	0	0.00		0	0.00		0	0.00		0	0.00		0	0.00
SAEs	0	0.00		0	0.00		0	0.00		0	0.00		0	0.00
Pulmonary Emboli														
All AEs/SAEs	0	0.00		0	0.00		0	0.00		0	0.00		0	0.00
SAEs	0	0.00		0	0.00		0	0.00		0	0.00		0	0.00
Stroke														
All AEs/SAEs	0	0.00		0	0.00		0	0.00		0	0.00		0	0.00
SAEs	0	0.00		0	0.00		0	0.00		0	0.00		0	0.00
Fatigue (Grade >=2)														
All AEs/SAEs	0	0.00		0	0.00		0	0.00		0	0.00		0	0.00
SAEs	0	0.00		0	0.00		0	0.00		0	0.00		0	0.00

							Cohort 1: Newly Determined Advanced BCC Patients (Non-BCCNS)				
							Metastatic BCC (mBCC)				
				No Vismo (N=1)							
	All (N=1)				Surgery Only (N=1)			Other Therapy (N=0)			No Treatment (N=1)
	n (%)	Exposure Adjusted Incidence Rate (Subj/yr)		n (%)	Exposure Adjusted Incidence Rate (Subj/yr)		n (%)	Exposure Adjusted Incidence Rate (Subj/yr)		n (%)	Exposure Adjusted Incidence Rate (Subj/yr)
Alopecia											
All AEs/ SAEs	0	0.00		0	0.00		0	0.00		0	0.00

SAEs	0	0.00		0	0.00		0	0.00		0	0.00
Amenorrhea											
All AEs/ SAEs	0	0.00		0	0.00		0	0.00		0	0.00
SAEs	0	0.00		0	0.00		0	0.00		0	0.00
CVD Event											
All AEs/ SAEs	0	0.00		0	0.00		0	0.00		0	0.00
SAEs	0	0.00		0	0.00		0	0.00		0	0.00
Congestive Heart Failure											
All AEs/SAEs	0	0.00		0	0.00		0	0.00		0	0.00
SAEs	0	0.00		0	0.00		0	0.00		0	0.00
Deep Vein Thrombosis											
All AEs/SAEs	0	0.00		0	0.00		0	0.00		0	0.00
SAEs	0	0.00		0	0.00		0	0.00		0	0.00
Pulmonary Emboli											
All AEs/SAEs	0	0.00		0	0.00		0	0.00		0	0.00
SAEs	0	0.00		0	0.00		0	0.00		0	0.00
Stroke											
All AEs/SAEs	0	0.00		0	0.00		0	0.00		0	0.00
SAEs	0	0.00		0	0.00		0	0.00		0	0.00
Fatigue (Grade >=2)											
All AEs/ SAEs	0	0.00		0	0.00		0	0.00		0	0.00
SAEs	0	0.00		0	0.00		0	0.00		0	0.00
						Cohort 1: Newly Determined Advanced BCC Patients (Non-BCCNS)					
						Locally Advanced BCC (laBCC)					
						Vismo (N=115)					
	All (N=433)			All (N=115)		Vismo Only (N=102)		Surgery Only (N=6)			Other Therapy (N=7)

	n (%)	Exposure Adjusted Incidence Rate (Subj/yr)		n (%)	Exposure Adjusted Incidence Rate (Subj/yr)		n (%)	Exposure Adjusted Incidence Rate (Subj/yr)		n (%)	Exposure Adjusted Incidence Rate (Subj/yr)		n (%)	Exposure Adjusted Incidence Rate (Subj/yr)
Muscle Spasms														
All AEs/SAEs	74 (17.1%)	0.10		66 (57.4%)	0.60		61 (59.8%)	0.68		2 (33.3%)	0.18		3 (42.9%)	0.37
SAEs	1 (0.2%)	0.00		0	0.00		0	0.00		0	0.00		0	0.00
Myocardial Infarction														
All AEs/SAEs	1 (0.2%)	0.00		0	0.00		0	0.00		0	0.00		0	0.00
SAEs	1 (0.2%)	0.00		0	0.00		0	0.00		0	0.00		0	0.00
Pregnancy														
All AEs/SAEs	0	0.00		0	0.00		0	0.00		0	0.00		0	0.00
SAEs	0	0.00		0	0.00		0	0.00		0	0.00		0	0.00
Weight Loss, Unintentional														
All AEs/SAEs	35 (8.1%)	0.04		29 (25.2%)	0.15		28 (27.5%)	0.17		0	0.00		1 (14.3%)	0.07
SAEs	1 (0.2%)	0.00		1 (0.9%)	0.00		1 (1.0%)	0.00		0	0.00		0	0.00
Squamous Cell Carcinomas														
All AEs/SAEs	55 (12.7%)	0.07		14 (12.2%)	0.06		12 (11.8%)	0.06		1 (16.7%)	0.06		1 (14.3%)	0.07
SAEs	7 (1.6%)	0.01		0	0.00		0	0.00		0	0.00		0	0.00

Arthralgia														
All AEs/SAEs	15 (3.5%)	0.02		13 (11.3%)	0.06		12 (11.8%)	0.06		1 (16.7%)	0.07		0	0.00
SAEs	0	0.00		0	0.00		0	0.00		0	0.00		0	0.00
Other [2]														
All AEs/SAEs	81 (18.7%)	0.10		37 (32.2%)	0.19		35 (34.3%)	0.21		0	0.00		2 (28.6%)	0.13
SAEs	72 (16.6%)	0.09		29 (25.2%)	0.13		27 (26.5%)	0.15		0	0.00		2 (28.6%)	0.13

						Cohort 1: Newly Determined Advanced BCC Patients (Non-BCCNS)					
						Locally Advanced BCC (laBCC)					
				No Vismo (N=251)							
	All (N=251)			Surgery Only (N=198)			Other Therapy (N=53)			No Treatment (N=67)	
	n (%)	Exposure Adjusted Incidence Rate (Subj/yr)		n (%)	Exposure Adjusted Incidence Rate (Subj/yr)		n (%)	Exposure Adjusted Incidence Rate (Subj/yr)		n (%)	Exposure Adjusted Incidence Rate (Subj/yr)
Muscle Spasms											
All AEs/ SAEs	2 (0.8%)	0.00		1 (0.5%)	0.00		1 (1.9%)	0.01		6 (9.0%)	0.06
SAEs	1 (0.4%)	0.00		1 (0.5%)	0.00		0	0.00		0	0.00
Myocardial Infarction											
All AEs/ SAEs	0	0.00		0	0.00		0	0.00		1 (1.5%)	0.01
SAEs	0	0.00		0	0.00		0	0.00		1 (1.5%)	0.01
Pregnancy											
All AEs/ SAEs	0	0.00		0	0.00		0	0.00		0	0.00
SAEs	0	0.00		0	0.00		0	0.00		0	0.00
Weight Loss, Unintentional											
All AEs/ SAEs	6 (2.4%)	0.01		3 (1.5%)	0.01		3 (5.7%)	0.03		0	0.00
SAEs	0	0.00		0	0.00		0	0.00		0	0.00

Squamous Cell Carcinomas											
All AEs/ SAEs	31 (12.4%)	0.07		23 (11.6%)	0.07		8 (15.1%)	0.08		10 (14.9%)	0.10
SAEs	6 (2.4%)	0.01		5 (2.5%)	0.01		1 (1.9%)	0.01		1 (1.5%)	0.01
Arthralgia											
All AEs/ SAEs	1 (0.4%)	0.00		1 (0.5%)	0.00		0	0.00		1 (1.5%)	0.01
SAEs	0	0.00		0	0.00		0	0.00		0	0.00
Other [2]											
All AEs/ SAEs	31 (12.4%)	0.07		24 (12.1%)	0.07		7 (13.2%)	0.06		13 (19.4%)	0.12
SAEs	31 (12.4%)	0.07		24 (12.1%)	0.07		7 (13.2%)	0.06		12 (17.9%)	0.11

							Cohort 1: Newly Determined Advanced BCC Patients (Non-BCCNS)							
							Metastatic BCC (mBCC)							
							Vismo (N=2)							
	All (N=4)			All (N=2)			Vismo Only (N=2)			Surgery Only (N=0)			Other Therapy (N=0)	
	n (%)	Exposure Adjusted Incidence Rate (Subj/ yr)		n (%)	Exposure Adjusted Incidence Rate (Subj/ yr)		n (%)	Exposure Adjusted Incidence Rate (Subj/ yr)		n (%)	Exposure Adjusted Incidence Rate (Subj/ yr)		n (%)	Exposure Adjusted Incidence Rate (Subj/ yr)
Muscle Spasms														
All AEs/SAEs	2 (50.0%)	0.30		1 (50.0%)	0.46		1 (50.0%)	0.46		0	0.00		0	0.00
SAEs	0	0.00		0	0.00		0	0.00		0	0.00		0	0.00
Myocardial Infarction														
All AEs/SAEs	0	0.00		0	0.00		0	0.00		0	0.00		0	0.00
SAEs	0	0.00		0	0.00		0	0.00		0	0.00		0	0.00
Pregnancy														

All AEs/SAEs	0	0.00		0	0.00		0	0.00		0	0.00		0	0.00
SAEs	0	0.00		0	0.00		0	0.00		0	0.00		0	0.00
Weight Loss, Unintentional														
All AEs/SAEs	1 (25.0%)	0.12		0	0.00		0	0.00		0	0.00		0	0.00
SAEs	0	0.00		0	0.00		0	0.00		0	0.00		0	0.00
Squamous Cell Carcinomas														
All AEs/SAEs	0	0.00		0	0.00		0	0.00		0	0.00		0	0.00
SAEs	0	0.00		0	0.00		0	0.00		0	0.00		0	0.00
Arthralgia														
All AEs/SAEs	1 (25.0%)	0.11		1 (50.0%)	0.59		1 (50.0%)	0.59		0	0.00		0	0.00
SAEs	0	0.00		0	0.00		0	0.00		0	0.00		0	0.00
Other [2]														
All AEs/SAEs	1 (25.0%)	0.12		0	0.00		0	0.00		0	0.00		0	0.00
SAEs	1 (25.0%)	0.12		0	0.00		0	0.00		0	0.00		0	0.00

						Cohort 1: Newly Determined Advanced BCC Patients (Non-BCCNS)					
						Metastatic BCC (mBCC)					
				No Vismo (N=1)							
	All (N=1)			Surgery Only (N=1)			Other Therapy (N=0)			No Treatment (N=1)	
	n (%)	Exposure Adjusted Incidence Rate (Subj/yr)		n (%)	Exposure Adjusted Incidence Rate (Subj/yr)		n (%)	Exposure Adjusted Incidence Rate (Subj/yr)		n (%)	Exposure Adjusted Incidence Rate (Subj/yr)
Muscle Spasms											
All AEs/ SAEs	0	0.00		0	0.00		0	0.00		1 (100.0%)	0.48

SAEs	0	0.00		0	0.00		0	0.00		0	0.00
Myocardial Infarction											
All AEs/ SAEs	0	0.00		0	0.00		0	0.00		0	0.00
SAEs	0	0.00		0	0.00		0	0.00		0	0.00
Pregnancy											
All AEs/ SAEs	0	0.00		0	0.00		0	0.00		0	0.00
SAEs	0	0.00		0	0.00		0	0.00		0	0.00
Weight Loss, Unintentional											
All AEs/ SAEs	0	0.00		0	0.00		0	0.00		1 (100.0%)	0.42
SAEs	0	0.00		0	0.00		0	0.00		0	0.00
Squamous Cell Carcinomas											
All AEs/ SAEs	0	0.00		0	0.00		0	0.00		0	0.00
SAEs	0	0.00		0	0.00		0	0.00		0	0.00
Arthralgia											
All AEs/ SAEs	0	0.00		0	0.00		0	0.00		0	0.00
SAEs	0	0.00		0	0.00		0	0.00		0	0.00
Other [2]											
All AEs/ SAEs	0	0.00		0	0.00		0	0.00		1 (100.0%)	0.35
SAEs	0	0.00		0	0.00		0	0.00		1 (100.0%)	0.35
